# Genome-wide identification and spatiotemporal expression analysis of cadherin superfamily members in echinoderms

**DOI:** 10.1186/s13227-023-00219-7

**Published:** 2023-12-20

**Authors:** Macie M. Chess, William Douglas, Josiah Saunders, Charles A. Ettensohn

**Affiliations:** Department of Biological Sciences, Carnegie Mellon University, Pittsburgh, PA 15213 USA

**Keywords:** Cadherin, Catenin, Deuterostome evolution, Echinoderm, Protocadherin, Sea urchin development

## Abstract

**Background:**

Cadherins are calcium-dependent transmembrane cell–cell adhesion proteins that are essential for metazoan development. They consist of three subfamilies: classical cadherins, which bind catenin, protocadherins, which contain 6–7 calcium-binding repeat domains, and atypical cadherins. Their functions include forming adherens junctions, establishing planar cell polarity (PCP), and regulating cell shape, proliferation, and migration. Because they are basal deuterostomes, echinoderms provide important insights into bilaterian evolution, but their only well-characterized cadherin is G-cadherin, a classical cadherin that is expressed by many embryonic epithelia. We aimed to better characterize echinoderm cadherins by conducting phylogenetic analyses and examining the spatiotemporal expression patterns of cadherin-encoding genes during *Strongylocentrotus purpuratus* development.

**Results:**

Our phylogenetic analyses conducted on two echinoid, three asteroid, and one crinoid species identified ten echinoderm cadherins, including one deuterostome-specific ortholog, cadherin-23, and an echinoderm-specific atypical cadherin that possibly arose in an echinoid-asteroid ancestor. Catenin-binding domains in dachsous-2 orthologs were found to be a deuterostome-specific innovation that was selectively lost in mouse, while those in Fat4 orthologs appeared to be Ambulacraria-specific and were selectively lost in non-crinoid echinoderms. The identified suite of echinoderm cadherins lacks vertebrate-specific innovations but contains two proteins that are present in protostomes and absent from mouse. The spatiotemporal expression patterns of four embryonically expressed cadherins (fat atypical cadherins 1 and 4, dachsous-2, and protocadherin-9) were dynamic and mirrored the expression pattern of Frizzled 5/8, a non-canonical Wnt PCP pathway receptor protein essential for archenteron morphogenesis.

**Conclusions:**

The echinoderm cadherin toolkit is more similar to that of an ancient bilaterian predating protostomes and deuterostomes than it is to the suite of cadherins found in extant vertebrates. However, it also appears that deuterostomes underwent several cadherin-related innovations. Based on their similar spatiotemporal expression patterns and orthologous relationships to PCP-related and tumor-suppressing proteins, we hypothesize that sea urchin cadherins may play a role in regulating the shape and growth of embryonic epithelia and organs. Future experiments will examine cadherin expression in non-echinoid echinoderms and explore the functions of cadherins during echinoderm development.

**Supplementary Information:**

The online version contains supplementary material available at 10.1186/s13227-023-00219-7.

## Background

**Fig. 1:**
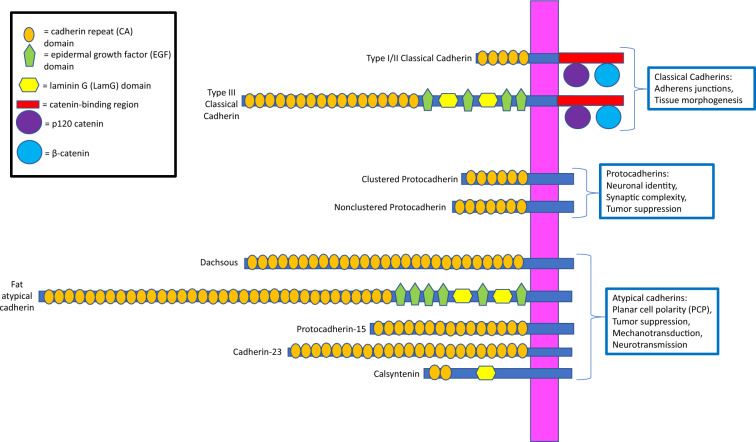
Main structures and functions of three cadherin subfamilies in deuterostomes. Domain architectures for representative classical cadherins, protocadherins, and atypical cadherins are derived from the SMART database. The domain structures for all the cadherins depicted are derived from *Mus musculus* with the exception of the type III classical cadherin, which instead originates from *Lytechinus variegatus*. The magenta bar encompasses the transmembrane regions of each protein, and the functions of each of the three subfamilies are outlined in blue. More in-depth descriptions of research on these functions are available in the Background section

The cadherin superfamily is a diverse group of calcium-dependent cell–cell adhesion proteins that are essential for metazoan growth and development. These proteins contain a minimum of two extracellular cadherin-specific repeats, a transmembrane domain, and a relatively short cytoplasmic tail [1–4]. Cadherin superfamily members are generally grouped into three subfamilies: the classical cadherins, atypical cadherins, and protocadherins (Fig. 1) [1]. Classical cadherins contain intracellular regions with a juxtamembrane domain, which binds p120 catenin, and a beta-catenin binding domain [5]. While the extracellular regions of vertebrate-specific classical cadherins, which include type I and type II cadherins, consist of five cadherin repeats, type III classical cadherins, which are found in protostomes and deuterostomes, contain ectodomains with epidermal growth factor-like (EGF-like) domains, laminin globular-like (LamG) domains, and more than five cadherin repeats (Fig. 1) [5–8]. Atypical cadherins possess wide-ranging numbers of extracellular cadherin repeats and highly variable cytoplasmic tails that lack catenin-binding motifs (Fig. 1) [1]. They are ancestral proteins that predate Bilateria [9, 10] and were previously referred to as protocadherins [11]. However, recent phylogenetic research has redefined protocadherins as a subfamily of non-catenin-binding cadherins with 6–7 extracellular cadherin repeats (Fig. 1) [12, 13]. In vertebrates, protocadherin-encoding genes are arranged tandemly in clusters or scattered within the genome [12, 13].

Classical cadherins are critically important for proper bilaterian development. The extracellular regions of classical cadherins interact with those of neighboring cadherins while the cytoplasmic regions recruit p120 catenin, beta-catenin and other actin-binding proteins to form stable homophilic cell–cell adhesion complexes [1, 14]. These complexes are generally referred to as adherens junctions and consist of molecular bridges that connect the plasma membranes and contractile actomyosin networks of neighboring cells [15]. Adherens junctions facilitate mechanosensitive intercellular signaling, and this cell coupling is essential for mediating biological processes that direct tissue patterning and morphogenesis [14, 16–19]. Some examples of these cadherin-mediated processes include the formation of a functional digestive epithelium by E-cadherin, a type I cadherin, in mouse [16], the establishment of *Drosophila* neural synaptic connections by N-cadherin, a type III cadherin [17, 18], and sea urchin germ line cell specification by G-cadherin (GCDH), another type III cadherin [19]. Classical cadherins also appear to be crucial for the proper development of the three primary germ layers during sea urchin gastrulation [20–22].

Various atypical cadherins also mediate development by participating in signal transduction pathways. Two well-studied, atypical cadherins are the Fat receptor proteins and their corresponding ligands, the Dachsous (DCHS) proteins, which regulate bilaterian tissue growth and planar cell polarity (PCP) (Fig. 1) [23]. In *Drosophila*, binding of DCHS to Fat enables the receptor’s intracellular phosphorylation by Discs Overgrown [24], which subsequently exerts a tumor suppressor effect via the Hippo pathway that prevents imaginal disc overgrowth [25, 26]. Fat proteins are also necessary for inhibiting mammalian cancer metastatis [27, 28], although the specific roles of the four Fat and two DCHS vertebrate orthologs in the Hippo pathway are currently unclear [23]. Fat and DCHS proteins also modulate PCP, or the organization of cells within epithelial layers [9]. In deuterostomes and protostomes, these proteins serve as directional cues that establish embryonic axes [23, 29–35]. In Drosophila, Fat and DCHS mediate spatiotemporal patterning of the wing [29], eye [30], and hindgut epithelia [31]. Fat4 and DCHS1 are crucial for various PCP-dependent processes in mice, such as hindbrain neuronal migration [32], sternum ossification [33], kidney growth [34], and cochlear elongation [35]. Another atypical cadherin complex consists of cadherin-23 (CDH23) and protocadherin-15 (PCDH15) (Fig. 1), which is located in vertebrate inner ears [36]. The CDH23-PCDH15 complex is essential for activating the mechanotransduction pathway that mediates hearing and balance [37, 38].

Several cadherin proteins are also essential for directing neural development. Protocadherins are suspected to play key roles in mediating neuronal connectivity and synaptic complexity [1, 12, 39]. Clustered protocadherins, which have six cadherin repeats (Fig. 1), only been identified in vertebrates [12, 13], play essential roles in establishing neuronal identity and promoting dendrite complexity within the central nervous system [13, 40, 41]. Non-clustered protocadherins, which have seven cadherin repeats (Fig. 1**),** also regulate neuronal connectivity [39], with proteins such as protocadherin-9 (PCDH9) being implicated in memory and emotional behavior [42–44]. These proteins also act as tumor suppressors that regulate neuron proliferation during vertebrate embryogenesis [40, 45]. Calsyntenins (CSTNs) are atypical cadherins that are essential for regulating the secretion of the inhibitory neurotransmitter, GABA, within protostome and deuterostome synapses (Fig. 1) [46–48]. Loss-of-function mutations in CSTN1 have been shown to reduce GABAergic neurotransmission within mice interneuron populations [46, 47] and at neuromuscular junctions in *C. elegans* [48]. Because of their impacts on neuronal communication, mutations in genes encoding protocadherins and CSTNs are associated with multiple human neurodevelopmental disorders [44, 47].

While cadherins have been well-studied in vertebrates and arthropods, they are currently poorly characterized in echinoderms. To date, G-cadherin (GCDH), a classical cadherin broadly expressed in epithelial tissues during early sea urchin development, is the only echinoderm cadherin that has been studied in detail [19, 20]. A previous phylogenetic analysis identified a complement of eight cadherins in *Strongylocentrotus purpuratus* that included mostly proteins that shared between protostomes and deuterostomes (i.e., CSTN, DCHS, Fat1, Fat4, GCDH) and lacked vertebrate-specific classical cadherins and clustered protocadherins [49]. This complement contained at least two proteins that were previously believed to be chordate-specific [49]. However, this analysis was based on an outdated genome assembly and only included one echinoderm species [49]. Since echinoderms are basal deuterostomes and closer relatives to chordates than most invertebrates [49], broader analyses of echinoderm cadherins will provide crucial insights concerning the evolutionary history of this important superfamily of proteins within deuterostomes.

The goal of this paper was to build upon our understanding of echinoderm cadherins and their patterns of expression during embryonic development. We first investigated the cadherin complement by phylogenetically comparing protein sequences from diverse echinoderm species to sequences from protostome, non-chordate deuterostome, and chordate species. This phylogenetic analysis allowed us to identify orthologs that are shared between echinoderm, protostomes, and deuterostomes. We next examined the spatiotemporal expression patterns of the suite of cadherin superfamily genes that are expressed during sea urchin embryogenesis. We aimed to compare our findings on echinoderm cadherin genes and their expression patterns to information available for other bilaterians.

## Results

### Phylogenetic analysis of cadherin proteins

**Fig. 2:**
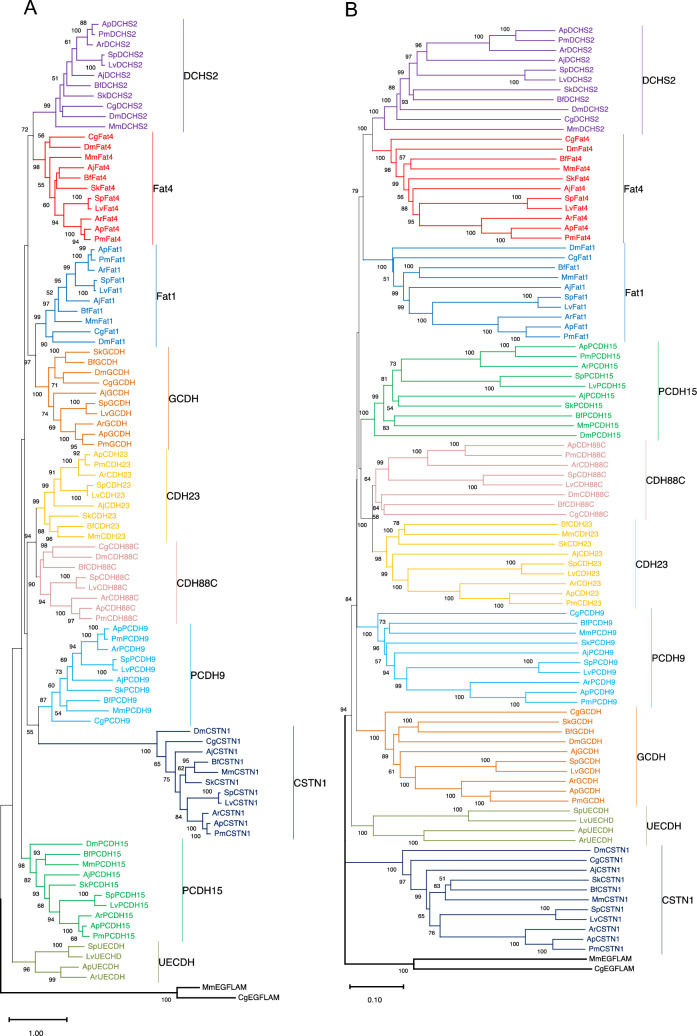
Phylogenetic analysis of amino acid sequences for echinoderm cadherins utilizing maximum likelihood and neighbor-joining methods. Both analyses were conducted on various cadherin sequences from echinoderm, non-echinoderm deuterostome, and protostome species using MEGA11 software. Clades and their respective orthologous sequences are labeled and color-coded. Two EGFLAM sequences from *Mus musculus* and *Crassostrea gigas* are included as an outgroup for both analyses. Full species and protein names for each taxon and their respective GenBank accession number(s) and amino acid sequences are listed in Additional file 1. **A**. A maximum likelihood analysis was performed using the WAG + F + G substitution model with 500 bootstrap replicates utilizing amino acid sites with ≥ 95% coverage across all taxa. **B**. A neighbor-joining analysis was performed using the p-distance substitution model with 5000 bootstrap replicates with pairwise deletion of amino acid sites

Our maximum likelihood (ML) and neighbor-joining (NJ) analyses identified ten cadherin proteins in echinoderms (Fig. 2) using amino acid sequences from six echinoderm species (see Additional file 1: Table S1). Unlike the analysis by Whittaker et al. our phylogenetic analyses excluded cadherin EGF LAG seven-pass receptor (CELSR) protein sequences, since these proteins can also be categorized as adhesion-related G-protein coupled receptor proteins (GPCR) due to the presence of seven transmembrane domains and a GPCR proteolytic site [49]. Three proteins (CSTN1, DCHS2, Fat4) have unambiguous orthologs in all the bilaterian species we examined, while Fat1 and PCDH15 appear to be absent in the hemichordate, *S. kowalevskii*, and the protostome, *C. gigas*, respectively (Fig. 2). Orthologs of GCDH were identified in all the species analyzed other than the mouse, *M. musculus* (Fig. 2). PCDH9 was identified in all analyzed species other than the arthropod, *D. melanogaster* (Fig. 2). One protein, CDH23, is deuterostome-specific (Fig. 2). Cadherin-88C (CDH88C) orthologs were identified in all bilaterians with the exception of three deuterostome species, which included the crinoid, *A. japonica*, the hemichordate *S. kowalevskii*, and the mouse, *M. musculus* (Fig. 2). Our analysis also identified a previously uncharacterized, echinoderm-specific cadherin (UECDH) that was present in all species other than the asteroid, *P. miniata*, and the crinoid, *A. japonica* (Fig. 2).

While most orthologs recapitulate the expected phylogenetic relationships in the trees containing all the cadherin proteins, DCHS2, CSTN1, and Fat4 exhibit unexpected tree branching patterns. MmDCHS2 is at the base of its respective clade in both the ML and NJ trees (Fig. 2), which makes it appear more distantly related to the echinoderm orthologs than to the protostome proteins. Also, within the ML phylogeny, AjCSTN1 unexpectedly appears to be more distantly related to the echinoid and asteroid orthologs than to the other non-echinoderm deuterostome orthologs, while AjFat4 shares a clade with BfFat4 and SkFat4 (Fig. 2A). These conflicting results may have been the result of alignment errors that arose when attempting to analyze distantly related protein sequence, for the cadherin superfamily consists of several functionally diverse subfamilies with distinct domain architectures that could confound the analysis [50]. Thus, to minimize the impact these errors could have had on downstream phylogenetic analyses, each set of DCHS, CSTN1, and Fat4 protein sequences was aligned and phylogenetically analyzed in isolation from the other cadherin orthologs. When the DCHS2 orthologs are individually aligned and phylogenetically analyzed, the ML analysis places the protostome proteins within their own clade (Additional file 2: Fig. S1A), while the NJ analysis reproduces the branching pattern exhibited by the original phylogenies by once again placing the mammalian ortholog at the base of the DCHS2 clade (Additional file 2: Fig. S1B). In contrast, when the CSTN1 and Fat4 orthologs are individually aligned and phylogenetically analyzed using the ML or NJ method, both resulting trees recapitulate the expected phylogenetic relationships by placing AjCSTN1 and AjFat4 as the basal orthologs for echinoderm-specific clades (Additional file 2: Figs. S2, S3). These conflicting results may reflect limitations in the individual analysis methods as MmDCHS2, which is one of two DCHS proteins in mammals [23], may have vertebrate-specific features in its amino acid sequence that complicate the analysis.

Most echinoderm cadherins (CDH23, CDH88C, CSTN1, Fat1, PCDH15 and UECDH) are atypical cadherins, as they lack catenin-binding motifs and contain a number of cadherin repeats that differs from 6–7 (Additional file 3: Figs. S4–S9). While two consecutive cadherin repeats were not detected in SpCSTN1 and SkCSTN1, they are orthologous to echinoderm proteins (i.e., AjCSTN1, ApCSTN1) and non-echinoderm proteins (i.e., BfCSTN1, DmCSTN1, MmCSTN1) that did meet this threshold (Additional file 3: Fig. S6), so these orthologs are still classified as cadherins in the analysis. PCDH9 is the only protocadherin present within the set of echinoderm proteins, as it contains 7 cadherin repeats (Additional file 3: Fig. S10). GCDH is a classical cadherin, as it contains a catenin-binding motif in all the analyzed species (Additional file 3: Fig. S11). Within the classical cadherin family, GCDH falls within the type III subfamily, as its extracellular domain contains 14–17 tandem cadherin repeats that are adjacent to three EGF-like motifs that are alternated with two LamG domains [8]. Interestingly, DCHS2 and Fat4 each contain predicted catenin-binding motifs that are characteristic of classical cadherin cytoplasmic tails in only a subset of the analyzed deuterostome species. All deuterostome DCHS2 proteins with the exception of the *M. musculus* ortholog contain juxtamembrane, or p120 catenin-binding, and beta-catenin-binding motifs, while only the Fat4 orthologs in *A. japonica* and *S. kowalevskii* possess these motifs (Additional file 3: Figs. S12–13). The presence of these catenin-binding domains in only a subset of the analyzed organisms makes it impossible to assign a cadherin subfamily classification to all of the DCHS2 and Fat4 orthologs. While the non-vertebrate deuterostome DCHS2 proteins, AjFat4, and SkFat4 appear to be classical cadherins, all other DCHS2 and Fat4 proteins appear to be atypical cadherins. On the other hand, several cadherin sub-families found in vertebrates, such as type I and type II classical cadherins, which have five extracellular cadherin repeats and an intracellular catenin-binding motif [2, 5, 7, 8], and clustered protocadherins, which have six extracellular cadherin repeats [2, 12, 13], are absent in echinoderms.

### Corrected annotation of cadherin gene models

During the course of our analysis, we determined that seven of the analyzed proteins (CDH23, CSTN, DCHS, Fat1, Fat4, PCDH15, UECDH) are encoded by incorrectly annotated gene models in at least one organism, based on several lines of evidence (Additional file 3: Figs. S14–15). For most mis-annotated orthologs (i.e., CSTN, DCHS2, Fat1, Fat4, CSTN, and UECDH), the proteins are encoded by inappropriately split, adjacent gene models that are oriented in the same direction on the same scaffold or chromosome (Additional file 3: Fig. S14). For most species’ mis-annotated orthologs, the N-terminal third of the protein and the C-terminal two-thirds are encoded by separate gene models, with only the downstream gene model encoding a transmembrane domain (Additional file 3: Fig. S14). Furthermore, only the upstream gene models for the DCHS, Fat1, and Fat4 proteins contain a signal peptide and a likely 5’-UTR, which contains multiple stop codons (Additional file 3: Fig. S14A, B, D, E). Another line of evidence is that the amino acid sequences determined to encode the N-terminal and C-terminal sections of these proteins align to adjacent regions of proteins encoded by single gene models in *Drosophila melanogaster* and *Mus* musculus, two species with high quality, extensively annotated genome assemblies. The only exception to this was BfDCHS2, which is encoded by loci on separate scaffolds that each align to separate sections of the complete gene models encoding DmDCHS2, asteroid DCHS2, and MmDCHS2. However, since the N-terminally aligned sequence does not have a 3ʹ-UTR beginning with a stop codon while the C-terminally aligned sequence does not have a 5ʹ-UTR, the separation of these gene models is still likely the result of a genome assembly error.

In contrast to the other cadherins, the current gene models for all echinoderm CDH23 orthologs other than LvCDH23 were determined to be incorrect fusions of loci encoding two separate proteins, only one of which is a cadherin. These determinations were made based on comparisons of these gene models to LvCDH23*,* with only the last two-thirds of the SpCDH23 amino acid sequence aligning to the LvCDH23 sequence (Additional file 3: Fig. S15A). LvCDH23 is encoded by an mRNA with a 5ʹ-UTR containing multiple stop codons, downstream of which is a sequence which encodes a predicted signal peptide (Additional file 3: Fig. S15B), suggesting that LvCDH23 is encoded by a complete gene model. Furthermore, SpCDH23 has a signal peptide near the beginning of the region that aligns to LvCDH23 (Additional file 3: Fig. S15C). Based on these considerations, we conclude that other echinoderm CDH23 proteins are also likely encoded by incorrectly fused gene models.

### Embryonic cadherin gene expression in a sea urchin

We used whole mount in situ hybridization (WMISH) to analyze the embryonic expression of cadherin family members in the euechinoid, *S. purpuratus*. In addition to G-cadherin, the only echinoderm cadherin that has been studied in detail [19, 20], four other cadherin family members are expressed at appreciable levels (maximum expression > 50 transcripts/million (TPM)) during embryogenesis: *Sp-fat1*, *Sp-fat4*, *Sp-pcdh9*, and *Sp-dchs2*. Zygotic expression of all four genes begins 10–20 h post-fertilization (hpf) (pre-hatching blastula stage) and levels of all four mRNAs peak ~ 30 hpf (early to mid-gastrula stage). Levels of mRNA expression decline modestly during later embryogenesis except in the case of *Sp-pcdh9*, which continues to be expressed at a relatively constant level [51].

**Fig. 3:**
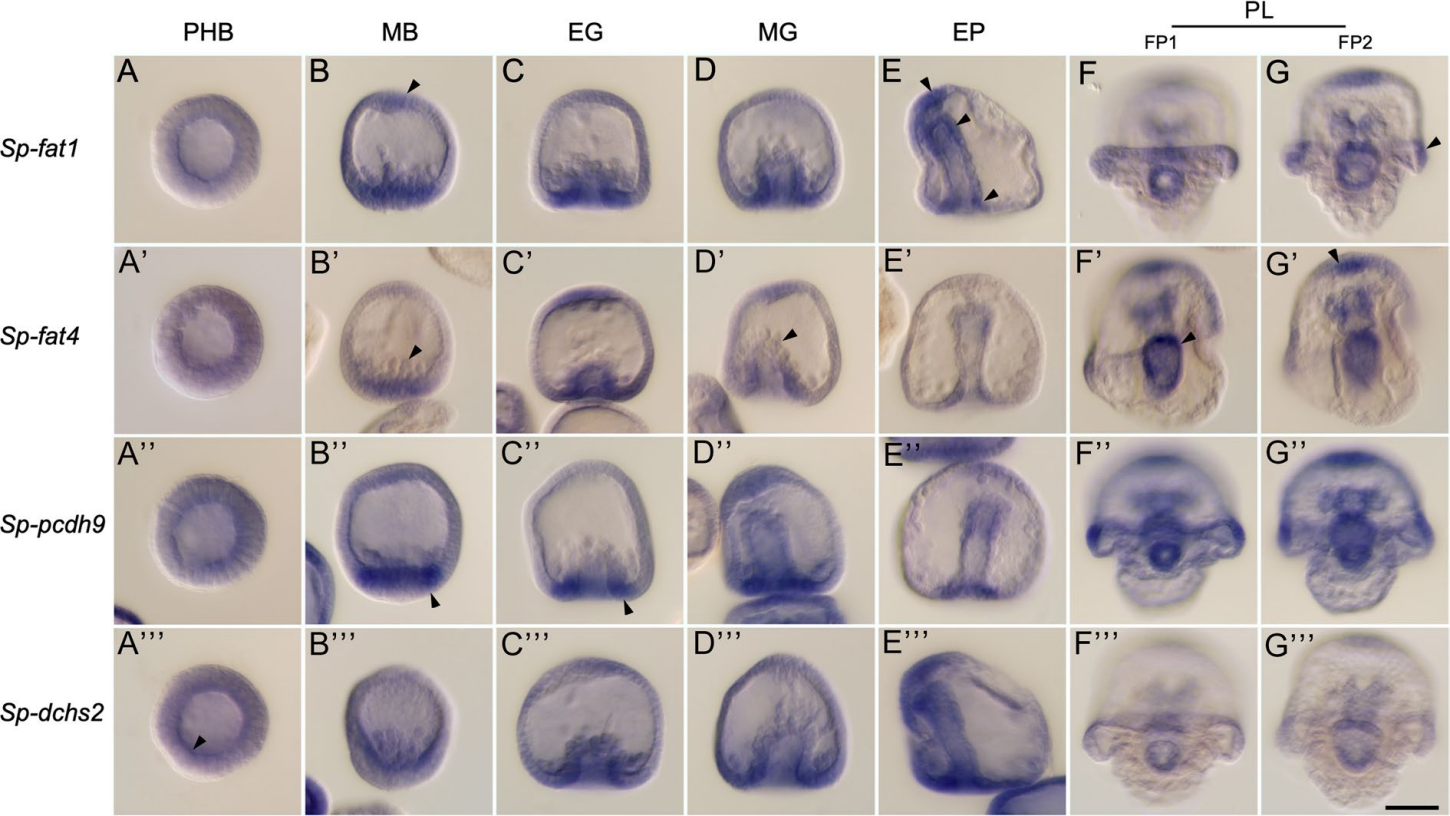
Developmental expression of *Sp-fat1*, *Sp-fat4*, *Sp-pcdh9*, and *Sp-dchs2* analyzed by whole mount in situ hybridization. All four genes show strikingly similar patterns of expression. Prior to the mesenchyme blastula stage, faint signal is seen in all cells and is highest in the basal cytoplasm (arrowhead, **A**’’’). By the late mesenchyme blastula (MB) stage, signal is enriched in the apical plate (arrowhead, **B**) and vegetal plate (arrowhead, **B**’’) but is absent from migrating primary mesenchyme cells (arrowhead, B’). At the early gastrula (EG) and mid-gastrula (MG) stages, intense signal is seen at the margin of the blastopore (arrowhead, C’’) and in the apical plate, but ingressing non-skeletogenic mesenchyme cells at the tip of the archenteron are unlabeled (arrowhead, D’). At the early prism (EP) stage, expression is detected primarily in the apical plate, the prospective foregut, and at the margin of the blastopore (arrowheads, **E**). At the pluteus (PL) stage, expression is elevated in the apical plate (arrowhead, G’), the portion of the ciliary band overlying the postoral arms (arrowhead, **G**), and in the gut (arrowhead, F’). FP- focal plane. PHB- pre-hatching blastula (16 hpf). MB- mesenchyme blastula (24 hpf). EG- early gastrula (28 hpf). MG- mid-gastrula (30–32 hpf). EP- early prism (44 hpf). PL- pluteus (72 hpf). Each pluteus was imaged in two different focal planes (FP1 and FP2). Scale bar = 50 μm

WMISH analysis revealed that all four cadherin family members exhibited strikingly similar spatial expression patterns during embryogenesis (Fig. 3; Additional file 4: Fig. S16). At the pre-hatching blastula stage (16 hpf), which sampled the earliest period of zygotic expression detectable by RNA-seq, we detected only low, uniform levels of staining, which could represent low levels of ubiquitous expression or background. At the mesenchyme blastula stage (24 hpf), however, WMISH signal was clearly elevated in cells of the vegetal plate. Primary mesenchyme cells (PMCs) that had migrated away from the vegetal plate were unlabeled. In some embryos, PMCs that were adjacent to the vegetal plate (and therefore presumably had just ingressed) were faintly stained, while PMCs that had moved from the vegetal plate were unlabeled. By the start of invagination (early gastrula stage, 28 hpf), WMISH signal was highly enriched at the margin of the blastopore. In many embryos, expression was also elevated at the animal pole in the region of the developing apical plate, although this was less consistent than expression at the blastopore margin (Additional file 4: Fig. S16). Non-skeletogenic mesoderm cells at the anterior tip of the archenteron were unlabeled. This pattern persisted at the mid-gastrula stage (30–32 hpf). At the late gastrula stage/early prism stage (40–44 hpf), signal continued to be elevated at the blastopore margin and in the apical plate, but expression was also now detectable in the anterior part of the archenteron, a region that gives rise to the coelomic pouches and foregut. At the early two-armed pluteus stage (72 hpf), the pattern of expression was complex, but staining was consistently apparent in the apical plate, ciliary band (particularly in the region overlying the postoral arms), and throughout the gut. In many specimens, signal in the gut was most intense at the sites of the cardiac and pyloric sphincters, which were forming at this stage.

To confirm these patterns of expression, for each gene we tested two different probes that were complementary to non-overlapping regions of the target mRNA (probe sequences are shown in Additional file 5). Although some probes gave somewhat stronger signal than others, in all cases, both probes showed identical patterns of expression. In addition, to rule out the possibility of off-target hybridization, we confirmed by BlastN against the S. purpuratus genome (v.5) that each probe was complementary only to the intended target mRNA, with no appreciable similarity to any other genomic region. Lastly, using the same samples of fixed embryos, we also tested a digoxigenin-labeled probe complementary to the alx1 mRNA, which was shown previously to be expressed specifically by PMCs [52], and observed the expected staining pattern, which was distinct from that of the cadherin probes.

**Fig. 4:**
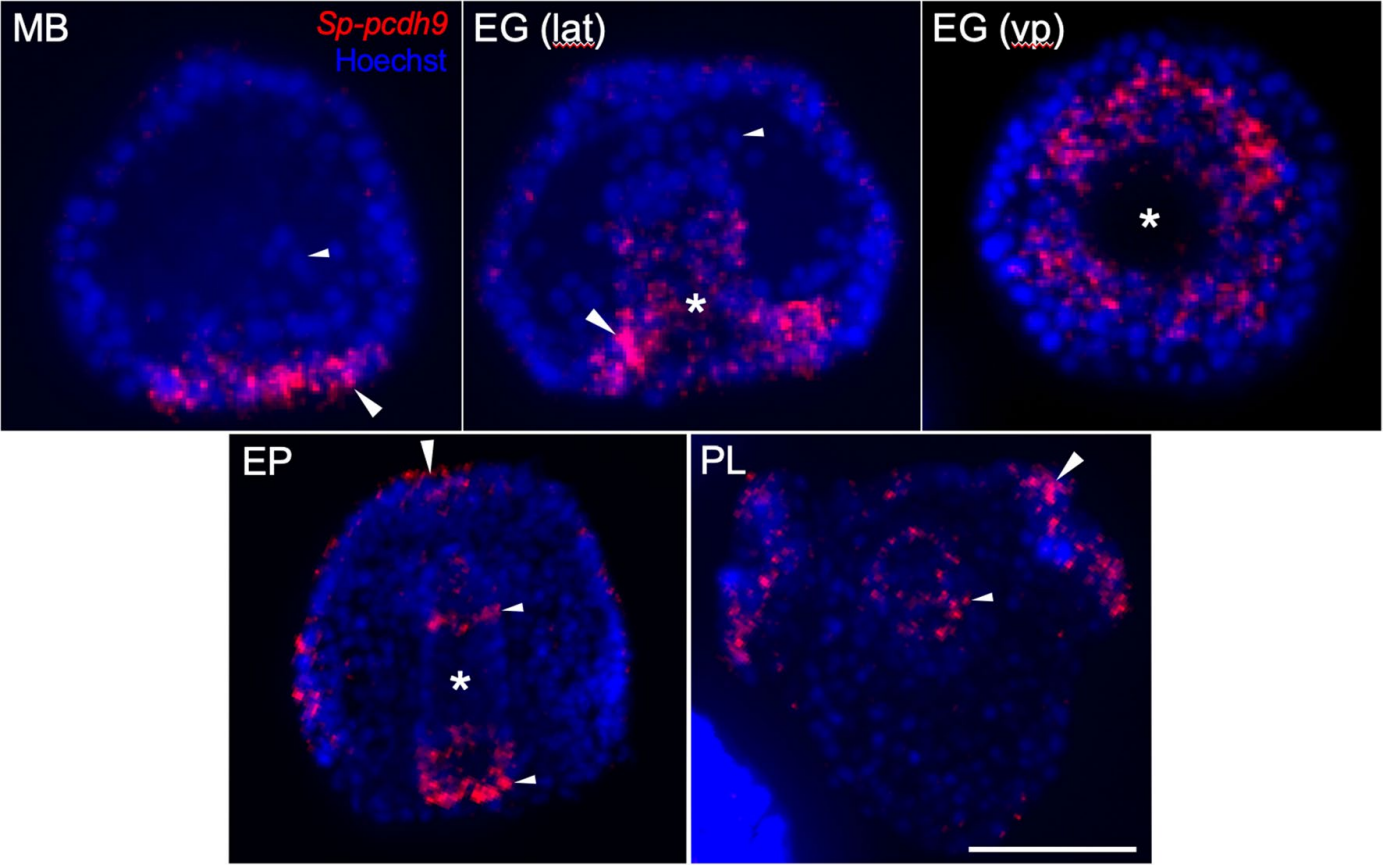
Fluorescent whole mount in situ hybridization analysis of a representative cadherin gene (*Sp-pcdh9).* Each image shows a z-projection of confocal slices (15–73 slices/stack). Red- *Sp-pcdh9 mRNA.* Blue- Hoechst staining (nuclei). At the late mesenchyme blastula (MB) stage, strong signal is seen in the vegetal plate (large arrowhead) but is absent from migrating primary mesenchyme cells (small arrowhead). At the early gastrula (EG) stage, signal is elevated at the margin of the blastopore (large arrowheads) but is absent from migrating secondary mesenchyme cells (small arrowhead). At the early prism (EP) stage, expression is highest at what appears to be the foregut/mid-gut boundary and in the circumblastoporal region (small arrowheads), as well as in the apical plate (large arrowhead). At the pluteus (PL) stage, expression is elevated in the ciliary band, especially overlying the postoral arms (large arrowhead), and in the gut (arrowhead). Asterisks indicate the archenteron. MB- mesenchyme blastula (24 hpf). EG- early gastrula (28 hpf). EP- early prism (44 hpf). PL- pluteus (72 hpf). Early gastrula stage embryos are shown in lateral (lat) and vegetal pole (vp) views. Scale bar = 50 μm

We also examined the pattern of expression of one representative cadherin (*Sp-pcdh9*) at higher resolution using fluorescence-based, whole mount in situ hybridization (F-WMISH) and confocal microscopy (Fig. 4). This analysis confirmed the dynamic expression of *Sp-pcdh9. Sp-pcdh9 mRNA* was initially enriched throughout the vegetal plate but became highly enriched at the blastopore margins during gastrulation. At late gastrula and post-gastrula stages, expression at the blastopore margin gradually resolved to the posterior hindgut (future anus). Expression also appeared in the foregut during gastrulation and appeared to resolve primarily to the foregut-midgut boundary, the site of the future cardiac constriction. Confocal analysis also confirmed expression of *Sp-pcdh9* in the ciliary band, with pronounced signal overlying the postoral arms (Fig. 4, PL).

## Discussion

### Evolution of echinoderm cadherins

Our phylogenetic analysis of six echinoderm species representing three families identified a total of ten cadherins, which was a slightly greater number of proteins than the analysis conducted by Whittaker et al. [49]. These ten cadherins include seven proteins that are identical cadherin superfamily members to those identified in this prior analysis. However, in contrast to that study, our analysis also excluded CELSR proteins, which have a structure that suggests they are distinct from other cadherin superfamily members [2, 49]. Using new and improved genome assemblies for *Strongylocentrotus purpuratus* and other echinoderms, we identified three additional cadherin proteins: PCDH15, UECDH, and CDH88C. PCDH15 is evolutionarily conserved among bilaterians but appears to have been lost in *C. gigas*. UECDH is a newly identified echinoderm-specific protein and likely originated in a common ancestor to echinoids and asteroids, with a later loss in *P. miniata*. All species except *M. musculus* contain orthologs to CDH88C, so it is possible that this protein is an evolutionarily conserved bilaterian cadherin that was selectively lost in vertebrates. Like the analysis by Whittaker et al. [49], our phylogeny shows that the echinoderm cadherin repertoire is more similar to that of protostomes and other deuterostome invertebrates than to extant vertebrate cadherins.

**Fig. 5:**
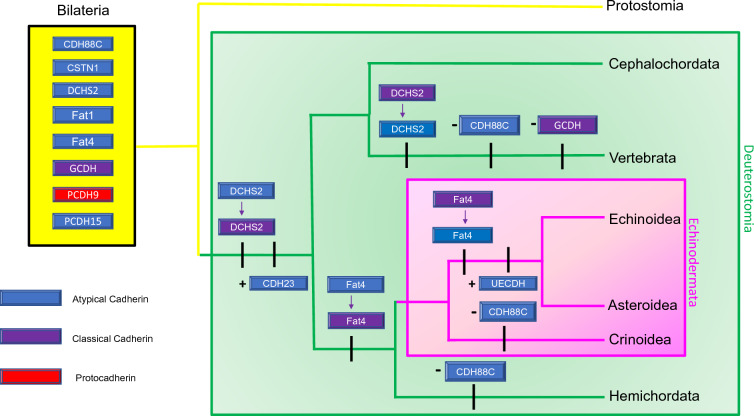
Diagram depicting the proposed evolutionary history of cadherin subfamily members in echinoderms. All cadherin subfamily members, which contain cadherin repeat (CA) domains, are depicted using blue, purple, or red rectangles. Classical cadherins are defined as cadherins that contain predicted intracellular p120 and beta-catenin-binding motifs. Both atypical cadherins and protocadherins lack these motifs, but protocadherins are defined as proteins that contain 6–7 CA domains. The ancient bilaterian cadherin toolkit suggested by our phylogenetic analysis is depicted using a yellow box. Additions, losses, or modifications of the proteins within this toolkit are depicted using black tick marks on the tree branches. Protein additions are represented using plus signs ( +), while losses are represented using minus signs (−). The conversion of a protein from one cadherin subfamily to a different classification is depicted using an arrow. If branch-specific loss is not noted for a taxon on the diagram, it is assumed that organism contains all the proteins within the ancient bilaterian cadherin toolkit. CDH23 = cadherin-23, CDH88C = cadherin-88C, CSTN1 = calsyntenin-1, DCHS2 = dachsous-2, Fat1 = fat atypical cadherin 1, Fat4 = fat atypical cadherin 4, GCDH = G-cadherin, PCDH9 = protocadherin-9, PCDH15 = protocadherin-15, UECDH = uncharacterized echinoderm cadherin

Most echinoderm cadherins likely originated in a bilaterian ancestor that predated the protostome-deuterostome split. Our phylogenetic analysis shows that five atypical cadherins (i.e., CSTN1, DCHS2, Fat1, Fat4, and PCDH15) in echinoderms are orthologous to proteins in at least one protostome species. PCDH15 is present in the arthropod, *D. melanogaster*, but not the mollusc, *C. gigas*, suggesting that it was selectively lost in at least one protostome lineage. The presence of these orthologs is consistent with previous evolutionary studies, which suggest that atypical cadherins arose before the protostome-deuterostome divergence [8, 9]. The bilaterian ancestor also likely had at least one nonclustered protocadherin (Fig. 5). PCDH9 orthologs are found in all the deuterostomes and in *C. gigas,* with an apparent loss in *D. melanogaster*. This result for PCDH9 differs from that of the analysis by Whittaker et al. [49], which misidentified this ortholog as being deuterostome-specific likely due to it only including protostome species that selectively lost this protein. This bilaterian ancestor also likely possessed a classical type III cadherin that was orthologous to echinoderm GCDH (Fig. 5). Both our analysis and previous studies have identified type III cadherin orthologs in multiple protostome species, with an apparent loss in mammals [2, 5, 8, 49]. This GCDH ortholog may have served a generalized function in forming adherens junctions, a common structure among all bilaterians [5, 7]. However, the suite of echinoderm cadherins does not include clustered protocadherins or type I or II cadherins, which are both crucial features of vertebrate cadherin toolkits [2, 5, 11]. The echinoderm cadherin toolkit only includes a single protocadherin encoded by an isolated gene, and all proteins with intracellular catenin-binding motifs have extracellular regions containing more than five cadherin repeats and additional non-cadherin repeat motifs.

While the echinoderm cadherin toolkit shares most of its orthologs with protostomes, we also identified deuterostome-specific innovations. The only vertebrate cadherin that appears to have originated in a common ancestor with echinoderms is CDH23, as orthologs to this protein are found in all the examined deuterostome species but not in protostomes (Fig. 5). In mammals, CDH23 is necessary for maintaining cochlear hair cell function and hearing sensitivity [36, 37]. CDH23 is not expressed at an appreciable level during the first 70 h of *S. purpuratus* development [51], but it is possible that this protein plays a role in the larval or adult stages of the echinoderm life cycle. Interestingly, DCHS2 and Fat4 proteins appear to have acquired predicted p120 and beta-catenin-binding motifs after the protostome-deuterostome split (Fig. 5), with these motifs exhibiting unusual phylogenetic distributions. Based on these distributions, DCHS2 with catenin-binding motifs appears to have originated in a basal deuterostome before being selectively lost in vertebrates, while Fat may have acquired catenin-binding motifs in an Ambulacarian ancestor before these were lost in an echinoid-asteroid common ancestor (Fig. 5). These new motifs may endow these PCP proteins with the ability to mediate cytoskeletal organization and function in a fashion similar to type I, II, and III classical cadherins [14, 15]. The selective acquisitions and losses of these catenin-binding domains results in the status of DCHS2 and Fat4 as classical cadherins or atypical cadherins being dependent on the organism (Fig. 5). However, considering these unusual phylogenetic distributions and the inclusion of only two chromosome-level protostome genomes, the analysis presented in the paper does not encompass enough metazoan diversity to conclude with confidence that these catenin-binding motifs are absent in protostomes.

### Shared spatiotemporal expression patterns of echinoderm cadherin genes

Our investigation of the spatiotemporal expression patterns of the four previously unstudied, embryonically-expressed cadherin genes (i.e., those encoding DCHS2, Fat1, Fat4, and PCDH9) revealed that they exhibited strikingly similar patterns of expression in epithelial cells during sea urchin gastrulation and organogenesis. The matching expression patterns of the Fat and DCHS atypical cadherins are consistent with previous studies demonstrating that they function as receptor-ligand pairs in the PCP and Hippo pathways [23, 53]. Unlike GCDH, which is expressed ubiquitously in embryonic epithelia of sea urchins [20], *sp-dchs2*, *sp-fat1*, *sp-fat4*, and *sp-pcdh9* expression is elevated in cells within the blastopore margin, archenteron, and apical plate before resolving to the foregut, hindgut, and ciliary band. An important question is whether these four cadherins serve redundant or synergistic functions during embryogenesis. In vertebrates, Fat1 and Fat4 are paralogs that both serve as planar cell polarity (PCP) signaling receptors, and whether they can accommodate each other’s functions depends on the tissue where they are expressed [54]. The *Drosophila* Fat1 and Fat4 orthologs have been demonstrated to play distinct roles, with Fat4 being indispensable for proximal–distal wing patterning [55] and Fat1 being necessary for egg chamber rotation and elongation in the developing ovary [56]. Since echinoderms are deuterostomes that have not undergone vertebrate-specific gene duplications [2], the precise functions and degree of redundancy of Fat1 and Fat4 in these organisms are currently unclear.

The spatiotemporal expression patterns of Fat1, Fat4, and DCHS2 suggest that these proteins may participate in echinoderm gastrulation and archenteron morphogenesis. The shift in localization of transcripts encoding these cadherins from the blastopore lip to the anterior archenteron during gastrulation mirrors that of Frizzled5/8 (Fz5/8) [57]. Fz5/8 is a non-canonical Wnt PCP pathway receptor that is necessary for primary invagination and archenteron formation [57–60], possibly through its activation of RhoA [58] or Dishevelled [59] and downstream effects on Jun-N-terminal kinase activity [60]. While studies suggest that the molecular relationship between the Fat-DCHS and non-canonical Wnt pathways depends on the organism and tissue [30, 61], Fat and DCHS orthologs have been demonstrated to be necessary for cytoskeleton-mediated epithelial tissue remodeling [9, 30, 34, 61–63]. Some examples of this remodeling include endomesoderm convergent-extension movements during zebrafish gastrulation [62] and apical constriction during mammalian neurulation [63], which involve mechanical processes that also occur during sea urchin gastrulation [20–22, 58]. Echinoderm Fat1, Fat4, and DCHS2 may also contribute to development of the archenteron into the larval tripartite gut because their expression patterns resolve to boundaries demarcated by the cardiac and pyloric sphincters after gastrulation [64]. Due to their roles in the PCP and Hippo pathways, orthologs of these atypical cadherins are essential for orienting and regulating cell divisions that shape tubular organs, such as the mouse kidney [34] and *Drosophila* hindgut [31]. The Fat-DCHS PCP pathway also appears to regulate the axial alignments of ectodermal and endodermal cells in a non-bilaterian metazoan [9], which suggests that it has a highly evolutionary conserved role in epithelial tissue organization that likely affects echinoderm organogenesis.

Fat1, Fat4, DCHS2, and PCDH9 may also be involved in the neurogenesis of the apical organ and ciliary band. Vertebrate Fat1, Fat4, and DCHS2 orthologs serve essential functions in regulating neuron migration, proliferation and shape [63, 65–67], such as inducing neurite outgrowth by antagonizing Hippo effectors [65, 66], enabling coordinated cell migration of different neuronal subtypes [67], and forming heterodimeric complexes that regulate neuroepithelial remodeling [63]. Based on previous vertebrate studies, PCDH9 may function as a tumor suppressor in echinoderm neuroectodermal tissues [40, 45, 68, 69]. This nonclustered protocadherin is necessary for regulating cell division in neuroepithelial progenitors during embryogenesis [40, 45] and preventing malignant central nervous system cancers [68, 69]. PCDH9 is also involved in mediating synaptic transmissions within sensorimotor systems [70], and unlike *Sp-fat1*, *Sp-fat4*, and *Sp-dchs2*, *Sp-pcdh9* continues to maintain high expression levels during sea urchin larval development [51]. Since *Sp-pcdh9* is expressed in the apical plate and ciliary band, which are neurogenic territories with chemically complex neuropeptide expression patterns [71], it may affect swimming and feeding behavior.

### Limitations and future studies

There are some limitations to our phylogenetic analysis of echinoderm cadherins. One is the incomplete nature of many of the genome assemblies we used, which may have affected our interpretations. The *A. planci* and *A. japonica* genome assemblies contain no scaffolds that exceeded 11.8 megabases in length [72], and only *D. melanogaster*, and *M. musculus* assemblies have correct models for all the cadherin-encoding genes examined. The genome assemblies of crinoids and hemichordates, which occupy crucial phylogenetic positions as representatives of the basal echinoderm family [73] and the sister phylum to echinoderms [74], respectively, are of relatively poor quality. The hemichordate and crinoid assemblies we used contain contigs with N50 values below 20 kilobases and L50 values above 5000 [73, 74], with the hemichordate genome being less than 90% complete [74]. The incomplete nature of these genomes may account for the apparent lineage-specific loss of Fat1 in the hemichordate and an absence of CDH88C in the hemichordate and the crinoid. They also limit the certainty of other conclusions, such as the lineage-specific retentions of catenin-binding Fat4 in only the hemichordate and crinoid and the restricted phylogenetic distribution of UECDH in echinoids and asteroids. To resolve these uncertainties, improved genome assemblies and gene annotations will be valuable.

Another limitation of this study is that cadherin expression patterns were only examined in an echinoid species. While many genes, including those encoding Fat1, Fat4, DCHS2, and PCDH9, are evolutionarily conserved among echinoderms [75], different echinoderm families have also diverged evolutionarily to the extent that they exhibit variations in their embryonic morphologies [76]. For example, asteroids have two ciliary bands while echinoids have only one, pointing to possible differences in cadherin expression (and perhaps function) in these two groups [76]. In contrast, the invagination of the vegetal plate and subsequent compartmentalization of the archenteron are highly conserved features of embryogenesis across the phylum [64, 75], suggesting that if cadherins play a role in these processes, those functions may be conserved as well.

A major goal of future work will be to explore the developmental functions of Fat1, Fat4, DCHS2, and PCDH9. Morpholinos and CRISPR-mediated gene editing could be used to perturb the expression of these proteins [77, 78], and dominant negative forms of Fat1 and Fat4 have been described [9, 57]. The similar spatiotemporal expression patterns of the four genes, however, points to possible functional redundancy which could complicate the interpretation of gene perturbation studies. Mis-expression studies may also be informative, as ectopic expression of a DCHS2 ortholog has been shown to alter PCP orientation within *Drosophila* tissues [31, 55]. Further studies will be necessary to test the hypothesis that cadherin family members, acting through the PCP pathway, play a conserved role in mediating gastrulation and archenteron morphogenesis in echinoderms.

## Conclusions

The echinoderm cadherin toolkit contains ten distinct proteins, and our analysis supports the view that this toolkit is more similar to that of a bilaterian common ancestor to protostomes and deuterostomes than to the cadherin repertoire of extant vertebrates. Some deuterostome-specific innovations were identified through the analysis of echinoderm cadherins, however, including (1) an atypical cadherin ortholog, and (2) catenin-binding motifs in proteins that were orthologous to major players in the planar cell polarity pathway. In situ hybridization studies in the echinoid, *Strongylocentrotus purpuratus*, showed that all four previously unstudied, embryonically-expressed cadherins exhibited strikingly similar spatiotemporal patterns of expression during embryogenesis. The expression patterns and orthologous relationships of the genes suggest that they may play roles in planar cell polarity within the echinoderm gut, ciliary band, apical organ, and their precursors. Based on the limitations of this study, future experiments should be aimed at examining cadherin expression in a broader range of echinoderms and perturbing the function of cadherins during embryonic development.

## Methods

### Protein selection

Amino acid sequences from *Strongylocentrotus purpuratus* were retrieved from Echinobase using ‘cadherin’ as the gene search term [72]. These sequences were then used in BLASTp searches to identify other cadherin-related proteins in *S. purpuratus*. The collection of protein sequences was examined using SMART [79], the NCBI Conserved Domain Database [80], and InterProScan [81] to check for the presence of cadherin repeats, transmembrane domains, and catenin-binding motifs. Signal peptides were detected using these three protein domain databases [79–81] and SignalP 6.0 [82]. The 5ʹ-UTRs of the mRNAs encoding the protein sequences were screened for the presence of stop codons and interrupted open reading frames using the ExPASy translate tool [83]. Only sequences that included at least one cadherin repeat and a single transmembrane domain were utilized as queries for species-specific BLASTp searches against other bilaterian species. Sequences that yielded reciprocal best hits with at least one protein with at least two consecutive cadherin repeats based on the highest bit score and met the homology thresholds of > 5% identity and > 60% query coverage were selected for phylogenetic analysis [84]. Only bilaterian amino acid sequences that yielded reciprocal best hits with echinoderm sequences were included in this selection, since this indicates that they are possibly orthologous proteins. All protein sequences and their corresponding species, Echinobase gene symbols, and NCBI Accession numbers are listed in Additional file 1.

### Phylogenetic analysis and ortholog identification

Amino acid sequences with possible echinoderm orthologs were aligned using the TM-Coffee MSA server [85]. Maximum-likelihood (ML) and neighbor-joining (NJ) phylogenetic analyses were conducted on the resulting alignment using MEGA11 software [86]. An NJ tree was generated using the p-distance substitution model assuming gamma distributed rates with 5000 bootstrap replicates. The best-fit function was utilized to determine that a WAG + F + G substitution model was the optimal method for generating a ML tree. A tree was then generated using 500 bootstrap replicates and a > 95% coverage threshold.

### Animals

Adult *S. purpuratus* were obtained from Marinus Scientific, LLC (Long Beach, CA, USA). Spawning of gametes and culturing of embryos were performed as previously described by Khor and Ettensohn [87, 88].

### In situ hybridization

Colorimetric in situ hybridization was carried out as described by Khor and Ettensohn [87] and fluorescence-based in situ hybridization was carried out as described by Khor and Ettensohn [88]. To ensure that mRNA detection was possible, we only examined four cadherin-encoding genes known to be expressed at appreciable levels (maximum expression > 50 TPM) during *S. purpuratus* embryogenesis based on a study by Tu et al. [51]. G-cadherin was excluded from all in situ hybridizations because its expression patterns have previously been examined by Miller and McClay [20]. For each of these four genes, we carried out in situ hybridizations using two different probes that were complementary to non-overlapping regions of the target mRNA. Alignments of each probe sequence to the *S. purpuratus* genome using BlastN showed no significant similarity to any other genes than their intended targets. The complete sequences for all the probes utilized are provided in Additional file 5.

### Supplementary Information

**Additional file 1: Table S1.** Protein sequences, abbreviations, and NCBI Accession Numbers utilized for phylogenetic analysis on echinoderm cadherins. All amino acid sequences that were utilized in the analysis are listed with their corresponding abbreviations after Table S1.

**Additional file 2: Fig. S1. **Phylogenetic analysis of amino acid sequences for echinoderm DCHS2 utilizing maximum likelihood and neighbor-joining methods. Both analyses were conducted on echinoderm, non-echinoderm deuterostome, and protostome dachsous-2 (DCHS2) proteins using MEGA11 software. The clade representing the DCHS2 orthologs is colored purple. Two EGFLAM sequences from *Mus musculus *and *Crassostrea gigas* were included as an outgroup for both analyses. Full species and protein names for each taxon and their respective GenBank accession number(s) and their amino acid sequences are listed in **Additional file 1**. **A**. A maximum likelihood analysis was performed using the WAG+F+G substitution model with 500 bootstrap replicates utilizing amino acid sites with >90% coverage across all taxa. **B**. A neighbor-joining analysis was performed using the p-distance substitution model with 5000 bootstrap replicates with pairwise deletion of amino acid sites. **Fig. S2.** Phylogenetic analysis of amino acid sequences for echinoderm CSTN1 utilizing maximum likelihood and neighbor-joining methods. Both analyses were conducted on echinoderm, non-echinoderm deuterostome, and protostome calsyntenin-1 (CSTN1) proteins using MEGA11 software. The clade representing the CSTN1 orthologs is colored dark blue. Two EGFLAM sequences from *Mus musculus *and *Crassostrea gigas* were included as an outgroup for both analyses. Full species and protein names for each taxon and their respective GenBank accession number(s) and their amino acid sequences are listed in **Additional file 1**. **A**. A maximum likelihood analysis was performed using the WAG+F+G substitution model with 500 bootstrap replicates utilizing amino acid sites with >90% coverage across all taxa. **B**. A neighbor-joining analysis was performed using the p-distance substitution model with 5000 bootstrap replicates with pairwise deletion of amino acid sites. **Fig. S3.** Phylogenetic analysis of amino acid sequences for echinoderm Fat4 utilizing maximum likelihood and neighbor-joining methods. Both analyses were conducted on echinoderm, non-echinoderm deuterostome, and protostome Fat4 proteins using MEGA11 software. The clade representing the fat atypical cadherin 4 (Fat4) orthologs is colored red. Two EGFLAM sequences from *Mus musculus *and *Crassostrea gigas* were included as an outgroup for both analyses. Full species and protein names for each taxon and their respective GenBank accession number(s) and their amino acid sequences are listed in **Additional file 1**. **A**. A maximum likelihood analysis was performed using the WAG+F+G substitution model with 500 bootstrap replicates utilizing amino acid sites with >90% coverage across all taxa. **B**. A neighbor-joining analysis was performed using the p-distance substitution model with 5000 bootstrap replicates with pairwise deletion of amino acid sites.

**Additional file 3: Fig. S4.** Comparison of echinoderm cadherin-23 (CDH23) structure to other bilaterians. Protein domain structures for various echinoderm, non-echinoderm deuterostome, and protostome species were visualized using SMART. Transmembrane domains are shown as blue rectangles while predicted signal peptides are shown in red. Class names for each representative taxon are given in parentheses. **Fig. S5.** Comparison of echinoderm cadherin-88C (CDH88C) structure to other bilaterians. Protein domain structures for various echinoderm, non-echinoderm deuterostome, and protostome species were visualized using SMART. Transmembrane domains are shown as blue rectangles while predicted signal peptides are shown in red. Class names for each representative taxon are given in parentheses. *Drosophila melanogaster* was included as a representative protostome species. **Fig. S6.** Comparison of echinoderm calsyntenin-1 (CSTN1) structure to other bilaterians. Protein domain structures for various echinoderm, non-echinoderm deuterostome, and protostome species were visualized using SMART. Transmembrane domains are shown as blue rectangles while predicted signal peptides are shown in red. Class names for each representative taxon are given in parentheses. *Drosophila melanogaster* was included as a representative protostome species. **Fig. S7.** Comparison of echinoderm fat atypical cadherin 1 (Fat1) structure to other bilaterians. Protein domain structures for various echinoderm, non-echinoderm deuterostome, and protostome species were visualized using SMART. Transmembrane domains are shown as blue rectangles while predicted signal peptides are shown in red. Class names for each representative taxon are given in parentheses. *Drosophila melanogaster* was included as a representative protostome species. **Fig. S8.** Comparison of echinoderm protocadherin-15 (PCDH15) structure to other bilaterians. Protein domain structures for various echinoderm, non-echinoderm deuterostome, and protostome species were visualized using SMART. Transmembrane domains are shown as blue rectangles while predicted signal peptides are shown in red. Class names for each representative taxon are given in parentheses. *Drosophila melanogaster* was included as a representative protostome species. **Fig. S10.** Comparison of echinoderm protocadherin-9 (PCDH9) structures to other bilaterians. Protein domain structures for various echinoderm, non-echinoderm deuterostome, and protostome species were visualized using SMART. Transmembrane domains are shown as blue rectangles while predicted signal peptides are shown in red. Class names for each representative taxon are given in parentheses. *Crassostrea gigas *was included as a representative protostome species. **Fig. S11.** Comparison of echinoderm G-cadherin (GCDH) structures to other bilaterians. Protein domain structures for various echinoderm, non-echinoderm deuterostome, and protostome species were visualized using SMART. Transmembrane domains are shown as blue rectangles while predicted signal peptides are shown in red. Class names for each representative taxon are given in parentheses. *Drosophila melanogaster* was included as a representative protostome species. **Fig. S12.** Comparison of echinoderm dachsous-2 (DCHS2) structures to other bilaterians. Protein domain structures for various echinoderm, non-echinoderm deuterostome, and protostome species were visualized using SMART. Transmembrane domains are shown as blue rectangles while predicted signal peptides are shown in red. Class names for each representative taxon are given in parentheses. *Drosophila melanogaster* was included as a representative protostome species. **Fig. S13.** Comparison of echinoderm fat atypical cadherin 4 (Fat4) structures to other bilaterians. Protein domain structures for various echinoderm, non-echinoderm deuterostome, and protostome species were visualized using SMART. Transmembrane domains are shown as blue rectangles while predicted signal peptides are shown in red. Class names for each representative taxon are given in parentheses. *Drosophila melanogaster* was included as a representative protostome species. **Fig. S14.** Echinoderm cadherin misannotations determined using genome assembly, protein domain structures, and untranslated mRNA regions. Several lines of evidence were collected in order to determine that there were cadherin-encoding genes erroneously split into two loci for various echinoderms on Echinobase. For A-D, the top image depicts the adjacent loci, which are outlined in red boxes, encoding each protein within the version 5.0 primary genome assembly for *Strongylocentrotus purpuratus *using the NCBI Genome Data Viewer. The top image in E instead depicts loci within the version 3.0 primary genome assembly for *Lytechinus variegatus*. The arrows projecting from each red box point to the incomplete protein structures encoded by the loci visualized using SMART, with signal peptides being depicted as red lines and transmembrane domains being depicted as blue boxes. The purple boxes outline the stop codons, which are depicted as dashes, and short open reading frames, which are depicted in red, within the 5’ untranslated regions (5’-UTR) of the mRNAs encoded by each misannotated locus, which were determined using the ExPASy translate tool. Translated amino acids are underlined. **A**. Fat atypical cadherin 4 (*fat4*). **B**. Fat atypical cadherin 1 (*fat1*). **C**. Calsyntenin-1 (*cstn1*). **D**. Dachsous-2 (*dchs2*). **E**. Protocadherin 15 (*pcdh15*). Echinoderm cadherin-23 (CDH23) misannotation determined using protein alignments, protein domain structures, and untranslated mRNA region. **A**. Alignment of original CDH23 protein sequences from *S. purpuratus *(LOC584236) and *L. variegatus *(LOC121431890). **B**. The purple box depicts the 5’-UTR within the mRNA encoded by LOC121431890, which is the gene encoding CDH23*, *in the version 3.0 genome assembly for *Lytechinus variegatus*. Short open reading frames are shown in red text while dashes depict stop codons. C. This image compares protein structures for *S. purpuratus *(Sp) and* Lytechinus variegatus *(Lv). The top structure shows the merged gene misannotation for *S. purpuratus *while the bottom shows the correct annotation based on comparison to *Lytechinus variegatus*. The image adjacent to the SMART diagrams shows the presence of a signal peptide in the corrected *S. purpuratus *model detected by Signal 6.0P, which was not detected by SMART.

**Additional file 4: Fig. S16.** Quantification of whole mount in situ hybridization expression patterns. For each developmental stage and gene, the number of embryos that exhibited elevated expression in the indicated region(s) is shown. The probes that were utilized for each gene, which have their nucleotide sequences listed in Additional file 5, are as follows: *Sp-*dchs2—Probe 2, Sp*-fat1*—Probe 2, *Sp-fat4*—Probe 1, *Sp-pcdh9*—Probe 1.

**Additional file 5: **Sequences of probes used for in situ hybridization. Nucleotide sequences for DNA probes that were complementary to target mRNAs corresponding to each cadherin-encoding gene were designed using nonoverlapping regions within the *Strongylocentrotus purpuratus *version 5.0 genome. Target specificity of probe sequences to only their intended target mRNAs, which are numbered and bolded, was confirmed using BlastN.

## Data Availability

All protein sequences used in the analysis are included in the manuscript and its additional files. All primer sequences used to generate probes for in situ hybridizations are available upon request.
