# Supplementary material for: Genome-wide identification and spatiotemporal expression analysis of cadherin superfamily members in echinoderms
Source: EvoDevo. 2023 Dec 20;14:15. doi: 10.1186/s13227-023-00219-7 (PMC10734073; doi:10.1186/s13227-023-00219-7)
Supplement: Supplementary file 1 — Additional file 1: Table S1. Protein sequences, abbreviations, and NCBI Accession Numbers utilized for phylogenetic analysis on echinoderm cadherins. All amino acid sequences that were utilized in the analysis are listed with their corresponding abbreviations after Table S1. [file 13227_2023_219_MOESM1_ESM.docx]

**Table S1**

| **Species** | **Developmental  Clade** | **Phylum** | **Protein Name** | **Abbreviation  in Paper** | **NCBI  Accession #(s)** |
| --- | --- | --- | --- | --- | --- |
| *Acanthaster  planci* | Deuterostomia | Echinodermata | cadherin 23 | ApCDH23 | XP_022080540.1 |
|  |  |  | cadherin 88C | ApCDH88C | XP_022111724.1 |
|  |  |  | calsyntenin 1 | ApCSTN | XP_022090488.1 |
|  |  |  | Dachsous 2 | ApDCHS2 | XP_022092788 .1 |
|  |  |  | fat atypical cadherin 1 | ApFat1 | XP_022108021.1 |
|  |  |  | fat atypical cadherin 4 | ApFat4 | XP_022091608.1 |
|  |  |  | G-cadherin | ApGCDH | XP_022110110.1 |
|  |  |  | protocadherin 9 | ApPCDH9 | XP_022100282.1 |
|  |  |  | protocadherin 15 | ApPCDH15 | XP_022101285.1 |
|  |  |  | uncharacterized echinoderm cadherin | ApUECDH | XP_022080498.1 |
| *Anneissia  japonica* | Deuterostomia | Echinodermata | cadherin 23 | AjCDH23 | XP_033109345.1 |
|  |  |  | calsyntenin 1 | AjCSTN1 | XP_033097836.1 |
|  |  |  | Dachsous 2 | AjDCHS2 | XP_033119936.1,  XP_033119933.1 |
|  |  |  | fat atypical cadherin 1 | AjFat1 | XP_033097613.1 |
|  |  |  | fat atypical cadherin 4 | AjFat4 | XP_033127144.1 |
|  |  |  | G-cadherin | AjGCDH | XP_033125355.1 |
|  |  |  | protocadherin 9 | AjPCDH9 | XP_033106016.1 |
|  |  |  | protocadherin 15 | AjPCDH15 | XP_033122343.1 |
| *Asterias  rubens* | Deuterostomia | Echinodermata | cadherin 23 | ArCDH23 | XP_033643870.1 |
|  |  |  | cadherin 88C | ArCDH88C | XP_033644427.1 |
|  |  |  | calsyntenin 1 | ArCSTN1 | XP_033642460.1,  XP_033642585 |
|  |  |  | dachsous 2 | ArDCHS2 | XP_033646268.1 |
|  |  |  | fat atypical cadherin 1 | ArFat1 | XP_033631595.1 |
|  |  |  | fat atypical cadherin 4 | ArFat4 | XP_033646357.1,  XP_033646358.1 |
|  |  |  | G-cadherin | ArGCDH | XP_033626095.1 |
|  |  |  | protocadherin 9 | ArPCDH9 | XP_033632045.1 |
|  |  |  | protocadherin 15 | ArPCDH15 | XP_033643933.1 |
|  |  |  | uncharacterized echinoderm cadherin | ArUECDH | XP_033644294.1,  XP_033644432.1 |
| *Branchiostoma  floridae* | Deuterostomia | Chordata | cadherin 23 | BfCDH23 | XP_035670190.1 |
|  |  |  | cadherin 88C | BfCDH88C | XP_035669276.1 |
|  |  |  | calsyntenin 1 | BfCSTN1 | XP_035696600.1 |
|  |  |  | dachsous 2 | BfDCHS2 | XP_035681485.1  XP_035664708.1 |
|  |  |  | fat atypical cadherin 1 | BfFat1 | XP_035681279.1,  XP_035681278.1 |
|  |  |  | fat atypical cadherin 4 | BfFat4 | XP_035681475.1 |
|  |  |  | G-cadherin | BfGCDH | XP_035680238.1 |
|  |  |  | protocadherin 9 | BfPCDH9 | XP_035681789.1 |
|  |  |  | protocadherin 15 | BfPCDH15 | XP_035669887.1,  XP_035681278.1 |
| *Crassostrea  gigas* | Protostomia | Mollusca | cadherin 88C | CgCDH88C | XP_034313307.1 |
|  |  |  | calsyntenin 1 | CgCSTN1 | XP_011449172.2 |
|  |  |  | dachsous 2 | CgDCHS2 | XP_034300844.1,  XP_034300627.1 |
|  |  |  | fat atypical cadherin 1 | CgFat1 | XP_034303171.1 |
|  |  |  | fat atypical cadherin 4 | CgFat4 | XP_011445615.2 |
|  |  |  | G-cadherin | CgGCDH | XP_011416906.2 |
|  |  |  | EGF-like, fibronectin type III and laminin G domains | CgEGFLAM | XP_011413779.2 |
|  |  |  | Protocadherin 9 | CgPCDH9 | XP_011441889.2 |
| *Drosophila  melanogaster* | Protostomia | Arthropoda | cadherin 88C | DmCDH88C | NP_731930.3 |
|  |  |  | calsyntenin 1 | DmCSTN1 | NP_001284722.1 |
|  |  |  | dachsous 2 | DmDCHS2 | AAA79329.2 |
|  |  |  | fat atypical cadherin 1 | DmFat1 | NP_001027138.2 |
|  |  |  | fat atypical cadherin 4 | DmFat4 | NP_477497.1 |
|  |  |  | G-cadherin | DmGCDH | NP_724070 |
|  |  |  | protocadherin 15 | DmPCDH15 | AAX12106.1 |
| *Lytechinus  variegatus* | Deuterostomia | Echinodermata | cadherin 23 | LvCDH23 | XP_041485604.1 |
|  |  |  | cadherin 88C | LvCDH88C | XP_041485949.1 |
|  |  |  | calsyntenin 1 | LvCSTN1 | XP_041480330.1,  XP_041480110.1 |
|  |  |  | dachsous 2 | LvDCHS2 | XP_041461524.1,  XP_041483662.1 |
|  |  |  | fat atypical cadherin 1 | LvFat1 | XP_041474678.1,  XP_041474592.1 |
|  |  |  | fat atypical cadherin 4 | LvFat4 | XP_041461446.1,  XP_041461436.1 |
|  |  |  | G-cadherin | LvGCDH | AAC06341.1 |
|  |  |  | protocadherin 9 | LvPCDH9 | XP_041456326.1 |
|  |  |  | protocadherin 15 | LvPCDH15 | XP_041485951.1,  XP_041485392.1 |
|  |  |  | uncharacterized echinoderm cadherin | LvUECDH | XP_041485410.1 |
| *Mus  musculus* | Deuterostomia | Chordata | cadherin 23 | MmCDH23 | NP_075859.2 |
|  |  |  | calsyntenin 1 | MmCSTN1 | XP_006539157.1 |
|  |  |  | dachsous 2 | MmDCHS2 | NP_001344094.1 |
|  |  |  | fat atypical cadherin 1 | MmFat1 | NP_001074755.2 |
|  |  |  | fat atypical cadherin 4 | MmFat4 | NP_899044.3 |
|  |  |  | EGF-like, fibronectin type III and laminin G domains | MmEGFLAM | NP_001403416.1 |
|  |  |  | protocadherin 9 | MmPCDH9 | NP_001258729.1 |
|  |  |  | protocadherin 15 | MmPCDH15 | XP_006513219.1 |
| *Patiria  miniata* | Deuterostomia | Echinodermata | cadherin 23 | PmCDH23 | XP_038061187.1 |
|  |  |  | cadherin 88C | PmCDH88C | XP_038061405.1 |
|  |  |  | calsyntenin 1 | PmCSTN1 | XP_038075569.1 |
|  |  |  | dachsous 2 | PmDCHS2 | XP_038055209.1 |
|  |  |  | fat atypical cadherin 1 | PmFat1 | XP_038055591.1 |
|  |  |  | fat atypical cadherin 4 | PmFat4 | XP_038055902.1,  XP_038055904.1 |
|  |  |  | G-cadherin | PmGCDH | XP_038077180.1 |
|  |  |  | protocadherin 9 | PmPCDH9 | XP_038055182.1 |
|  |  |  | protocadherin 15 | PmPCDH15 | XP_038060162.1 |
| *Saccoglossus  kowalevskii* | Deuterostomia | Hemichordata | cadherin 23 | SkCDH23 | XP_006822896.1 |
|  |  |  | calsyntenin 1 | SkCSTN1 | XP_006818111.1 |
|  |  |  | dachsous 2 | SkDCHS2 | XP_006816966.1,  XP_006816965.1 |
|  |  |  | fat atypical cadherin 4 | SkFat4 | XP_006812461.1,  XP_006812462.1 |
|  |  |  | G-cadherin | SkGCDH | XP_006818278.1 |
|  |  |  | protocadherin 9 | SkPCDH9 | XP_002741390.1 |
|  |  |  | protocadherin 15 | SkPCDH15 | XP_006812420.1 |
|  |  |  |  |  |  |
|  |  |  |  |  |  |
| *Strongylocentrotus  purpuratus* | Deuterostomia |  | cadherin 23 | SpCDH23 | XP_030835682.1 |
|  |  | Echinodermata | cadherin 88C | SpCDH88C | XP_030835749.1 |
|  |  |  | calsyntenin 1 | SpCSTN1 | XP_030843301.1,  XP_030843302.1 |
|  |  |  | dachsous 2 | SpDCHS2 | XP_030836545.1,  XP_030837145.1 |
|  |  |  | fat atypical cadherin 1 | SpFat1 | XP_030836440.1,  XP_030836441.1 |
|  |  |  | fat atypical cadherin 4 | SpFat4 | XP_030836541.1,  XP_030836540.1 |
|  |  |  | G-cadherin | SpGCDH | XP_030831012.1 |
|  |  |  | protocadherin 9 | SpPCDH9 | XP_ XP_787157.2 |
|  |  |  | protocadherin 15 | SpPCDH15 | XP_030835702.1 |
|  |  |  | uncharacterized echinoderm cadherin | SpUECDH | XP_030835990.1 |

>SpCDH23

MFQCTSILQCISSLLVCDGTNHCNDGSDEGLVCQNPCEPNPCLNQGVCSGSIGSLTYTCVCTAGFSGQNCGTDRASNDAPQFIYTQDPTVKVTEGQSPSIVVFTLNATDPDGDPITYDLFDDAARSIFTINQFTGQLYALPGLDYETLGNQVIITVSVTDGINDPVPQTIYVRITDVNDIAPTFTNLPNITYVFENATFGSELYQVITTDEDTFYGGFVTYSISLVTPSTPGSAPNNYFSIGETDGIIQLATSLNYEEIQQYFVTVVARDYGTGDTSLSSTATLTVSVLNIQDTNPVFLNIPYDVTIPETLPVGSTVQTITATDPDSNDGNIITYNLVVSDSQGYFVIDQTTGIITVNSQMDRDNPVLPATYPLIVSAVETSSGGVSFAQVSFNVTVSPANDEPPMFDQNSYSASIPESASVNSQLPITITVNDGDLITDSTVTVSLQNGNSVPFRVSPTQVVGSGTVDILITQPLDFENNQQYTLQLQAVDGDGQDIAPLMVTVTDVNDNNPVFINRPIDNIYRGSINENDQTGTFIVQVSTTDADSGSNAAVTYSIISGNINNVFTINPTSGAITNTQILGPNTPQTYTLSVRATNPTPGVAPATGTSTSTVIITVNDVNDSPPRFSDDEFTVFINEAAQVGTTVTRITATDEDIPEGDRLTYIITAGNTGGHFQINPVSGDVTLLRQLDRELESQHNLTIRATDQGAPPQSATTTLKVIVLDFNDNAPQWSPQSYVDSILENVPLNTFVLQVVATDADQGTNADLSFTINNANNVPFVIDDQGNIRTSGPLNRETVPQYNFLVTVLDNGSPQQAGNPPATVTINILDVNDEPPVLDRNSYEFTVSEDVNFNTFVGQVTATDPDLSGTLTYTFVPADPDFSYISGTIRTNTQLDANQQQVYQMTVFASDGVQNSQSAPVTINVLDVNNQAPSFGLGTYVKSLPEDSPFQTLVVDLNATDPDNGNTGLIIYSFESLSRTQGPFTVDPYTGVVEISTTAGLDRETQDEYTMIVIAVDQPASGQPMTGTATVSVIVTDVNDNAPTFRDISYEESIFENHPLGSNILTVQADDPDLGVNAVVLYRIISGNNNNLFNVDGQGNINLGSTPLDREMQDQYFLIVQAYNSDLSVGTNEVPVIINILDINDVTPLFQQQNYYRPDLSENSPSGTPVLQVFAQDQDLDLAGDVQYSLIGNPNFLTINSDTGVISVNGPIADLSGSRNFTMTVRATDQASPFLRGVATVTVTVVDDTVTIPIFTLSRYPATVIENVNADTPVTRVSATLNGNPAGITYAIDPNASPAVLEIFKIDENTGHITTEGAIDREVEDFYAFTVFAYHEDSVAPGSTSVWVNILDENDNPPEFITVPPSPVSVTENTGGNVPVGLFRATDPDTGINAQMTFSISGAGSNFFNVFQTANGGGTIVVTSPLDRERQDFYQLTVTATDGGNPSLSSSFPLNITVTDVNDNYPIWTRNVYEGSINENNFTSNPIITVMVNDLDLPSSNNFEYSIIAGDPMGMFEINSNGEITVSSLLDREQQETYQLTIELIDQTNSPPARTTTLAIITVGDENDNTPIFAETVIRVDVTEGPSSQNIPVYMLTANDIDLGLNRDIIYAIGSQQPQSHFQINPETGMISTTTALDWETISEYTLIVTATDQSPTEPRTGTATVIVSVNDINDGAPMFVRDVYGPYSVVEEVSNSYIDSFQATDTDQGLAGTVTYSVIGQYSNLFFIQPQTGVLTIRPDRALDYETITEFNITVVATDMGNPALSSSTLVGINVININDHTPIFEGIPYTITLSKNISAGSLAYVVRATDDDAGIFGEVRYQITDGNTDNIFRIDQTIDSMGATEEKTGEVFVQGLLETGTYRLVVEARDTPGDIPSSRASSTTLTIVVTDIGDLAPTFPGSSLFTGNVFENAPVGQPITMIPPIRAINSDATGQLVYTITGPDSQTFMINQLSAGIQVNGPLDRETKDFYEFGVIVTDSMGLTAMGTVHINVLDVNDFAPAFNDSVYNFTIPENSPGGYYVGGVSAYDLDGDNVNFFIQTGGEDKFDIGGTSGTITVTPGAVLDREEKPFYTLTVMVSDLRNPPLSGTATVYIYLIDINDSPPKFDASFLDQIISIPEDTPGNTIVTTVTATDDDLNSNINYQIVSVSVYDEDDVDITSFFDYRTVFSLNPTNGNITLTSPVDREVTEKFVFSISARDLNSQNPIFGTSQENAQVTILITDINDNHPAFQPPGTTFILRQLPESFGAGSPIPGNLLALDPDLGLNGEVMYIIQNPSGVPVTIDQNTGQLTLLVTVDREQQPWVNLTVLAVDKGTPSLNTSIPVYLEIIDNNDNNPVFGAQSHTTTIIESEPIGAFVFNVNATDADSGGSGRVTYSLIGGDGKFTINQDSGVITLLQQVDKETQSQHMLTVFARDNPGDSEDSRTGSTTVIVNVLDANEFPPVVTQPIFTIDEGGIGGEVVGVIEAYDPDDPDDPNQSLYYVVVSSQPEVGGTLFIINNATGEIITTGPLDRDNNLHPARVDLDVIVYDDGNPNLGTPTTAIIYINDLNDNQPTFDLPLYDVTVVEGQYSDPIITVVANDPDQNSNLRYEILGGNLNNTFVLVDGGLIPVKPLDFETFTNYNLTVVVTDDSGNRDTTYVAVTVTDANDHNPTFVPDQYSLTVTENEPPGTQVGAVITIDGDTLPEYTDIIYTILTGNGLGNFTINSTTGIITTATILDREKTSEYVLTIGAENPVGGTTGTATVSIIVRDVNDNTPTFEENPYISTVTTNTLLVDVHANDPDEGGNGIVIYEIVSGNEDNRFQIDNRTGVITLVNPLDSQLIRNYTLVVIGHDLGNPRLQSNTTVDLRINYLGLNAPPSFVRPNQGSTVFITENNPLGQLIINAFAVDDDVGANGVVDYSILPNFDYSDFTIDPVTGDITILVSADREIKDSYTLTVVATDRGDPNLSTSSTFVIRVLDENDNNPAFPRPDGMNNPIVLNLNVFENANISDLVGTVDGAIDLDLGANGQIFYHIVDSTVGGIFVINSTTGVVTVNGTLDREDIGVHTIYILATNDATYNGTGPYNVLDNISLKEVKITLLDINDNAPRFTSTVISACIPFDISLGMTVVSVAVVDLDGGTNLGIAYSLNSIQFINSEGERLAAINAFTINSETGQIVTNQRFGEAYLQGYFDIVVQATDVNTGQFAVASIRLCILNNNQRVVVVIDAGIDVVRDNREILIEILQNITGGIVFIDDITTHRDEDGVIVPDSTQVLIHVVDPNTGEVLDANLVTALIDENAMSIEVLFDDLDVIAVYPLFGGGGLGGLGVLEAALLAFGILLFLGALIFAIILCCLRRRLLRKMQGGGVTLGKAHADLLKENRSTSYQGSNPLWLDADTSQPDDWPDSISLLGGTVKDYEAQEATMNFLSDEIPVEIAEDAVVMTSMVSDKSVRNKMSNGGIHRNGYANGFANGSISTVKRVDTTNLNGGTRTMRNNIYEDGTVSAHMQQQHPWGGGGGLTEEHYSSTTSTTRSAPPPRRTETDSRQFSSSLSSRSTPVLRETQNSLRHRKMLTEQTAAEIREMLQDMPPSHLEQQSEMAAAAYREQMEYEAELTPITEEGSMSSRSTNTRGSSYRGQDSLERNKRNGTISKQYQDPMGKFAGESSLEEQYKEEEINGEDSKTKIKTYSAKRVSSKVYTGGEMVPIPELDSDHMLSGIESRLALQDEHSSMSGSSSPQGRSFTYQVLPDSGGSNAENPSVASHTMSSQSDDSGSRNGEFNLAMMDDEDDDDADIVEERRVRRTIKRETSGGDGIGAMGGGGGGGRGYSNNGFNDEESAL

>LvCDH23

MNTRTFPSIPGAVALVLAILSSCAILTSGQDPCNPNPCQNQGTCSGEQGSTSYTCTCTPQFSGLNCATDRDSNAAPQFLYDLIPQVRIIEGQSSTILVLTLNATDPDGDQVTFGLYNDFARSIFTLNEQTGQLYAVEGLDYETLGFQVTVDVFVTDGINDPVLQSIYVRIVDVNDNAPLFTNLPNITYVFENATIGHELFQVITTDEDDLFNSIVTYDISQVIPSQPGVDLTNYFVIGAQTGIIQLATALDYEQVTEYLVTVVATDLGVNTRLSSSATLTVSVLNIQDTRPVFINIPYDVTIPETTPVGSVVQTITALDPDSSDANTIIYNLLVSDSQGYFVINQTTGIITVNTRMDRDNPVLPSTYPLIVSAVETSSGGVSFTQVSFNVTVSPANDEPPSFDENSYTVSVPESAATSSQLPVTITVDDGDLITDSTITVSLQNGNSVPFRVSPTQVFGSGAIDIFITQPLDFDDGTRQYTLQLSAVDESGQDLAPVTVIVTDVNDNNPEFTNRPDDNIYRGSINENDQTGTFIVQVTTMDRDSGQNAAVQYSIISGNLNGVFQINPTSGAITNTQVLGPNTPQTYTLSVRASNPTQGVGVSGEAISTVIITVNDVNDSPPRFSADEFTVYINEAAQVGTTVTRITADDDDIPEGDRLTYAITAGNLGGYFQINPVSGDVTLLRQLDRELESQHNLTIRATDQGAPPQTDMTLLKVIVLDFNDNPPRWSQQNYVESIPEDTPVNTLVLQVIATDDDEGINADLTFAINNANSVPFVIDNEGNIRTSGRLDREMEPQYNFLVTVLDNGSPQQAGNPPATVTINILDVNDEPPVLDETSYEFTIAEDVNFNTFVGQITATDPDLTGTLTYTFVPADPDFSYTAGTIRTNAQLDANQQLIYQMTVFASDGVQNSQSAPVIINVLDVNNQPPSFGMGTYVRSLAEDSPFQTLVVDLNATDPDNGNTGLIIYSFESLSRTEGPFTVDPYTGVVEISTTAALDRETQDEYTLIVIAVDQPASGQPMTGTATVSVIVTDVNDNAPTFRNISYEMSVFENHPLGTNIVTVKADDPDLGVNAVVLYRIISGNTNNMFIIDGQGNINLGSTPLDRETQDQYILIVQAYNSDLSVGTNQVPVIINVLDVNDVTPTFQQLNYYRPDLSENSPVGTPVITVFAQDQDLDLAGDVQYSLVGNPNFLAINGETGVISVTGPISDLNGSRNFTMTVRATDKASPFLSGSATVTVTVVDDTILLPIFTNTRYPATVDENVNADTFVTRVSATLNGSPAGITYTIDPNASPAVLEIFKIDENTGSITTEGAIDRELEDFYAFTVFASHEDSVAPGSTSVWVTIGDKNDNAPVFTAVPSSPVSIPENMLGTVPVGIFTATDQDIGINAQMTFSISGPGSELFNVFQTASGGGTILAISPLDRETQDFYQLTVTATDGGNPALSSSFPINITVTDVNDNYPIWTSNAYDGSINENNFTSNPIITVMVTDRDLPSSNNFEYFIVDGDPMGRFQINSNGEITVSSLLDREEQGSYQLTIELRDQTNSPPAQTTTLALITVLDENDNTPIFDETIIRVNITEGPSSQGIPVYIMSASDIDLGLNKEIIYAFESQQPQSDFEINPGTGVISTITALDWESISEYTLIVTATDQSPTEPRTGTATVIVSVNDINDAAPMFVQDVYGPYSVQEEVSNAYIDTFQATDTDQGLAGQITYSVIGQYSDLFFIQSQTGVLTVSPDRVLDYEDITAFNITVVATDMGNPALSSSTIVGINVININDHTPIFQGLPYTITLNKNITTGSLAYVVRATDDDAGIFGDVRYQITDGNVDNIFRIDDVIDSSRGIERQTGEVFAEGQLETGTYRLVIEARDTPGDVPNSRASSTTLTIVVTDIGELAPTFPGSSLFTGNVFENAPLGQPITMIPPIRAINNNANGQLVYTLIGPESQTFMINQLSAIIQVNGPLDRETKDFYEFEVIVTDSLGLMANGTVHINVLDVNDYAPVFNDSVYNFTIPENSPGGYYVGNVAAYDLDGDNVNFFIQTGGEDKFDIGATSGTITVAPGAVLDREVKPFYTLTVMVSDLRDPPLSGTATVYIYLTDINDSPPTFDPSLLDQTISIPEDTPGNTLVTTVTATDDDLNSVINYQIVSVSVYDEDDVDITSSFDYRTVFSLNPINGSIMLTSPVDREVTEEFVFTISARDLNSQDPIFGTSQEHAQITILITDINDNHPAFQPQGTTFILGQLPESFGAGSPIPGNLLALDPDLGLNGEVMYVIENPSSVPVTINQDTGQLTLLTTIDREQQPWVNLTVLAVDKGTPSLNTSIPVYLEIIDNNDNNPVFGNQSYTTSIIESAPTGSFVFNVNATDADSGGNGRVTYSLIGGSGKFTINPDTGVITLLQQLDKETQPQHMLTVFARDNPGDSEDSRTGSTTVIVNVLDANELPPVVTPPIFTIDEGGAAGDLVGVIEAFDPDHPDDPNQSLYYVIVSSQPEIGSTLFTIDNATGSILTTVPLERDNGLHPAKVDLNLIVYDGGDPNLGTPVTAIIYINDLNDNRPMFDLPLYNVTVVEGQYSDPIITVVANDPDENSNLRYEILGGNLNNTFVIVDGGLIPVKALDFETFRNYNLTIVATDDSGNTDITYVAVTVTDANDHNPMFVPDLYSLSVIENEPPGTQVGVVTAVDGDTHPDYTDIIYTILTGDNLGNFTINTTTGVITTATILDREKTSEYVLTVGAENPVGGTTATATVSIIVRDVNDNTPVFEENPYTSTVTTNTLLVDVHADDPDEGGNGIVIYEIVSGNDDNRFTIDNRTGVITLVNPLEGQLVKNYTLVVIGHDLGTPRLQSNTTIDLVINYLGPNAPPSFVRPSQGSTVTITENNAAGMWIIDAFAVDNDFGDNGVVDYSILPNFDFNDFSIDPVTGNITILVSADREMKDSYTLTVVATDRGNPSLSTSSTFVIRVLDENDNDPAFPRPDGMNNPVVLNLNVFENANVSDVVGTVSGAIDLDLGANGQIFYHIVESTVDGVFVINSTTGIITVNGSLDREDIGVHTVLVMATNDATYNGTGPYNVLDNISLKEVEITLLDINDNAPRFTSTVISACIPFDIRLGSRVVSVSVVDLDGGTNLGIRYSLTAIRFINSQGESTAAINAFRINNLTGEIVSNQQFGEAYLQGYFDMVVQATDINTGQFAVSSIRLCILNNNQRVVVVIDAGIDVVRDNREILIQVLENITGGIVFIDDITTYRDENGNIVPDQTQVLIHVVDRNTGEVLDANLVTSLIDQNAMSIEVLFDDLDVVKVYPLFGGGGLGGLGILEAALLAFGILLFLGALIFAIILCCLRRRLLRKMQGGGVTLGKAHADLLKENRSTSYQGSNPLWLDADTSQPDDWPDSISLLGGTVKDYEAQEATMNFLSDEIPVEIAEDAVVMTSMVSDKSIRNKMANGGIHRNGYANGSVSTVKRVDTTNLNGGIRTLKNNVYEDGTVTAHMDHPWGGGGGLTEEHYSSTTSVTRSAPPPRRDETDSRQFNSSTRSTTVVRENQNSLRHRKILTEQTAAEIREMLQDMPPADLEQESEMAAAAYRQQMAYEAELTPITEEESVSSRSTNTRASSYRGQDSLERNKRNGTISKQYQDPMAKFAGESSLEEQYKEEEIDGQDSKTKIKSYSAKRVTSKVYSGRDMTAIPEMDSGEITMSSVGDIKIVNSRNISSSPQSCPSDHMIGGMGSKLAIQDDHSSMSGSSSPPGRSFTYQVLPDSGGSNAENPSVASHTLSSQSNDSGSRTGEFNLAMMDDDDDDVDVVEERRVRRTIKRETSGGDAMGAIGGAGRGYTNNGYNDEESAL

>ApCDH23

MNCEILDACTSSPCLNGGVCNPSSFGFTCTCAPGFNGVRCTNSDPCGRQPCQNGGSCFESTGPTGFFCRCTSGFIGATCQFPDLCSYSPCLNGGTCSNFPLGSDFLCVCPQGYSGKRCDEFNPCSSDPCLNGGTCNRDSLGQVYSCICLISYVGTNCEFGPNCFSPTDFRCGSGECIPASRQCDQSFDCADSSDENTCSRQCTSQEFRCTSGTCINIQLVCNGINECPDGLDEQNCASPCDSTPCLNSGACMNSGTNFICNCAQGWTGTTCDQVAPRNNLPPTFLNQIISINIPEDTLAGTIIYTLNAADPENDTLTFGLSDFVAQQLFIIVPNPNDPMSAFLTLKTQLDREVQGFYSFVVFVTDGSNEVIHNGNLLLSDINDNPPMFTNLPNTTTVFENAINGPTIFDVNAVDPDSGLGGIVTYSITSVSGVHVGSDALFSINQFSGVVILTGPLDYETNQVYSLDIQALDLGNNSLRTTSTLTVQVLDVQDTGPVFINTPYDRTINEGTPLNTVVVTVLAVDQDTINANNVTYEILGGNLGSFFRVDSVTGEVILVRELDRENINTPAAVELIVRAIEIGSNGAASVIETFIITINDIDDESPVFNQSTYTVSVSEAETPGFVLPVGIEVSDGEIVIQGNFRITLQNFNIVPFSISPSSAVDQAIVTLTLIRPLDYENTRSYDLQLMAEDTAISTATVVVTVIDVNDNDPIFTHGTQTIALEEGSPMGTVITSVTVTDADEGINANTEFNIIAGNQAGLFAINTITGVITTTSVIDFENSASTYTLTIEARNTQPAVSPADGVSEVTVLVTITDINDSPPVFQQPNYLIQIIELSDTGLTIGRVIANDADSGPAGEVTYTILSGNDNGTFLVDPNSGNIILLQPLDRETVSFYNITVQAQDRAAPPLSATTYVIVEVRDFNDNDPQWIRDRYTASVVEGQPANTFVIQVMAQDADIGRNALLRYELAQLSQYLYINLTTGQIFTSMPLDREQTETIEVNVRVRDDGDPRRFSSQLAVVTVTVLDINDTPPTFAGTPYRFEVAENERAGTFVGQVTAADPDSIGTLTYSFAFPQTQFLIDPTMGTITTINSLDREQQDQYNIQVNVTDGTFETTTDVLIVVTDVNDNQPVFSQVLYEVTVAENTPARIILNLMANDSDIGLNSDIIYQFDPSSNTPIGPFLLDRLTGELRTTSSLDRETQDSYTLVVNAIDREGGSGSLQAVATVLVTVTDVNDNRPVFEFQNYAESVAEDAAQGQQIVTVRATDGDTGDNAVILYRIIQGNGNNNFQINSSTGVISRGPTPLDRETEDSYLLTVEAYNDGDLPPRNTATVTITVTDVNDEAPRFTQDVYLKPDLLETAEAGTVVVTVSANDPDLGQGGEIQYSITGGNEGNYFVIDSYSGRITVLSTLPDYSIQSTYNLTVTAQDQAQPFHTATATVRVLLVDAQDDPPEFTMARYEVNLTENVGEGYPFLQVVAQVPGKPNAMVTYSLEPNVNPTILQMFDVDPVTGWLTTKGMIDYETGPPLYTFTVLGMTEAGLSGSAAVWVHIQDLNDNPPVFVTFPNGAVTVDENMAGGFVVATVAANDADSGINADVVYVISGGNEEGHFDIIPNAEEALIVTTTALDRETVDRYTLTITASDQGIPSMNSSIVVEVVVNDLNDNPPVFDQVNYTATVEENVLPNQPVTTILVTDMDASSSNNIQFQIDPASNPNGVFQINSQGEITLTTQLDRETQAFYDLTVIMTDPNYDPTFMETTHVYVTVLDQNDNQPVFPDQPSVTVTEGPSSTGVIFVTVTASDADVGNNSEMVYTITRGNGEGIFGIHSNNGSIYIINELDWETTQTYELIITATDQAVNSNDRRTGTVTVVINVEDINDTPPQFPSSYFGPYSVSEGVPGTYIGPFVAVDMDSGAGGQVTYTIIGEYSDLFIINPTTGVLTLKPTAELDYETSQEFNITIIATDAGMPSLNGTTTVGIIVINVNDNSPRFQDTPYRTSINDTSPVDAWVYKVLATDDDAGPEGEVTYSIFDGNTGGVFRIDPVTGNVFVNKTLTNGVYRLVIKAQDNPANPDNAREVTETLTILVADSTTVIPIFPNNGTFTGSVLEHSTVGTFVLTVSVENEADAGDLTYAISGQDAGPFFVNPSTGVITVNGILDAEIQDHHVFDVSAIDSRGVSANGKVNITILNINDHLPQLDEIVFNFTVPEDAGDGYYVGQVNASDDDNTNTMLRFTIERGAGDKFTIDPVTGVITVLVACRGDICDNQPLDREEQDQYILTVSVSDQGSPPLSNSGVVYIYVTDVNDFQPYFPDNFLDVVISVSEDAALNHTVTTVQAVDHDQTASLTYSIVSVTATNLLGEPIANISAIEGLFGIDPYTGTVYVSETLDRETAAQVVLTISANDSASVDPALSTSNPNAKVTIQITDVNDNPPVFQPPGTTVIVVTVQEESGMGTIITNVEAIDPDDPINGVVTYVLMGNLTQFVTIHPFTGQITVNQVIDRELYDWLNFTIIAMDSGTPSLSTSIPISIQILDVNDHNPVFNQTNFMATVIENANPGTFVIVTTATDQDIGQFGEVTYTLTGGDGLFVINSTTGVITTLQPLDQEVQSIYTLTVIAEDNPGGSQTNSRQGSTTVTVIVGNVNEHAPTTQREFPFIILEEQPGGTLVGVIDASDPDDPMQELNFTFILVEPPDGNVLFFINQTTGEIFTTGPLEADNATYGSTFNITVLISDNGSPPQTTTTTTIITIQDTNDNNPIFPNGPYDLATSEASPVGFQVTVVMAEDIDQNAMLTYRIIGGNINGTFFINPTNGEILITKPLDFETRTAYNLTVQVSDQDGRTGTTYVVINVMDANDQGPVFLRQPYLFDVYENVDLGTEIGHVEAVDADPDPSNSQLTYHILSSVPASAPFVINQTTGVITSTGILDREMQDSYMLTVEVRNTRSVPGEEDVTYKLSTPVTITILDVNDNTPVFAGGDNITRSFPENSQVDFPIYQPSAQDMDIGENSQVTYSIISGNVENTFSIDPQTGAIILARQIQSLTVPTYTFTLVIEARDNGNPSLSSNITLNAVVGDYNDNPPQFVTPSQGQLLFLPENEPIGFRIASVLAVDIDSGANGQVTYGFLNEASAKLFFFQQEGNTTYLYANFSADRETRDQYNLVLLAMDGGTPQPLQTPLEVTVVIVDKDDNEPFFLRVDNNVAIVQTLNVTEHSNVNTSSGYVTTALDLDLSPNNVIYYYIVGGNENGFFGINSTTGEIIVLGDLDRETIATHRLIIKATNNASYVPSGIYDVSKDMSLKEVEIVVRDINDNGPRFTTVLYTTGLPLDAEINTQVTCIKATDLDVGGGGAITYAIQSATFVLGQDQTTFDNIFYIDENSGCIRTRNLLNEVKAGGYFDLTVTATDKTAGLSDTALVRVFVLDTNQQVVVVVDADIDTVTNLQDQLISIIANITGGIVNVDSITYFIDSNGNTVRDQTMIVLHVIDPETNSLMDADIVLRLIDENSKSISLLFQQYGVVDVYALMGGVGGAGFGIIEIALLAIALLLFLGALIFIIILCCLQRKYLKKIHGNSPAVIYATRADANVKDTATYQGSNPLWLETEGGIPDDWPDSISLLGGGAAREYESQEASMDFFSDIHAEVAKDALVMTAMVNDNQTSSVRSRTTSNGGLVSNGRVSMGADQGKLRVVSSSAPLIMDSTNTDGSVSTLTNRINRDGSVTASLSGKDGGYSASGATYSYSTASFSGDGVRPSTSKKLTSEKAQEIRQMLDEDQGVEATFLDEAYQDQTSLAAARASYREQVEFDNQLSPITEEDTASSWSSWTRDSERSGARYAGSGTLDRNRTSGYSSKDYPDPMKNYTTSTSIEESYSRSVKGPGKSESSSFSRRANSKTYSAGVATSIGMVTLTEETPEMLEEDESETSSIEEGAGKTYVYNVRGDSSEGASSGHSATKRATETSLMNFTHEEDDADEVIEEREVRRSVKTTRGFTNSGYDNYLDEESRL

>PmCDH23

MFNPCSSNPCQNGGTCNRDSLGTIYNCNCVVGYDGTNCEFGPGCFSSTYFHCVSGECVPSSRQCDQSFDCQDGSDEASCQRQCTNQEFRCTGGTCINIQLVCNGINDCTDGLDEQNCPSACDSSPCLNGGVCENAGTIYICTCAQGWTGTTCSQVVSSSNDPPSFVNQVISIEIREDTVAGTTLVELRATDPDSDTLTFGLYDFTAQQLFVIVPNPNDPKSAFLTLKGSLDREVQSDYTFVVSVTDDGGNEVIHSGTLLLSDVNDNAPTFTNLPNVTTVYENANVGFQVFYVDATDPDLSLSGLVTYTITEGMDAPFFINLYNGIVHVTGPLDYETDQVYNLAITASDQGTNSLSTTMRLTIQVLDVQDTGPIFIDTPYDRTINEGTPLNTVVVTVTAVDQDTVNANNITYTILAGNMGNYFSLDETTGQLTLVREVDSENLNTPSSVNIIVRAIETGTNGGTSAVNNFTITINDIDDEPPLFNQSTYSVSVSEAENPGFVLPVGIQVSDGEITIQGNFRITLQNSNIVPFSVSPSSAVDQTRVTVTLIRPLDYETTKSYDLVINAQDTATSTATVVVTVTDANDNDPIFTHGTQTIALEEGAPMNTMVTTVTVTDADEGINEETEFSIIGGNQEGLFTINPTTGVVTTTSVIDYENSARTYTLTVQARNTQPAVSPADGVSEVTVVVTITDINDSPPVFQQPNYLIQIIEFVLELSDTGLTIGRVIADDADSGAAGEVTYTILSGNDDGTFSVNPGNGNIVLLKPLDRETVPIYNITIRAQDNAAPPLSATTYVVIEVRDFNDNDPQWVTNQYAASVVEGQPADTFVIQVMATDADIGLNALLRYELAVPSQYLYINQTSGQIYTRVPLDREQTETIVVNVLARDSGNPSRSSAQLAVVTVTVLDINDTPPTFTGAPFRFEVAENEPAGTFVGEVTANDPDTIGTLVYTFTSPQTQFLIDQASGRITTINSLNREQQDRYEVEVQVTDGTFEATADVVIVVTDVNDNQPMFSQAIYPVTVVENTPAGIILNLMANDTDIGLNSAIIYQFDQSGNTPIGPFFLDRVTGALRTTSPLDRETQDSYTLVVNAIDREGGTGSLQAVATVLVTVTDVNDNRPVFDFQNYADTVPEDAAEGQEIIQVGATDRDIGDNAVILYRIIQGNNDNNFQIDATNGIISRGPTPLDRETKDSYVLTVEAYNDGDLPPRNTAAVTITVTDVNDEVPHFTQDVYLKPDLLENADARTVVATVSANDPDLGQGGQILYSITGGNDGNHFIIDSVTGVIRVLGSLPDYSIKSTYNLTVTARDQAQPFHTSQARVMVLLVDAQDDPPEFTMTRYEVNLTENVGEGFPFLQVMAQVPGKPNSIVTYSLDSNVNPSILQLFDVDPITGWLTTKGTIDYETGLPLYTFTVLGVNDGTLPGSAAVWVYIQDVNDNAPVFVNYPTSAVTVDENKAGGFVVATVSANDVDSGLNGNVVYEITGGNDEGHFDIIANNGEALIVTTTELDRETIESYMLTITASDQGVPVMNSSIFIDVVVNDLNDNPPGFNQTSYNATVEENVLLNRPITTVLITDLDTPASNNIVFQIDPASNPNGIFQINSQGEITITSQLDRELQAFYDLIVVMTDPTYNPSFMETTHVYVTVLDQNDNPPIFPDQPPVSVTEGPSSMGEIFVTVTANDADVGNNSEMVYTITGGNGEGIFGIHSNNGSIYVINELDWETTQRYELIVTATDQAMNSNDRRSGTVTVVIDVEDINDTPPQFPSDYFGPYSFSEGVPGTYIGTFVAVDSDSGAGGEVTYTIIGEYSDLFVIDPTTGVLTLKPTAQLDYETSQAFNITIVATDAGVPSLNGTTSVGIIVVSINDNSPKFQDTPYRTGINDTTPVDTWVYKVVATDDDSGPEGNITYSILNGNTGGVFRIDPLTGDVYVNKTLTNGVYVLVIKARDNPENPANAREVTETLTILVADSTSIVPIFPDDGTFTGSVQEHSTVGTFVMTISVENAADVGDLIYTISGPDAGPFLIDPSTGIITVNGLLDAEIQDLHVFDVSALDSRGMSASGKVYVTILNINDHPPQLDELVFNFTVPEDAGDGYFVGHVNATDDDNSDTMLRFTIERGAQDKFTIDPTTGVITVLVTCRGDICDQQPLDREEQDVYTLTVSVSDQGSPPLSNSGVVYIYVTDVNDYQPTFPADFLNAVVSVSEDVALNHTVITLEATDMDLDAMLTYRIVSITATNLQGEPLTNISAIESWFAVDPYTGVVYVENSLDRETAAEVILKISANDSASLDPALSTSNPNAMVTIQITDVNDNAPVYQPPGTTYIYVTIQEESSMGTIITNVRAIDPDNPINGEVTYMLIGNLSEYVTIHPVTGQITVNQIIDRELYDWLNFTIIATDDGTPSLSTPIPIMIQILDINDHNPIFDQPDYTATVPENSDGVTYVTVVTATDQDLGQYGEVTYTLTGGEGLFTINSTTGVITALQPLDQEEQSVYTLTVIAVDNPGGSQTNRRQGSATVTITVGNVNEYPPITQPNFPFDIPEGQPGGTLVGVIDATDPDDPMQELNFTFIFVEPPDGDVLFYINQTTGEIFTTMPLDADNATYGNTFNITILISDNGNPPLTTTTTTIITIQDTNDNDPLFPNGPYDLTTSEATPVGNQVTVVIAEDIDQNAVLTYSIVDGNLNGTFFINPTNGEILITKPLDFETRTMYNLTVQVSDQDGRTGTTYVVIRIMDANDQGPVFLSQPYLFDVNENVDIGTEIGLIEAVDADPDPSNSQLTYHILSSVPANAPFAINETTGVITSSGALDRELQDSYMLTVEVRNVRFVPGGEDVTYKLSTPVTITILDLNDNIPIFSGGDNITRTFPENSEINFPIYQPSAQDNDIGENGRVTYSIVSGNVENTFSIDPQTGAILLARQIQTLTNPTYAFTLVVEARDNGDPSLYSNITINAVVGDYNDNAPQFVTPIQGQTLFLPENEPIGFQIAPVLAVDIDSGANGQVTYGFHNEESARLFYFQQEGDTTYLYANFSADRETKDQYNLVLLATDGGTPNPLQTPLEVTVVILDKQDNEPFFVRVDNVAIVQQLNVTEHSNVNVSVGIVTLAQDHDLPPNDVIYYYIVGGNDKGLFGINSTTGEIIILGDLDRETIATHNLLIKATNNASYVPSGIYDISKDVSLKEVLITVGDINDNGPKFTTVLYTAGIPLDAEINTQVTCIKATDLDLGGGGAITYGIQSATFVLGQDQTASNDIFYIDENSGCIRTRNLLNGITAGGYFDLTVTATDKIAGLGDTALVRIFVLDNNQQVVVVVDSDIDTVTQLQDQLVQIISNITGGVVNVDSITYYIDANGNTVRDKTMIVLHVIDPETNSLVDANIVLRLIDENRMSIQVLLQRYGVVDVYALMGGVGGAGFGIIEIALLAIALLLFLGALIFIIILCCLQRKLLKKIHGNSPAVIYATRADADVKETATYQGSNPLWLETEGGLPDDWPDSISLLGGGAAREYESQEASMDFFSDIQPEIARDALVMTAMVSDGQSASTRSKTISNGGVSSGRVGSDKAQLRTVSSTGPLILDSANMDGSVSTLTNRINRDGSVSANLTGQEGGYSASGAAYSYSSASFSGGPSTSKKLTSEKAHEIRQMLEEDQGVETYLEDDMYRDQNTLAAARASYREQVEFDNQLSPITEEDTASSWSSWTRDSERSGARYAALGSLDRNRTTDRPSKDYADPMNNFTTSTSIEESYSRSVKSPSKTESSSYSRRANSKTYPAGVSTSIGMVTLTEETPDMIDDEESETSSVGGAGKTYTYNIMGDSSEGAHSMSGGSSGHSAPKRATETSTMNFSHEGDDTDEVIEEREVRRSMKTTRGYTNSGYDNFMDEESRL

>ArCDH23

MPCTSLEFTCAPGVCISRNLICNGINNCVNGLDEQNCPSPCDSGPCLNGGACRNVGTSFICTCTFGWTGDRCSIQTSGSNDPPRFDESSIVINIPESTSAGSNVFHLDAIDPDSASLTFNLHDNVAQQLFIVVPDGPNGAWLTLKQQLDREEQHIYNFLVSVTDGFNEVQQSGTLLLSDVNDNAPVFVNLPNTTTVHENVNFGYNVFTVMATDQDALLAKIVQYSISEDTPGPFSIHPVTGVITVSGNLDYETNEVYNIKIIARDSATNSLSSESFLTINVVDVQDAGPEFLNTPYDKAIDEGTPVNTVIQQVVAMDRDTLNPNSITYDIISGNQGSFFALNPNTGEITVIRELDREDPNLPSSVEFNIRATEVGPGGGQSAFTTFNIVINDLNDEPPMFDQSVYSVSVSEAELPGFILPVSILVTDGEAMIQGNFRITLQNGNTVPFSVSPSSAVDQASVTLTLIRPLDYESIKMYNLQLFAEDSATSSATVVVTVTDANDNNPLFSNQLQPITLTEGSPQNTPVITLSTTDADEGTNAVTRYRITGGNQGNLFSINPTTGVITTTSNVDFENSAQTYTLTIEAYNTAPAVPPATGVSVATLVVSIEDINDNPPIFQPSVYDRTVYEDAETGRTIIRVFASDADAGPAGEIRYSIIGGNVDGSFSIDALTGDVILRNRLDREILDFYDIIIRAEDNVVPSQSATAHLTITVGDSNDNSPQWSQLQYMTSIFEGLPANTFVTQVSASDIDLGMNAKLQYYLATPSQYFWINQTTGQIYTSMPLDRELMESITLNVMVRDLGNPQRNSAQPAMVMVTVLDVNDTPPTFTGIPYNFEVAENRPFGTFVGQASAMDPDTIGTLIYSITSQTSNFFINPANGRITTINSLDREQLSSYEFEVQVTDGIHTATEVVSVTVTDENDNDPVFTEGNLYELTVEENMLSGLILRLQATDADEGLNKKIVYQFDPTSNPPLGLFFLDQETGELRTTGSLDREVQGMYTLIVVAKDRNGEAGGRSSVATVLVTVTDTNDNRPYFDLQSYSQSVPEDIPQGGFITKVEAIDRDLDANAVILYRIIAGNEENHFSIDANGTLTRGPVPLDRETKTFYQLTVEAYNAGDSQPRNTATVTITVTDVNDEVPVFTQNVYLRPDLSENALGGAHVATVLANDPDLGSGGQVLYSLTGGNTNFFFIDSQNGIITVQGSLPGYDVQQTYNLTVTATDQSQPFHTSTSTVMVHLVDAQDDPPEFTTDKYEVHLNENMGQGYPFLQIDASIPGKPNIPVTYSLGPNVNPIILELFELDATTGMLTTKRPIDFEAGQNLYTFTVLGESDGVLPGSSAVWVYIDDVNDNNPVFVNPPTDVTVTENVNPGFIFTTVTATDADSGQNGELIYTITGGNQQNHFDLVTDVNGATHIATSTSLDRETIENYILTITAADKGEPTRNSSIDIAVNVGDVNDNAPMYNQTINTASVTENTFINIPVGTIVFSDNDLASSNNIEFYIDDTSNPGGIFQINSLGQITMTGPLDREQQAFYDLVVVMRDQTYNAGFSETSHFYINVTDQNDNQPIFPPQPPVIVTEGPSSTGNIFVTVTADDNDFGENSNVIYTITGGNADGIFGIHQNNGSVYVIKELDWETTKNYELIVTATDQAGNVNDRRTGTVTVTVNVDDINDTPPVFPLDYYGPYSFFEGVSGTYIGTFVASDSDSGLGGEITYTLIGNLSNLFFIHPDTGVLTLKPTAELDYEFVSDFNLTITATDAGTPSFSGNTTVGINVLNINDHSPQFIGTPYRVSINDTEAIGSLVYNVVATDLDSGPDGNITYSIFDGNTGEMFWINPLTGNVYVNKTLTNGVYTLIVKAKDNPLNPQNSKEVTGTLTILVTDSTSTIPVLINNGSFNADVFEHSPEGTYVTTVAVANPADVGQLTYSISGPDAAPFFINPVTGIITVSGVLDIEVKDNHIFEVTAIGASGVSATGTVAVTILNINDYPPVIGQTFNFSVPEDATNDHFVGVVTATDQDYGSTTFHFSIVSGGEDKFAINPDDGTITVSAVCRGDVCDETPLDREIQDLYTLNVQVSDLGSPLLSTQEIVYIHVTDVNDFRPAFDFTNIVVSVEENSMLNKPITTVQAIDQDLSANLMYIIESVTATDMFGNTPTIDVSQWFSVNSTSGVVYVSGQLDRENASEVILTISGLDLASNDAATSTTDPDATVTITITDVNEHGPVFQPDGTTVISTSVQEESNVGTIIINTVAIDPDNPINGMVTYMLTGNFSQYAEINPTTGQITVNKNIDRELLEWLNFTVVATDSGNPSLSTSIPINVQVLDVNDNNPVFNQSEYVTTVTENTGSGTFVIITPATDKDVGQYGSITYKLTGGDGFFTIDSTTGEITTLQSLDQETQGIYTLTVTATDNEGGSDSRQNTTTVLVYVGDVNEHAPTVSPNYYFDVEENLPNRTLVGIIDATDADNPMETFNFTFTFANPSDGNGVFYIDQNTGEIFTNQQIDSDSTLFAENYTFTILVSDNGNPAKTTETTTTIYIKDLNDNNPAFVNAPYNITISELFTGNVMTVEGQDIDKNSVLTYTIVNGNINDTFFINPLTGVITISNRLDFESRKKYNLTILVEDQDGRSSTSHAVINIQDANDQTPVFVGPLPYQFNIDENVPLGTDVGTIFATDADSDPTNSQLTYHITNVVPFTINEMTGLIETSGMLDRETQDRYLLDVQVRNQGHTVATQVLINILDVNDNTPTFGGEDNITRSFPENSQGNFPIYQPTATDADIGENGRITYSIVSGNVENIFSINQKTGAIVLSRPITTLTDLVHNFKLIIEARDNGTNQLHSNITLNMVVGDYNDNGPVFILPNSTILTLPENQPIGTFISNVLATDRDTGANGVVTYGFLNEDGTHFTLQVNGDGALLLSNFVADREIQDRYTLVLLASDGSDTNRRETPLELTILITDENDNEPFFFRFDENTGVVQFLNITEHSSSGTSTGIVNQASDLDLAPFNNVYYYIVGGNGNGTFRIDKNTGEIFVVGDLDRETTGFHNIIIKATNNATYVPSGVYNVKSDVTLKEVQIEVKDINDNGPQFGQKIYISSILTNAEEQTQISCIKATDVDLGDSVDYTIQSVSYTLDGEVTTFDDTFYIEQDTGCIRSNKILTGVGAGGFFDISVVATDKSGKTDNANVRVFTLDDKQKVVLVVDGAREDVIKQQAELIEILKTITGGEVVIDDVTTLINENGVLVPGQTMVTFHVIDQKTNSAIAAAEILRLIDINRATISTLFQQYAVVEAFALGGGAVGAPFGILEAALLAIALLLFLGALIFIIILCCLQRRFLKKIHGNSPAVIYATRADADVKEAAAYQGSNPLWLDTADSYPDDWPDSISLLGGGVNREYESQEASMDFFSDMQTEVARDALVMTALINDGSIQGQAGAGRVKTLSNGAVASNGRVASNGRVTSKGRLAGSGVTASNGGYGSGAGRSSAGMAGSGRSNTGMSSTTTTQLSSSGARMVDGIDGSGGTLANRLNRDGSVTANLNSGVQDGGYSGSSASYTYSTVGFGGPGPSASGSQRLTNEKAHEIRQMLDEDETVEATFLDGTMYGIGDNSAGMAAARAVYREDVEFENQLSPITEEDTTSSWSSWTRDSDRSGGRHPGGTLERNRTTGRSSKDYQDPMTNYTTSTSVEESYTHSIKKPSQTESSSYSRRANSKTHSAGVPTSIGMVTLTEETPEMIDLGPDEDSEAESSIVDGRTYTYNVMGGDNESNHSLSGTSSGHSSSRRKETNMITQEDEDDDDEDGDVIEETETRRSKKTTRAYTNSGYNDYMDEESRL

>AjCDH23

CAIDESPTDNAPRLDSIPPSVIRISESATVGSLILTLSGSDPEGATVIYGIDQPEPNYLSVGPVDGEVRI

RQQLDAETDLFFQVDFTISDGTNLPVEYPSFILVTDVNDNAPQFVNTPYRFQVPEDAPIDYLITTISTTD

PDTGIQGAEYQLLENTTIISVNLTSGRVLLKQQLNHEVTKTYQFTVQAFDLGSPRLENTTDFILEVQDVQ

DSLPVFVNTPYTSTVQENAPIGTIVTTVSAIDQDTGVPNVISYSIVGGNEAGFFYINPATGNVTVNSVID

RDMEGFSGIVQLTVMATELGSSGGANVSTIFSIMVEDVSDQAPIFGQPQYDVSISESAQQGQQLPLQILV

SDADMGTNADFSLRLQGNHSELFSITPLQASGSTEVNLTLLQSLDYESSTSYSLQIVAVDTTNTELSSEA

TVMINVINENDNAPIFPPVPYIANLQENVAVGSPVIQVIAADNDLGSLGQITYTLSGSDRFAINSGTGEI

SVAQPLNYEESKNHVLTVTASDGGVPPRTADTSVTVYVTDVNDNAPQFTREIYEAYVEENEMYSVGREIT

TVRADDPDLSSNNLVNYFFLKTFPNGTTATSTTFDNFIIMASLGSGYIYTTQTLDFETIMSNPLAITVVA

EDSEGLRDSATLNIHVIDKNDNAPVFKQPSYEATIPENFANGDEVLTVSATDSDGSSIYGSPSILYSLKP

PNSYFTVNAVSGSVSTIATLEADDGTETIEFEVVAMDGGQGLSQMSSSAMVTIRITDVNDNDPVFVDNKI

TAYVPENATVGHLVAMVSTTDRDQLNNTDVTYSIISGNDNGFFTIDPLLGNISTTGNLDFETTGSFTLII

LAEARTSPVPPATGAATAIVEVFLQDVNDNAPVFTKEVYNFQVSENEVPGDVIGDVSATDADSTVNAEIQ

YSIISGNENGLFTLLTQSRGVLTLAQPLDRETSPSHVLTIRAEDLGQPSLSSETTVRIMVLDANDNDPIW

IQDTYSVNVTDGSSPGLLLQVQATDSDEGKNSQLIYTIKPPNADFFINSSTGELFTVTEIDREESSFITL

FVNVRDDGDESRSATSSAEVEITVIDVNDNAPVSLLPDYSFTVEENSDSGTIVGRVQASDADSTSHLLYS

FVNSSMEFTINITSGEITTQRSLDRESKAHYTLTAMVIDGKYSVSVPITITVVDVNDNAPQFTKAQYEVP

ISEYAVPGSPVIQVLAIDNDEGDNGGVMYSILDGNYGNAFRIDPATGRMQVATSLDREVTPMYTITILAT

DRAEGDAMSSTALVIVTILDENDNRPLFTQNVYTANVAEDVPMGTNIIQVSATDADVGVNADITFSIISG

NENSLFAMNSTTGQISRGMNAFDFESAGLHSLIVQASNQDGEQSTATVHITIDDVNDEMPQFTQELYQRP

DLSENALAGDVVVIVSATDRDTGSGGVVSYSIVAGDELGLFGMNPTSGALTVVGSLDYETFRNYTLTVAA

TDQSPPNNVGYANVKIMIVNVNDEVPRFERTRYTEYVYENVMVGYSVAQVTAIDRDGQASVVYSIDTNGD

ASNLFDINSTTGEITTKEHLDRETSPEISFTIFASDGGIERGSALFSVILLDVNDNKPMFDVSSEVIAEI

MENSPIGSLVTSFIATDTDFGPNSQISYNFISGNEEGRFSLSVLPDGWVQVNTTTDIDREMQSSYLLNVS

AIDNGIPSQHNNLLIRVVIQDANDNTPEFSAKEYSATIKENSVANTVVVNVQASDADDDQNGAIRYDITA

GNPGVFAIDAISGNITTTYTNLDREVKEFYNLTVVARDLGSPSKKSTTYVEITVLDANDNAPIFEESSQT

VNIIEGTSSVGIVVATVLATDADLGTSGDVQYFIEDGNIGDVFLINSTTGVITTLATLDREVVEEYVLTI

TAIDQDSNENNRKSSTATVTVQVEDINDVVPTFPSSFVGPIFVQEGIRGEYLGTYIAEDNDIGLSGNILY

SLGNESRDMFMINSVTGILSLKINVVLDYETKKEYNISILATDRGSPALVGMMVLYVQVLDQNDNSPEFN

NLPYLATVPENISVGASVFQVNAYDIDGADFQPIRLSIIEGNYGAVFRIDETGTVFVNNSLDRETTSLYE

LVIAATDNVQDPDNSRTVSCTFVVQLFASDPDGYPLTFKLDNGGLNKFTIDSSSGVITVATTLDFEQQKF

FTLTISASDTGSSPQTGYSTVYLQILDINDSRPKFTKLISNYVIQENSPPMLIGNVTAEDDDTSSNISYT

VMSLIAFDGNSIPISDDSLFQDFIRLDSMTGDIYANEGIDREIAVRLELTINARDLAAFNPDYYDSDPDA

VVRIEISDVNDNAPAFSSANIIQSVAEGTPIGTVILNVNANDPDSGLSGAVLYTLINTTEIITIDNTTGQ

VTLTQELDREANEELTFLIKASDGGVPSLSSILHLDLLVLDINDNNPVFSQTYYHVEIPENIVVSADVIQ

VTATDIDEGSFGTVMYSLSGGQGKFSINQMTGLVIVSAPLDKEEQGSYTLTVTARDNPSGSVNNRRESSA

QVIVTVEDVNEFPPLASMDQYTFEVEENLPADTLIGQVVAEDPDNSPNQQLTFTITNSTSPEFLSLLRIN

SSTGELFTTVSLDRESNAYPSTTILTIEISDNGFPQMSSQPLVEVEILDVNDNDPEFLSPTYYANISEDS

SIETLEISSENETNKQMRIRAAQQTRAMTELLEKTARYYVGRCIAVDNDAGSDTSQIIYSIIGGNSAKAF

AVNSSTGELETTTMLDRELVASYTLIIQASNQDGLASTTQVSITIEDINDVVPHFEPSFLPIQVLSESVD

IGTVVVTVTAKDEDEGANGEVVYSIIGGNDGGVFTIDQQSGTILTVQLLSEQNPQVFTFDLVVMAMDEGD

DPLSSTTVVSIKINDENNHKPVFISPVAGQRLTVPENVDAGYLITIVSATDGDAGDSSIITYGFDDSRTE

DHDILLKFSIDPNTGRLLSNTTFDREMQETYTLVLTAQDNGVPRQTTQQEIIIEVSDVNDNEPGFPTIEP

GVPPVQVMSVPEGAVNGTYVGTVMEAVDPDKEKDYYYFIVDGNDPVRFNLNKDSGELFVVGPLDREVGSA

VSLIVKVSSDPVYTTTQRRKRATTDLLSDRSLLEVIINLEDVNDNGPVFAEAVYIAGVPAEADPGTEIFT

MTAVDADSAEHTSTEYAILATASVKDGVQAEADSTFSIYKKTGLITLSKKVDGDAYEYFDITVRAMDTLS

SIESLANLKVYVLDPNERVALVVNAPIEDVQAKQDQLIEVLSNITRGIVFIDDIVLYTTENEKGDPEQTL

ISLNVVDPETNEAMPADVVLMRIDENFQLINALFDVVDSYPVITGSGGQNWALLEIALIAIAILIFIGTL

AFCVIVCCLRKSYRKKLSMRHAVSFGNAQNLLDNNPYDYQGDNPFWLDGEASMSDWPDSISLIDSRQVPY

RDGPMDFFSGTTTFDASHKAKNGTIQNALPPPPPPPGQSSSNTPSEASSIELLKQELAAYGSSLDPRAIP

INESELEETHSGMLTPKRVKEIHQMLEELTSTDSAAAAYRQELDYDNQLSTIAEEEASMISSLTEGSSLE

HGRHNHDLYAKVMRPTTIQEDGECADVSSESTSSSTQATVLSVTTYKLKTNGDKDEPIVNGHVTEIESEE

SDSESDGCKKHTYTVNHSSEFSDDDSVHDSDKNSDISTTTAKQRTYVYTINADDDGSKEQSEAANQRLRV

DMGGEVALSDDSSVELMQEISDNNNFSFSGVTNPAFHEEETHL

>SkCDH23

MAGQLKTLITLLLSFTILSYVNGDEPPRFITDITSLQLREDLPLGTEILVLNGYDAEGPVHYALYDDISQELLDVNSVNGSVKLKKSLDREVNPHHEVIFSVVDNMGQQQPMETSIYVLDVNDNAPVFENTPYKVSISENVTVGTTIFEISATDSDSGRNGIIFFSFSTEINSFSIPSPNRYVIINQPLDYEDINIYELTIVATDQGIPQQSSEASLIIKVTDVQDNPPMFTGLPYTTSVDENSPTTLVVQTVRAFDQDIGLSNQISYSIVSASVPELFTINADTGEIRGQRLIDREAEGFPGYVTLTVEALEVGPDGGDSAQTEFTITINDVNDESPLFSQAEYSITVSENAHIALPLDIHVRDRDQGEFSTISLSLHGQYSELFRLEPSRVLGEADVIILVDHPLDYETAVQYQLYIYANETADITHFDMTRVRITLINENDNRPIFSSQSITVDVKEDLPVGSNVTMVTATDDDIGIYGDITYTISGADQFIIDAELGVISLIQSIDYELSLRYQLTVRAEDGGNPAEADNTRVIINVIDVNDNPPVFKDSEYFATIEENTVYLNKSIVRIRATDRDSAPYNQITYHILDAPSEDQFSVTTDGVNGFVFVNTPVDYEELIDGGLTLTLSAEDLDGLSDVAVLYIEVLDKNDHGPEFTHQTYNVSVFEDIQTSDTVTTVTAIDGDMSSALGTESIVYSVSQPGVFRINPGTGEITTTTFLDRDMGVDSYIVQVLAVDGGHGNEQKTATSMVYITILDVNDNNPVFENVRQTVYIAEDQPIDTVIGNVTASDLDFGMNGDIIYSFISGNTDDVFSIGLFDGEILIKKELDYDDLYQVYILEILAHDQGVPVDPATGSATATVTVSVIDVNDNSPVFEYTVYQFAVSEEADPGITIGMIRATDMDSTTNAYIDYNITDGNIDGSFNMKRVGSGEIILERQLDRENIDMYGLTVIASDRGIPRHSAVCYVTIIVRDVNDNDPIWIDEPYMTTISEDVDNGTYVFQVSAIDADIGSNQLLVYEINPFTPYFKVDSASGVITTTDVPLDREMNTAQEIILTVKDSGRPLRRSSHSAIVHVTVLDINDNVPVFEDTPYIGSVVENQPAGTSVIQVSASDLDFGENGTVSYFIVSLEPNTNNFDMNSSTGLITLTHPLDREYIQAYNITIQARDGGMDPNVAIATVQIEIEDDNDHSPLFTANSYEVSVLENATRGTSLIVLSASDGDIGTNADINYFITHGNQDGKFQIHFQSGLVQTSAFLDREVVEEYNLTIEAIDNGVYAKTGTCYVTVNVLDINDNRPIFTESNYDVTVMENVTQDYTIVTVHALDADIGENAKITYAIIAGNTQNSFVIDSNYGDIRRNEQPLDRETEESYILTVEASNNDSNMFRSQVRVSMMVYDVNDEKPVFTQSVYYRPDLSESAGKGTSVILVSAEDPDLSDGGRVVYSITAGNDMDEFSIDSSTGLITTDAALDYETKNNYSLVVMAVDQSPPYHSGTASVVVIIVNINDEPPSFNQTRYYSTVKENVAIGTSVVKVTAVEYDNQNPIQYEFDPNTNPEAMALFTINIDNGLISTINEIDREIRGFYTITVLANDGGTEKGSTTVWITVLDKNDNAPSFDVFSDISVSVKEEMYTPVGSMIGRVKATDRDEGINAMVNYEIIDGDVNNMFNITTNTVNEGIIKNIQLLDREVKDTYRLIVSAYDSGDVPLNTSMIVTINIEDVNDNIPNVGGGTIITTVYATDEDIGSNGVLKYYIVDGNTDGTFRMDRNTGEISTRPDPPDREKQDFYNLTVLAEDEGDDVIQQVTVTVLDINDIVPYFDASFLGVYEIAEDTSGPFIGTFRATDLDEGNNGHIQYSLYGDIYDEFSISPSDGDLRVRRGVELDRERIDIYNITLIATDMGNPPLSGSLDRETTSMYILTVTAKDNPENPTNARRDGTELIVTLLDENDQIPTFTQSLYSGHIEENSQEDNTVTMDDAVLAVDADFGNNSVITYSISGNGSQYFYIHQHTATIHVKETNSLDREAENIYRFLVTAIDIGGLNSSAEIVVNIVDVNDNSPVFTPSNMTVFIPENVVGGHVVMEVLAIDADIGLNQEVTYRVESGGQDKFTINPNTGLIKVSIASTLDREQRDQYTLVVIATDRGNPSQSGTGTVSIIVGDINDTPPSFTVLSQNFYLRENSPVGTTAAWVIAVDPDFDSLLQYSIVSIAAIDEEDNIVADSNLYENWFSINEFSGTVQVAGILDRENVSVFQLTISAVDLSSQYNGSRHSNPNAEVSIHLLDVNDNDPVFQPPGLEYIHEQLVEQSPLDTVVTTFTALDRDKGLQGMVRYEIVDNKTDLLQISDPAVGVITVNGVVDREIYQWLNFTVKAWDFGNPPRYSQIPVFVEILELNDNNPIFRQSLYETSVVENAIAGTEILVVIATDADSGSYGEVRYMLAGGAGKFTVDEITGAISLVHSLDREIQSEYTLTVTAKDNPTGLSSDRRENSVLVIVDVLDVNDNVPIPGAANYRFEILENVPVYSAVGTVVAHDADSGANGVLQYIISEVSEDDLFDINSSDGQIFTINSLDRESLGNKEMVSMVVKIQDQGVEHHEVTVPVLITVLDVNDNSPYFAYAMYNVTLSEDDFGGTLVYQFSAVDLDQDPVITYTLLDADLYSEFTVSEQTGALTTVSSLDHESRPEYLLTIEASDEDGNVGTTQLYIRVLDTNDQAPLFAITLYQFSVKENESVGSLVGQVVATDSDSVMEHSKIFYHIINGNPDNDFYLNENTGVVLINKQLNRESRAMYELEIEARNVADGIQSNVTLYDLATVIISIEDINDEAPMFTKQQYSRRILENEQIDTSIITVTAVDFDDGTNGEVYYAIEEGNYNVYVKKLVAVVSCNTNQEPGYFITTVIATDGDLGDNGVVIYDFFKTEGDNEDWQNFYIDETRGDLYTTFTADRELKSFYTIVLLAKDLGEPQFESTQAVTIKVLDENDNIPIFSNVEVAQTMYISEHSTNGTIVGEIEQAIDLDEGDNAKVYYYFAGGNSDGYFEIDKTTGIIILMKEVDREKVNQFVLTVKASSDPDFGEDIEERRRREVIEPIDMNDINDPTLKTVVIKVVDINDNGPQFPRDEYTAGLSLDAKYESKLITVTAKDEDTGNNSIVYYSILSIYHLDTTTDLPPRVQPNAFKIGRLDGIIRTNELFVSFYHGYFELNVLAEDNGHKNDTAIVKIYMLKDNQRVKIVFNNDPDTVKEFTDQFISLIANITGALVHVDDIQYHISDTGGVDFKRTDMLIHAVDPNTNSIMDVHHIIELIDANYADLENFFKVYNVIEIVPAVPSREEDRISLLEAALVAIGIMLFIGVLIYIFCICWIRHKHKNKLEAIATFSYGASTSDLLRDSSYQGSNPLWIDNYSWISDWPESLSHFEREYEAQELTMDFFHDDDGSMRDTSSLMAKRHMKTFSPLPNGIDRRSQSPPSAESRSPSGSDFEIELIQSVTSGESDTDESPGVLGANGNNTEQELSNDYVGLKDYSIFITPRRMLTPISEEDSGSLTSSCKTDTMKTTGSRFSGIQSDYEEDESEESNDERLTLTVLDNLDSSFRINEKGASGAVNHSDKYQPLENHSSQDTDSESSSGSDGAYQIYHVNSEPRDFSLNIPLDDRDAAPLTQPQYLEANHGVENNAFEYDFMVFKSSDEDNSDSSKESIQVSDIDNRNTSNILSAALAHSELETFL

>BfCDH23

MVLPTVPFPELSVLVATVVVWTSHTVQGNNPPFFTMTQQEWIWTLPEDTPRGTAIAQLTAVDPEGKSVLYGVEGEIGQRYFLVDPVTGIVELYREFDREVDDEYTVKFSVIDDAPANKVEHEATIYITDVNDNPPTFEGTPYETKIPEDLPVGSTVFTVQARDPDKGAGGTNTFFFDPDGGNPWLSTSTNLVIQVKDVQDSDPVFLGLPYDATVREDAEVGHVIVRISAMDQDSGIPNDIQYSIVTGNVDNVFDLDDLTGELRVAKVIDREAEGFNQGPLTITVQALEIPTGDSPGGAFIRTTFDIAIIDVNDETPTFDQSEYSITVSESTLVGAALPLTLQVNDPDEEENGAYMLHLVGLTAPQFLVSPSSAMGEAAITLRVNKPLDYEVEQMHLLEIFANETNGEKYGHATITITLINENDHRPTFTQSTYSLSVEENIPIGTSLLNVSASDRDIGEFGDVTYFINDDDPRFRIDGNTGVIYTLDQLDYEDAWRFSLTIIAQDGGTPPQQTAARVRIDIVDLNDNRPIFQRDDYVGTVRENEASQTPILRVRATDEDSPPYNILVYNITSGDSLHNFTIGTEDGYGYIYTSIIFDSIFFPFPPLRGRATDEDSPPYNILVYNITSGDSLHNFTISTEDGYGYIYTSAPLDYEMMTGNLFELMVTATDGGNHTLNSTTRVVIEVEDQNDNGPVFNQQEYNVSVREDISAGDTVLRVFASDADMSEELGQDSVIYSMSGSSRFRINPRDGEVTTTTFLDHERETDYVLEIKAVDGGQGLDQRTATVFVNITVLDVNDNSPVFVQPEYHTNILENITDMEIVVQVNATDEDSTTNGDILYAITGGNTDDAFIINPQSGIIQRGVVPLDRETLDGYVLTVDAYNGGKPEMRSTVRVNVRILDVNDVSPVFTQTQYGRLGLRESAGVGTSAVLVRAHDPDLELGGQVDYGFVSGDRGKFAIDESGLITTSAELDFESWRNYTISVYASDRAPPYNTGYATVVIVLANENDEPPEFNAEHYDAAVSENVDVGTSVLQVYATSPDNLAAIEYLFDPNTNEEVLRRFSINGSTGLISTIEEIDREEAAQYDITVISTDGGIETDSATVSIVILDENDNFPKFNIFSQTDVSVTEGPLTPNGSDIARVIARDPDDADNGRVEYTIEEGNDLEYFKIEKTESGDGIIRNLHPLDREEQEEYKLRVVACDQGSPQLCNNVTVVVTLEDINDNVPAFIFNSSYRYNVSVQENVGGGTTVTQVQAVDIDAGDNAFLSYYIISGNEDSMFRMDRLSGDITTRPNSPDRETKDFYNMTVLVEDEGSPQLQAYTTVYISILDENDNAPVFEFPTYSYTLREGEGSAGIYIVDINATDIDQGLNGKVVFNIASGNDGNEFDIEPDTGKIRTVNELDYENSAGEYRLAVIASDQAPDVNDRQTSTTTVTISVNDINDVVPHFLREHVGPVRMAEGLLGSFVGSFPAFDEDSGDFGKIDYTIDDGDENDEFYISLLDGDLKVKQGLELDRERTAFYNITLRAQDRGRPPLKGFMTVSIEVLDINDNDPIFQDLPYYCRVSENAPVDTLVFKVSANDTDEGNNAFITYSITSGNIGQVFRINGTNGEIYVNGLLDREVIPQYTLTVTARDNPQNPTHVRRDTTQVYITVIDENDQRPRFTQPLYRGRISENSQAGTAVDMSEDILAVDADATTNAEVHYSIFGKDARFFRIDSVKGELFVRDGSLLDRETIGNDLSFTVMAADPGGLNESAGVYLEILDQNDNSPIFISTLFRANVSENAFPGTSVTRIEASDQDDGLNQLITYRIESGGQDKFTINPETGVIRIAHRQALDREARDQYVLVVKATDHGIPTRSGTSTVLIEVEDINDSAPYFLQYIHTATVSESKEVGSWIMNVTAVDRDLDSLLDYSISAVVATDLQGNHIVNGMGYLDWFRVDQFGGGLFVNSTLNRERAEVVQLTIGVEDLASDVPQRARSGRDAQVTISIIDENDNTPTFQPPGVAFYQERILEEVPVGTVVMSIIATDADKGKNGIITYRIVNGSLATEFFAIRDKYLGTITVSRPVDHEQHQWVNFTVQAADEGKPVRTADIPVSIEIVDINDNNPIFSLKKYQASVLENQPAGTEVLRVEATDRDSGSYGRVQYHLSGAGGRFAIHPDQGNITLTTPLDREEQTDFLLSVTATDNTDGLPSNSRENSVQVVVTVLDENDNTPEPSTSKFDFRVPENQPAGEYVGTILASDRDEGDNGRLQFSILKQEPNHVMLFDIENTTGVISSARLLDRETMGYNGIVELTIQISDLGSPPRMSSTKAVIKILDSNDNSPYFQQSSYSLAVREDEYGGTSILGLVALDDDEGNVLTYSITAGNEHRDFAVGASTGHVIVAKSLDHETRPAYTLTVTAFDQDGRNGSTTLLVKILDVNDNPPVFSQDVYRVEVMEDIQPGALVGQVEATDADALPQNKKVLYHILSGNVYNTFRIDTESGEVFTQAAVDRETLSLYQLTIQATNPSGDSQSVNGTTQITAQLLPWKSTQMFITIADVNDIAPVFDEEEYARPILENEPIGTSILYVHAEDGDLGNNSRLSYSIAEGNVDNAFKINEKSGLVMVNTILSEKARIINEYRLEVVARDHGTPPLEGRTIVLLTVVDVNDKKPRFVRPQRNMTVSVPENSPSGHFVYQAFAVDEDTGINGIVSYDFFIPNGRERGGDWEKFAIDPFSGNITTVEPLDRELQAEYTLIIVARDQGLPEPFESTRPFRVEVEDVNDNEPEFPRDVSGLVYMQTLSVPEHAEVGTEVGRVTEAIDKDEGDNAIVFYFITGGNEGKFFLLDKQTGILSVHRDLDRETKPVHTLLIKASSDPDYQVPRQRRRVIDVSDSSLQQLVIEVEDINDQPPRFTRKQYTAGVTVDAKYGSELIQIEAIDADIGNNSVVYYYIESTTYIERGKQPKPMKKTFQLDVLSGVLTTNELFVDYTKGYFELIVRAEDDAKHTDKSKIGVYVLRDEHRVKVVINDIPDYVRTFREQFIAFASNITGAVVNMDDIQYHVDEGEIDFTKTDMLIHVVNTKTNKIMDVDNVITTIDENYDSLERLFKDFNVIEVLPFVPPRTDKDNIDIVRILLILIGVVIFFGAWIFICLMLRYKRLYKRNVRAAIAGSIAGAQVIQNPNPAATPVTSHQETTHPGGGAGGAPPPAGGAADPRYSQMMGPGVGMEMFDVPNTNKYSFEGANPIWLDPYYSQFAEFHEPTEIPVVLEDGFTIEGDEYETQEVSMEIHGDQEDESPKKGSHSSDAILKSVLNDHWPNNNSSQGDRAPRPPPRRVGVHSENNNNSNKRNAAANDSSEGSLKTGSLMTRTGSSTVIVNPAVLQDCRDVISSGEESDEENSRSGSSGSNSQSGMIKEKEVHLWKT

>MmCDH23

MRYSLVTCYAVLWLLMLVPGSWGQVNRLPFFTNHFFDTYLLISEDTPVGSSVTQLLARDMDNDPLVFGVSGEEASRFFAVEPDTGVVWLRQPLDRETKSEFTVEFSVSDHQGVITRKVNIQVGDVNDNAPTFHNQPYSVRIPENTPVGTPIFIVNATDPDLGAGGSVLYSFQPPSPFFAIDSARGIVTVIQELDYEVTQAYQLTVNATDQDKTRPLSTLANLAIIITDMQDMDPIFINLPYSTNIYEHSPPGTTVRVITAVDQDKGRPRGIGYTIVSGNTNSIFALDYISGALTLNGLLDRENPLYSHGFILTVKGTELNDDRTPSDATVTTTFNILVIDINDNAPEFNSSEYSVAITELAQVGFALPLFIQVVDKDENLGLNSMFEVYLVGNNSHHFIISPTSVQGKADIRIRVAIPLDYETVDRYDFDLFANESVPDHVGYAKVKITLINENDNRPIFSQPLYNVSLYENITVGTSVLTVLATDNDVGTFGEVNYFFSDDPDRFSLDKDTGLIMLIARLDYELIQRFTLTVIARDGGGEETTGRVRINVLDVNDNVPTFQKDAYVGALRENEPSVTQLVRLRATDEDSPPNNLITYSIVNASAFGSYFDISIYEGYGVISVSRPLDYEQIPNGLIYLTVMAKDAGNPPLYSTVPVTIEVFDENDNPPTFSKPAYFVSVLENIMAGATVLFLNATDLDRSREYGQESIIYSLEGSSQFRINARSGEITTTSLLDRETKSEYILIVRAVDGGVGHNQKTGIATVNVTLLDINDNHPTWKDAPYYINLVEMTPPDSDVTTVVAVDPDLGENGTLVYSIHPPNKFYSLNSTTGKIRTTHVMLDRENPDPVEAELMRKIIVSVTDCGRPPLKATSSATVFVNLLDLNDNDPTFRNLPFVAEILEGTPAGVSVYQVVAIDLDEGLNGLVSYRMQVGMPRMDFVINSTSGVVTTTAELDRERIAEYQLRVVASDAGTPTKSSTSTLTVRVLDVNDETPTFFPAVYNVSVSEDVPREFRVVWLNCTDNDVGLNAELSYFITAGNVDGKFSVGYRDAVVRTVVGLDRETTAAYTLVLEAIDNGPVGKRRTGTATVFVTVLDVNDNRPIFLQSSYEASVPEDIPEGHSIVQLKATDADEGEFGRVWYRILHGNHGNNFRIHVGSGLLMRGPRPLDRERNSSHVLMVEAYNHDLGPMRSSVRVIVYVEDVNDEAPVFTQQQYNRLGLRETAGIGTSVIVVRATDKDTGDGGLVNYRILSGAEGKFEIDESTGLIVTVDYLDYETKTSYLMNVSATDGAPPFNQGFCSVYVTLLNELDEAVQFSNASYEAVIMENLALGTEIVRVQAYSIDNLNQITYRFDAYTSAQAKALFKIDAITGVITVKGLVDREKGDFYTLTVVADDGGPKVDSTVKVYITVLDENDNSPRFDFTSDSAISVPEDCPVGQRVATVKARDPDAGSNGQVVFSLASGNIAGAFEIITSNDSIGEVFVAKPLDREELDHYILKVVASDRGTPPRKKDHILQVTILDVNDNPPVIESPFGYNVSVNENVGGGTSVVQVRATDRDIGINSVLSYYITEGNEDMTFRMDRISGEIATRPAPPDRERQNFYHLVVTVEDEGTPTLSATTHVYVTIVDENDNAPVFQQPHYEVVLDEGPDTINTSLITVQALDLDEGPNGTVTYAIVAGNIINTFRINKHTGVITAAKELDYEISHGRYTLIVTATDQCPILSHRLTSTTTVLVNVNDINDNVPTFPRDYEGPFDVTEGQPGPRVWTFLAHDRDSGPNGQVEYSVVDGDPLGEFVISPVEGVLRVRKDVELDRETIAFYNLTICARDRGVPPLSSTMLVGIRVLDINDNDPVLLNLPMNVTISENSPVSSFVAHVLASDADSGCNALLTFNITAGNRERAFFINATTGIVTVNRPLDRERIPEYRLTVSVKDNPENPRIARKDFDLLLVSLADENDNHPLFTEGTYQAEVMENSPAGTPLTVLNGPILALDADEDVYAVVTYQLLGTHSDLFVIDNSTGVVTVRSGIIIDREAFSPPFLELLLLAEDIGQLNGTAHLFITILDDNDNWPTFSPPTYTVHLLENCPPGFSVLQVTATDEDSGLNGELVYRIEAGAQDRFLIHPVTGVIRVGNATIDREEQESYRLTVVATDRGTVPLSGTAIVTILIDDINDSRPEFLNPIQTVSVLESAEPGTIIANVTAIDLDLNPKLEYHIISIVAKDDTDRLVPDQEDAFAVNINTGSVMVKSPLNRELVATYEVTLSVIDNASDLPEHSVSVPNAKLTVNILDVNDNTPQFKPFGITYYTERVLEGATPGTTLIAVAAVDPDKGLNGLITYTLLDLTPPGYVQLEDSSAGKVIANRTVDYEEVHWLNFTVRASDNGSPPRAAEIPVYLEIVDINDNNPIFDQPSYQEAVFEDIAVGTVILRVTATDADSGNFALIEYSLVDGEGKFAINPNTGDISVLSSLDREKKDHYILTALAKDNPGDVASNRRENSVQVVIRVLDVNDCRPQFSKPQFSTSVYENEPAGTSVITMLATDQDEGSNSQLTYSLEGPGMEAFSVDMDSGLVTTQRPLQSYERFNLTVVATDGGEPPLWGTTMLLVEVIDVNDNRPVFVRPPNGTILHIKEEIPLRSNVYEVYATDNDEGLNGAVRYSFLKTTGNRDWEYFTIDPISGLIQTAQRLDREKQAVYSLILVASDLGQPVPYETMQPLQVALEDIDDNEPLFVRPPKGSPQYQLLTVPEHSPRGTLVGNVTGAVDADEGPNAIVYYFIAAGDEDKNFHLQPDGRLLVLRDLDRETEATFSFIVKASSNRSWTPPRGPSPALDLLTDLTLQEVRVVLEDINDQPPRFTKAEYTAGVATDAKVGSELIQVLALDADIGNNSLVFYGILAIHYFRALANDSEDVGQVFTMGSVDGILRTFDLFMAYSPGYFVVDIVARDLAGHNDTAIIGIYILRDDQRVKIVINEIPDRVRGFEEEFIRLLSNITGAIVNTDDVQFHVDMKGRVNFAQTELLIHVVNRDTNRILDVDRVIQMIDENKEQLRNLFRNYNVLDVQPAISVQLPDDMSALQMAIIVLAILLFLAAMLFVLMNWYYRTIHKRKLKAIVAGSAGNRGFIDIMDMPNTNKYSFDGANPVWLDPFCRNLELAAQAEHEDDLPENLSEIADLWNSPTRTHGTFGREPAAVKPDDDRYLRAAIQEYDNIAKLGQIIREGPIKGSLLKVVLEDYLRLKKLFAQRMVQKASSCHSSISELIHTDLEEEPGDHSPGQGSLRFRHKPPMELKGQDGIHMVHGSTGTLLATDLNSLPEDDQKGLDRSLETLTASEATAFERNARTESAKSTPLHKLRDVIMESPLEITEL

>SpCDH88C

MQTSSNFCARFHIRSYQGKMSCLLTILVVAATVSLGQGAVNWEEDLEYTEISEYLIPDDYIGTLLATETTDASKTFQYGIYRIIANIPSGYVDAFDYLEVDPNSGNVTVKALMDFEKFDQMKAEWGADTVRTTVDLFIRNENDNSPEFQDEPYSAEVSEAAEIGDSVTNVTAIDGDTGDTVSYELFSGPDCEVDHFRVGQTSGEIFVNGILDYASVVQFVVCVIAMDDEGRNDTTKVVISIRDAQNQPPVFIGQPYDKSIPEDTAVGEVVLTVVANDGDLGVNLRNVIKYMLIDDADGTFSVHITNGDIKVEKALDRDTDPITQLYTLIVQAIEEDDDDQPMEQINTNTSIYIRLSDVDDNPPTFTPKGVVNATIPEHSPRGTQLDFSLEAFDLDSGEAGRIILSLEDDEGIFYLVSETIFNSGVVDVRVDNSSALDFERISEVTFQIKASDAQGGNFSVVTATVYLTNINDNTPVFSQSQYDVSLPENSVENTPVQQLEATDADVGAELTFELLGYNNDWFNINSSTGYISVSDTADFNAELLSVYFLTCVVSDGERVSTALLNVTVTDVNDNPPLFQLDLYSVSIPEYNVDMLEEEDEGLNILQVLATDADINVINRMLTYSIESGNEEGKFDINSTTGMIFRKASIDRETEEATFEMVVMAIDGGTPSMNGTTLVTISIEDVNDNNPIFLAALYNAEVPENAAFGTLVANITAVDMDVDSLDVLYTIEEASPFLVNQEGLITLSGTVDYEIQMTYNLTVTATDREDTSRQGSCTVMINIMDVNDEPPEFDTPSPSSLSVNESAAYCDSLPGSDDCHVLITLHATDPDTNTDLTYSILEVKGYDENSRLVEGNFSDQFGVDSETGDVFVRTELDRETVQRFDVTVVVDDMKAEENNDTQTDSAELVITVLDVNDNAPEFIDLPDNFTTPETDQVGMVISVNILADDEDAEDNGIVIYELLDDAGGLVAISETTGILTVKQPIDKELNDSLTITFCAKDGGDPPLNTTVTRQIFITDVNDNTPVFNVSLPNEASILEDTTNGTLVLTVYATDADPDFGDLTYTISAGVGLEDFVINPENGDITVQNELDREEQALYTLTVVASDGIRQDNLEVTITILDVNDNHPEFTESFPESTTISETAEPGSSVAAIVAVDDDEPGTNNSAVRYIMTDENQSGFFKMDYVTGVVILNTTSTDALIPGVYVLVVVASDLGDPPLMTDPRNFSVIVTDYNDEAPVFVQPKQGATVYVDENYSGHITTAEATDGDLGDNGVVTFAFDTGLGATDDYKYFTIDETTGAIEITSETDRETKEIYILGIIGMNAKSDPIRSSQVTFYVQVNDTDDNEPAYWGERTGPEGPQDPPEAALLYVQENLGEGTFVGTVPEAVDLDSDLYNQIYYFIVSATSEDYFILDKYSGNLTLANPIDREKRPEFSLVIRADSYPDYEPDMNVIYDPLKEDNLARVTVTVSDVNDSPPRFEEDRYTAGVLYSTSFGVEVITVNAVDPDLGENAVTVYTILSQKSYDADGNGGVEVDVFAINDGKVINSVTFQASDDSYYIVELQATDSLNPNFTDNATLSVYLMTEIEQVSALFADEPVNVRETQDDFIRLLEEIMENVGGYDAVSVNIDSIDYQLDADDLGIPNRTEMLLHVVDDDTNTVISPSIVQDIIDHAYGYTEELLSYHLYEVEFKTKQVEADVLSALQTASFSLAFLMFLLLVFILVAFYWIVAGYRRRIRALEAFEKVEKKEAPAPGTNKFAENKNPIYNLNAADVKEPLYIDVDEAGNEAVESPGPKEEEFQEVELDFDAELNNETNGEGNDKLLAAVLDTYEKNTGVNLGFDPGESNQNIYMSDV

>LvCDH88C

MSCLFTILLLASSLSFGQGAIEFDEDLANEQISEDLEIGAYVGTIKARDTNDEDAVFVYGIYDLSSSPDAYDYLEVDGASGNVTIKADIDYESFTSMSAQWLANAIKSPVVTLYITNENDNAPIFQDEPYSKEISENEPVNSTVATVTATDADPRDTIIYSLSNSGANCEMEDFRVESTSGEIIVAKKLDYASVVQYTVCVKAADQGGLEDTTTVVISIRDAQNQPPVFIGQPYDKTIPENTAVGHVVLTVVANDGDLGVNPRNTINYTLIDDADGTFSVRTNGEIVVEKELDRETEPITQLYTLIVQAIEDDNSDQSFAQINTNTSIYIRLSDVNDNIPTFNQDGPIGTTIPERSPPGTQLDFGLEAFDLDSGDSGKIALSLEDDEDVFYLASDTIFNSGVVDIRVDNSSALDFERISNFTFQIRASDSETLNSSVITAVVYLTNINDNTPTFSTDQYDVALPENSPEGTFVIQLKATDADIDSVLEYELLGYNNEWFAIDRSYGNITVSADAEFDAELLSVYFLTCVVSDGDRVSTALLNVTVTDMNDEAPDFQSEDYSVTIPEYNLDDLEQQELQILQVVATDADSNEVNRILTYSIESGNEDGKFDIDSASGMIFRNASIDRESEAATYELLVMATDSGSPPLNGTTDVIITIEDVNDNDPLFLEDVYNATVAENAASGTEVANVTAVDADVDSLAVLYIIEGASPFLVNQEGIITLGGTVDYEVQMMYNLTVTATDREDTSRQDSCQVLIAIVDVNDERPVFDTPSPSSLSVNESDTFCDSPTGSDNCTVLITLNASDPDTNADLSFSISTVKGYDENNGMVEDDFSSQFGVVSGTGEVFVRTKLDRETVQRFDVTVGVKDLNATGDDADYQTDSAELVITVLDVNDNPPKFIELPDNFTTPETDQVGMVISVDILANDIDAGNNGMVMYGLLDDADGRVRIDPDTGILSVNSPIDREMSDSLTIIFYVKDKGDPPLNTTKTRQIFITDVNDETPVFNETLPSEANVPEDVANETWVLTVHATDKDSDFGELTYSISAGVGAEDFEIDNKTGEITVQKELDRERQSVYTLTVVASDGTRQDNLEVTINILDVNDNSPVFTDSFPESTSISETAEPGSSVAAIIATDDDEPGTNNSAVRYSMINHNQTDFFKINYETGVVTLNTSTTDSLIPGDYVLEVTAFDLGDPRLESDPKDFWVEVTDYNDEAPVFVNPTQGSIVYVNEGYSGYITTAEATDGDLGDNGVVTFAFDTSLGATKDYIYFTINNITGEINIVETTDRETKETYILGIIGMNARSDPIRTSQVTFSVQVNDANDNEPAYWGKGTGPNGPQEPPEPLQLEVQENLEDGTFVGTVPTAIDLDSDPSFTQIFYFIVEGTNEDYFYLDEVSGNLTLLRPIDREVRPQFSLVIKAVSDPEFVPQENIPYNPLSDGTLARVTVLVTDVNDSPPRFDADRYTAGVLFSTPFGVEIITVNATDPDVGSNAVVEYEILSQTSYGFDTDGNLKTSPVNVFGINSFGQVINSVIFQDSVDSYYIVELQATDSQNGDFKDNATLSVYLMTETDQVSAIFADEPGNIRDRQNEFKQLLEEIMEEVGGYDDVSVNIDSLDFELDGNSLPIPNRTEMLLHVVDDIENIVISPATVQEVIDDAYGYTEELLSFHLIEVEFKTKQVEPDVLTALQTASFALAFVLFLILVLLSVVFYLSVTSYKRQIRAATIDLYRAGEKKEPPAPGTNMFAESKNPIYNPMATEVKEPLSDEDEAGKEAKESLQPKEEEYQEVELSFDVEPHYEDVGEGNDKLLDDVLDKYEKQAHVNLGFDQGESNSQNLYISDV

>ApCDH88C

MTRMWWAQFFVLVQLTQLVTTRPPEFRCQDDFDGIGPNCDGTYEYRDMNKFQIEETTEVNKLLYTLEAFDPDGTAVSYDIVSYTADTNPYEEDGFGYINVDRTTGNVSTKAEIDYEAVHLLWIRWKLTDSTSEETLKSVSVFVKDVNDNDPKFTSERYQLQEAVKEGSMTSNDVIFTVSASDDDSEETIRYDIIEDHQDWPCNGDLFEIDAISGNITLKVNTSLDFEINTQYTICVRASDSGKPSGEPRYDYAQVIVYVQDEQDTAPFFTITNYDREITEDEPLGFGVIRVTARDGDRGVFTPNVIEYSLVGGDDKFEINNGTGQITIKAMLDRETKSSHIVTVTAQEIRADGTPEVQPSSAEVNITFQVGDIDDENATFSSRAERTSLPENSLQGSFVPLNELSVSDTDQAPNNEFKLLLGGADKDTFRIQNENVRGEATVDVRVKNNTLLDFERRQYLDFEIYATNGDGVVYDVARVLVNITDQNDETPTFSQQQYSFEVQENSRGVFVGDVNATDADSEPFSQVTYQLFGLGDNPQFAMDRMTGEITTRPDSSLDFEGQKVYFFTCVATDGERSGTATVQIELTDQNDNAPAFSSETYTAQVDENTIPESSLLSVSATDRDRDNQVSFSLFNDSYSERFEISSAGELKLLQKLDFEEDGPTIKLIVVATDSGSPPQSANATVIVRIQDMNDQSPIFTEKVYDGNVSEDATRGTLVLKVSATDDDQENTVNSRIDYFFVDSVSDFRLSIATGEVTVNSDNLDRDIQGNYSLEVIAIDRGEPARNGTCYVNITLTDVNNKPPIFKDQAPTAEIYENATVNSPVTIVTAVDPDKTAYLSYTIISVAALDEDGNDVSGSFGENFWIDNKTGEVFTGDTLNREVVATFDIKITVEDLNGQQGTDQENQVTLIVNILDINDNAPEFSETNYQFTVTENTAVDTVISTSIGATDKDQGDNAVIRFRLADDVDSLMKIDSTSGTLSVNGTFDREKYPNYTALVVAFDKGYPELSSDATIVIIINDINDNDPEFNTSMNYEAEIPENFKNQTTILQAIATDADGSEKFGSVTYEISAGNPDDIFCVDEFEGDVMICYDKELDREEEEIYRLTVSASDGERIANKQVTIKVLDINDNPPEFTSQPGKLFLQESVDVGQLVTTLTATDVDEPNTNNSKVVYDISAGNSRGLFEITTERGGGVIRTAASLSNEVGVYNLHVVAMDAGMPPLKSEPLFLAIEVTDVNDDTPEIIFPRDLDVFYILENYTGFVLNVTAKDTDSGPNGDVFFRFIDSVGENGLDSKYFDMIQVDNYTVAINIHTVTDRETKEIYELKVEVKDRGPFPLASQISFTVQVNDTDDNEPLFWTDPSNKPAAEFSVLENDGSAVIGSVDFAADADEPQNWHIYYFIVDGNSEEQAKESFKLDKTSGELSLQKPLDREQNQTHMLVVKVTSDEDFIPVAPIPYNEDDNTLLPVTVTVKDENDNGPAFYVEDKLYTGGVKYNAEFESEVIKVEAIDSDVGEYAIVVYKITEQKYTSTDGVVREDNAFAIIERTGSVITNRVFEKGDAGYFTIRVEASDSKNDAFKDEATVSIYIYSEEQQVEMTIFKSTDEVRGYEERFKDVIEEIILKYGVLNSNPSVKRATSAITPAVVIDDIQILVVDDAPKPGWSIMRFHVTNKADNTIIAPSVIVKAIDDAYDYTEIIRTEFGVEDVVIAIPPTEPPPDLTWIQWLLVGILCAVVVFAVGLVATICIMRSGYRRKLRAATAGTYERIIPSSNANHAPNTNQFTEPGSNPLFNKKLSDEFENRYVETPEDAEEAAEDKKKEKMSNGHAKQIADDSISNSEDQEIVVDMFDDSADYEEVPDNETGDKLLMEVLADYDKEKKGQVNLAGFAELTITEI

>PmCDH88C

MAAVWLMQLSLVLLVSKFATGTPPSFLCEDENADYGNNCDMIGNYHDMNGFDIAETNEVNKLLYTLKAVDPDDENSPVTFEILPYTGTLLPDQANGFDYLNVGSSTGEVTTKTELDYETVHRLYFKWKLTDQDGEMFTTTVFTVGITDINDNDPEFTSSSYRLEAIQEGSKTPDDILVTANATDRDTGDDIKYDIVDSHLGWPCNGDIFKIDPSTGGISLKAGETLDFETSTLYTVCVRANDSGAPVGEPRYAYAQVIISIQDVQDEPPYFTKTNYDQEIPEDTPEGSDVLEVLAREGDRGVLVPNLILYSLTGGDGKFEIGNSTGQITIKELLDRETKTSYIVTVVAQEIHADGTLQAPPSSAEVNITIQVGDVDDKNATFTANVVDISLSENTLEGSFVSLDGLAVQDVDQAPNNLFYLHLGGPDADAFVIPNEDEAIRGDATVDVRVSNEKKLDFEANKFLEFEIYATDALGNQYDVAYVHVNITDQNDETPTFDRSEYSFTVEENSRGTVVGFVNATDRDSEAYSQVTYQLFGQGDNPQFEMNSTTGEIRTRNDSNLNYERQSVYFFTCVATDGQRSGTAIVQIELLDQNDNAPQFSATSYTTQVDENTIPSGPLLTVSASDADPSTDNNEITYTLWNDTYSDRFTIKTTDAGGVIRLQQTLDFEEDGSEIEIIVIATDTGEPQLSANATVKVRIQDMNDQSPIFTEEVYYGEVSEGAPRGELVLRVNATDADQENNVNSRIDYFFSDQTVSDFRIVIGTGEVRVNSGNLDRDEKGSYYMEVIAIDRGEPTNSGTCYVNITVLDINNKAPYFPEEDPTVGIYENATSGSLVAMVTATDLDETALLSYGVDLDNSAVYDEDGEEVPTTVDQFFWVENTTGDVYTTDTPLDRETTARFEITITVEDLAAFGSTPQTAEVLLTINVQDINDNAPVFSESGYEFNVDENTDVGSIISNVISATDKDQGKNGEIRYELADNVDSLLRIDPDTGILFVNGTFDREKYPNYTAIVVAFDKGSPELSSNETIVITITDVNDNGPKFESQDYTAEISEDFPNGTTILQVVANDKDESMQFGTVEYQIAAGNPDGIFTIGETDGIIMIEDGEKLDRETEDTHILTITASDGEKQDSVEVTITVLDVNDEVPKFATHSDTINLQESVEPGNLVIALSAVDKDQLDTNNSRVQYFIDGGNEEGLFKIDSLEGDILTQKELSNRVGAYRLRVVARDMGTPPLQSETLITVIVTDVNDDTPEIISPINLEVFYIPENYTGFVLEVEAKDTDIGPNGDVTFRFIQSAGETGKDDQYFDMVSAGNDTATISIHTKTDRETKDTYYLTVEVKDNGIAPLAATVSFTVQVNDTDDNEPIFWTDSENKPKADFTVSENDDSAVIGSVDLASDADEPQNQHIYYFIVDGNAEDQANGAFVLDNTTGVLSLQKPLDREQNETLQLVVKVTSDMDFSPAAPIPYDDKDNTLLEVTVTVKDTNDNGPVFYIKDKLYTGGVKDDAVFNSEVIKVEAVDKDLGDFAVVVYTITSQKYTSTDGKERDDNAFGIIKDTGSVITDKVFQKGDAGFYTIIVKAEDSLNSDFSDSATVSIYLYSEDQQIEMTIFKSAEEVRGYQDDFKQTIEEIILKYGDFSSSQSVKRASTTFTPVIVIDDIQIRVVNGQPQPGWSVMQLHAVNTEDNTIIAPQLVVNAIDDAYDYTEIIRGVYGVDEIVIAIPPTEAPPDLTWIQWLLVGILCAIVVIAFGLVATICIMTSRYKRKLRAATAGIYEPINPAANANHAPNTNKFTEPGSNPLYNSKLSDEYEKRYSQSPESAEAAEEDTKNEKISNGHAKKIPPDSKSVEEQEMVVDMFDDSQDYQEVGDNETGDLLLLQVLADYDKDREDGLVNLPEFANMTITEI

>ArCDH88C

MSNMQGLQVLCLLVGIQMVAPAPPMFQCKYEQRPGECRADAEINMDNEQIEETDAGTYLYTLEAWDADNDTVTFEILPYYTDDLPEFEANGIHYLQVNETSGVVTSKAVIDYEKVHTLRFGWNLTDSGGEGFVQKSIAVTVKNINDNDPVFTSKRYQVDPITENSLKPNEGIATVIANDTDSEEGILYGIVQAYSDWPCNGDIFKIDPTTGVISLNESESLDFEKVSLYTICVNATDSGKPTGSGPANFGYAEVILAILDAQDTAPVFKSSNYDGEISEDTPVGSVIATVAATDGDRGVLVANKISYSLVGGDGKFGMNNNSGKISILEGLDRETKDSYTVTVTAQELNENDTNEAFPSSTTVNITFQVGDADDENATFSNHSREKTLFENSLTGTFVEIVGLTAKDKDLSPNNVFYFALGGRDRDAFDIRSRKITGEGGVDLRVWNNTDKLDYEKYKFLQFEIYAQDISGDIVYDTVYVNITLLDQNDETPTFSQDAYVFSIPENSKDEFVNITEATDKDSDEFNQITYELLGQGSDPQFVINKTTGEIYSKVDAALDYEIQKVYFLTCVASDSQRRGTATVQIELMDENDNPPEFNPTEYTVNVEENQIPDEFLVQVTARDIDDAIENKEIFFSLLNTFLSEKFRIDKITATTAIISLDDILDFEEDGPLFNLTVIAANRGNVSKMTTANVNINIQDMNDHSPVFVEDVFVGEIPEDAKRGDFVLQVKAEDMDRVGSANARIDYFFRETDVSVSAFRIEISTGRITVNDQLDRDLEASYELEVEALDRGDPYQPPGNCSVIITILDVNNKKPTFSEETPVEEINEGVVNGTLITMVTASDSDETAELVYSFDTISGFNKDDKIEQNFSGWFSINQTGGVFTVIELDREKVTRFVIPVVVKDVNATEEQMAIATLSLTILDVNDNPPAFSDVDYEFDVTENDIEGTIISAIISATDKDEGSNGEIIFDLVDDKNLMKIDPETGILQVNGMFNREEMATYHALIVATDKGDPVQNTTKNITISIIDVNEFPPKFKLSNYSVALLENFTAGMEILRVSATDQDAGDIFGHVTFGIASGNADDIFTINGTTGVIMIKEGAKLDRETEEEHILTINAIDGGGRSENEAVEIIVLDVNDNAPVFNKFEPRSIQETVPEGDIVFTVTATDADQPGTPSSQLEYSIEGGSDKAEELFNITTSDNTGNIMVKSSLANRVGIYSLLVVVRDGGSPSNNANATVQITVTDVNDDPPEITFPTDLSTIYVEENSTGFVLNLTALDSDVGINGEIYFKFVQSSDGQEFFELDSKSNDDGVCMVSFKDGHVTDRETKAEYELTIEAYNKGDGNNKFATRVTFIIKVNDTDDNEPIFWEDESDQSIEFSVFENNATAVIGLVSEATDADEPSNQLIFYFIVNGNTEDCFTLNKESRQLSLKKSLDREKNDTYENIVIKVTSDFNYDDSIIVPYDPTDNTLLQVTVKVLDVNDNGPIFTSKSYTGGVEYNTGFDSEVIVVKAIDADFESNAIVNYFKTSEKKTDPDGTDYPEIIPTFKVDNLTGSIRTLRNFESRDTGYFTLEIVAYDSLNSQYRDTATVSIYLFNEDQQVIFTFALSTDNVRKIEEDFKATLVEVIEKYGDFGSSAKRAAKTVEPVIVVDDLQVFTVDNQPVPGRSVMKLHGVNKEDNTIIDPVIIVNSIDVAYDYVDEFYEKYKVFEVLGPDSVPPEPSDWVQWLLVGILSFFVVLAIGLVVTNCIVTSSYKRKLRAATAGLYETKSKAKGAYSLPNTNKYAEAGSNPVYTRTLNEGYEVAPLSNQDAPEYEVVSDQENKKQPIGNGYSHQQVSSDPLSEEQEVTMEMYDDSADHEHVEDDELGDQLLLEVLKDYDQRKTTGSNSLTGYANFPVTEI

>BfCDH88C

MLWRQAVVLGMVCIILRTGAQANQPPIFTQGMDYQQIPEDTAVGSTVYTLSATDADGDTLTYGVSGQVSNNLFIVDPSSGVVTLKTPLDRELTDEYQITVTVSDGTNPQVVQTPTIFITDANDNSPVFQGIPYEVSVNESDVSQTSIFRVTATDADQGIGGTVSYFLEAGDESKFEVDRPTGVVNLKEELDYEESSVYQLRIRAQDGGGSYQGTQVFQSSTTVLIVNVVDQDDQPPLFLGQPFSTQVNEDTPLGTSIITINARDGDYGIDNPIVYSTEGDDGTFSIDSITGVISLARTLDRESKTDEGGVYSFDVQARENSIEARTTNTSVRITVVDVNDQIPTFYNGTGNSPQNYFTATIPEGTSAGIPLGGLDMRVEDNDEGDNGRFTLTLDEEGSRYFEVVPSTVYGQAAVNIRVKNSRLLDYETLQSVQFKVIAREDLAAEHYNSTATVLVTLEDTNDNSPVFNQSKYDLSVAENSPDGTVVGTITATDLDSGEFAEIAYSLQGSGSEKFAVNNLTGVITVAKGDELDRETIPFYFLTLLAEDGGGRASNVQLQITLLDVNDEAPTFQRSSNKVYIQEDAAAFDEPLQVLAVDRDEGTNKVIKYSIISGNDHDNFTIDEDSGVIDLTQPLDYEALGGGDEFHLVVQAKDEGQPPLNSTTTVIVVVEDINDNDPFFLEDMVNTTVSEAAAADTLVVKLNATDEDSGFNGRIDYRIASGSKDKFYITNDGEIKVSPGGRVPLDRDLYGDGYTLIVTATDRGNPDQKTGTATVFITIEDINNKDPVFVSSEKTDTLPEYPNTTYPSKLADMPAVDSDIDSSLEYTITSIMAQDENGQVVTVGSNLFNITSKGEVWVNGELDREFAEDYVLRLEVNDTAAPWPGEQTATATLTIILQDVNDNAPKFSSDEIEQNIQEQLPIGQLLATITANDPDKGPNGEVMFSLTGTNLLAIDSSTGQIKVNSTIDREYGQWLNFTVTAKDGGNPPKSTDKSYNWRVSDTNDNDPKFEQPSYSGEVSENATVGTSVLTVTATDADFGQFGAVRYSLVPVDSNFTIHPDRGEISVDGKLDREMEEQVTLTVTATDNPGGSENNQREKSVEVTIKLLDVNDEAPTFSQNIYTEYIQEDEVAGTSIITLQAVDRDSAGPNSQIEYTILAGNEAGVFTVGRDSGIMTTLKPPGLAPGQDINVTVLASDKGSPIRSSTTIVRVKILDTNDNAPIFVYPAANASTTVLENEPVGTFVVRVLATDEDEAQNGNVTYYFDPSDEYNGDRANFTLDPISGNLTTKVVLDREEKAQYVLYIQARDAGFPPLTGATVIRVHVGDVPDTDPVFKPQLDPVTGEVVPQTLTVGENLVPGTVVGFVLNAFDADEGATIFYYIVGGDPNGYFDLNKNTGEIKTTRTFDREAEASYTLVVKASNNASYSVPGGSRRRKRDIAYDPADPTLQEVIIEIGDQNDEPPRFTKKEYTAGITTEFKFGDAVTVVTAIDPDAGNNSVVTYTITRQLYFDRDSEGNLMSTPKDATGTFNIESDTGQIVTGKVFSSDEKGYYTLNVSARDIGGQSDNASVEIYLLREDQRVKIVFQRTPDEVRAFKDEFAALLSNITGAIINVDDIQFHTTDDQQTDELRTDMFIHGVDRTTGLIMDKERLIELIDANYEFLVRLLRDYNVVEYQVAVPQVQDVTYDNLIIAIAAMAGILVISIALIATVCVCWTSRLKRELRASQAMLYSTKDVIPDLSEKDALQVPGTNQFAIDGSNPMWGKEVYVNEIAEESDSDDSLDDNEVDVKSPQAPSAPPRDDYADEEITVNLYNDEYDNYTKFPPGYANGNFILDAALKEHEDAKRNGPHRNFSLETTEI

>CgCDH88C

MESALFKIIWVIFGFLCSGLNATNNPPSLLNFNRYIFVREDFPINGSLGILSARDLDGPMEIKFTIKDEVTRSLVRLSEHFGESTVNRSVEIILKKQLDRDHEPSDRKLYFNLADGEGSSANNLAVQVTLFISDVNDEVPEFVNLRYKESIYENATVGTTVIRVSAQDPDNGLGGTVSYYMEPVAQAADELYKNAFRINSDTGDVIINSSLDFERHNFYEFVIKAKDGFGNESINNADFVVTVLDVQDTPPAFFNLPYSVVVGEDKAVGERVLQVTALDGDRGVPNGIRYTFVQGGYQNFNVDPNTGWITIKTELDRDDASIQDSGGVYAMYVKAEEVMSGVNYGNTTATTLVTISVSDVNDNTPTFNHLNYTATIQENMQSGVPVIFTANTIMSVNDIDQGLNSYFEIVLEKDGQPYYDFAPLPKEVFSESSILIRVNNSVDLDYEKSKTATFDVIARELRTNPKRSSSVKMMVHILDVNDNQPQFENTSYSAQIAENSPTGTTFTRLQATDLDSGVFGKITYSVRGGNGRFGINDSTGEIYNTEALDREKISEYYITVEAKDGGGFRTTVELNVKVTDTNDNKPLFTREAYFASLKEGATSFVRELKLEATDLDEGMNSQVLYSISNIQPPVTNTFTIDDATGVVGLSRPVDYEQLTSSGQIMLGVQVCDRGLPSLCSVINATVEVEDENDNKPVFNQTTYQVNIYENASIGSLAVKVDAYDLDGTAPNNEFVFRIESTTQEKFRVNFRTGEVLVESELDHEQSSFYSLNISATDRGSVSLEGYCIVNINVLDVNDEIPYFDPRSQSLAILENKPVGSQVVTLTAVDTDSNPRLRYQILQDSILATDEEGRDVNVTANNIQNYFDINETTGILFVKSVLDRETAEKIELKVLVKDLNGWQPSADQQTATATLTVTLLDVNDEPPEFLPGPQYHVNVSEGREVNDLVITVAAVDKDKKQTVFYRIGQDDINSFQIMDSRVGTVTLKKQLDYESNHEVSFTVIATDSASPPSSVLTSTATVSVSLIDINDNTPQFLPHPTQYSVEENAPNGTFVGNITATDRDSGRFGEITYSLQVTNDDQSLTIDSKTGIISVLKPLDREMREEYILYVTATDNNDANPNQQRSERTGAIFVKVTDVNDNHPIITNIGTTPKSVVENSQNNTIVDTILANDADIGANAEVEFSLVSDDTNTSDSWFYITTQYNSDIKNNEGVIRTRQSLLGHVGMYYVTVKVQDKGTPTRLSSNMTLLIEIIESNENLNQPQFVVPKGPTATLQIKEQQPVGTTVIQVSATDADHGKNAEVHYYLAPEKDYTKFQIHKDTGLLTTRAVLDREQQAQFEIRVIANDSGIISVLDNSITIFVQLLDIDDQEPEFPRRPDMEPYILGPVPEEHSMEYCGTVDVAIDRDSHVNNSLIFYYIIGGDMVDHFYLNQTGELYLIKAVDRDGDVTGTPINFINLVIWATPLGYLTFDKYSTVPGTQRMVSQKIEMRPPDDYDSSNTTLLWAQVTIKDINDHPPKFRNQNLSVGITRKTQFQETIFTLSDEVTDLDSGNNSVHTFHLMSLAIYPEALRNKLPSAPLSLSSDGIIRTNTIFQSDMSGYILMDVSAQDIDGKMANASLRISLINDDQRVKIIFRMTPMQVRNFSETFRSKLERITGYHIVVDKIQTHENAQGKPEADKTDMFIHGEVIKPFRIVSAAELLSKIDGEAAALVPILNEYNILQIVSAVQQTEEEDSNRLLIMGLILLAIVLGIPCIVMAIVIFLITKSYQRKLKAATAMVYGQENDMQKNQLPGTNLHAYENANPIFLEKVLSQESQDYDDTDSVDNNVVDSPVPQTVDTQEVSMTFTADSSKDYTHNSSLKTISSVIGSQENSFGHRNPLFSNVNGDTPRSHLINGLQASEI

>DmCDH88C

MDYKKVRLKGQRRRSFSSYISSISIQVIILTTLISLVASNRPPRFAIDGQSEIVLRLKESPETKVGTLIYTLKGYDPDNDPLTFGKRNSHDSEIIRIENTGGNEAKIFLAKELDRELQDEYAIVLTLTDSHYSDHNYVTQSFLLLVEDINDNVPTFLPYQNAIEIPEGSAPGVVSTLEATDADEGAYGQVVYYLQELDGDNDVFSIATHQGKGILRLQKELDYERKSLYQLRVLAIDRANQGPINTGTAAILVKVKDLEDQPPEFVEVQAVARIAEDAPVGTKVLRVRAIDGDRGINNPIAYSLEANDLFDINPHTGIVHTLTKLDREEQSDQVNGAHILRISATELSKSNTQMAPTTVRTEVTVIVSDVNDEIPTFGETVYRCEVNENAQTNTPLNFIDEEVQNVVFDHDEGNNGTFRLFLDPPNDLFEIVPELAVNEANFMLRVKNSKSLDFEQFTEVNFTIFAREVDEPSRWSSAHVQIFIRDQNDNFPEFSQTIYNASVLENSEQDTIITHVQAVDVDSGDYGTMGIRYTNLRGGIAHLLNLNPITGVITIKQAGGTAFDREIISRHYLTVEAIDNAGQGNRNTAQIIVDILDVNDNAPTFPQRQYETKLLENQAEFETPLQLEARDADLNGTENSQVTYEIVEGLYRSNFTIDPQSGLLRPVHSFDFEELVDGSSRRSDPYTGGSFSIREIDLLVRARDSGIPMLSTVVPVLIYVQDVNDNAPIFQQSFYAKTVPEDLPGGSSVLQVTAIDRDGSAPNNVVVYRIQTGAGDKFIINSETGVISVAHGANLDPDLTESKRSLYTLSVIALDGGLGNSQLMTTCTVNISIQDVNNKPPVLKEMPALQILENTPVGTLVYRIQATDLDHKAILRYKLNPEHCEGRTEEGALVKSSEYDFLGAFEVDSIEGTLKVVKLLDRERVEHIKLAITVEDLAAAKGRQIAEGFLSIQVLDENDNNPKFRLPFYRQSITENSINGAMIVNVLASDVDKNRTITYALEGNPTYRSLMHLDPQTGEIVVASKIDHEQHQWLNFSVRATDSGIPARSSLVDVYITVLDENDNNPYFVGGSKNYTISENAAPGTRVATLQAGDADSGDFGKITFLMDRISSQGKFTIDADTGVLTVADRLDRESKDSYNLVIEAWDNYQFGFLAGESRNAFKQVFISILDENDNPPEVDLPMSCVLITEYHELHERVASIVGKDADDPTTPNGRLDFAITRGNKDGMFELRQIDAWNAQIFASKSLRNRYGNYSLTITTRDMGLPANIVHNTLDICVSDFNDHAPVFVRPLHNTTVRIPENATVGTLILQAYASDGDMGQNALVRYRLKPDPLGSYKMFEVDGSTGELFLKEQLNREKQKIHEIRIEAYDQGLPTSLSSDLDLTIYVRNVNDYEPQFMVNEISVNFTEHSDPGSERIKLPDTIDKDQLELDDPNDTPSQVCYFIVNGNEAGYFRLDPETHILTVDRELDREVIANFTLYVRATENCRNDSISGGEKRRRMGIEINNRLGGIVHNQQFGYDRFKHSRQLRSTESNEYGPEDAELLSYEAYDSSQEDPTGGLATTHPELDSTVIKVKVRVLDINDNPPRFRSKIFTGGITTNADFGLKFMRVEATDADEGDNGRIGYYQVGEIRQTLSEGLENVRKAPFLLDQETGEVQLNFDPQKGMKGYFDFVVLANDTSGMKDVAHVFIYLLREDQKVRFVFRLQPDELRSRVDSFRDTLSNITESIVNIDEIKVHENKDGSVDKTKTDLYMHLVEREDNSIYEVSEVLKLIDSHIESLNGLFKELNVLDTQAAEAQLLTAGPSRGPLFVWLIFTNLFLATLLVVTIALCASQRNGYRRQLRAAKVNIFRGHSSMSLQDAQEPATRVPNTNKHSVQGSNPIWLKGYDNEWFKSEESGSIGGHDSLDDNFLAVATQDMHETLKGTAKLFNNSNDLNRHFNLYNQIDKMTNNAQILARKLETTEL

>SpCSTN1

MDNDSFTDVSSITTDNEDDNDTFTTVTDLTYYTHDLFNDDSSDVILDISLTKDEYELINDTSNHAPYWEDEIFRGEINEHGRLEMLPPALVAHDHDEGANGKICHFNILESVPFTASVQTGATGGAGTIEAVSGAINAAVKDCDENREWKFHIQASDCGTPSLPSPTAQVHIVMRDPNNNKPRFLQNSYEATVEEGTVPHKFLQIQAVDDDCSPGFSTICSYDIMTPGMPFKIDIKGKRTLFSFPERSLRFDFKLQDIFSKMASHMSLLASNNCPLDLSRISAVSPLTSDGGPTYSLQIKSCDCSGLCSETIPINIHVSQQCQPGWKGVPKRIEYLPGSKATALAPKAFLDTCGRLVCNSSSLTTTVTLQTDHIGFGCDRETYSTQSQRTLCNADAGTYDLLPRPNAGEQWTKKLTSDNGNESDQIYQFDGVEDAVIVPEQFAPPNNLTSTFSISFWMKHRNIVGGDPEYIVCKSDSKEMNRHHYGLYVHNCRLVFVSRQETGDGFYPSMYRWKLRQVCDQEWHRYTINVEELQASLFIDGEAQIGPRVSEDWPLHHTTTPTSTTVGACFKGAQDKFANYFQGYLAGLSVLPGQIETPEVVSCMVRCKEGLSFNVGNLPVSVARINTARTNLTLSGELTEQFSSVLQQVGYINERTFPTQGRRPLTISTKASCNGVEIVIPDVSSYVMVLHPHEPTISITGSQATSQTVANLVAGIAPFKTITIVSIMTEQEEEELGADQQEVTGGDTPALITHNLDSCSALVKQGRLVDGETLTIAEDILTQHGLTVVNSENGIVIKGLATLKDYQNVLRQISYSQVASGKNQDRRLTLHCSELNGRFVSNDFDVRIGVLHQAMRALNKPSHVKQSVMFSHSNVRVHDKLKENSPKHITSKNAVSSTAMAIVVVLCVGFLLFMIVLGVFRIYSAHKANRAFDGQDMDWDDSALNITVNPMEGPIATGETDESSEDDDEDSLEDESSSSEEEEEEVCVEIKGTPLEWDDSTMKF

>LvCSTN1

MLDPLVAHDLDEGADGKICHFNIGDSVPFTASVQNGAKGTGSVEAVSSAINAAVKDCDETREWRFHIRASDCGTPPLSSPTAQVHIVMRDPNNNKPRFLQNSYEATVEEGTVPHKFLQIQAVDDDCSPGFSTICSYDVMTPGMPFKIDIKGKNLTFKKNLSSFDLFLLFCYLNGCLLRLVMVNSVFLMSATSPLTSEGGSTYSLQVQSCDCSGLCSDTIPVNIHVSQQCQPGWKGVPKRIEYLPGSKATPLAPKAYLDTCGRLLCNSSSLTTTVTLQTDHIGFGCDRETYSTQSQRTLCNADAGTYDLLPRPNAGEQWTKKLTSDNGNESDQIYQFDGVRDAVIVPDQFAPPDNLTSTFSISFWMKHRNIVGGDPEYIVCKSDSKGLSRHHYGLYVHNCRLVFVSRQETGDGFYPSMYRWKLKQVCDQEWHRYTINIEELQASLFIDGEAQIGPRVSEDWPLHHTANPTATTVGACYKGAQDKFANYFQGYLAGLSILPGHIETPEVVTCMVRCKEGLSFNVGNLPVSVARINTARTNLTLSGELTEQFSSVLQQVAYVNERTFPTQGRRPLSISTKASCNGVEITIPDVSSYVMVLHPHEPTISITGSQATSQTIANLVAGIKPFKTVTIVSIMTEQEEEELGAEQQEVTGGNTPELITHNLDSCSVLVKQGQLMDGETLTIPEEVLQQHGLTVVNSDSGMVIKGLATLNDYQNVLRQISYTQSSSGKSQDRRLTLHCSELNGRFVSNDFDVRIGVLHQTMRALNKPSHVKQSVMFSHNNVRVHDQLKEKHSPQHITSSKSAISSTAMAIVVVLCVGFLLFMIVLGVFRIYAAHKANRAFDGQDMDWDDSALNITVNPMEGPIPTGDTDESSEDDDEDSLEDESSSSEEEEEEVCVEIKGTPLEWDDSTMKF

>ApCSTN1

MLRRSRWAGGALLVVLIALSGVNCSEDTTNTKPYFTERIYHGEVNKTSGQVSIHPQLIARDDDAGPNGEIVEYRLDPEADVPFTVSLGGQGQAVLRAVDPQSLDCERSQAGWQFSVQALDGGRPQKTSHRTEVHIVINDSNDSPPHFVKSSYSGMVLEGIAEENVVTVEARDSDCSAAFSTICQYEISTRDGSTVPFAVGFDGRISTTKALDAEIQSRYLFNVVAYDCGGRSSEPVPVTITVSKLCSPGWKGVPRRIEYSPGSGVQPLAPMMHLETCTQPNCTTSEVITTVKLQTRHIGFGCDRETYSADSQRKLCGSAAGSYDLLPSPSLGQKWTEDLLTDEGHDSEQIYEFDGEESAVIIPEKYAPSNLTYRFSISYWMKHGETEGLNKEHILCSSDSEGLNRHHYAVFIHNCRLVLLLRHQDEGKSDAFFPTEFRWKVDEVCDKQWHRYTFNVDFPSVMLYIDGELHQTKMISDDWPLHHSNYPVALTIGACYDGGKGRFSGHFQGYLAGLSVLPGHVETEPTIQCMYSCKEGLTVTPDDLAADRLQFNAMKTELLIKGDLNDASQLLRRVSYFNSRKFPTPGQRALTIKTITTCNGIPMPMTDVSAYVMVVQPPEPTITISGPSEMAQETHEVVAGIRPFASISIVSTMSEEQEEEEEAALEEIGQKPDVPLTVSHRLDSCNITTGTYLTGGEQFTIAAELLASYNLIKFDSSEGVSIKGVDTIEHYVEVLRQVIYSSSDTEQLSHVLTVTCTELNGRFQSNEYKITVNILRKSIKQAIRPNHDKAAPRSNILFKPGKNTLEEEMKEKMPLTKLGQITASSTAMTIVIVICVGFLVFMIVLGVFRIYAAHRDSTYDQQDFDWDDSALNITVNPMEGPTETHVGVDSDSSDDEDDSCQDDLDDSSEEDDDDDDEDDASGEIDVEIKGTQLEWDDSTLKF

>PmCSTN1

MVNISRRKQRVRGAEDNTEMFRRSRWAGGALLVALFALSGVNCLEDTPNTKPYFPEKIYHGEVNKTTGVVTIHPQLVARDDDAGPDGEIAGYRLYPDVDVPFAVTLGEHGEAVLRATDTQELGCEKSQAGWQFSIQALDGGMPQKTSHRTEVHIVVNDSNDSPPRFVQSSYTATVSEGVIEDNVLTVEAKDSDCSAAFSTICQYEITTRDGATVPFAIGFDGRISTTKALDGDTESRYLFNVVAYDCGGRSSEPVPVTITVSTLCSPGWKGVARRIEYTPASGMQPLAPMMHLETCTQPNCTTSEVITTVKLQTRHIGFGCDRETYSSESQRKLCGSPSGSFDLLPSPGMGQKWTEDLLTDEGHDSEQIYEFDGEESAVIIPEKYAPTNLTNRFSVSFWMKHGETEGLNKEHIMCSSDSEGLNRHHYAIFIHNCRLVLLLRHQDEGKSDAFFPTEFRWKLDEVCDKQWHRYTFNIDFPSVMLYIDGELHQTKMISDDWPLHHSNYPVALTIGACYDDFDTDARAGGKGRFSGHFQGYLAGLSVLPGHVETESTIECMYSCKEGLSIAADDLAADRIQFNAMKTELVIKGDLNDASQLLRRVSYFNSRKFPTPGQRALTIKTITICDGIPMPMTDVSAYVMVVQPAEPTITIEGPSQMAQEGHEIEAGIRPFASISIVSTMSEDQEEEEEAALEEIGKKPDVPLTVSHRLDSCNITTGTYLTSDESFTVPAELLASYHLLKFDSSEGMSIKGVDTIEHYVEVLRQVVYSSSDTTEKLSHTLTVTCTELNGRFQSNEYKITVNVLRKSIKQAIRPNHDKAAPHANILFKPGKNTLEEEMKEKMPLTKLGQITASSTAMTIVIVICVGFLVFMIVLGVFRIYAAHRDSAYDQQDFDWDDSALNITVNPMEGPTETHIGMESDSSDDDDDSCQDDLDDSSEDDDEDDDEEEDGSGEIDVEIKGTQLEWDDSTLKF

>ArCSTN1

MLLRRRWACGALVVTLLAVSGVICQEDTTNTKPYFDEKIYHGEVNKTTGLVSLHPHLIARDEDSGANGEIVQYLIDPEADVPFTVSVDSTGEAILKATTLDALDCQKSQSGWQFNIQAMDNGMPQRTSHRTEVHIIVNDSNDSPPHFVKSSYSATVSEGTREENILTIEAKDNDCSAAFSTICQYEITTDDVPFSIDFDDMAYDCGGRFSESVMVTITVNKMCSPGWKGNSRRIEYTPGTESQILAPMIHLETCVAPRCNVSEVITTVKLQTRHIGFGCDRDTYSSQSQRKLCGSAPGSFDLLPTPSLGQKWTEDLLTDEGHDSEQIYEFDGEESAVVIPEKYAPGNLTSQFSIAFWMKHGETEGLNKEHILCNSDSEGLNRHHYAIFIHNCHLIFLVRHQDEGKSDAFFPTEFRWKLDEVCDKQWHRYTFNVDNLSVYLYVDGEVRQTKMISDNWPLHRSNYPTALTVGACFDGGKGRFTGHFQGYLAGLSVLPGRMEEEATIQCLHSCKEGLSMNTEDLPADRVQFNSINTELVIKGDLSDASQLLQRVSYFNSREFPTPGQRPLTIKTIATCDGIPIPMSDTSAYVMVVEPEEPTITISGPTQMAQEADKIASGIKPFASINIVSMMSEEQEEEEEAELEVTGQEPEVPLTVGHRLDSCNITTGTYLTSGETFTISADLLTSYHLTKFDSSEGMSIKGVDTIQHYVEALRQVVYTNSKKSDKKSYTFTVTCTELNGRFLSNEYKILVNVLHKAVKQAASPNHNKAASHNNILFKPGRNTLEEEMNEKTPLTKLGQLTGSSTAMTVVIVICVGFLVFMIVLGVFRIYAAHRDSAYDQQDFDWDDSALNITVNPMEGPVETHIGIESDSSDEDDDDSCQDDLDDSSEDDDDEDDDDEDGAGEIDVEIKGTQLEWDDSTLKF

>AjCSTN1

MASAKALFLFAVFLHLDSIYCQGITTVSTAVHNSYRPYIEDASYHGEIDENTNSVNLHPHIFAKDDDEGVNGQICAFHVLDETVPFRIDVVDKITGQGNLVVLDPERIDCERQSEWKFEIQAGDCGNIARYSHKSTVHIVMADVNDNKPYFSKIAYEVAVDEGELLKNFTQVEALDDDCSAQYSAICEYEIITPDVPFIIDNKGRISSTKPLNPSDVSSHVFSVAAYDCGNKKSDAVTVTVTVNRLCVQGIKGLSKRIEYMPDTDNHILGAKMFLELCAPSKCNSSSVSMVFEMETDHIVMGCDRDTYSVAAQRQVCNAPDEIIDLLPTPNVGEKWTEDLQTDEGHENTQVYKFNGEDSQVSIPDKFLPSSLNTTFSISTWMKHAPEKDIKETILCYSDAEGMNRHHMALYIHNCRLVLLVRRHGDDPDDYRPAEWRWKLSEACDKEWHHYVVNVDLPEVKLYVDGHHYAAHHIVTDRPLHPTAYKSTLTVGAAWEGALRQYIDHFNGYLTGLAILPGKTESKEVISCIFACKERLTINKDTIPMHEITQTKSGNRITIKGPLDENALSYTMQQIGYINERQYPTPGQRPLTITTKATCDGQAVKIRKVESYLMVVRPSEPTIILSGSNHIARMASDLVTGILPLSDISIITQLQEVEEIDEETGEQLDPLTVAQNLDSCSVVLDSLMLSEHESLTISYELLEQFNLKVISSSSGLIVKGIQSIENYEEVLHDIIYTNDRGHDYRIFTVQCTELNGRYVSNEFMVQLTILHKKLTANPLPKQMVPKLVKPRLQGPELPNELLKRKDHMMFKAASNGQEDISQKITSKNYKSTVGGSSSIMTALIVICVGFLAFVVFLGLFRIYMVHRNSGEEKQDMDWDDMSLNITINPMDPENAQEVMEDTDSSEDEDESANNSDESEAEEEEEVLEPVKGSPLEWDDGNLKM

>SkCSTN1

MLFSLRRIYGFAVVLIFLRIGSVLSENEQNKHKPWFDEEFYHGELNENERLVQLHPSLVAFDSDDGAAGQICNYVIKDDGIPFTIEVDKNTGEGFLRATEPADCEKQRDYTFDVQAEDCGETPRLSHKNTEPLSYDMATNYILAVTAYDCGGKKSPSVLVTILVSKICTPGWQGIPKRIEYEPGSGRASAAGSIELLPTPGLGHAWTEDLSSDEGYESDQIYEFDGEMSAVLVPDNIVPANLTDHFTISTWMKHGPNYGEGKETILCSSDKSGLNRHHYSLYIHNCRLVFLLRRDFGNLETFRPAEFRWKLDKVCDKEWHHYVLNVDFPKVTLYVDGDSFEPHHVTEDWPLHKSNFATQLTVGAAWEGADGEFVHHLKGYLAGLTMRPGSTESEHVITCLNGCKESLEFHDFDLLELGEEATFDSTQSELTLKGQLIGDNFQRLMQHISYVNSRQFPTPGKRAITLKTKAICDGVEIPISNIDAYVMVLQPPEPTITVTCSEHHAHKESDFLEGIKLFGDVHIVSTIEDEEDEDEEMATEAVNIAHNLDSCSMQVNPPLNTKTERITLPDEELKKLHLDKYNSTQGVVIRGVDSIANYEDILRKVVYINTDPSQYYERSFKLSCSELNGRYTSNLVEVQVNILHSSFPGPKVNNDKASEHVVSFKHNNIGKQEINKEPGLQNKESASKTGTAMTFVIVSCVGFLVFMIVLGIFRIRAAHRRAQVGDDRQEMAWDDSSLTITVNPMQQTLEYDDQEFQAVGDSDSSDDDDDDDDSSCHDDLDSSEDEGEPSKEHQLEWDDSTLTY

>BfCSTN1

MNSMSSFRHLGEKGREHLIFFTRFKTSRAATADKKRETMIIRWSVLLLALLCGVLAETRQDGRTWEDDVDKIGTKKLDDSTEEDDSDKYWKETKSSEEDDKALSDDDWLRSQLDQQRWEQQASGRAAQRLAQDVWQPLTFDRPPKPLDLFPKQQSNSNNGNKHKPWLEIEDGQIFHGLINENDMSMVELHPKLKASDKDSGDAGKICQFRITQQDVPFDAFVLNPNTGEGYIRARKELNCEDKKEYTFTIQALDCGVPEKGIQQKKSHKATVRIQVNDVNEHGPEFTASGYEATVMEGKMYDNILKVEAEDRDCSPGFSQICSYEIVTPNVPFQISEDGIISNTEPLQYVDGHNYILTVTAYDCGQRRADLDVLVTITVKEQCHPGWKDFQKRIEYEPFSGTKALLPKVYLETCGEEVSDITTTFTLETSHIGKGCDRDTYSSSSLLKLCGASQGSIDLLPAPGLGSEWTQNLPTDEGHEGDQVFSFDGRQTGVEIPEGVVPKNLTNQFTIATWMKHRETFGGDKETILCKSDKKELNRHHYSLYVHNCKLMFLLRHDGGDLETFRPAEFHWKIPQVCDKQWHHYAITVDFPEVNLYVDGIPYSPFLVTDDWPLHPSKFDTRLTLGACWQGGDQEMSQYFRGELAGLLIHPGKTETRRVISCLHNCNESLEFHSFDETGPGSEVHFNPDQTQLFLRGEFTPKNPDNEAYTLDKYTKVLQKVAYQNGRQFPTPGMRALKIHTEVNCFDDETCIEVPDVDAYVMVLHPEEPTINLSGTDHLARRVAELDQGVAPFRDLRIVSTIVRDVEEEEEEELQEGGTTEVTPISHNLDACQITVDPPLESGKEELTVPKEDLARLRLEKSAATNGLVIKGVDTIANYEDILRKVVYRNSKAGDYFDRVLKLKCSEMNGRYESNEYAVELKVLHTLNVANPESHVKVAAAVAQNVPAQFIHSKGVQEAAVPHQVPQVVASSQSANVVTSLVIVICVGFLIFMIVLGVYRIRAANRRNMDSEWDQAGEPRMEWDSSDLTITVNPMDVEYEGAESDSSDDDDDSAHDDSDSSDDDDEPPREVLGYGQELEWDSTAMVA

>MmCSTN1

MLRRPAPALAPAVRLLLAGLLCGGGVWAARVNKHKPWLEPTYHGIVTENDNTVLLDPPLIALDKDSPLRFAGEICGFKIHGQNVPFDAVVVDKSTGEGIIRSKEKLDCELQKDYTFTIQAYDCGKGPDGTGVKKSHKATVHIQVNDVNEYAPVFKEKSYKAAVVEGKQHSSILRVEAVDADCSPQFSQICSYEILTPDVPFTVDKDGYIKNTEKLNYGKEHQYKLTVTAYDCGKKRATEDVLVKISVKPTCSPGWQGWSSRIEYEPGTGALAVFPSIHLETCDEPVASVQATVELETSHIGKGCDRDTYSEKSLHRLCGAAAGTSELLPSPSSSFNWTVGLPTDNGHDSDQVFEFNGTQAVRIPDGVVTLDPKEPFTISVWMRHGPFGRKKETILCSSDKTDMNRHHYSLYVHGCRLVFLLRQDPSEEKKYRPAEFHWKLNQVCDEDWHHFVLNVEVPSVTLYVDGIPHEPFSVTEDYPLHPTKIETQLVVGACWQGGDLHMTQFFRGNLAGLTVRSGKLADKKVIDCLYTCKEGLDLQVPEDANRGVQIQASSSQAVLTLEGDNVGELDKAMQHISYLNSRQFPTPGIRRLKITSTVKCFNEAACIEVPPVEGYVMVLQPEEPKISLSGVHHFARAASEFESAEGISLFPELRIISTITREVEPEADGSEDPTVQESLVSEEIVHDLDTCEVTVEGDELNAEQESLEVDVTRLQQKGIEASHSDLGVVFTGVETMASYEEVLHLLRYRNWHTRSLLDRKFKLICSELNGRYLSNEFKVEVNVIHTANPVEHANHMAAQPQFVHPEHRSFVDLSGHNLANPHPFAVVPSTATVVIVVCVSFLVFMIILGVFRIRAAHQRTMRDQDTGKENEMDWDDSALTITVNPMETYEDQHSSEEEEEEEEEEESEDGEEEEDITSAESESSEEEEGGPGDGQNATRQLEWDDSTLSY

>DmCSTN1

MTFHKTFGYGCIVLICFELLFAGVETSSENDDEYLTQKEIILEKSYHGLIRENETLVEITPLIKVNEEKICNFHILKKPYHEIPFKIELVNNLGILKARRTLNCENRKSYHFEICAIYCDGTPSNTANVHITVIDVNEYAPTFLEPSYVIEVDEGRLYNEILRVEASDKDCTPLFGDVCKYEILNNDEPFSIDNEGSIKNTEPLSHKASHNHILSVVAYDCAMKESAPIMVSIKVRRVCETKFVGMPERIDYTSGSTESLQLFPNARLDLCDISCKNEEDLRIHSSIALKTKHISFGCDRDISNCTSGQKVKDLLPHGAEWTKELSYDEGLEPIFHFDGSTGVVVPATVIDHYDFSSQPFSILTLFRHNSQVEINKHVKEHIVCSADDHKMNRHHMALFVRNCRLIFLLRKNFNEGDLNIFSPAEWRWKIPEVCDNEWHHYVLNVEDSSKVDLFIDGVRFENSIENRHSNPEVIDDWPLHAAHGVNTSLAIGACYQSLENRLKHGFNGDISEVKVSLNSVLTAEDIKCGTTCAEHLLAPKPLQSNNEKSYSDNSQIKENIEMNEIYISAKNKHDIEQFMRKVQYINTKQKPTVGRRNIEVLTTLNCKNESSLRLPPIETYIMVNEPIAPLGIDIDVVSASLETSDLTPPSYSPKIAISGTSNKLVSYQEIKLGVHILEKTCIDSVSKNNGKLEEKNHIDSCSVVVFPSLNPDHEDIKIDGDESLSSSMDIKTNINKDGVEMIGKDTISNYINVLRSLVYSNKKPAYYLNRVFKLSCAQQSSQYKSGEYTLTLTVLHPKQTLFKSTNVLPSSLSKVNFIGNTDNETSFHRNSGSVNGNDNNQPTESKVYSYSLLHTNNVQEPKSHIHSFIHKAEGSHVTMLIILVSVFLAVLLCGVSIARLKNNQKYIEHHQPCPKISDDGLIWDDSALTITINPMQADVTSDASSESENSESEDEEALKDGFTHINQLEWDNSNIFQQ

>CgCSTN1

MMKTLWVIGATLILMDFISGMSEPNSHDPVINSKLKNGRGLPVFHGNVTEGSRVVQLDQPLKATDQDVQGGARFICGYRVISPRKNIPFTVELLDRTTGEAQVKVAEGRKVSFEKRKRYKFSVIAYDCGQPARESNRAIIFIHIEDVDRFAPTFDKPDYEVDMEEDRLYDPLLTLHAHDRDRSKQFKEICDYEIKNPYVPFTIDKNGNIRNTQKVYYKDRHNFILKIIAQDCGGKPSKAVFVNILVKEKCRPQWTNFPEQIQYLANSGKQKVASGASLQWCDHTCVPDQVTLQMQLATKHIGKGCDRDTYSITSQRKLCGANDRSVDLLPSPSLTTSWTQYLPTDDGKESDQVFYFDGESNAVEVPENRFNHTLRKHFTIMTWMKHEHAELKDKKYRKEHILCMSDGENMNRHHYSLFVHGDKLVLLLRREAEDASDMEVFKPAEWRWKIPEINQRDEWHHYAISMNFPHVQLYVDGNMVIPDDDNSEVVDDWPLHKTNKVQDTKLIVGACWEGGHHKFNHYFRGYLAGLSVLKGKTESERVIRCLNNCQENLDFSALSAMHSGTTVSFNREMTDFRIQSKNASEVQALLREVHYINSRKFPTPGRRNLTVTTAIHCGSTEIFLPVVQSYILVRPAAEPSITIRGRSTLSSTVDKLKAGVPIFEDINISVDFAVENTEGKVNHILGKDEIQGDGDHNIELGKEEKLQLKLEKKKAKQDTTSKVSDAQFILLDSCTVKAEPTLDLLHERLELPTMIMNEFNMKIEGTQTREGLVISNADNIENYILVLRGIRYVHEKDTAFRDRSFHLSCSSQNGRFSSNVMDVKIMKSHEENNVVLPMRAQQAVQHIQLPSVSGTSNMAIGSASPNVGMIAIIVVCVGFLLFMIILGVIRIRAAHRRTQVVQVEPEMEWDNDMNITVNPMEQETHPLHVFDYDDGVGKSMRDDSDSEDDCSSYHDDEDDDSSDEETVPKKELEWDNSI

>SpDCHS2

MALSWRVCIALLVLWALICARTCSAQQDLLYYSVKENQPVGTFVGSLQDDTSFGSPFTTLNPSAEFMIESDTGIITTGAVLDRETAPDQEFVIIVFAGEMFTALVVTIRLNDTNDNNPIFSQDNRTFEVTEAVQPYMTKINGVTATDADEGINGIQGYRIVGGNIDNTFDLDVRVRETGVYMDIVVNKTLDRETISMYTLIIEAYDGGDPRLTANTTVNIKVTDVNDNSPVFTLTSFTATINESAPVNTTVLQVTASDEDEGLNGEVVFNLQRDDGFFRIDPASGIVYLNKVLNYEDAQAHDFLVIAQDKAEPPMSSQPAYVSIMVININEKPPSLEVLFLPDGGASQVSEAAVADTTLARISVTDPDDGVLTNVSMTITGGAGQFDLQKNSNQVYFLIVATTASSFDREEVASYDISIRATDHGSPPQHAEENLTIAVTDVNDNPPIFDLPLYHATIIEASEPGTPVKQVHATDADEGVNAQIIYRISPEGTDYSDWFEINPMSGLVTTLQKVDREETGSVFLTVIASDEGEISLSSSVIINITITDVNDNQPVFFPGSYNASILEEQEIPYCFLQVRKHFFYLGCFISLFNMGIELPPRRTENGTPSLQSVAKYVNYRTYLDVNGTYWDQNVLVEYSFFEDLSGTWKIFQLGVKSDIIALTEILNEKGFIIIPALDLLNSFTYCTYMYIYVRSSCIYNFLSFPSSYHLQVNATDPDEGSAGQVTYSPSTDLVPTPSQFSINPLTGDLCVISRLDRDAGQEEYNFAVKATDGGGLMSHAFVRVILEDINDNVPVFTSLNYRGHIEYDAALGTDVKLVVAEDRDAREFGRVTYTIDSSTNDGTFAINQTTGLITLVGSLDWVQHPVYTLEVIATDGGGRTSANHAQVTITVAGPDTSPPEFESSSYAFSVQENAAQDLVGAVKAGHIDPGITFPITYTLTSGDPNDFFRINTTSGEIFTRGSGPDHEQQAFVLITIQAASGMPPTYDDVQVNITVIDVNDNVPQFMAQTARTSIPESTGIGSSIYVATAMDLDSGDNGLVRYRLTSNPGNTFRIVEATGEIILNKEVSYNDQLSFEVDIQAYDAGEPSLEGSLTLTVIILDVNDNGPVFTMGFYPVSILENLPISTPVVQVEATDNDRGANALITYTLTPNNDSPYFAVFPREGWLYTRQVLDRELKSTFTLTIMASDSGSPPQNSSATVQVTVVDANDNAPHFTKKSYHFHINENLPAGTEVDRVEAVDPDLGPNGQVTYTLSPSGDFTIDEEGVIRTTHPLDREAAYSYRLIIRAVDGGNPALSSSATVYINVNDLNDNAPVFSQDRTYQASVREEQPAGEFVAWIIATDTDSGVLGNISYSLIDSSPKFIIDSSGVISTAAAIDREDESYYTLTVLAQDGGNPPMQATASVRVRILDLNDNVPIAAMSAYIFTADENIQPPTVVGVVSASDMDVGDNGKIYYYITEGNDFDVWDVNHSSGEVYNVRVVDYEMAAHYTLTIIAQDNNVPQTHSTSISVTINILDQNDNTPDFDQDLIYLTLQENVGINHVLWTVSATDADSGANGLIRYTILTEQDFFAVDEYTGTLRTIQNVDHEINHVFLLVIQAQDQAINVSARRAATSTVEVRIYDSNDNNPVFVSRSSTDVMEDEPVGFNVIHIIATDEDSGENGRVGYTITSGNEDGKFSLDAVTGLLTIAHSLDHEHKRDYELVILATDHGTVQRMATQTLTIRVIDVNDQPPRFEQSIYVMNVSESQAPGTYVGMVVAIDGDSGVNGRITYELPEEIARGMFRINATTGEIRTKAILDREQRDSYVVTVYAMDGAFPARYDMATVLVYVMDVNDNNPTFAPPTFDITLPENQSPGVIHNAIATDADEGPNGDLIYSITGGDPKNAFSINQSTGAIFTVGALDREDEATYTLTITATDQGTSPRSGTTTIRVTVTDLNDNDPVFGSMSYYKSIPESTAINATILTVVATDDDEGLNGDVYYTLDNTTIGLFSIDPEHGEITTTGKFDYEKETRYTFQVTATDSGVFGPRSERVQVIIDISDVNDNAPVFKTIPIRANVTQDASSNTFVANVEADDKDSGVNGEVNYRFTQQSSSFAIDTVTGVITTKSLNPGTLFYHLEVMAFDLGSPSLSSNGIVEVWVGTSGSGGLQFGQQTYLVQPSEAADNGDVVLSLSAFLPDGSSSNDIVYSLVSGNENGAFGIQVQAGGSAILVVADTTKLDYETQPNIRLVAEAMRTPENSSPMYGYATVQVELTDANDNAPQFVQDRYQSRVWEVPNSDIYVTQVSATDADEGTNGAIYYEVTSGNTDNAFAIDHVTGIVTTAKSLDYEIEDSYVLTVVARDGGSPQLTGTATMRIGIVDVNDNQPVFVQMDPVSVSEDLGVGLSVTTVTATDSDTNPNIQYSFTSTGNPGGYFAIDQYSGIITLARSLDRETQDGYTLELEATDTEYTTTMTLEVIVTDENDNAPVFRQESYQVTLPELTQPNVAVVAVNASDKDIGLNAELTYSFVDSHPSFYIDPITGVIFTNQVIEFNDVETTIQLYVTATDSGAPALSEFVTVRIEITDVNNQSPVFVEDIYSASVSENVSLGHHVITVVAEDMDLSPENSKINYFIESGNDQGKFKIDANDGSITVLERLDREATDTYTLIIGAVDNGEPVNNATAEVVIEVEDINDHSPIFDHVQYEGRIAENATASTYILTVHATDRDIGYNGEVEYSIISGTYSDLFAIDVVSGAVTVRGELDREEVERVEFTVQASDRGMENQLASVVLVTITITDFNEFPPYFPQLFYHFLVPENMPVETYVFTAGALDNDAGEYGELTYSIRGLGPSNGDDYFTIDPVTGEVFTAAVFDYESANSYRFVVQAQDTGGLSISIQTEVNITSVDEFPPMFNDQEYYFEIGDGADAGDYVGQVTATDADGGEDGVVIYSLTHEFFYIEATTGIIKVAKPLNSSARKRRGLEDRFTHRSRREIDLSAQSNFALKIIASTGKPGTLIDDTTAAVSVVNAASSIPVWIIAIVVVAILIILLFVVIGIILVCRQRRRRRKKEAQADKRSVAGSMSPRSYDVTFDPVEMGAQGMVNHGMNSSGHFPHRQLYGIPGSGGGMGHTNISEPSNSASSGRGSTTMEDEEIRRINEGGTGAPKNNIREKVIDSGIAQDHEDGSVSDVNSPREKHLNYLNSTSVESMHVFGEEGGGEAGGGLDIGHLIYHRLDEVGAEEDDAIMDGTRLFGFTEDGHPSMAGSLSSIVNSDEELSGSYNWDYLLDWGPQFQPLAHVFAEIAKLKDDTVAKRQMTDPRLQQKKSLHANVKNYPPPLLTNTPQGPIKPVAQRVMNNTSMSNHSQHLPRSPVVQESAFVPAVVSPDLSPSLSPLAPGSSSLSPLVTSTGVSSQSSRVTSGTTTPQRMHYGGGIVFTHPTANDDEIQI

>LvDCHS2

MALSWRVCVTVFALLALMSVRTCVAQESVSVYYSVKENQPIGTFVGNLQDDTSFDPTFTTFDQITEFTLEQGTGIIRTNVVIDRETADEQEIIMIVFAGATFGQTIVVTIRLNDTNDNSPIFSQNNRTFEVTEAVQPYMTKINGVTATDADEGINGIQGYRIVDGNIDNTFDLDVRVRESGVYMDIIVNKTLDRETISLYTLVIEAYDGGDPQLTANTTVNIKVTDVNDNSPVFTQTSFTAIINESAPVNSSVLQVTASDEDEGLNGEVVFSLQRDDGFFRIDPATGIVYLNKALNYEDAQAHDFLVIAQDKAEPPMSSQPAYVSIMVVNINERPPSLEVLFLPEGGAPQVSEAAVADTTLARISVTDPDDGVLTNVSMTIRGGAGQFDLEKNSNQVYFLIVASTANSFDREEVANYDISIRATDFGSPPQHAEVNLTIAVTDVNDNPPIFDLPIYHATIIEASEPGTPVKQVHATDADEGVNAQIIYRISPEGTGYSNWFDINPMSGLVTTLQKVDREETGSVFLEVIASDEGETSLSSSVIINITITDVNDNQPVFFPGSYNASILEEQEIPYCFLQVRKLFLYFLLVLLMVSEETLQVNATDPDEGSSGQVTYSPSSGLVPTPSQFSIDPRTGILCVISRLDRDDGQDEYNFAVKATDGGGLMSHAFVQVVLEDINDNFPVFTSLNYRGHIEYDAAIGTEVKHVVAEDKDAGEFGRVTYAINPATNDGTFSINQTTGLITLIGSLNWVQHPVYNLEVIATDGGGLISANHAQVTITVAGPDTSPPEFESSSYTFAVQENAAQVLVGRVKATHVDPGITFPITYTLTSGDPNDYFRINTTSGEIFTRGSGPDHEEQAFVLVTIQAASGMPPTYDDVQVNITVTDINDNNPRFMARTVHTSIPENTQIGTPIYVATATDRDSGDNGLVRYELASNPGNTFGINAETGEIRLIKEVSFNDQLSFELEIRAYDAGTPPLKGNLTLTVIILDVNDNGPVFTQSFYPVSILENKPISTPVVQVEATDNDRGANALITYTLTPNDDSPYFAVFPREGWLYTRQVLDRELKSVFILNIMASDGGNPPQNSSATVQVTVVDANDNAPHFTKSSYHFHINENQPAGTEVDQVEAVDPDQGANGQVYYSLTPSGDFTIDDEGVIRTTRPLNREAAYSYRLTIRAVDGGNPVLSSSATVYINVNDLNDNSPVFSQDRTYLASVKEEQPAGEFVAWIIASDADSGVLGNISYSLINSSPKFVIDSSGVISTAASIDREDMDYYTLTVLAQDGGNPPMQATASVRVRVLDLNDNVPIAAMSAYIFTADENIHPPTVVGVVSASDMDVGDNGKIYYYITEGNDFDVWEVNHNTGEVYNVRVVDYEMAAHYSLTIMAQDNSIPQTHSTSIDVTINIIDQNDNTPDFDQNLIYLTLQENVGINHVLWTVSATDADSGPNGLIRYSILTDQDFFAIDEYTGTLRTIQNVDREINHVFLLVVQAQDQAVNVSARRAATSTVEIRIYDSNDNDPVFVSRTSTDVNEDEPIGFNVIHIIATDEDSGENGRVGYRIISGNEDGKFSLDSVTGLLTIAHSLDHEHKRDYELDILATDHGTVQRTATQTLSIRVIDVNDQPPRFEQSIYTMNVSESQGPGTYVGTVVATDLDSGINGKITYELPEEIARGLFTINSTTGEIRTISILDREQKDSYVVTVYAMDGAFPARYDMATVLIFVMDINDNNPVFAPPTFDITLPENQSPGVIHNAIATDADEGPNGDLIYEITGGDPNNAFSIGQSTGALSTLRALDREDLAMYTLVITARDQGTSPRSGTTTIRVTVTDLNDNDPVFDTMSYYKSIPENTAINSSILTVVADDADEGLNGDVYYTIDNTTVGLFTIDPEEGEIMTTGKFDYEKETRYTFQVTATDSGIFGPRSERVQVTIEISDVNDNAPVFKTIPIRANVTQNASPNTFVANVEADDKDSGNNGEVNYRFAQQSSSFAIDTVTGVITTKTLNLGILFYHLEVLAYDLGSPSLASNGIVEVWVGTSGSGGLEFSQPTYVVQPSEAADNGNVVLSLQAFLPDGGRSDDISYSLVSGNENGAFGIQVQSGGNAILVVADTTKLDYETQPNIRLVVEAMRTPENSSPMYGYTTVMVGLTDANDNAPQFVQDRYQSRVWEVPNSDIYVTQVSATDADEGTNGAIYYEITNGNTDNAFAIDQVTGIVTTAKSLDYEIEDTYILTVIARDGGSPQLTGTATMRIGIVDVNDNRPVFVSMDPVSVSEDLGVGLSVTTVMATDSDTNPNIQYSFTPDGNPGGYFAVDQYSGIITLAHSLDRETQDRYMLELEATDTEYTTTTTLEIIVTDENDNAPVFRQESYQVTLPELTQPNVAVVAVNASDKDIGDNAALTYSFVEDHQSFYIDPITGVIFTDQVIEFNNVETTIQLYVTATDSGTPALAEFVTVRIEITDVNNNSPQFGEDIYSAIVSENVSLGHHVITVVAVDLDLSPENSQISYFIESGNDQGKFQINANDGRITVLEKLDREATDSYTLIVGAVDSGEPKNNATAEVVIEVEDVNDHAPIFDHVQYEGKIAENATVDTYILTVHASDRDIGYNGEVEYSIVSGTYSDLFAIDVTSGAVSVRGELDREEVETVEFTVQASDRGMEDQLASVVLVTITITDFNEFPPYFPQLFYHYVVPENMPVETYVFTAVALDNDAGEYGELTYSIRGLGSSNGDDFFTIDRVTGEVFTSAVFDYEATNSYRFVVQAQDSGGLSISIQTEVNITSMDEFPPVFNDQEYNFEIGDGVNTGDYVGQVTATDADGGEDGMVIYSLVHEFFYIEASTGIIRVSKPLNSSARKRRGLEDRFLHRSRREIDLSAQNNFALLITASTGKPGSLTERTTAAVSVVNAASPIPVWVIAIVVVAILIILVFVVIGIVLVCRQRRRRHKKEAQADKRSVTGSMSPRSYDVTFDPVEMGAQGMVNHGMNSSGHFPHRQLYGIPGSGGGMGHTNISEPSNSASSGRGSTTMEDEEIRRINEGGVGAPKNSVREKVIDSGIAQDHEDGSVSDINSPREKHLNYLNSTSVESMHVFGEEGGGEAGGGIDIGHLIYHRLDEVGAEEDDAIMDGTRLFGFTEDGHPSMAGSLSSIVNSDEELSGSYNWDYLLDWGPQFQPLAHVFAEIAKLKDDTVAKRQTADPRLQQKKSLHANVKNYPPPLLTNTPQGPIKPVAQRVLNNTSMSNHSQHLPRSPVVQESAFVPAVVSPDLSPSLSPLAPGSPSLSPLVTSTGVSSQNSRVTSGTTTPQRMHYGGGIVFTHPTANDEEIQI

>ApDCHS2

MTCIALCLGQEQTPSITYNIEEGLPVGTQVGDISVDQQIAGPYLSVHVPADYLEFTQDGIITTKAVLDHEQESVFDFFALPITPNPPQIAIRVSVTDVNDNSPVFPNSEIDLDLSESTPIGAKRSLDSARDADDGIFSTQGYRILSGNVDNKFELDFRTAPNGEVFLDLVINDTLDRETTPNYTLVIEAYDGASPPRLGNMTVNVNILDINDNPPVFAPTRYSSTVNETLAVGSPILQVTASDADEGINGAVMYEVLPQSDPNEIFGIDPNTGWLYLNKELDHELFDSIVLVIMARDSATQPERSYAYATINILNVNEQPPNINIVFLSEDGEPKISEAAVPGDFVARVSVNDPDEGDLTNVNVTLIGGEGTFGIMTRNNIIYLICLQSSLDRELIPSYDLSITARDFGIPPLHTQVDITIYVEDINDNPPIFDEVEYSASILEIVERGSMVTQVHADDADAGMNAAVTYQIVNEPGSYSEWFTINSESGLITTDQLIDRETVDTVRLTVQASDAGIPSFSANVTVVISLRDVNDNQPQFLLSSYNASIPEDTNINTCFLQVQAEDPDSGNFGLVTYSLSLGYGSQPPPEFYIQSDDGYICTATQLDRDAGRTSLEFPVQATDGGGLSSIALVKITLLDVNDNRPVFYPSTYAVDVDESSPEGTVITTVSAQDQDSGTFGRVTYSILSGNSQGIFSVADESGVITLIGTLSRQQQSLHFLEVSAVDGGGQSSLDNAQISISVVGPDDSPPMFNQSHYSFTVRENAQRFHILGSVHARNVDPTNLAVISYSIYSGDPQGYFSLNPATAELSINSPPDYELYPYLILTIQAASGNPPLYGLTNVNVTIIDENDNTPQFPKNREVVVVPESTAVGSIVFVAMATDRDSGDFGLVRYKLVQNPDNKFSVHRVMGQVVLEDEISYNDQSSYEVIVQAFDRGSPSLKTNLTLNVLVQDVNDNGPQFNPATYQMDVSEATPISTRVVQVMATDQDQGINARITYTLRPSVDATYFAIFPDDGWVYTRQRLDYELRSEFILDVIASDNGSPPQNSSAVVRLLVTDMNDNSPQFAQTTYYLNLDENMDANTEAGQVVAEDRDSGINAQITYTLEGSSAFGINPSTGVITTTQSLDRERTASYDMTVTATDHGQPPNTAVTSVHVSVNDLNDNRPTFLHRSQYDASIMEEQTPGEFVAKVIAFDPDSGENGTITYSFESGGDHFDLDSTTGLITTKSTLDRERNQLYMISVIARDNGTPPLQSIIDVVVHIQDINDNVPVAERAAYTFTIEENHEPGSTVGQVRAHDPDAGDNGKIFYSIIDGNDYALFGINRTSGVIINTRVVDFELSSHYQLTVFLEDNGPLHPQTTRVTVNINVLDMNDNAPVFATDPIQFTLSENVAIGHLVRTFLATDADSGTNSDIRYSIQTPQPMFSIDEITGALTTTAEIDRESDSRFLFVVQATDQAAILSDRRSSTVTVSILVEDRNDNAPVFESRDFTHINEDEPVGYPILHVHASDADVGENSRLVYEIKSGNEEGRFAIESITGLLSVAHPLDREHETLYTLNITATDHGLPPLTSWQTLEINVGDVNDLPPHFDPSSYVMEVREGVVRGTYVGTVTAQDGDTGTNGDITFEIPTGIADNKFTVDASTGVIVTDGELDREAKDSYSVTVYARNGGFPVRYDVASVIVYVQDSNDHAPGFATSHYELSVPENEPASAVHVVVAEDKDIGDNSNLTYSIRDGNIGGLFELNSVTGQLSTTGPLDREVTPQYNLTIRATDNGVPSQSGDTVITVNVLDENDNDPVFTMPSYHYELAENTGIGVSLMTVEAVDADEGTNAMVQYSLDNSTLGLFGIDVNSGQLTTTGLFDFEKESQYIFQVCATDGGSFGPRSEKVQVVVDVTDINDNAPVFTMVPFRANLSLGSLSGAYVTRIVAEDKDSGDNREVNYRFAQPSAEFTLDASTGVVLTSVVMDTEGLYRLEVEAYDLGTPSLSTRGLVEVHVGNAAAVRLEFLSSEYAASVLEDESAHTQILILKAVRSDGNPAGQVTYSIISGNDDGAFEITGQSDSGILSVVDSSTLDYETTQQARLVVQAEAPSIGAVPLYGYATVLLNLLDANDNIPRFVQDKYTTSVWEGQAVGIYVIQVSANDADQGGNGQITYTIRSGNFDNAFNLDPVTGIVTTNAHLDREIRDNYKLTIAARDSGSPQLMGSATLKITVVDDNDSRPRFPPMNPLVISEAAEVGYPLVTVTANDADTYPTLTYQFTAGGNPDNKFAIDQFSGSITLAEPLDREQRDIYRLEVQASDSRYTDEITLVVRVRDENDNEPQFSQQSYQAALSELTPSGYPVLTVNATDADIENNAQIIYSMGVAPVQGFVIDPVTGAITTNQTINFNPAQPIIQLVVTATDHGIPPLSSVVAVRVQVLDINNNAPTFEYALYSASVDEDKSIGWLVTTVTAKDEDPSYHNQKIDYTIVSGNEDGKFEILNEGATYEIMNEDENFKSGDIVLIDALDRELVDSYTLIVAATDRGLPQRNSTAEIVIRVVDVNDHAPRFNQTHYVGTVQENATTNTSVVQVFASDLDEGRNAEILFDIKSGNEMGMFSINATSGLISVEGNLDHDTIPEVRMSVRATDRSADNPLHAVVQVIIEITDYNDNIPYFPFMMYLERVAENQPAGTSVFQAHAIDKDTGEYGQLTYAIIEGSGKDLFNINSVSGEVTTVRPFDYEKEESYRLLIRAVDKGGESVSVQAQVEITSQDDYQPHFDQKEYLFSVAQDAAVGTVVGTVHASDNDTGADGVVLYAFERSGEEYEHEFFAINQSTGIITVKKRLNSSSRRKRDLPGAIYRNRRQIAEGKDQFSLIVKASSGKQGSKEDRVSITLNIGPSTTPGGYAGLPTTTILAIVIPVVFLIILIIIILFFIARRRRRENRKKDSQPPPYQAGERSLSPRSYDIAFDNVEMGHSAMVNHAMIPEQPLTAGDTSYNIYPARNLYNMHRNNMNSSMEVGHLTRTDISEGNSASSGRGSTTVEDDEIRRINERSVSDDHTASVRDKVLDSGIQQDHEDGLSLRDTASDVMGSPREKNISYMNSASAESMHVFGEEGGGEAGGGMDIGNIIYARLDEVGAEEDDAIMDGTRAFGWGDDIQPSMAGSLSSIVNSDEELSGSYNWDYLLDWGPQFQPLAHVFAEIAKLKDDTVVKRQLERPQQKHKLVKQYPPPLLTNVVQGPIKPVAPLAMNNGQSTQNPQTLPRSPIVNESAFSAVAVSPDLSPSLSPLAPGSMSISPMVTSTGVSSTQSSSRGNSGSSTPQRASRNPRQVASIRFVPSFTGDEEEIQI

>PmDCHS2

MAFPPCTGRHRTNVKLNVKPWRNLSSVVLLLMMYIAVCLGQAQTPSITYNIDEGLPVGTEVGDISVDQVIPGPYLPLYAPTDYLQFTSSGVISTKVVLDHEATSEFNFVAVAISDSSQIVIRVRVTDVNDNSPVFPNSEINLDLSESTPIGAKRSLDSARDADDGIFSTQGYRILSGNVDNTFSLLFRTAPNGEVFLDLVIENSLDRETTPDYVLVIEAYDGGSPARLGNMTLNINILDINDNPPIFSPTQYSTTVNETLAVGSRILQVTASDADDGVNGVVIYEIRRQSDPNEIFRIDPNTGWLYLNKPLDYETAEAVVLAISARDNTTQPERSFSYVTINILNVNEQPPNINIVFLHQDGQPKISEAAVPGDFVARVSVNDPDDGELTNVNVTLLGGQGKFGIMTRNNIIYLICLQSSLDRELIPSYDLSITARDFGIPPLSTQLNITIYVEDINDNPPIFDQEMYSASILEIVETGSMVTQVHADDADIGDNAAITYQIMNEPGSYSEWFTINPASGLITTEQLIDREIADTVRLKVQASDGGVPSLSANVTVVITLRDVNDNQPQFLLSSYNASIAEDTDINTCFLQVQAEDPDSGDFGHVTYSLSLGYGSQPPPEFYIRNDDGYICTATQLDRDRGTTSYEFPVQAMDGGGLNSIALVKITLLDVNDNRPLFYPTTYAVDVDESSIEGTVITTVSAMDQDSGTFGRVTYSIVSGNEQGIFTIADGVVTLTGALSRYQQSMHSLEISATDGGGQSSLENAQIRISVVGPDDSPPVFNQSHYSFTVRENTQRFHVLGSVYAENLDPTNLAVISYSIYSGDPQGYFSLNPATAELSINSPPDYELYPYLILTVQAASGSPPLYGLTHVNVTIIDENDNTPQFPSNREVVVVPESTAVGSTVFIAMATDRDSGNFGTLRYKLVQNPDNKFSVHRVTGQVLLESVISYNDQSSYEIIIQAYDRGSPSKKGNLTLTVLVQDVNDNGPQFNPATYQMDVSEATPISTRVVQVMATDEDHGINARITYSLKASIDATYFAIFPDDGWLYTRQRLDYELRSEFILEVIASDNGSPAQNSSAIVRIRVTDMNDNSPQFAQTTYYFYIDENREANSEAGRVIAEDRDSGMNAQITYTLEGNSAFRIDPSTGVISTTQSLDRENTASYDLKVTAMDHGEPPNMAVATVHVGVNDLNDNTPTFVRQSQYHASIMEEQMPGEFVAKVIAFDPDSGENGTITYSFASGGEHFNLDPSTGLITTQSTLDRERSQMYIINVTAQDNGTPSLQSSIQVTVRIQDINDHMPVAERAAYTFTIEENIEPGSTVGQVRAHDQDAGDNGKIFYSIINGNDYGTFGINRTTGVIINTREVDFELSSHYQLTVLLEDNGPVHPQTTTVTVNINVLDMNDNTPVFANDPIQFSLSENVAVGHLVWTFLATDADSGTNSDIRYSILTPQSTFTVDEITGALTTIAEIDRESIPRFLFVVQAEDQATVVSDRKSSTVTVSILVEDRNDNSPVFLSRVFTHINEDEPVGYPILHIHASDADLGENARLVYEIKSGNEEGKFAIESITGLLSIAHSLDHEHERLYVLNITATDHGLPSLSSWQTLEIRVEDVNDLPPHFDPSSYVMNVFENLDRATYVGTVVAEDGDTGTNGEITFEIPYGIAEDMFTVDASTGVIVTNGRLDREAKDSYSITVYARDGAFPARYDVASVIVYVQDTNDHDPTFATSRYELSIPENEPASDVHVVVAEDKDIGVNSNLTYSIIDGNIDGLFVLDSITGQLSTTGPLDREVTPQYNLTVRATDHGAGPRSGESVVTVNVLDDNDNDPVFLMPSYHYQLAEDTGIGVSLMTVEAVDADEGTNAMVQYSLDNSTLGLFGIDVNTGQLTTTGLFDFEKESQYIFQVCATDGGSFGPRSEKVQVIVDITDVNDNAPVFTMVPFRANLSLGALSGAYVTRVVAEDKDSGVNREVNYRFAQTSAEFTLDATTGMILTSVVMNTEALYRLEVEAYDLGTPSLSTRGLVEVHVGNAAAVRLDFLSSEYSASVLEDESADTQILILKAVRSDGNPSGQVTYSIISGNDDAAFKITGQSDSAVLSVLDSTSLDYETTQQVRLVVQAEAPSIGAVPLYGYATVQLNLLDANDNAPRFVQDKYTTSVWEGQSSGIYVIQVSANDADQGTNGQITYSIRSGNFDNSFNLDPITGIVTTNIELDREIRDAYKLTLAARDSGSSQLTGLATLKITIVDINDNRPRFPPFNPLVISEGAEVGYPLITLTANDGDTNPTITYHFTPTGNPGNKFAIDPFSGSITLAETLDRETQDIYSLEVQASDTVYTDDVTLVVRVRDENDNEPQFSQQSYQVALSELTPSGYHVLTVNATDADIDNNAHIVYSMGVAPVQGFVIDSVTGAITTNQTINFNPAQPIIQLVVTATDHGNPPLSSVVAVRVQVLDVNNNAPAFQQDIYSAFVDEDKAIGSLVTTVTAEDEDPSYHNRKIDYSIISGNEDGKFEIKADSGDINVFDSLDRELVDTYTLIVAATDRGLPQRNSTAEVVIEVLDVNDHAPRFNQTHYFGTVQENATTNTSVVQVFASDLDIGRNAEIQFDIKSGNEMDMFTIDVTTGLITVKGSLDYDTVPEVRMSVRATDRSGDNPLHAIVPVNIEITDYNDNVPYFPFMMYLERVAENQPAGTPVFQAHAIDKDTGEYGQLTYAITEGSGKDLFNIDPVSGNVTTVRPFDFEMEESYRLLIRAVDKGGESVSVQAQVEITSQDDYQPHFDKKEYLFSVVQDAAVGTMVGTVHASDNDTGADGVVLYGFERSHEFFAINQSTGIITVKKRLDSSNRRKRDMIESLYRNRRQSVDGKDQYSLIVKASSGRVGSKEDIVPITLNVNPPAVPGGQVGLPMTTILAIVIPVILLILLIIIILFFIARRRRRENRKKDPKPADYQAGDRSLSPRSYDTTFDTVEMGHSAMVNHAMIPEQPLTAGDTSYNIYPPRNLYNMHRNNMNSSMEVGHLTRTDVSEGNSASSGRGSTTVEDEEIRRINERSVSDDHTASVRDKVLDSGIQQDHEDGLSLRDNASDVMGSPREKNISYMNSTSAESMHVFGEEGGGEAGGGMDIGNLIYARLDEVGAEEDDAIMDGTRAFGWGDDVQPSMAGSLSSIVNSDEELAGSYNWDYLLDWGPQFQPLAHVFAEIAKLKDDTVVKRQLERPQQKHKPVKQYPPPLLTNVVQGPIKPVAPLAMNNGQSKQTSHSLPRSPIVNESAFAAVAVSPDLSPSLSPLAPGSMSISPMVTSTGVSSGQSSSRGNSGSSTPQRASRNPKQVTGIRFVPSFTGDEEEIQI

>ArDCHS2

MLILLLCISLCHGQALTELNYNIEEGLPAGSIVGDISSDQSAPGPYLVILIPDEGQPFEMTSQGVIQTKEPLDHEDLSFFSFIAVPLSSSTTQIYIKITVTDVNDNYPVFLNLETHLDLSEATPISTKLSLDSATDRDEGVFSTQGYRILSGNEDGTFILEFRTAPNGEVFLDLVVYQSLDRETTSNYTLIIEAYDGATPPKTGNMTLNINILDINDNPPVFSPTLYSATVNETLAVGTKILRVTAFDADEGLNGAVIYEVRRQSDPGELFRIDPITGWLYLNKALDYETASSNVLVIIARDNGTQPESANAFVTISILNVNEQPPMINIVFLSGNGEPKISEAATPGDFVARVSVNDADDGELTNVNVTLVGGEGQFGVITSNNIIYLICLQRPLDRELVPSYDLSIHAHDFGIPPLHTRQNITIYIEDINDNPPIFEEVEYSASIIEIFEIGSMVKQVQADDADAGSNAAVTYRILDTPGSYSNWFQIHPVSGLITTKELVDREIQEIVTFIVEARDSGDPSLAANVSVTVTLRDVNDNQPQFLMSSYNASITEDTEIGSCFLQVEAEDPDSNSFGPISYSIGIGFGYQPPPEFFIHNDNGFICTAALLDRDAGITSYEFLVQATDGGSLSSVASVKITLQDVNDNNPRFYPSTYAVDVEESSMDGTVITTVSATDLDSGLYGSISYSIVAGNDMDLFTIDPIQGIVTLTGTLNRQQASVHYLQVSAMDGGGQAAQENADISISVVGPDDSPPVFYQQQYTFTVDENTPRFHFIGTTTAANADPSNLAVISYTIYSGDPQGYFTLHPANAELTVNSPPDYELYPFLLLTIQAASGSPPLYGLTQVNISIVDINDNSPQFVSNKEVVVVPESTSIGSDIFIATATDRDSGDFGLLRYKLVLNPGNTFSIHRVTGQVILEEEISYNVQSSYEVIIQAYDRGSPSRKANLTLSVVVQDVNDNGPQFNPATYQMSVSESTSISTQFLQVMATDQDQGVNAQITYSLKPSIDAAYFAIFPDDGWVYTRQRLDFELKQEFIIEVIASDNGSPPQNSSAIVRITITDENDNAPQFQQETYYFMIDENLDANAMVETVVAVDRDSGRNSQLTYTLSRSDAFIINSNTGTITTTKMLDRETEASYKLTVSVADHGEPSNTATAVVYVKVNDLNDNPPIFSQSLQYHVSVMEEQNPGDFVAQLIATDLDNGENARITYSFLRGGEKFNIDPSTGLITTDSSLDRERSQVHIITVLAQDHGSPSLQSQTQIVARIQDINDHIPVAERDVYSFTIAENIFPGSMVGQINAHDQDAGDNAKIFYSIVDGNYNNMFGINRTTGVIINTKQVDFESLSYYELIVLLEDNGPVQPQMSRVIVHINILDVNDNTPVFATDPIYLGLSENVPIDHVVWTFSALDADTGTNSDIRYSILTPQSTFSIDAVSGVVTTIADIDRELVTQYLFVVQAEDQAEDEMDRKSTSVTVSILVEDRNDNTPIFVSRDFTHINEDEPVEYPILHVHANDADLGENARLIYEIRSGNEEGKFVMDSITGLLSIAHSLDHEHERLYHLNITATDHGLPPLTSIQILEIRVEDVNDLPPHFDPSSYVMNVFENLDRATFVGTVVAEDGDTGTNGEITFEIPYGIADNMFTVDSTTGVIVTNDRLDREAKDSYSITVYARDGAFPARYDVASVVVYVQDANDHNPTFLLAYYVLSVPENEASSLVHVVVAEDKDIGDNSQLSYFIVGGNIDDLFQLDAVTGQLSTSGPLDREFTAQYNLTIRATDHGDTSRSGESIVTVNVLDDNDNNPIFSATSYHHQLSESTVIGQSLLTVEAVDADEGTNAVVQYSLDNSTLGLFGIDVNTGEITTTGLFDFEKQEHYIFQVCATDGGSYGPRSEKVQVTVDVIDVNDNAPVFTMVPFRANLSLGAPSGTYVTRIIAEDKDSDVNGQVNYQFVQPSAQFNLEASSGVILTTVDMNTSALYRLEVEAYDLGSPSLTSRGLVEVHVGNAAAVRLVFTSPEYSASVDEDESPHSQILIVKAIRSDGNPVGAVSYSIISGNEDGAFEVTGQSDSAILSVLDSTSLDYETTKQVRLVVQADAPSGGAVPLYGYTTVQLNLLDANDNSPRFVQDKYTTSVWEGQSSGIYVIQVSANDADHGTNGQISYFIRSGNIDNAFNLDANTGIVTTKANLDREIRDSYKLTLEAADSGSSRLTGTATLKITIVDINDNRPRFPPVIPVIISEGAEVGYPVTTVIANDADTNPSIIYQFTSTGNPGSMFAIDQFSGSITLAEPLDREIQDIHSLEIQASDGVYTDTLTLVVRVRDENDNEPHFSQQSYQVTLSELTPSGYPVLTVNATDADIDNNAHIVYSMGVAPVQGFVIDAITGAIRTNQTIEFNLAQPVIQLVVTATDHGNPPLSSVVAVRVQVLDVNNNAPAFTQTIYSAYVDEDKAIGWPVTTVTAEDQDPSYHNRKIDYSIVSGNQAGKFEIKADSGDIVVFDSLDREVEDTYTLIVAATDRGLPQRNSTAEVVIEVQDINDHAPRFTQSQYSGSVQENATVNTSVVQVSATDGDIGRNAEIHFDIRSGNELDMFTIDELTGWITVKGNLDHDTVPEVRISVRATDRSIDNPLHAIIPVYIQITDYNDNVPYFPFMMYLERVAENQPAGTLVFEAHAIDKDTGEYGNLTYAITDGNGQDFFNIDPITGNVTTKQPFDYEAEDSYRLVIRAIDRGGESVSVQAQVEITSRDDYSPLFDEDSYSFSVAQDAAVGTKIGTVHAKDADKGIDGVVVYAFERGDEFFAINQSTGIITVKKRLDSTNRRKRWVDDHTYRKRRQATDGKEQYSLIVRASSGKNESKEAIVSIVLNVGPPSTGGRQTATPVTTILAVAIPIFVIIILVVIFLCFIRVRRKREYRQKNPKPSDYQAGDRSLSPRSYDATFDAVEMGHSAMVNHAMTPEQPLTAADTPYSMYPPRNIYNMHRNNMNSSMEVGRTDVSEGNSASSGRGSTTVEDEEIRRINERNVPEDQGGSVREKVLDSGIQQDHEDGLSLRDNASDIMGSPREKNMSYLNSASAESMHVFVEEGGGEAGGGMDIGNLIYARLDEVGAEEDDAIMDGTRAFGWGDDIQPSMAGSLSSIVNSDEELSGSYNWDYLLDWGPQFQPLAHVFAEIAKLKDDTVVKRQLERPQQKHKAAKQFPPPLLTNVVQGPIKPVAPLAMNNVVTTSNSHVLPRSPIVNESAFAAVGVSPDLSPSLSPLAPGSMSISPMVTSTGVSSGQSSSRGNSGSSTPQRTARNPRQAASIRFIPSFAGDEEEIQI

>AjDCHS2

MSSLICCHHPFKLSFNWKSSIVFCFIAIIFARTTNGQADVTFEVEEGLPAGTIIGVLGNPPYENSFDISECTSYIAFEKLDGTLKTKEILDRETVASCELIVFSVSNDVIQVKVDIIDINDNPPIFPNPSSVMNISESTPLTRLNIDYATDADSVVYGIKRYEILSGNEEGTFNLSSKSLPSEDTLFLDLELLSELDRERVAQYTLVIAAYDGGNPSLSGTTTLIINVEDVNDNAPMFLLTSYSATVNETVPVGTRILAVTATDLDEGPNGRIFYEINRRQSDPNEIFRIDPLTGDLYLNKPLDYEVQQTHEIIVVAKDSSPDPLENTAFVTITVTNINEGAPTIMILFFSNDGNPKISEDAEPGDYIGRVSVNDPDEAELTNVSVILTGGNGNFGIETSDNIIYLVCIKRVLDREQTPYYNLTIFANDYGSPPLNAHKNFILEILDVNDNPPEFDQSDYYPQIQEVADPGTFVTQVGATDADAGVNAQITYSIASGPQASWFHVNPVSGLITTSSQVDREISSVVDLTVVASDGGTPSLSSSAMVHVTIRDVNDNQPQFTPSSYNASVHEDIANGTCLLQVSQSSILIMFIHTNCLFRFKLNYKQSKVLLRLSNFIMMFGNEDRFIKLIFLQLFLQVVAIDPDADGFGRIQYSLPLNYGAPPPPEFFIKSDTGQICTTMTLDYDVLPRAYEFLVQASDDGGLSSVARVQISLIDVNDNRPVFYPEFYSENKSESTAVNTNLVTVFAQDADSGDFGKVTYAIVAGNDLKLFAIDQNTGILRLNGSLNRQQRPLHRLQVQATDSGGLTSLKNAEISVSVLGADDSIPVFDMAHYEFSILESADRYDVVGTVHATNQDPANQDVITYSISSGDPNSYFSMNPTTGEITVNTLLDFESDEQVTLQVKASSGSPPSYDDSFVLITVLDVNDYTPRFTSNEVQIPLSESTPVDSFVFYASASDIEKGLNGEVRYRFNENPEETFYIVQATGEVKLAKALDYNVRDLYVCKIEAYDLGTPSLSSVLTLEIRILDMNDNGPRFEISPYNVDIPEDLAVSQRFLQVSATDNDRGSNARITYSIRASDDAAYFSIFPDTGFLYTKSPLDRELKAEYVLQVIASDNGVLPQNATATVYVHITDINDNAPLIESESFYFYVTENMAMDTVIGTIGAEDRDTDDNSHLIFSLRETSKFQINANTGEITTTKSLDREVLSAYDFIVVVADRGQPPLSSQAVVHVVVNDLNDNPPTFLNSMAYTAEVLEEQPVGTPVLRVVASDPDKGTNGSVRYSLISGSRMFHINPTTGEVTTTQVLDREEVSQYMISILAQDGGTPPMESTIMVQVQVKDVNDNPPLVENETITLTVMENVRPGTELGRIKATDVDEGGTSKVTYYIVAGNDFGIFSINRTSGSIFNVKELDFEFKAHHHLTIEVHDNQIQPQSTTAYANINVVDENDNAPVFASNPIGFGVEENIPADRTVWTFSATDADSAENGVIRYRIATTQSQFRIGAESGELITISTIDRESYLQSRITLVVVAEDQAVDPADRLMTSVTVQIVIEDKNDNRPVFTSRPQTFAMEDEPIGYPVIHVVAIDNDLGENARVVYQITSGNEDGKFLLDAITGVLSVAESLDREHRHAYSLNITATDNGLPQLSSTQNLTVFVEDVNDMPPHFEPTSYIMNVSENMLPGTVIGQVYATDGDTGENGRLTYNIPSGIADDMFTVVADTGELKTTSQLDRETKETYIVTVYVRDNAFPARYDLASVLVNVVDENDHPPMFGVDTYQLDIPENQPGTVVHTVAATDNDFGVNGALEYSIIGGNVDNSFSIDPVTGQLATTGALDRETTEHFSLVIQASDGADPSMTATMVISVDVGDQNDNDPVFTSQSYSASLPENTPQNTTVLTVHAVDEDLGVNAAIQYSLDNSTQGLFTIDPDTGIIRTSGTFDFEVRHSFIFEVKATDGGPYGPRTEKVQVTVDITDVNDNAPIFETEPILANITQDAMQNTYVTTVKAEDKDSNDNSRIKFSIDEDNIQYFTIDEDSGVITVNSQLSPSIPLYRFEVIATDQGTPPLTNSVVVEVRVGNAAISSLNFDALEYEASIPEDARYNTPVQTVRAEHSDKNNNSPIMYSIIRGNDDGAFFINENSGAITVAQENVLDYETTANVQLLVQAKSESGGTVPAQGYAKVNIHLQDSNDNSPKFGQERYATTVFEGSNENYYVVKVSATDDDTGSNAQITYRFANTGQSDHFRIDPLTGIITTNVQLDREVEEQYKLTLEAVDGGTPPNTGSATIKISINDKNDNAPYLPIIQPVSINEGAKVGTVVYHVQANDADKDPTLTYDIVDGNINSEFQIDRFSGTVTLNAPLNREEREQYRLKISVFDGVETAERDMVVNVLDENDNAPVFSEQSYEVTIPELTPPGYAIETVNATDMDEGTNSQIVYSMGVAPILGFYVDPITGTIYTNQTIDYNPSQPVIQLVITATDHGNPPLSAVVAVRIQVTDVNNNSPNFEQNLYEGSVMENAPIGSDVLAVHAEDSDPSYDNNNIFYSIISGNEENAFRVDGNSGDITVLGLLDREQKSVYTLVVLATDRGSPQRNSTAEVVITITDVNDHSPVFESAVYRGNVSEAADIGTSVLEVRATDADEDAKIRYEIISGNDKDLFKIDIRSGVISVKSILDHDVQNPQVVLSVQASDDLQLSEVNVIIEILDENDNKPYFPSLMYIESVKENQPVGTSVFTAQAEDKDTGIYGSLTYSIQGGDGQDFFSIDPNTGEVTTLVEFDYEDTDVYRIHILATDYGGEKKAVQAQVDIKSVDEFQPVFSVLEYQFDVPSDAAKGTSVGTVHATDQDKGEDGVVRYSFENFEEYFVINETTGVISVSKNMSEARKKRSANAKKRKIREAPVQEAILVRASTGRPDSLEARATVTVVVEPATHIGTGSNLNLILAITLPLIVIVLIILVILLYLLLKRRNGQQKPNNTYAQGRYSPPTYDSSTFDRVDMSHGIMANGAIPISPADMHRIGNLYAMRGANSSMEVGHLTRTDISDQSNSASSGRGSTTVEDEEIRKINEGSRKSDHSSNQRDKVIDSGIQHDHEDNGSVLDDNDILQNGKVINGFLSSKSVESMHVFGEEGGGEAGGGVDIGNIIYAKLDEVGAEEDDAVMDGTRSFGFDDHGQPSMAGSLSSIVNSDEEFSGSYNWDYLLDWGPQFQPLAHVFAEIAKLKDDSVARRQIEQRPPQQKSAFQPKLKTHPPPLITSIAQGPFKPVVPSSSSRTRDSSPRSPISHENIFQSLAVSPDLSPSLSPLAPGSPSISPLVTSTGLSSLQTNSGQVTPQRMNNRGHYSVKLPPLGSGNQEEFQI

>SkDCHS2

MWLVICIVLLHSPFLSADTTQSLIFVTQEGQAPGTLIGDVATMYEETYGEIVEGPFFLSPASPTVLEHLQIGDDGEIRTKIILDRENEDSYDFIAITMTTSIVISVQVLVTDANDNPPRFTHEVSYIEISEATPIGAKRSIDEARDADEGIFATQTYEIVSGNVDSAFRLDFREGGGVRDRVLYLDLVVNNYLDRETIPRYELVIHAIDGGVPAKTGSTLLNVTITDANDNQPVFNQSRYSARIAETASVGRSILQVHATDIDEGSNGAVSYDINRRQSDPEEYFRVNSQTGVLYLNKELNYEDKSVHELIIEARDNGTYPERSTAFVTVHVTNVNENPPNVEILFLGNGGLPYVSENAIPGDYIARITVIDPDEGELTNASISLDGGDGKFGIEEVDNIIYLVCVEQPLDREEQSSYDIKITAQDHGLPPLRTEKIVTITITDINDNAPHFDQSVYYASVIEVADPGTSVFQLVGMLHIMAQDGGNPPLSTNVTVIVTIRDVNDNEPSFDNSFYNASVFEDVSTDYCVLQVAATDPDADEFGRITYSMGHVFGISPPAEFSIRPETGWICATMSLDRDRGTTSFLFPVQATDGGGLHSVTMVNINILDVNDNKPIFYPHIYAVDLSEDSAVGTEVISVAATDRDAGAYGLITYQITSGNENNIFTINSRSGMIRLQLSLNRQLQSLHQLTISATDGGGLQSLIDADVSISVISSEDSPPIFDHPIYSYSVSEDSPRFQSISTVHAAHADPDNRDSITYTIHSGDPDGYFSLHPTTGVLSINSALDYESHVYVLLNIQAASGNPPTHGRAQVNISIIDVNDNFPEFARASEVVSVSETITPGTILPFIPVATDRDSGDNGKVEYRLVDSNNMNNFDGLFSINKLTGQIQIHRTVEYSEMSEYELIIEAYDKGHPSLSSVMTLKVYIQDMNNNGPQFYPTSYEVDVAESLVVGERIVTVSATDGDLGNNARITYTLRASQYAGNFGIFPDTGVLYTKKMLDRELVPDYVLEVIAKDNGDPVQSATATVIIHVTDVNDNNPRLLHESYHFTINENEIAYSYVDTVSASDRDQGTNGEIVYSIQPDDTFSIDEQTGMISTNMMLDREANFMYTLTVTATDLGTPARQDTSVVYINVLDENDNPPEFNHKGDYVANVREDEPPNSAVIQVTAKDPDSGSNGVVSYDLVAGGIFNNQVMFTIDETNGMIRTRAVLDRESKSSYHLTVMARDHGNPPKEAVAFVRVEVKDENDNSPKFMNSTYAFTVQENTPPSTNIGQVIAADRDAGNNGAVRYYIIDGDIYGTFEINHTTGNIFTVKYVDYELAASHVLTILASDMNILHPLSTTTTVHIYITDLNDNAPEFANDPIIFPLRENVAIGHVAYTFSAVDADSGWNGKIRYNISGHSGKHIMFEIHPETGELKTTDYINREEYSEYTLVIEAMDLPLEPQQPLIGTTTARIIIEDENDNSPLFVSRSYTYVMEDEPLGYPIMHINALDPDFGDNGRVVYNIVDGNEEGKFTIEHSTGILSLVDPLDREQRDAYGLNISASDHGVLPRVAFQYLIIYLEDVNDNAPVFTSDMFVMDVQENQDAFIYLGSVNATDADIDVNGQVLYSIPNGIANDMFWINADSGAIYTNGALDRETLGSYAVTVYANDQAFPSLFDTTLVLVNVLDMNDHAPVFAMSTYSISVPENGQTTRLHVVVATDADTGTNAEIRYSITGGNEGNRFVIDPITGEFSTQITLDRETKHHYELEITAEDMATNPLTGTMNITVDVVDLNDNAPLFENLPYTVSIPEDFAVNGTVLTVVATDADLGTNAGVNYTLENLMERVFRIDCKTGDVTSVRRFDRESQAEYFFHVNATDESVSNPLSSQAPIHIIITDINDNAPEFTQEPFTANISNDLPVDSSVLQISSSDSDEGVNAEVTYSLLGTSSYFKVESNGIVKSIATINAQSMHRIDVRVTDNGVPSLSTDGYVLIHVDDATPPIEFQQGLYERHITEHAVYNLVVINMRSEVVNPVGGIRFAIEHGNDAFEIEEDSGIVTVKDPSLLDRETTPHITLTISASVNTDRYGFTHLKIILDDINDNPPIFIHERYSAQVWENELRNTYVTQVIATDADIGDNAVITYSITKSEPDEQDFIIDSGSGVILTRFILDWEIHKTYKLTIKAVDGSGSNALTSSCVVRVKVIDTNDNSPTFAEHNGVKIKEGKYYIFNVAVPELTPTGVSILTVNATDRDSGLNARIIYSIDVNPGIGFTIDPHTGILFTNDTIRVQSNPHVVHLGISATDHGEPALSSLVSVRIEVTDVNDNSPMFIPSVYEASVSEAVTKGHNVTRVHATDADYSRENNQVDYSIISGNIDNMFEISASSGQIAVLGDLDRETLALYTLIVQAADRGEPQQSNVATVNIEVEDFNDHTPEFNEEEYEGTVSEGVPVGTTILTVHATDRDIGLNAMIRYDISSGNELDLFKIDQSTGVITSKSRLDHDTQQSVHRLSIIAMDSGIDHQLQAVTSVIIHVTDENDHAPFSPALMYVKTLAEEEPIGTYVFTAHAVDNDGGRYGVLTYTLEDSPGRNYFEIDAESGEVTSLKKFDYETDQWEYLLSIKASDIGGKYVTVQARIEITGVDEFAPEFSSSDYSFLVPANAEPGYTVGQVSAIDKDAGLDGLVLYYFDKELDMFTINETHGYITVKDSLEVKTQRDVSSVREARATNEADMYQFNVVARSGKPNSLSASTKSDITLDYDCDTCPGALSSSETSLSGLPLALVIVFAIIAAISVIVGLIGFLIWYRRKRRPLPPPLQTTDGTRSVNSYDASFDPVMLTAGSTIEQHLISGPNGVVPVYTMNGTRKPQRMDSPHTSMEVGRTDVSDQSNSASSGRGSATVEDEEIRMINEKPIVPDHHGNNQLICHLPPDSGIPQDEDAMSDDMSVNERSANEVLKSLGVFNESYSKGENFNKILNQSVESMHVFGEEGGGEERGAFINSKLHDSGEDTAIINGTRAFGMGDDGLPSMAGSLSSIVNSDEEFSGSYSWDYLLDWGPQFQPLANVFAEIAKLKDESIAKKQLEEHKPRHKQSLSPKVKNFPPPLLTTVVQGPLTPVQPVAINRSRINQGATLPMMPRSPITYDHSFSSPAMSPNFSPSLSPLANRTPSISPLVTPLSSTVNSNTSTPQRSPRHSTIITLPALAGVDEVTI

>BfDCHS2

MSRRNSRTDMVPPWTLLACLFLLLHPANSQSHEATFQTEEGQAVGTYIGNIVEASGITGPFYLSPDPSDT

QLLQHLSIDEHTGVIRSGISLDRETRDFYELLAVSTSQGQVVTVKVQVLDRNDNNPIFPQSLYQLELSES

TPVGVKRNIDSAQDADAGVLGVQRYEINSGNIGNAFRLDARRGGDGVLYPELVVSNPLDKETTPEYNLII

YAYDGGTPPRSGSTTVRVTVGDVNDNQPIFTQSRYSAEVLENATVGTSVLDVTASDQDEGQNGVIIYGID

RRQSDTEEFFAINSETGVISVNKPLDFEDRSVHELIVYARDNGTQPERTTAFVSVNVLDVNDNLPTIDIT

FLNENQSASVSEGAEPGDFVARISVSDSDNGENVNVNVTLEGGDGRFGLTTQDNIIYLVVVARPLDREVV

PNYQIRVIAQDSGAPPLTVDRTINLQVTDINDNAPEFVGSYAPSLSEAAPIGTSVFQLTARDRDIGVNAE

ITFSILSTPGTRSDWFTVDPASGLITTRAIVDREIDPSPRITIDATDHGTPAMSTTIQVTVTLLDFNDNE

PIFNPTFYNATVLEDEDVGHCIVQVSASDPDEGVNGQVTYSLPPTPIARQLPFQIQPATGWICMTQALDH

DTEPSRYDFSVTAKDGGNLDATAWVSIFIQDVNDNHPEFYPLSYPADVQENSPVGLEVVTVSASDRDSGR

FARLTYRIVSGNGGGKFAINANTDFDFESGVVIPTPKIVAGIEGRSFMIDIAQEGLITVAGPLNSGVQSVHNLVVSATDGGGLVSQVNANVAVSVIGTGEMPPVFSQARYSFSVPEDAARQHTVGTVMASHSDPGKTDPITYSIHSGDRQGYFAIDPTTGRIYTQLNLDHETVSSLLLNIQASSGNPPVYGRAQVNITILDINDNRPEFTVATESVAITENVSPGTIIFVASAQDQDAGSNGLVRYSLLNNQDNLFSVNSISGEVKILRSVDADDDNPLRYELKIMAHDQGTPQRFSNLTLLVIIQDENDNGPMFSPTFYDVQVPESASVNYRFLQVTALDRDSGLNEFITYYLSNSADSPNFGMFPDGWLYVKHRLDRELRDSYFLQVVARDNGSPPRNATANVRVTVTDDNDNDPRFTQESYHFSLVENLPTGTTVGTVFAVDADIGNNGDLDFSIIPNNSSFIITTGHQRGVIKTKRPLDRETTARYDLVLKVRDRGTPPRTATATLHIVVEDMNDNAPYFSHSGQYLGEVEEHQPNGTEVIRVLADDPDNGENGTITYRLVTSTPGMNGEEMFNIHSQSGLITTNAPLDREQKPTYVLSVAAVDGGTPPRERTTFVHIHVTDSNDNAPVFVNSTISIEVEEGLEAGSTIGSVHALDNDEGENGRVSYEIMQGNLYGTFGVDRNTGRIFTAKELDFELNARYRLIVQAQDNNARSQKSSTIYVNVNVIDINDNAPTFRSDPVMFGLQENTAVNSTVWTFSATDVDSGRNGAVRYRIMDQLPNGNNFRIDSVTGALQTTTPIDREQTSQFTLVVEATDQPVNLSHARTKTTTARILVEDVNDNTPVFVSRTETYVMEDEPVGYHVMYVIAVDDDFGDNGRVTYQIVSGNQGGKFLLDPNTGLLAIERRLDRETESRYVLNITATDHGTPSRSASHLITIHVRDVNDNQPRFLQDTYQASVSENQSPGTSVIQITALDADAGTNGVLTYNIPRGVAEDRFTIEEQTGVIRTAFRLDREEKDSYIVTAYARDGAYPSRFGFTSVVVSVLDTNDHAPVFKDAEYMMTVPENQPNYGVIHTVVAYDADIGTNGQVRYEIIDGNVGGKFAVDAVTGELSVISDQALDRETVPLYHLIVQAHDNTDAPRTSTTNITVAVSDENDNNPIFTDPSYQQTIQEDVSVGSTVIRVTAQDRDEGVNGEVFYFLSNETNGMFRIDNTSGIITTTRLLDREKQSVYSFDAYASDRGPFGPRTSNVRVTVDISDVNDNAPVFTQVPFETTIERNIGVNQQVVTVTAEDKDTGSNAEILYRFDSSNANSQKFNIGPQSGIITTQVSLVGDSANIFRLQVIAEDQGSPAKSSTGLVVITMSDSSMTTLRFDNGTYSAYISENAIANTDIISVSASREDGQPASVTYSFASGNDDESFEINPTSGLITVKDSTNLDFETAQRVRLIVTAQATTPSLYGYATVWVNLRDENDNAPRFDQDRYTTSVWEGNDRGMFVIQVSATDADSGTNSEVQYSIVSGNHDNAFVIDRDRGIISTNVRMDREVRDSYRLELRAVDKGTPRLTGSATLRISVVDINDNRPTFPEPYPPVNVLEGAEVGSELALVTANDIDTDPTITYTFTTHGNPGGVFSIDRYSGKVTLAQPLDRESVQSYIITIMASDEVHTDQTNVEINVLDENDNPPVFDQQTYQVDIAEMVPPHYSVATVTATDRDAGSNAEITYSMTVAPVEGFYVDPRNGTIFTNKTIEINRMQSTIQLVIEARDHGVPSLASVVAVRIQVNDINNHAPQFTAGQYEEHVSEAAPRGTTVATVTATDADQSHDNSNIHFGIVQGNEDGKFQIETIEPSPNSDQYMGKIILFQAIDREEVPMYTLRVLASDRGTPERNSTVTVYVLVDDVNDHSPIFNSTQYVAQISEEAVIGAFVVRVFAFDADSGPNADIHYEITSGNDDSLFAINSQSGVITVANPLDYDTVAQHNLTIRAKDSTSDGAKHTITSVLINLIDENDNPPDFPVLMYLEDVPEGRPVGTLVFTAEARDKDAGIYGTLSYVILDRDVTNDGTSDVAQGKDAFEIDSSTGEVRTREVFDYETQNTYGFYIRATDTGGLSTDVQVGVSIQSVDEFPPVFTQDNYIFTVIANADVGTFVGQVEATDADGGPDGIVSYSIRNDKFAINESGVITVKASLLEGNTVTDPSRRKRRQAENEDQINLIVEASSGRPGSLSEQKAISVKIDRSCPGCNPSAQSALSPLTPLSLALILIFALIAVILCVVLIVLKFKRRKQRPPPTSFDGSFDTVVVQNTHSFGRDGSQKQSQQLRMFKGHPSFATADNSADMGTLTRTDISEHSNNSQSSGRGSSEVDEDEEIRKINETPASISPNSDQHKVREIPDSGIQHDEDAMSEMSVKDTADLISRLAVTSNQGSQINTMISKSVESMHVFRDEGGGEAGGDMDIGNLIHQKLHEVGMEENEAIMDGTRDFVLIDDGQPSVAGSLSSIVASEEELRGSYNWDYLLDWGPQYQPMADVFLEIAKLKDETVAKRQIHHPKSAFTPKVRTHPPPLITNLPQSSVSTIAPIALGGSSRTSQATSISSLPRSPISHESTFTSPALTPSFSPSLSPLATRSPSVSPLVTGPNSGASTPHRRSAYSSTFMTRVPSPTGSEQELRI

>MmDCHS2

MSPAGRRMGEGRQPAGSPRGRPRGAGAQSSLLRLFVHAWLWAASGSSAQVFNLSLSVDEGLPPDTLVGDIRAGLPAAQQQDGNGFFLSEDSDDSPLLDDFHVHPDTGIIRTARRLDRERQDHYSFVAATLLGEVVQVEIRVNDVNDHSPRFPRDSLQLDVSELSPPGTAFRLPGAQDPDAGLFSIQGYTLLQASDMPQDPTGPFFQLRYGTPGLPASPSLPVSSSPLEPLDLVLLRRLDREAAAAHELHIEAWDGGSPRRTGLLHVQLRVLDENDNPPVFEQGEYRATVREDAQPGSEVCRVRATDRDLGPNGLVRYSIRERQVPVASAGGGPLGDPGYFSVEELSGVVRVQRPLDREEQAWHQLVVQARDGGAEPEVATVRVSIDVLDVNDNPPAIHLLFLTEGGAVQVSEGAHPGDYVARVSVSDADGDPEKEEEAAGVLGARLLGAGSIKLSLESGNGVFALRPGGPPGVFFLCIEGLLDRESQDLYELRLVATDAGSPPLSTEESLLLWVSDLNDQPPVFSQEHYWASVSEAAVPGTSVVWVSALDADQAGTDHAKLRYELVQLSDPCQSEALSPEEECVPSFSINPDNGLISTIRALDREVQETVELRVVAQDLGEPPLSATCLVTITVDDVNDNEPVFRRQVYNVTLAEHAAVGHCFLQVKASDADAGLYGLVKYSLYDGFQSYEAPPAFQIDPQDGRICVSQDIDRERDPGTFDLLVKAKDGGGLSAQAFVRVEVDDVNDNYPVFTPSTYVTSISGQTPPGTEIINVLASDRDSGIYGTVAYELIPGDQSSLFTIDSTTGIIYLTSTLSHLEATTIFLMVCARDGGGLTAATNADVTIHIMQTTLAPAEFERPKYTFSVYEDVPEDTLVGTVKARESLNSSEPITYRISSGDPEGKFSIHRWLGSIRTLKPLDHEAQPMVVLTVQAQLGSSPACSSTEVNITVMDVNDNRPEFPTASDEIRISQTTPPGTALYLARAQDRDSGLNGLVRYSIASPQPSEFSMDQGRGVLYLRESLGSKADFRLILVAKDQGVPPQVSQLVLTVVIESQERIPAVAFENLVYQVEVSESLPLTTQILQVQAYPLYPWRPTSKTFYSLDVSVDSAVFGIHPHTGWIYLRRQLDYEFTQTYKFRVYVHTSEDRLLQNVSTSVIVHVLDENDHSPAFLQNRVFLNVEESPIPLGVIGKMTAIDADSGKNGQLSYFLLTDGKFFKMNPNTGELINWLALDREHQGHHQITVLVTDHGSPPRNATMLVYVTITDINDNWPFFPQCLPGKEFHFKVLEGQPVNTLVTTVFAKDLDEGLSAELTYSISSDYPAHFKIDANNGEIRTTSILSHDYRPSYRMTVIASDHGVPPLQGKAIINIQVIPLSKGRVLMSQNIRHLVIPENTKPSKIMSLMKSPDPLQQDHGGKLHFSIAAEDKDDHFEIDSSTGDLFLTKELDYEMTSHYLIRVISKDHSQSPAWNSTVFLSIDVEDQNEHSPSFQDEFIVISIEENVPVGTLVYVFNAKDGDGSFLNSRIQYFAESSSVGVNPFLIHPSSGALVTASPLDRENVPTFILTVTASDQAVNVTDRRWRTLVAEVVILDVNDHSPTFVSYPITYVREDAEVGAVVHRITAQDPDAEMNGEVAYSILSGNEDMVFVLDSSSGLLRIACPLDYEVKTQHILTLVAHDGGMPARSSSQTLTITVLDVNDETPAFKQLLYETSVKENQSPGVFVTRVEAEDTDSGVNSKHQFEIMPGPAFGLFEINPDTGEVVTAVTFDREAQGIFRLRVLVRDGGVPSLSSTADIICTIEDENDHAPEFIVLHHDIEILENRDPEVVYTVLAFDMDAGNNGAVTYHIAEGNTDEYFAIHTTSGELSTTRALDRELISNFTLTILCSDLGNPPRSSAMQLHVRVLDDNDHSPAFPMLHYQSSIREDAEVGTVVLVLSAVDRDEGLNGQVEYFLMEEVSGAFTIDRVTGILRTSHALDRESRSQHTFQAVARDCSTQGAKSSVLSILISVTDANDNDPVWEENPVDAFISPMLALNQTVVHLRASDPDAGPNGTVTFSFADRQSVFSIDGYTGEVKLQQNLSSEHFPIWLQLLATDQGTPARTTMGLLVVHKEGEGMKLSFSRYLYTGLVTENCEPGTSVVTVKAFAPVSSPDAITYSVVSGNEDGVFSLGSNSGQLIVEEPGLLDFEVRSEVRLIILAESNGHQAFTQVTVAIQDWNDNPPRFAQSVYQASVSEGQFYSVHVIQVSATDLDQGLNSQIEYSIVSGNQAGAFRIDELNGVILTNSILDYESSGSYSLIVQATDRGVPRLSGTALVKIQVTDINDNAPVFLPSEAVEIAENSLPGVIVARVSVHDADLNPAFTFSLVKESSSAAKFAISQDTGVVVLAQTLDFEEVTEYELIVRVSDSVHHTEGSVIIRVLDVNDNPPVFTQDFYQAAVPELTPGGYLVLTLSATDLESSGDISYRILSPPEGFTIDPRNGTIFTTNSVSVLEKIPTLRFLVEANDGGIPSLTALTLVEIEIQDVNNYAPEFPAGCYNLSLSEDTPIGSTLMTFSTIDGDYSFENTHTEYSIISGNLHNYFHIETSLLGSEHPHQQRGALVLLHALDREASASHKLVILASDHGCPPLSSTSVIAIDILDINDNAPTFSSRHYQAHVKESTPVGSHITMVSADDPDKGSHAEIIYGIISGNEKEHFYLEDRTGVLYLVKPLDYEETVAFTLTIQATDEEEKHVSFAAVHISVLDDNDHSPQFLSSTLACITPENLPPLSIICSVHALDFDTGPYGEVTYSIVSPCLVTHGMHPYQDLFAIDPLTGDIHTEQMLDYESVREYCLLVQAKDRGDASASLEVWVEVEGIDEFEPIFTQDQYFFSLREKGQGQQLIGRVEASDADAGVDGEVLYSLRTPSTVFSVNKTNGNIYWVRAPLLGSSQLVKEDTLEVKIIAHSPKPGSKSTSCSVFVNVSLPAEGHHRTVLVHSFSISLVVSLLVFLSLVCTLIVLILRHKQKDPLHSYEEKKTPSSPDADPKLTGAASELKAGQETAEYRGVTGPGEVMPAEWLNLMSVMEKDIIHLFRHSNYSGHCSVDGETAEDKEIQRINENPYRKDSDYALSDQGSRVPDSGIPRDSDQLSCLSGETDVMTSSEVMEASHMFEEGVGGEGCDVIYVQNNALSLRREATAGVLAESRRESFTSGSQEGRCVAPSTQMTSSDDVRGSYAWDYFLSWEPKFQHLASVFNDIARLKDEHMQVPGIPKDTSFVFPPPLITAVAQPGIKAVPPRMPAITLGQVLPKFPRSPLPYHGGSLPEVMTPNFSPSLSLLTMQTPARSPMLPDGESRGTHMLGPWHDRKAEDEVQG

>CgDCHS2

MKFWGSVRVLLLLSSVAAVYGEVVLKFDVVEEQDPLTYIGQINVSAYPPPYTVMQGDEGISLDTQSGRIQTARRLDRETKSEHTVALFSTGKGFVAIQIQVNVTDINDNAPYFPNGSKSLSLSEISPNGSKVHIGSVIDRDIGVNTVKPPAVIISGNQENMFKLETKTTGSQNTLYLDLVLNGKLDYDNGVKKYSLVIQVSDGGTPQRSTDLTVTVNVLDANDNAPEFTHTKYSTVIPENILIGASIVKVSATDIDSGENGHITYLLDRQRDPEEHFVIEPNTGIIRINKQVDYEHQKHYELSVIARDNGSQTLESNAVVEVNITNVNEQPANINLVYLIQDNKTYENATVGGFIARISVSDPDSPNNYFANVNVTLQGDMGYFGLVTDDKVVYRIKILKPLDRETYSNYNLTITAEDSGPGGPPLYATKSFTLIVDDVNDNAPKFNQTVYTASVPEKAPAGTSVVNVMATDPDLGENARVTYSIISNPSSDSDWFEISVTTGFITTKSTANIDCEHNSNPWITVIATDHGSPPLSSTATVSITIDDVNDLEPVFERSYYEAKVPENVAVGSCILAVSANDPDCQGRTQNLVRYHLDNSTASRPFTGRSYQWTNLCQQSSMAGDSSNTFKLNSTSGEITLMRSLDYEQVQLYKLYIEARDQGNPPRTSTLSLTISVLNVNDNSPVFSASSYVFNVSESALVQSNIDQVQATDRDGDHVSYSFRDSMYLNLFGIGSSSGFIYIKQELNREEKDKYTLTVVARDSGKGYRSSSVPVTINVLDANDHSPVFKQSFYTFYILENMAAGTVVGQTTATDKDTGENAYLRYQFEQPEPHLKIDSDTGVITTTVSLDREEKNFYNLTVVASDHGDPKKFDYTNVKIFVGDLNDNSPKFLNSQPIEATVNENEPKGTSVLNISAKDDDALENGTISYSLIADGDANVLKYFAIHPKTGLVTTLEVLDFEQKNRYALGVIAKDHGNPAKSSSSSLVVHVKDENDGRPIFSSQNLTIQVVENIKTGSIVGKVEAKDWDSGENGRVSYSIIGGNVFDVFAVNVSNGNIYCIRNVDYEEASSHSLAVKAVDNSPYNPKSSTINVIVEVVDVNDNPPVFEKDPVLISRKENLPTGYTVHTFTATDKDSGVNGSVRYSIQSQTPDRALFTIDPISGDLKVASILDYEEVKQVSLVIQAKDQCHAGCQQKATITAWLSVLDVNDNTPVFKGNSSYSVFENETVGPQGFPVTHIIATDADSNVDESGNGVLAFSIVDGNEEGHFAIDRSSGLLSIQTTLDRETTPSFTLAIQVRDQGTPQRSAIKLISIHVLDINDHAPLFSQSSYTVDIQENLPPQSNVLQVQATDLDIGVNGKLSYYLPPGVGGDKFVLDSTTGQLSTTASLDREAQASYTLTAYVRDFGYPERYSSATIVVRVTDVNDNLPMFVKDEVSLSIPENTEQRAIHQVVAKDLDTGDNARLSYTIISGNVGSAFTIDPETGQLSCKSLDRETVSSYNLTIRASDHGSPQRNTMCRVFVKVLDKNDNYPQFSALEYAHTIKENVVVGTFVLQVSASDIDEGENAHITYSLGNDTDGLFQVDSQSGNITTNGLFDYEKKTSYVFFVVAKDGGQKDIKNKTVQVRINLSDVNDNAPVFTEMAYHKNLSVNTASDTLVVRVKADDRDSGINGEVRYSLSNSGTDLATYNMFRIDAVTGDLYTKQTLTTSGVRMIQVVANDLGTPELNASGIVEVTVGGGSGDLSLRFDQASYHVMIRENPSRGTPVTTVRATFVGSGSGPITYSLVNSNDQLVFSLGASTGTITINNGATLDFEVAQEVHVMVVATSGGLSTYTRLVVQLTDVNDNAPKFAQNVYYSSTWEGEEGDQVYVTQVLAVDADSNSNADIMYNIIGGNEGFTFQIYPPHSGIVVTTLPLDYEIQDSYSLTIEAVDHGQPPLSSTCTLNIAVVDINDQRPKFPDPMPVNLSEAADVGSLVRLITANDRDRNPTLLYDFTPNGNPGNTFTIDRFSGRITLAKPLDHEVRKHYAVGLSVNDTKHSAQTILEVYVEDENDNHPVFSQQSYQADIPEFTGSGVKILTVTATDADSGINSVLTYNLIVNPTGGFYIDPNTGELFTNKTVELRQDQQVITLVVTAQDGGRPRLSAVVAINLQVIPVNHYAPEFPPQQSKFIFAEDIKKGENLWSVTATDRDMKQEIYYSIKAGNQHNTFGIDRQSGLVFLNENLDRELVPTYSLIIEASDNGNPFKTNTLVVSVEVRDVNDQRPVFQPTMYFKELPENTAHSNSFLQVHATDDDEGVNAEIQYTITSWECTQIRFSAFATIVINVTDVNEYPPVFPVPFYYASVNEGPTGTKVFQAHANDKDGGPYGVLSYQIKNTVVFKIDSVTGWVSTNMNIDYENLPYLSDPGQNQYRFDLAVTDPMGLEDTKPVIVTINDVDEFAPVFTQNKYAFEVPGNCQTRSCDRQATNGVENVMESTSIVEVSIDTKCAGCALAQSQGEPDEPAIILVVIIVIIVIRFRKRKTPTVVYETEYPNQFDFPVANDDGSPPAYDEKYRNHPITPDISERSHHSQSSGRGSVEADEDEEIMMINSHSSVLNNSSGFRSKNMPDSGIQDDDNTSEPSVQNSKDYLARLGIEPVHTQIKTQNIMQSVESMHQFTEEGGGEDVEYNNDSSADIVISDSNNDLGFHEPVEMQQHVGSLSSVINSEEEYSGSYNWDYLLDWGPQYQPLAHVFTEIARLKDDRQTPKKQPVKTVPQRKQNINLNPQVKMDPPPIITNAPPKAVQHGQPQNSQGSKHSSRTNSTMNVSSLPSLPRSPISYESSFTSPAITPSYTPSLTPLATRSPSISPYGSGHNTPNRQRGNGHRQMMALSSESEQELRI

>DmDCHS2

MLRSSLLILLAIVLLGSSQAASHDQERERKLEVFEGVAVDYQIGYIGDFGGIDSGPPYIIVAEAGVETDLAIDRATGEIRTKVKLDRETRASYSLVAIPLSGRNIRVLVTVKDENDNAPTFPQTSMHIEFPENTPREVKRTLLPARDLDLEPYNTQRYNIVSGNVNDAFRLSSHRERDGVLYLDLQISGFLDRETTPGYSLLIEALDGGTPPLRGFMTVNITIQDVNDNQPIFNQSRYFATVPENATVGTSVLQVYASDTDADENGLVEYAINRRQSDKEQMFRIDPRTGAIYINKALDFETKELHELVVVAKDHGEQPLETTAFVSIRVTDVNDNQPTINVIFLSDDASPKISESAQPGEFVARISVHDPDSKTEYANVNVTLNGGDGHFALTTRDNSIYLVIVHLPLDREIVSNYTLSVVATDKGTPPLHASKSIFLRITDVNDNPPEFEQDLYHANVMEVADPGTSVLQVLAHDRDEGLNSALTYSLAETPETHAQWFQIDPQTGLITTRSHIDCETEPVPQLTVVARDGGVPPLSSTATVLVTIHDVNDNEPIFDQSFYNVSVAENEPVGRCILKVSASDPDCGVNAMVNYTIGEGFKHLTEFEVRSASGEICIAGELDFERRSSYEFPVLATDRGGLSTTAMIKMQLTDVNDNRPVFYPREYKVSLRESPKASSQASSTPIVAVVATDPDYGNFGQVSYRIVAGNEAGIFRIDRSTGEIFVVRPDMLSVRTQPMHMLNISATDGGNLRSNADAVVFLSIIDAMQRPPIFEKARYNYYVKEDIPRGTVVGSVIAASGDVAHRSPVRYSIYSGDPDGYFSIETNSGNIRIAKPLDHEAKSQVLLNIQATLGEPPVYGHTQVNIEVEDVNDNAPEFEASMVRISVPESAELGAPLYAAHAHDKDSGSSGQVTYSLVKESGKGLFAIDARSGHLILSQHLDYESSQRHTLIVTATDGGVPSLSTNLTILVDVQDVNDNPPVFEKDEYSVNVSESRSINAQIIQVNASDLDTGNNARITYRIVDAGVDNVTNSISSSDVSQHFGIFPNSGWIYLRAPLDRETRDRYQLTILATDNGTPAAHAKTRVIVRVLDANDNDPKFQKSKYEFRIEENLRRGSVVGVVTASDLDLGENAAIRYSLLPINSSFQVHPVTGEISTREPLDRELRELYDLVVEARDQGTPVRSARVPVRIHVSDVNDNAPEIADPQEDVVSVREEQPPGTEVVRVRAVDRDHGQNASITYSIVKGRDSDGHGLFSIDPTSGVIRTRVVLDHEERSIYRLGVAASDGGNPPRETVRMLRVEVLDLNDNRPTFTSSSLVFRVREDAALGHVVGSISPIERPADVVRNSVEESFEDLRVTYTLNPLTKDLIEAAFDIDRHSGNLVVARLLDREVQSEFRLEIRALDTTASNNPQSSAITVKIEVADVNDNAPEWPQDPIDLQVSEATPVGTIIHNFTATDADTGTNGDLQYRLIRYFPQLNESQEQAMSLFSMDSLTGALSLQAPLDFEAVQEYLLIVQALDQSSNVTERLQTSVTVRLRILDANDHAPHFVSPNSSGGKTASLFISDATRIGEVVAHIVAVDEDSGDNGQLTYEITGGNGEGRFRINSQTGIIELVKSLPPATEDVEKGGRFNLIISAKDHGQPEPKKSSLNLHLIVQGSHNNPPRFLQAVYRATILENVPSGSFVLQVTAKPLHGAENANLSYEIPAGVANDLFHVDWQRGIITTRGQFDRESQASYVLPVYVRDANRQSTLSSSAVRKQRSSDSIGDTSNGQHFDVATIYITVGDVNDNSPEFRPGSCYGLSIPENSEPGVIHTVVASDLDEGPNADLIYSITGGNLGNKFSIDSSSGELSARPLDREQHSRYTLQIQASDRGQPKSRQGHCNITIFVEDQNDNAPRFKLSKYTGSVQEDAPLGTSVVQISAVDADLGVNARLVYSLANETQWQFAIDGQSGLITTVGKLDRELQASYNFMVLATDGGRYEVRSATVPVQINVLDINDNRPIFERYPYIGQVPALIQPGQTLLKVQAIDADLGANAEIVYSLNAENSAVSAKFRINPSTGALSASQSLASESGKLLHLEVVARDKGNPPQSSLGLIELLIGEAPQGTPVLRFQNETYRVMLKENSPSGTRLLQVVALRSDGRRQKVQFSFGAGNEDGILSLDSLSGEIRVNKPHLLDYDRFSTPSMSALSRGRALHYEEEIDESSEEDANNSTRSQRALTSSSFALTNSQPNEIRVVLVARTADAPFLASYAELVIELEDENDNSPKFSQKQFVATVSEGNNKGTFVSQVHAFDSDAGSNARLRYHIVDGNHDNAFVIEPAFSGIVRTNIVLDREIRDIYKLKIIATDEGVPQMTGTATIRVQIVDVNDNQPTFPPNNLVTVSEATELGAVITSISANDVDTYPALTYRLGAESTVDIENMSIFALDRYSGKLVLKRRLDYELQQEYELDVIASDAAHEARTVLTVRVNDENDNAPVFLAQQPPAYFAILPAISEISESLSVDFDLLTVNATDADSEGNNSKVIYIIEPAQEGFTVHPSNGVVSVNMSRLQPAVSSSGDYFVRIIAKDAGKPALKSSTLLRVQANDNGSGRSQFLQNQYRAQISEAAPLGSVVLQLGQDALDQSLAIIAGNEESAFELLQSKAIVLVKPLDRERNDLYKLRLVLSHPHGPPLISSLNSSSGISVIITILDANDNFPIFDRSAKYEAEISELAPLRYSIAQLQAIDADQENTPNSEVVYDITSGNDEHMFTIDLVTGVLFVNNRLDYDSGAKSYELIIRACDSHHQRPLCSLQPFRLELHDENDNEPKFPLTEYVHFLAENEPVGSSVFRAHASDLDKGPFGQLNYSIGPAPSDESSWKMFQVDSESGLVTSAFVFDYEQRQRYDMELLASDMGGKKASVAVRVEIESRDEFTPQFTERTYRFVLPAAVALPQGYVVGQVTATDSDSGPDGRVVYQLSAPHSHFKVNRSSGAVLIKRKLKLDGDGDGNLYMDGRDISLVISASSGRHNSLSSMAVVEIALDPLAHPGTNLASAGGSSGGSIGDWAIGLLVAFLLVLCAAAGIFLFIHMRSRKPRNAVKPHLATDNAGVGNTNSYVDPSAFDTIPIRGSISGGAAGAASGQFAPPKYDEIPPFGAHAGSSGAATTSELSGSEQSGSSGRGSAEDDGEDEEIRMINEGPLHHRNGGAGAGSDDGRISDISVQNTQEYLARLGIVDHDPSGAGGGASSMAGSSHPMHLYHDDDATARSDITNLIYAKLNDVTGAGSEIGSSADDAGTTAGSIGTIGTAITHGHGVMSSYGEVPVPVPVVVGGSNVGGSLSSIVHSEEELTGSYNWDYLLDWGPQYQPLAHVFSEIARLKDDTLSEHSGSGASSSAKSKHSSSHSSAGAGSVVLKPPPSAPPTHIPPPLLTNVAPRAINLPMRLPPHLSLAPAHLPRSPIGHEASGSFSTSSAMSPSFSPSLSPLATRSPSISPLGAGPPTHLPHVSLPRHGHAPQPSQRGNVGTRM

>SpFat1

MAGVRWCLGRGTLTFLFLVLQLGCTIVLGQDIQFTRSIYNATIAENSPIRTFISTEQKMGVYLPDPSATVRYTLQESDVSDLFSARPEVVGDFAFLRIRTRPCRNKSQCPNLKVNREVRPEYYLTAIAQSSSGGVRSTSTAEVHIVVQDVNEQPPLFLRGNYAATISEDYPVFSNIIQVEAHDSDSGTNGEVYYSFRQQTDQFAIHTTTGVVSLTRPLNFDQQSSYTLTVVAKDRGFTPYGGLPLTSPVQLRIYVQQVNKHSPEIRIEKQPTVSRNSEIGTVFAVVGVTDEDGGVNGEIAALDIIDGDPGGFFRVQPSPSPGRYQLQIARSLLSSSLPHAFNVTVAASDRGLSPKSTSLVVGVVLDGAMNYEAYFTNDFFNTTINELMPVNTPFLSVAPRSAVGNTGFSYVITDSSLPFSIGTHTGMLSVAVDLDRETTPEYEFTVAVRNAKQQTIADTRVVVILSDANDNNPSFEEEIYTVEVEENRPAHSQVLVVRASDPDLGENGFISYSIANLNAVPFTIDHETGSINTSKVLDYETMRHQYRLRVRASDWGSPFRREAKAVVIVNLINLNDNRPKFEKINCVGAIAKSFPSGRAIALISALDFDENRPRYWITSGNVNDLFSINPQSGNLTLSRPVADSDPLFYSLRIEARDGPTSVSYMRTNMTITNNANPSPQAHARGLKVTCQETDVAEEIEELYGQLEASNQEVEHLPPNYAELYSSNVHEPEFETGFRTSVEVPEDAPVGGTVLTVNANDLDHGFNGKLLYAISDGNDKSHFQMDFETGELKVLMPLDRETQSTYDVTVKISDMGKPQHTRKSPLTITLTDVNDNAPQFDLKQYDVAIPEDAEVGLSFLAVHASDRDEGRNQEVRYSIISDSPSFDIDIITGELSVAGPLDRETNPVHEIRVQATDLSDSPLSSSVIVRVSLDDINDNPPRCLLPEYKVKIREDLPTGAAVLSVQAHDPDNPPNGTVRYRLDTSGSSKFSIDTDYGTLRLVEMLDYEVTQLYEIVVAIRDEGIPAMSSTCRIVVEVVDVNENTHAPVFPNYVLTGSVHEGHINDTEVMTISAADMDMGIDGEVSYTIRDGSGLGRFSIDSSGMGVYVLTVEAVDPDTSSSGNLTYRLKTNSAKKYFTIDERTGVISTASKALDREAKKGEEHHFEVEVSDNGIPSLKSIAKVVVTVEDENDNDPKFIPASTVTIPAMDDPGERRFICRMMATDKDVGSNAELSYFIRDGNTQSRFYIDATTGEVTCSKTLIDGERYVLQIEVKDNGPMRRRSDLARLPIIVTAAPVPSSSAPFFEEMLETETRITENYVVGSFINAILAIDPDGDEVRYAIVDGNEDGAFFIDPQGGFILIAQPLDSETKSSYDLTISASDGFNEATTMIRILVTDVNDNPPILEQELYEIDIPENTRVNTPILQIMATDRDATDRLVFTLPSTSDPASRYRFRIDTHNGYIYLNESLDHESQRQHVLNVEVKDYNYGTHRTYARVVVNVMDSNDHMPEFSAESYEGKVYETAAVGTRVVDVYAFDRDQGSNAELTYSIVQGNIGQAFNIDPILGTVTVGHELDIEQEAEYALVVQAIDHGVQPLSNVCFVNVTVTLSNNAPPRFREEEYIMEVAENLLGNQFVAQISAVSRSSVYYEITRGNDARRFDINSNSGIITTTMPLDYEEVTMYNLTVQATNMVGLRTNTCLVIHVQDANDNAPTFSSVEYVGSISESASLKSVVLDENSRPLVISAEDADSGHNAHLVYEIIERDAQMYFSIDESTGAIRTKMSLDHERIPEFEFTVQVHDTGSPQMMANKPARVRITIIDINDSQPQFVKAVFEAQLLLPTYAGVFILTVTAIDDDDVSMSQLEYSIHEGDRSHHFSIDAQSGDIFVANGTSLSSEYDLTVRVTDGLNFNDAQVLIHVEARPTSSLRFPANECYAIIRENSSTVHDVAVLSVIGSTLNEPLTFTILNPSPMFSIKPTSGIVRNTGIPFDREEQDSYHIVVEARDRRTPPRVAHIIVNVEILDINDNPPIFIHHQYNAIVQVDASVGEVVRQVTAIDRDVSKNGEVQYTLVEGGEGHFAIDSSSGVIIVREKLGANSQNKNFTLKIKASDRGKPNHSTVVDVPITVRNKAMPVFEEQYYRATIPEDLELHSAVVQIHAISPNGRDLIYSIGEGDEFNQFDINPLTGVINLIGAVDYETRQLYGLEVLASDTLTGASAMVTVDITISDVNDVMPSFNQKIYQAVLSEAVSVGATVLQISTTDQDSPKNSGVRYQIVQSDNQTGHFHIAANSGLILTSHVLDYEEHKMHNFVVVATDSGMPPLRSETRVSIRITDMNDNPPQFVHRDYYCSVSEIAERGAFVTSVSATDRDISDIDQLTYSIVSGNEDMTFTINKKTGVISLSNSQKPQLMSGRDYHQLNVSTSDHVFTSSATVHVNITRVNRHSPVFSQDEYVTDFMENGTAGEVVFQVMATDDDSGDNGVVTYSIISAEARRMFDIDSATGEIHSRMLLDLESVSDRTLPVPIMAVDGGGKTAYTTVNVILNDRNDNVPRFEMREYKAFTRSGGESHVPILVVTATDVDSGSNALLTYSFESGTAQSALDLFEIDPKTGSISASSSLEGEEGSTFQFFVGVTDGGNPPLNWVTAAEIYVLSQDDELPFFDGLVNEPDIDVAENTPVGTILSTFLAHSNSSLTYSLVPGNEAHTNDPSHFSIDEHGNLTLVSSLDFESVSWYKLIIKASTNTEPSIATYFTVLVTVTDVNDNMPVFEDTTYSISVPENTPVDDAILKVIARDADAGSNGQVLYYLDVENDLEVFEKFEVNEMSGMLTIQEGLDRETAVTYDILILARDVTNADFSATATVHVTVVDFNDSPPRFTQKVYPGEVLESDPIGTVTVSIQAVDGDVGTNAHVVYYITSGDPFNHFAIESNSGKIYVANPLDRELKDTYRLNVTATDGLFSDTAQVVISVQDINDNSPVCAQTLYTENIRENLAAGHYILYVSASDADIGSNAEITFSLEGQGSELFTIEKDLGVNTGTVRTSGALDYETQQFYRFQVIASDGGGLSCSSDISIGLLDVNDNPPAFTQLVYNVSVSENTTVNTLLTRVQAVDPDKDANRRHRYSVRGGNSEGTFSLDDQSGILTLTEPLDRELRSHYNLTLAVTDILQASLSTSSQLIVNVLDENDNEPQFESDLYNASIAEDAEIGSTVVTVIAISQDVGLNAEIMYQIVSGNEHGKFKIDSAIGEITVASELDFEMSNKYYLTVKATDMGTNPLSDTTTINIFITDANDNAPLFSEDIYSTTINEAARVEGDVIQVMATDADSGANGRVSYSIIHGNLFNQFIINPDNGLVSLAAKLDREQIASYSLGVRASDAGDEPSFTDVTVQIIVDDINDNPPVLPRQNYTIIQQADTPVESDILQLNATDLDTLANGPPFTFEIVRGNDNGAFVITSTKMLKNRIWFNRDIETQYNLTIKVTDSARSPLYSLSYIKIIIVDPMNVPEVVTPVKITISSFEETFPGGWIGQISATDRDPYDDLVFELQNDNVFRIGQEDGIIIAPPDLDEGFYHINASVSDGQFTVYAEANVTVRMLNQAMLNNSATITFADVTPEEFLPNSGIFMYTVANHFPGATPEDVTIITIHPSEENIENTDVVFSIHRSKSRESFFKPKQIQRQVEALSAALQERMKHDVVSAVSDLCTLFSCEESYICSNDVRLDYSNILTFSTDDESFVSSRLYRDYTCTCVVGSCATTTKKPTTTRATTTPALRTPSVINQDNPCASNPCHANMNCRWDGFDGYVCVCTDDSLSCLPGPDEAMNFNGQSYVTYNHLVLSTTSTQFSSSVNTDRPNGIIMDGAGEFDYSVLEIENGYLTYRLNCGSGEAKIRITQKKVNDFGWHKISINREKNHAVLTLDEQYTAEGTAPGENQDLNIDTISIGSSPPAKGRVRRSADVGFTGCMGGLSLDNSPLPLLGDSVQPHNIGKCQRKWPMACSPNPCQNDGTCLDYGYSTTCDCLHNWSGERCEKKNCLDAECEGIPTITSGFPMRLIIIIIGVLVAIIIIVFILAACRYHRNKRRRSRYPARDGMGRSYDDDYKRDSKLSDSDYLQSLPLNPVAATPPSPTPPPLPTRPASYTRSNHNSLNNLDRDRHDDIPFHGQPISMPPSLAPVPSNSASDSDSIAKPTWEFDTPSTHNSFIDGRDSNKGLSPHPLQHSQPSILPPVRRGHNTPMNIRHPVESPPAYRAVNPNQADMVSMSSVNTENEDDGLRAHGLQRGKKD

>LvFat1

MAGVRWHLWKGTMAFLLLVLQLGFSLVLGQDIQFTRSIYNATIAENSPIRTFISTEQKMGIYLPDPSVTVRYSLQESDVSDLFSARPEVVGDFAFLRIRTRPCRNKSQCPNLKVNREVRPEYYLTVIAQSSSGGERSSATAEVHIVVKDVNEQPPLFLRGNYAATVREDYPVFSNIIQVEAHDSDSGINGEVYYSFRQQTDQFAIHTTTGVVSLTRPLDFDQRSSYALTVVAKDRGFTPYGGVALTSSVQLRIYVEQVNKHSPEIRVEKQPTVSRNSEVGTVFAVVGVTDEDGGANGEIASLDIIDGDPGGFFRVEPSSTPGRYQLKIANSLLTSNLPHAFNVTVAASDRGSSPRTTSLVVGVVLDGAMNFEAYFTSDFFNTTISELMPVNTPFLSVAPRSAIGNTGFSYVIQDPSLPFSIGTHTGMLSVAVDLDRETTPEYDFTVAVRNSKQQTIADTRVLVILSDANDNNPSFEEELYTVEVEENRPVNYQVLVVRASDPDHGENGFISYSIANMNPVPFTIDHQTGSINTSKVLDYETMRHQYRLRVRASDWGAPFRREAEAVVVINLINLNDNRPKFEKINCVGAIAKSFPSGRAIALISALDFDDNRPRYWITSGNVNDLFSINTQSGNLTLSRPVADSDPLFYSLRIEARDGPTSVSYMRTNMSITNNANPNPQAHARGLKVTCQETDVAEEIVGLYEQLQASNQEVEHLPPNYAELYSANVHEPEFETGFRTSVAVPEDSPVSSTVLTVNANDLDHGFNGKLLYAISDGNDKSHFQMDFETGELKVLMPLDRETQSVYDVTVKVADMGRPQHTRKSPLTITLTDVNDNAPHFDLKQYDVNIPEDAEVGLRFLAVHASDRDEGRNQEVRYSIISDSPSFDIDIISGELSVAGPLDRETIPIHEVRVQATDLSDSPLSTSVIVRVSLDDINDNPPQCLLPENKVKIREDLPTGAAVLSVQAHDPDNPPNGTVHYRLDNSGPSKFSIDTDYGTLRLVEMLDYEETQLYEIVVAIRDEGIPAMSSTCRIVVEVVDVNENTHAPVFQNYVLTGSVHEGHINDTEVMTITAADMDMGIDGEVSYTIRDGSGLGRFSIDNAGMGVYVLTVEAVDPDTSSSGNLTYRLKTNSAKKYFTIDERTGVISTASKALDREAKRGDEHHFEVEVSDNGSPSLKSIAKVVITIEDENDNDPKFIPASTVTIPAINEPGERRFVSRMMAIDKDVGSNSDLSYFIRDGNTQSRFYIDATTGVVTCSKTLSEGERYVLQIEVKDNGPSRRRSDIARLPIVVTAAPVESSNAPIFDTLMETETRITESDAVGSFINYVFAIDPDGDEVRYAINDGNEDGAFFIDPQGGFILIAQPLDSETKSSYNLTISASDGFNEATTMIHILVTDVNDNPPILEQELYEIDIPENTRVNSPVLQIMATDHDATDRLVFTLPSTSDPASRYRFRIDNHNGVIYLNESLDHESQRQHVLNVEVKDYNYGTHRTYARVVVNVMDSNDHMPEFSAESYEGKVFETAAVGTRVVDVFAFDRDQGSNAELTYSIVQATRHDDLRGNIGQAFNIDPILGTVTVGHELDIEQEAKYSLVVRATDHGVQPLSNVCFVNVTVTLSNNAPPRFREEEYIREVAENLLGNQFVAQISAVSRSSVYYEITRGNDDRRFDINSNSGIITTVMPLDYEEVSMYNLTVQATNMVGLRTNTCLVIHVQDVNDNSPVFSSVEYVGSISESASLKSVVLDEDSRPLVISAEDADSGPNAHLVYEIIERDAQMYFSIDESTGAIRTKMPLDHERIPEFEFSVQVHDTGSPQMMADKPARVRITIIDINDSMPQFVKAVFEAQLLLPTYAGVYVLTVTAVDDDEVSMSQLEYSIHEGDRSHHFSIDPRSGDIFVANGTNLRSDYDLTVRVTDGLNINHAQVLIHVEARPTSSLRFPANECYAIIRENSSTVHDVAVLSVIGSTLNEPLTYTILNPSTMFSIKPTSGIVRNTGIPFDREEQDSYHIVVEARDRRDPPRVAHIIVNVEILDINDNPPIFIHHQYNAIVQVDANVGEVVRQVTAIDRDVSKNGEVQYTLVEGGEGHFAIDSNTGVITVLEKLGANSQNKNFTLKIKASDRGKPNHSTVVNVPITVRNKAMPVFEEQYYRSTIPEDLELHSAVLQIQAISPHGRDLIYSIGEGDEFNQFDINPLTGVINLIGAVDYETRQLYSLEVLASDTLTGASAMVTVDITISDVNDVMPSFNQKIYQAVLSEAVPVGSTVLQISTTDQDSPKNSGVRYQIVQSDNQTGHFHIAANSGLILTSHVLDYEEHKMHNFLVVATDSGMPPLRSETRVSIRITDMNDNPPQFVHRDYYCSVSEIAERGAFVTAVSATDRDISDMDQLTYSIVSGNEDMTFTINKKTGIISLSNSQKPQLMTGRGYHQLNVSTSDHVFTSSATVHINVTRVNRHPPVFSQEEYITDFMENGTAGEVVFQVMASDDDSGDNGVVTYSIISAEARRMFDIDSETGVLMSEMPIEGDSREIHSRMLLDLESVSDRMLPVPIMAVDGGGKTAYTTVNVILNDRNDNVPRFEMREYKAFTRSNVEIDVPILVVTATDVDSGSNALLTYSFESGTAQSALDLFEIDPKTGSISASSSLQGEEGSTFQFFVGVTDSGNPPLNWVTAAEIYVLSEDDELPVFDGLVNEPDIDVAENTPVGTVLSSFLAHSNSSLTYSLVPGNEVHTNDPSHFSIDEHGNLTLVSALDFESVSWYKLIIKASTNTEPSISAYFTVLVTVTDVNDNMPVFEDVAYNIKVPENTPVDDAILKVVARDADAGSNGQVLYYLDVENDLEVFEKFELNEMTGILTIQEGLDRETAITYDILILARDVTNEDFTSTATVHVTVMDFNDSPPRFTQKVYPGEVLESDPIGTVTVSIEAVDGDIGANAQVVYYITSGDPFNHFAIESNSGMIYVTNPLDRELKDTYRLNVTATDGLFSDTAQVVISVQDINDNSPVCAQTLYTENIRENLASGHYILHVTASDADIGSNAEITFSLEGEGSELFTIEKDQDQNTGTVRTSGTLDYEAQQFYRFQVIASDGGGLSCSSDISIGLLDVNDNPPAFSQLIYNVSVSENTTVNTLLTRVQAIDPDKDANRRHRFSISDGNSEGTFSLDDESGILTLIQPLDRELRSHYNLTLGVTDILQASLTTSAQLIVNVLDENDNEPQFESDLYNASIAEDAEIGSIVVTVLAISQDVGLNAEIMYQIVSGNEHGKFKIDSTTGEISVAFELDFEMSNKYYLTVKATDMGTNPLSDTTTINIFITDANDNSPVFSEDIYSISVNEAARVEGDVIQVMATDEDSGDNGRVSYSIVHGNLFNQFTINPDNGLVSLALELDREQISSYSLGVRASDAGEEPRYTDVTVQIIVDDINDNPPLLPRQNYTIIRQEDTAVEADLLQLNATDLDTQANGPPFIFEIVRGNDNGAFFITSTNMLRNRIWFNRDIETQYNLTIKVSDSARSPLYSLSYITIIIIDPMNMPEVVTPVKISINSFEETFPGGWIGQISASDADPYDELVFELQNDNVFRIGRDDGLIIAPPDLDEGFYHINASVSDGQFTVYAEANVTVQMLNQAMLNNSVTITFADVTPEEFLPNSGIFMYTVANHFSGATPEDVTIITFHPSAENNDNTDVVFSIHRSKSRESFFKPKQIQRQMDSLSAALHERMKHDVVSAVSDLCTADSCEESYICNNIVRLDFSDILTFTTDDESFVSSRLYRDFTCTCIVDSCVTTTKKPTTTKATTTTTPKPRTPSVINDPCASNPCHANMNCRRDGYSGYVCECMDDSLSCLPGPDEAMNFNGQSYVTYTDLVLSTSSTLFSASINTDRPNGVIMDGAGEFDYSVLEIENGYLTYRLNCGTGEAKIRITQKKVNDFDWHKISINREKNHAVLTLDGQYTAEGTAPGENQDLNIDTISIGSSPPAKGRARRSADIGFTGCMGGLSLDNSPLPLLGDSVQPHNIGKCQRRWPMDCSPNPCENDGNCLDYGYSITCDCLHNWSGERCEIKNCQDDVDCEEYPTITSGFPLRLIIIIIGVLVAIIIIVFILAACRYHRNKRRRSRYPPRDGMGRSYDEDYKRDSKLSDSDYPLSLPLNPIATPPSPTPPPLPTRPASYTRSNHNSLNNLDRDRHDDIPYHGQPISMPPSLAPVPSNSASDSDSIAKPTWEFDTPSTHNSFIDGRDSNKGLSPHPMQHSQPSILPPVRRGLNTPMNIRHPPVESPPAYRAVNPNQADMVSMSSVNTENEDDGLRAHGPQRGKKD

>ApFat1

MAVMVKLVRPATVLFTVFLVLWTAAAQEPTYNFQFTEPLYNASIPENAEVRTYITTQPKTGIYVPLDSDIIGVKYILLDEDRLFRATDERVGDFVFLRIRTIPCKSKDEACSSVINREQKAEYRLRVKATGRIRGQSPVTAQTTVVVRVTDANDQRPLFLERSYSVSVPENMPVETSIITVEATDADSGTNGEVYYSFKRWTDQFAIHPTSGVVTLTRALDYQQTSQYSLIIIAKDRGMMPYNMAPPFEATLRVDVEEVNKHAPVMIVQMQMHLSRNSEIGTVYASIMVSDEDDGRNGQISDVRIVDGDPEEYFQIVNEGGIFHIKTQLSFKGPSPTDFNLTIVASDQGNPPKTATEVIPVKISGTYKHAMFFRSRLMNISISELVPINTPLISVAPENEDLGLTISYSLAESKSPVFSINERTGYISLRQELDREMKSVYDITVYGTVQESQAVAYVVVNVLDANDNNPEFAQKSYEVEIQENLAAHTPVVQVTAQDEDLGENAFISYSLANMNPVPFTIDHMTGFINTSKILDYETMRREYRLRVRASDWGAPFRRESEVIVTIRLQNINDNRPRFEKIDCVGTISRTLSPGESIALISAIDFDGDERELSIVSGNTGNLFALHPASGNLTLRRQIEDTDGLFYSLRIMASDGENSASYMYTNISINNNRNINPGAHVRDSKVRCKSTSALTDIRDLLLRRTEDMSLSEDIPDVDFAELYNANHNNPQFDDDFPTIISVREDVAVGNEVVLVKASDRDPGFNGKLIYAISDGNDDSHFQMDFETGVLRVLRPLDREQDSSYALTIKVSDLGQPRSTEKRHITINVEDVNDNAPVFDQTQYDADLSEDTYSGMVFLAVHASDRDIGRNRDIRYSIVSDSPQFAINSGTGELRVTQSLDRELTPVHKIKVQANDMATSNPLSSTTMVTVTIQDINDNPPRCMRDIFSVRMREDLPTGTVLMTVQALDPDEGPNGQVTFGITDETGTFAIDEQYGTLRLISELDYESVQIYDILINLRDHGSPANRSECHVFVQVVDVNENRHTPTFNSFYDEGTVLENATIGTEVMRVTAVDRDTGMDGSVSYSIKDGSGLGWFTIDNGGTIRTAELLDRESIPYFWLTVYGQDHGAVPLHSILEVFIRIGDVNDNSPIPTKAIFEAYIPENSPALEEVIRVEATDPDETSNQALTYEITSGNAYRHFAINSYTGVITTTDNALDREREEYHTLQITVHDNGSPSQSAITHVIVHIQDENDNPPTFNEGALVSINVPAQDRTDEPRIIYVAMASDKDIGENGELSYYIREGNNAGKFDIDPTTGIISTTKLLMEGDQYNLVLEVLDNGRPTQQWARFRLVILISAPVAESPNPPMIVDAMDGAVSEKDPFGTFVTNIFAYDPDDDDLWYSIKAGDDNNNFFMQPSSGIIFVASKLDAERQLTYDLTIAVSDGFHEVTQTIRVSVLNANDNPPLLEQLKYTVDIYENTTIGMEILQVRASDRDRSDRLTYSLKGAADQTSLSMFRVHSKTGMLTTIAPLDHEMRHRHVLTIQVKDHTLVTHRNYTRVVVNILDSNDHAPEFGASAYEGRVFETAAVGTQITQVYAYDKDQGTNAEVTYSIISGNIGEAFNIDPILGIITVGKELDRKEKAIYDLTVKAKDHGAPSLSSFTTVTVSVTLSNNAPPKFSQAEHIVELMENGRPNQFVMEVVAVSRSSVYYRILDGNHARRFDVNANTGVITTEVSLDFEEQMYYNLTLEATNMVGLSCTTQVLIHVQDVNDNPPFFTATNYEGSITESAPLSSVVLDAANQPLVIAALDTDSDRNAQLVYEIVEPDAQKYFMIDPSTGAIRTRMALDHEDISQFDFSVQVHDSGEPELWAASPAHVTIHIVDINDSPPEFTLDIFEANLLLPTFEGVTVLTLEAVDLDTVSISQLEYSIITGNQGKEFTVDATTGVLVVADSSNIQGTYELGVRVTDGLYYGYAEVSIQAVPRTASELRFALDDIYTVIQENDTTVRDLAFLNVLGNGLNEPLTYSILNPDGMFDIRPTSGVLRNTGVAFDREKQDQYNVVVEARDMRDPPRMAHVIVHVEILDVNDNVPVFVHHLFNAIVQVDANIGEVVRQVTAVDRDKGLNGEVKYTLVEGGEGHFDIDSTTGTIVVRRPLRANSQNKNFTLKIKASDKGKPRHSAVVDVPITVQNKAMPLFQEQYYQASILENIQLHSAIIQIQAISPHGRDVLYSVSGGDQFNQFDINPNTGVVNVIGPIDYESQQAYRLTIQASDTLTGAFASVMLDITVLDINDIAPSFHQKVYQTTLSEAVSVGSTVARVQASDLDSPANSRIEYRIITEGEVASFFHLDPDSGLILTSQVLDYERYPRHDFVVLATDSGVPMLSSETRITVLVLDMNDNPPVFSQAQYECTVSELAARGEFVTAVTATDPDISDTGKLTYSIVSGNDKMAFAVNSKTGVISLSNTHEPSLASRYTLNVSVSDRVFTSSAQVRVSLAPVNRHAPEFSLSEYNIEFPENETAGGVVFQVVANDADEGPNGDVTYSIIGHEARMRFEIDSHSGEIRTRVPLDQENSKERTISIPIMASDPGGKTSYTKVNVILTDKNDNRPQFERQQYEAFIPADLEVGSEIIKVSANDADLGSNSQLTYYFDNGTSDSIIDQFEINTQTGLLSVRQSLQGKEGTQFVFFMKATDSGAVSLIGRVPVSIYVLGSHDELPIFQGQEQMLNFELSEDHAIGDTITTLTAQSNRTLMYSLVLGTDPRTNHPAKFSIGRDDGVLSVTSQLDYDTCKWYKLIVKAETQEVPGLANFLEVAISVLDVNDNPPVFDDSSYEIRVPENIPVDSEILQVHAMDVDSAIFGQISYNLAPSEEDEDLEALDMFAVDSETGVIKTKASLDWEMFPSFTMVVVATDGEIGEVQNTAETTVHVVLMDFNDSPPRFTHPFYAGQVLESVAIGTVVTAVLATDSDLGSNSNLVYYITEGDPFGHFAIESNSGQIFVSRELDRELKESYNLNVTVTDGAYMDATMVTITVLDVNDNTPICAQTVYSDDVTESLPSGTDIIQVSASDIDAGDNAVITYSLSGDDAALFAINPSTGQISSAGPLDYETQSLYHFEALASDGSFSCVSQIFVGLLDENDNAPIFSSSVYNESVSENTTLNTLLTRIQAIDPDTGINRQFSFSIMEPDIQSFSIDPSSGIITLRQVLDRENRSIYNLTLQATDANNPFLVSQALLVVNVLDENDNEPQFEHDLYNASLSEDVAIGTTVVTVEALTKDVGVNALITYEIISGNEHNKFHVDEYTGKVTVANSLDFEMSNSYYLTIKATDSGLRPLSDTTMVSIYITDANDNVPQFGQIIYYSEINEVARVEDSIVQILATDADSGAYGEVTYSILRGDLFDQFTINEKNGLISVAAELDREQTSSYSLIIRASDGGEPAQFNDVSVQVSVGDINDNPPRFLRSNYTVIKREDMAVNSKLVQLNVTDPDSSVNGPPFTFTIVKGNEDNDFNIDSDGILYNRFPFNREMKDVYELTVQVTDNGKPPLSSITHVTLEIIEPINPPIVKSPMMITIKSYEDDFPGGVIGWVDAVDADPADVLRYELANNMGNAFRVDPETGSILAQPDLDEGFYQLNVSVSDGQFVSYCDVQVTVDTVTTEMLDNSVTIGFQDLTPEKFLYLYFNVFKHIISNILRSAESADVQVISIQQSEMAAGDTEVLFAVEKSDRRGVSYYKPKSLERQINTSASSFVQRMGVTVKAIVADVCLDKVCDKQRRCESHVTFDQAHVTTIDDGEKAGFVSARHSREPICVCKERVEGCSDLPTAEPPRPQTPTITDPCSSNPCPAYRSCQANGDAFSCVCADNSLSCGGDPSLPMSFSGNSYMRYDLLDVSSLAVRVAVAIHTMEPNGVIMHGEGDDYSTLEIIDGFVHYTFDCGSGPANIHLTTRRVDDGQWHEVSISHQGNVATVTLDRSASATGSAAGDKRALNLNKIVFGARVADQVRARRETVSDGFIGCMGDMNVDGLRLERTGEGITPSNIGECPSKFLKACSGNPCEHEGSCIDYGYSFVCDCRPGWNGNRCEFPFTPCYPDPCANGGTCYSTGNDFQCDCKEPYSGRNCNEVLCPEQVCLNDGFCKLGSMTCNCTGTGYGGTYCQTELTHCSQNPCRYGHHCVQLTKSYKCCSPSSADEVCRSLDLDAVPNITAGPFAIGMVEIIGIVVAAVIAIVLVLLFAVFMRRRRRRRQSHHIHASANQYSMDTFKRDSKGSDYGYPPSPPSPRPPPLPDRPASYTPSNHNSLNNLDTDRYGYDDSQAYHGQPISQQPSLPPLPSNSASDSDSIAKPAWEFDAPSTHESYADGRDSAKNAPISAHHPLQHSHPCILPPARDGRMPLEPPAYDQFSHVEVTSMSSINTENDDDALPAYQWDCSDWMPAEPLSNIPEVQYETPESPTNTNLSDEDIEDEFVGEETDYPAENEDMPYPQGAMRDFQQQLANYPPVDDTPYVGLSSHYQQHPNQYLPKHAISNSTLPPLEAENEEDISDGLPYPQRGQFPGGAASTGDLNFDLEGAIANMDNMSMSVYTDTNASCSDVSGMCDPDSEMALSEYDSVDGTDDEYEEDLPDDRELNAQLKNLTTDV

>PmFat1

MAVRVKPSCWAVTLLITVFTAALWTAAAQEPAYNFQFTEPLYNASIPENAEVRTYITTQPKTGIYVPLDSDIIGVKYTILDEDRLFRATDERVGDFVFLRIRTIPCKSKDETCSSVINREQKAEYRLRVKAVGRIRGQSPVTTMTNVVVKVTDANDQRPLFSQQSYSVAVPEDTPVETSIITVEATDADSGTNGEVYYSFKRRTDQFAIHPTSGVVTLTRALDYRETRQYSLVIIAKDRGMMPYNMAPPFEATLRVQVAEVNKHAPVMSVQMQTQLSRNSDIGTIFASIMVTDEDDGSNGEVTDVRIVDGDPEEYFHIEYEGGVFHIKTQFVFKAASPADFNLTVVASDRGSPPQTTTEIIPVKISGSYQHAMIFRSQIMNISVSELVPINTPLISVAPEDQDPSLAITYSLAESKSQVFSINERTGYISLRQELDRETKSVYNITVNGVIEESKAVAYVVVYVLDANDNSPVFDQQSYSFEIKENFPVHTPVVQVTARDADLGENGFISYSLANTNPIPFTIDHMTGFINTSKVLDYETMRREYNLRVRASDWGSPFRRESEVLVAIRLQNINDNRPQFEKIDCVGTISRTLTAGENIALISAIDFDGDERELRIVSGNTRDLFALHPTSGNLTLRRQIEDTDGLFYSLRIMANDSETSASYMYTNISINNNRNINPGAHVRDSKVRCKSTTALRDFRDLLHQKTEDEKFSENVPGVDFVELYNTNHHTPQFDEDFPSIISVREDAAVGSDVVHVKASDSDPGFNGKLIYAISDGNDDSRFQMDFETGILRVLRPLDREQDASYALTIKVSDLGQSRNTEKRHITINVEDVNDNAPVFDQTQYDIELSEDTETGVVFLAVHASDRDIGRNRDIRYSIVSDSPQFAINSGTGELRVTQSLDRETTPVHNIKVQANDMAANNPLSSTTMVTVTVLDINDNPPRCMRDIFNVRMREDLPIGTVLMTIQALDPDAGPNGQVTFGIADDDTSTFAIDEQYGTLRLISELDFESIQIYDILVNLQDQGSPANTSQCHVFVQVVDVNENRHAPAFNSFYAEGTVLENATIGTEVMTVTTTDMDTGMDGVVTYSIKDGSGLGWFTIDNGGTIRTAELLDRESIPYFWLTVYAQDHGAMPLHSILEVFIRVGDVNDNSPIPTKAIFEAYIPENSPALEEVIRVEATDPDETSNQALTYEITAGNAYRHFAINSFTGVITTTDNALDREDEEYHTLEVTINDNGSPSQSAVTHVVVHILDENDNAPKFNEGVFVSINVPGQDRSEERRIIYGAVASDKDIGENADLSYYIREGNNAGKFDIDPTTGVVSTTKLLAEGDQYNLVLEVLDNGRPTQQWGKFRLVIFITAPVTESLSPPVIVDAMDGTVSEKDPFGTFVTSIFAYDPDDDELWYSIKAGDDNNDFFMQPSSSVILIASKLDAERQSTYDLTIGVSDGFHEVTQTIRVMVMNFNDNPPLLEKLEYSVDIYENTTIGTEILRIRATDRDHSDRLTYSLKGAADQASMSIFRVHSKTGALTTTAPLDHETRRRHVLTVQVKDHNLVTHRNYTRVLVNILDSNDHAPEFGASAYEGRVFETAAVGTQITQVHAYDKDQGTNAEVTYSIVSGNIGEVFNIDPILGIITVGKELDRKEKAIYDLILKATDRGTPSLSSLTTITISVTLSNNAPPKFTQDEYMVELMENRLPNEFVVEVVAISRSSVYYRILAGNHARRFDVNANTGVITTEVSLDFEEQMYYNLTMEATNMVGLSCTTQVLIHVQDVNDNPPYFTATIYKGSITESAPLSSVVLDAANQPLVIAALDADSDHNAQLVYEIVEPDAQKYFMIDPSTGAIRTRMMLDHEDIPQFDFTVQVRDSGDPELWAASPAHVTINIIDINDSPPEFTQDVFEASLLLPTFRGVKVLTLQAVDLDTVSISQLEYSIVTGNHGKEFNVDTTTGVLTVADGSNIHGTYELGVRVTDGLYYGHAEVSIRAKPRTSSELRFASDDIYAVIQENDTTVRDLAFLNVLGNGLNEPLTYSILNPNGMFEIRPTSGVLRNTGMAFDREKQDQYNIVVEVQDMRSPPRMAHVIVHVEILDVNDNVPVFVHHLFNAIVQVDANIGEVVRQVTAVDRDKGLNGEVKYTLVEGGEGHFDIDSTTGTIVVRRPLRTNSQNKNFTLKIKASDKGKPRHSAVVDVPITVQNKAMPVFQEQYYQASIPENIQLHSAVIQIQAISPHGRDVLYSISGGDEFKQFDINPNTGVVNVIGPIDYESQQAYRLTIQASDTLTGAFASVMLDITVLDINDIAPSFQQKVYQTTLSEAVSVGSTVARVRATDIDSPANSRIEYRIVTEGEVASFFHLDPDSGLILTSQVLDYERYPRHDFIVVATDSGVPMLSSEARITVEVLDMNDNPPAFSHQRYECTVSELAGRGEFVTAVTATDPDISDTGKLTYSIVSGNDKMAFAIDSKTGVISLSNTHEPSLASHYTLNVSVSDRVFTSSAQVHVSLAPLNRHAPEFSLAEYNIEYPENETAGGVVFQVVASDADEGPNGQVTYAIIGHEARMRFEIDSQSGEIRTRVSLDQEDPKERTITIPIMASDPGGKTSYTKVNVILTDKNDNRPQFERQLYEAFIPAGLEIGAEIIRVSANDADLGSNSQLTYYFDNGTSDGIIDQFEINTQTGLLSVRQSLQGKEGFHFQFFMKATDSGTVPLIGRVPVSIYILGPHDETPVFEGQDQMLNFELSEDHALGDTIATLTAQSNRSLTYSLVPGTDPRTNLPAKFSIGQDDGVLTVTSQLDYDTCKWYKLIVKAEIQDIPKLAAFMEVTVSVQDINDNPPIFDDSSYEIRVPENIPVDSEILQVRATDVDSAIFGQIRYNLAPSEEDEDLEALDMFEVDPETGVLKTKAVLDREMFTSFTVIVVATDGDTGDAQNTAETTVHIILMDFNDSPPHFTHSSYSGRVLESEPVGTVVTAVFATDADLGSNSDLVYYITEGDPFGHFAIESNSGQIFVSRELDRELKETYNLNVTVTDGAFMDAAMVTITVLDVNDNVPFCAQTVYSDDVTESLPPGTDILQVSASDADAGDNAVITYTLSGDDAALFAIDSSSGQISSAGQLDYETQSLYHFEALASDGSFSCASQIFVGLLDENDNPPVFSSSVYNESVSENTTLNTLLTRVQAVDPDTGVNRQFTFSIDDADVQSFTIDRASGIITLRQILDRENRSIYNLTLQATDTNNPSLVSYALLVINVLDENDNEPQFEHDLYNATLSEDVAIGTTVVTVEALTKDVGLNALITYQIISGNEHGKFEVDMYTGQVLVADTLDFEMSSSYYLTIKATDSGLRPLSDTTMVSVYITDANDNVPQFSQVIYYSEINEAARVEDSIVQVLATDADSGAYGEVTYSILRGDLFSQFTIDERNGLISVAAELDREQTSSYSLIIRARDGGDPAQFDDVSVQVSVGDINDNPPRFQRSNYTFYKREDLPVNSKLFQLNVTDPDSPVNGPPFTFTIIRGNEDNDFNIDADGVLINRFPFNREMKDDYQLTVQVTDNGKPPLSSVAHVRLEIIEPTNPPIVKSPVKITINSYEDDFPGGVIGWVDAVDADPDDVLRFELANNMGNAFRVDPETGSILAQPDLDEGFYQLNISVSDGQFMSYCDVQVTVDPVTTEMLNNGVTIRFQDLTPEKFLYLHFSVFKHIISNILKSADSDDVQIISIQQSELAAVNTEVLFAVQKSDRRGVTYFKPKSLERQINGSVSSFQQRMGVTVQAIVADVCLDKVCDKQRRCESHVTFDQARVTTIDDGENSGFVSARHSREPICVCKERVEGCSDLPTAEPPRPQTPTTDPCSSDPCPDYMSCQGNGDSYTCVCADNSLSCGGGDPSLRPMSFTGSSYMRYDLLDVSSLSVRVAVAIHTVESNGVIMHGEGDDYSTLEIVDGYVQYTFDCGSGPANIPLTRKRVDDGNWHEVSISHHGNVATVTLDRSVSATGSAAGDKRALNLNKMVFGARVTDQVRARRETVSDGFVGCMGDMNVDGLRLERTGDGITPSGIGECPAKVLKACAGNPCEHEGTCIDYGYSFVCDCRPGWNGNRCQYPFTPCNPDPCANGGTCYSTGNDFQCDCKEPYAGRNCNEVLCPVEVCLNGGFCKLGSMTCNCTGTGYGDTYCQMELTHCSQNPCRYGEHCEQLSESYKCCSPSSPDEVCRNLNLDAVPNITAGPFAIGMVEIIGIVVAAVIAIFLVLFFALFMRRRRKRRQSHHIRANANSYVMDTFKRDSKGSDYGYPPASPPSPTPPPLPNRPASYTPSNHNSLNNLEADRYGYDDSQAYHGQPISQQPSLPPLPSNSASDSDSIAKPAWEFDAPSAHESYVDGRDSTTNAPITANRPLQHSHPCILPPARDGHMPLEPPAYDEFSHAEVTSMSSINTENDDDALPAYQWDCSDWLPAGEPLSNIPEVQYETPESPTNTNLSDEDIEDEFVGEDTDYPAENEDMPYPRGAMSDFQQQLASYPPVDDTPYVGLSSHYRQHPNQYLPKHAVSNSTLPPPDPEEDISDGLPYPQRGQFPASAASTGDLNFDLEGAIANMDNMSMSVYTDTNASCSDVSGMCDPDSEMGLSEYDSVDDTEDSEEDDFPDDRELNAQLKNLTTDV

>ArFat1

MAIKMAHIHPAILLVFTVFFAIVRTTNAQEATSPHNFHFTKPVYDASIPENAPVRTYILTEPKTGIYVPLDSAITGVKYTIESETRLFRAADERVGDFYFLRIRTVPCKSKDDSCSSVINREQKSEYRLQVKAVGRMPGQASLVAYTDMIVTVTDVNDQRPLFLESEYSVNVAEDIPIQSSITTVEATDADSGTNGEVYYSFKKPTDQFAIHPTSGVVTLTRQLDYKVVQIYRLVIIARDRGMMPYNMASPFETTLWIHVQEVNKHAPLMTIQTQSQLSKNSDIGTIFASITVSDDDDGTNGQVNNVRIVEGDPDGYFAVANEGGSFHIKTQGLFKQASPGHFNLTVVASDQGSPPKTTTQIIPVEISGSYSHAMFFRSPLMNTSVSEFVPINTPLISVTPEKMDQSIAVSYVLADSKSPVFIINEHTGFITLNQELDRETKSVYNITVFANVQESQAVAYVVVYVLDANDNAPLFDQDMYEVDLQENMPINYPVMQLTARDADIGENGFISYSLANMNPVPFTIDHMTGFINTSKILDFETMRREYRLRVRASDWGFPYRRESEIVVTIHLKNINDNRPQFEKIDCVGTISRTLPAGQFIDVISAIDFDGDERVLSIVSGNPGDLFELHPSSGNLTLRRRIEDTDGQFYSLRIRASDGQGSNSYMFTNISINNNRNVKSGGHMRDSKVRCKSTTALEDIKDLLRRKIEDDRLSESIPDVDFSELYNANHHQPQFDVDFPSIISVTEDAAVGSEVVQVLASDSDPGFNGKIIYAISAGNTGSHFRINYETGLLEILRPLDREQFSNYELTIKVSDLGQPRNTEKHHITINVEDVNDNAPEFDQMEYSAELSEDTETGTVFLAVHASDRDIGRNMDVRYSIVSDSPNFAINSGTGELRVTKPLDRETIAVHEIRVQASDMSPDNPLFSIAVVRVAVLDINDNPPQCMRQIYSVNMREDLPIGTVLMTVQAVDPDFGPNGLVTFSIEDDIASIFSIDRDYGTMRLISDLDFESKQTYDILINLQDQGSPANSSQCHVFVQVIDVNENRHHPAFRNFYAKGSVLENATIGSSVMTVTTVDFDTGMDGFVTYSIRDGTGLGWFTIDDRGTIRTSEHLDRESIPYFWLTVYAQDHGAVPLHSIIEVFVDVEDVNDNSPQATKAIFEAYIPENSPALEEVIRVEATDPDETSSQALTYEITAGNPQFAINSYTGMITTTDTALDREEKDTHVLQVTIHDNGYPSRSATTYVMVHIEDENDNAPVFNEKTYASINIPARDRTDHREIIYRAVAKDDDIGRNAELSYIILEGNEAGKFDIEPTTGIVTTSKLLPEGEQFNLVLEVLDNGRPTQQRARFRLVILIKRAIKPSPNPPEIFDAWDGSVSENDEVGTFVTNVFAFDIDDDDVWYSIKDGDEYKDFFMRPSDGVIIIASKLDAEKKNSYDLTIGVTDGYNELETHILVTVLNANDNPPLLEQLEYTVNIYENTTVDSEILVVKATDLDEGDRITYSIDSASSESSLSKFNIQSRSGILTTTAPLDHEMQKVHILTIQVKDHTFVTHRNYTRVIINILDSNDHAPEFGANEYEGNVFETAAVGTRVTEVYAFDRDQGTNAQITYSIVSGNIGEAFNIDPVLGTITVGRELNRKEKANYFLHIQASDHGLPILSSLTTVTISVTLSNNAPPRFTDDEYMVELMENQMPNEFVLEVAAISRSSVYYQILRGNDLDHFDVNANTGVVTTEVSLDYEEMMFYNLTLEATNLVGLHSTTQVLVHVRDVNDNMPRFISSSYEGSITESAPLSSVVLDLNNQPLVIAALDADSDHNAQLVYEIMEPDAQRYFVIDQNTGAIRTRKILDHEDIPTFEFRVQVRDSGVPVLFAASPAHVTINIIDINDSPPEFVQDTFRAEILLPTFKGVTVLTVKAVDLDTVSISHLEYSIIKGNQGKEFAIDGTTGVVTVNDGSDIQGRYEIGVRVTDGLYYGHAEVVIRAKPRSSSELRFASDDIYAVIQENDTVVRDIAFLNVLGNGLNEPLRYSILNPNGKFHIRPTSGVFRNTGVPFDREEQDQYDIVVEVNDMRDPPRMAHVIVHVEILDVNDNVPVFVHHLFNAIVQVDANIGEVVRQVTAVDRDKGLNGEVKYTLVEGGSGHFDVDSSTGTIVVRRPLGANSQNKNFTLKIKASDKGQPRRSAVVDVPITVQNKAMPVFQEQYYHSSIPENIQLHSAVIQIQAVSPQGRDVIYSISTGDEYKQFDINPDTGVVNVVGLLDFESQQVYKLTVQASDTLTGAFANVMLDITILDINDIAPSFQQKVYHTTLSEVVSVGSTVARVTAFDLDSPANSRIEYRIATEGKIASFFHLDPESGLILTSQVLDYERFPHHDFIIVASDSGVPMLSSETRISIDVVDMNDNPPQFSQSRYECVVSELAVRGGFVMAVSATDPDISDSGKLMYSIVSGNDKMAFAINPKTGVISLSNTHNPELASHYTLNISVSDLVFTSSAQVEISLAHTNHHTPEFSREEYNIEFPENETAGGVVFQVVAVDADEGVNGEVTYAIIGQEARQRFEIDSDSGEIRTKVSLDQEKPKERAISIPIMASDPGGKTSFTKVNVILKDKNDNQPRFERQQYEAFISSDLEIGTEIISVSANDADLGSNSLLTYYFDNGTADSIIDQFEINTQTGLLSSRQSLQGKEGTLFQFFMKATDSGTPPLVGRVPVSIYLLTSHDVPPIFESQEQQSFELPENHAIGEIITTLRARSNQTLTYSLVPGTDLLTNNPVKFSISHDGGVLSVSSQLDYDTCKWYKLIVKAETEEIPKLASFIEVTVSVVDINDNPPVFDDTLYEIRLPENIPINSDILQVHATDIDSAAFGKIIYNLAPSEEDEEFEAMEMFTIDSETGIIQTIAALDREMFSSYIMLVVATDDVEGQVQHTAEATIHIVVMDFNDSPPRFTHPTYSGQILESDPIGTVVTSVFSTDADLGSNADLVHYITDGDYFGHFAIDNNSGKIFVSKELDRELKELYYLNVTVTDGAFMDSTMVSITVQDVNDNPPICAQTVYSDDVSESLPEGTDILEVMATDEDAGDNGVIVFSLSGDDAALFAINENTGLISSAGTLDYETQPLHHFEVIASDGIFSCVSQIFIGLLDENDNAPVFSSSVYNESVSENTTLNTLLTRIQAVDPDTGINRQFTYSIDTSEIKSFTIDRSSGIITLRQILDRENRSSYNLTLQAMDTNNPSLVSNAVLVVNVLDENDNEPQFEHDLYNVTLSEDVPVGTTVVAVEALTKDVGVNALITYQIISGNEHGKFSVDQFTGKVMLVTELDYEMSSSYYLTVKASDSGLRPLSDTTMVSVYINDANDNVPQFSQIIYYSEINEAAQVEDSIVQVLATDFDSGAYGEVTYSILRGDLFDQFIINEKNGLVSVAAELDREQISSYSLIVRASDGGEPSQFNDVSVQVSVADINDNAPRFQRSNYSFVKREDTAENSHLIQLNVTDPDTLVNGPPFTFSIAQGNEENDFSIDADGFITNLLPFNLEMKDKYQLTVQVTDNGKPPLSALTYVTIQIVEPTNVPIVQSPLRITINSHEDDFPGGVIGWVHAEDADPSDVLVYELINNMGNVFRVDQDNGRILAQPDLDVGFYQLNISVSDGQFTTYCDVQVTVNPVSTEMLDNSVTIRFRELAPEKFLSFFFNTFKQTISNILRSGGSADVTIISIQQSEAVPTDTEVLFAVQKSDRRGIAYYKPKSLERQINTSASSLEDRMLVTVQAIVADICPENACDKQRRCVTQVTFDQSSVTTLDAETVGFVSARHSRAPVCVCKEKEEGCVDGPTPLPGQPDTGTDHCASSPCQSFMNCVQDRDGYSCVCADNSLTCGGGDASLRPMSFNGNSFVQYDLLDISSLSVRVAVAVHTTEANGVIMYGEGDDYSALEVSDGYVQYTFDCGSGPANIRLTTKRVDDGDWHEVSVSHQGNVATVTLDRSSSATGSAAGDKRALNLNKMVFGARVTDGMRVRRETVSDGFVGCMGDMNVDGVRLERSGDGISPSSIGECPSKVLKACSSNPCQHEGTCLDYGYSYVCDCRPGWNGNRCEYPFTPCYPDPCANGGTCYSTGNDFQCDCQEPNTGKTCNEVLCPDEVCRNGGVCSASTKTCNCSGTGYGSTYCGLELTLCSQNPCRYGEHCEQLAESYKCCSLSSMDEVCRSLDLESIPNMTAGPFSIGMMEIIGIVVAVVIAIFLVLIFALFMRRRRKRYQSRNLNLDSSRYPMEKRDSKGSDYDYHHPPSPPSPRPPPLPDRPASYTPSNHNSLNNLDSDRYGYDDAQPYHGQPISQQPSLPPLPSNSASDSDSIAKPAWEFDAPSAHESYVDGRDSAKNVPINAHHPLQHSHPCILPPARHGHMPLEPPAYDQYNMAEVTSMSSINTENEDDALPANKVVPITADEVNKSALVGKPPSPSTPSSNQSDSDTSQDTVISFEKGQSPIKPSEKPRSPSKISPVAPVKSISDGSPTEPRKENCQALKNAAPIIQKSPSPKSPKKYAPKSPILKKRPSSTSPVLETDIDAPIATPKSVRFNMEPEISSFEVDSSQSDTQTDSDTERSYADMIGLKIERRSDGGSVVSDPSNRNSDCEIGFNNVPPLDFLETQKALGVEGPVQILADVSCQTVDDTNESSTSPSFLEMETPLETMTEFHDTHQTVYITQNPEEDDEEGKKKAYQWDNSDWLPSEPLSNIPEMQYETPESPTNTNLSNDDIEDEFVGEDTDYPAENEDMPYPRALRDFRQQLENYPPVDDTPYVGLTSHYQQHPNSYLPKHSISNSTLPQPDMEYDDEITDGLPYPQKGQFPPRGAAAAVNDLNFDFEGAIANMDNMSMSVYTDTNASCSDISGECDPDSEMALSEYDSADGADDEEERDGDLNAQLKNLHTDV

>AjFat1

MKHQVIIMWSLVVVLLLNVVIVKSQTGQFKFTEDVYKASIDETSKTSLYITSESKMGIYVTDLQLATKIAYTIMGEESKTFKADNARQVGDFVFLRIHPKTTLNRERRPSWVLRVKARATDKSGTLLEAETRIMLTIEDINDLRPIFHGSSEPLNIREDTPVQTTIYTVQASDADSGTNADIYYKFLDDTDYFVIHPTSGGISLTRPLRPHREKAEYSLLIVAQDRGGTLAPSKTSTTSINIKILEVNYFPPTIKVDSLEILTLDSRKEKVIAVVTVSDDDLGDNGGIKSVLLTGEYSDYFNINKDDESESTYQISIGKEFPSDMPLIFTLSVVATDTGDPPKSSSKKITVERSNGKIKYFGKKLFDITVSELIPMDAPIFTVASEQVLSDVKTRPEYNIVAGNKRGSFKINPSNGYLSAAKTLNRERIPNFNLTIRASNHPSHQTYVSIKILDENDNYPVFKQNRYQTTVQENLPANSPVIRVSATDDDNGDNGFVSYSIANLESVPFEIDSFSGQINTTMVLDYEVMRKEFRLHIRASDWGSPFRRETEVDVIISLQGTNDNKPMFEKAYCSGTISRDIRSGNQVGVFSAIDFDSDPVTYTIARGNVGELFAIDRTTGTLKLKRDIQNSDPSLYNLKLMASDAPGSAGYLWVNMSIINGRNANRNERRKNAKINCESSDALTKHTDMISKSTKANEEIVEPNNEFTERFGRNEFTPEFDEQFPSTFDVLENTPKGSTIVIVKARDRDPGFNGKLIYAISSGNEDFPFHLDHNTGVLSVLTPPDRERTGTYDLSVTVTDLGKEAKTKKKDIRINIVDVNDNSPTFVTEDYSEKLYENVDIGEIFLRVSANDPDAGSNGEIRYSIQTDNDNFLIDTTNGDISVASELDRESIPIHELRVVATDSSESAPLSSTVTVTVTLQDVNDNAPRCIPPNYFVRMLEDLPSRVLVIDVEAIDEDEGKNGDVTYSLKKGGDDKFVIDPLMGTIRLADRLDYETQQVYSLLVEVKDGGSPRKKATCSVQVEVIDVNENVHAPSFGNRFYVNGSVEENVPINTYVMQVTADDQDSGRDGEVTYSIRNGSGLGRFTIDSEGIIRTASPLDRESATHYWLTIYAQDKGAVPLHTTIEAYIEILDVNDNSPQPTEPVYYAEVAEKSPVGRSVVQVKAKDLDSSSSQQLLFSIQNIWDANNNEVSNVFAIDESTGVISTLSDDLDREETDGYSLEVLITDNGTPPMSTVAMVIITVTDLNDNKPRFKKNYYTPKIPVKDRTSEAMPIFRVYATDNDAGLNAELSYFITQGNDNQRFSLDAKTGILSTRRSLSASADYPLLSLEVLDTGTPKQKRGTAKISLTVFDPPRNSPNPPQFLDAGLTVVNIQETLRVGGLIEVVNAIDIDRDILFYQIADGDPTRTFFIDPNEGIIQLALPLDWEVKAHYDLNISVTDGTHTVYKQLLVNVLDSNDNAPVFDKTEYEVTISENAPVDPSISILTVRATDQDSARRLSYELLNNTDSAREGLFKVDGLTGTITVAKRLDHENRKQHTLNIMAKDHGIPTKKNYTRVVINVTDFNDHAPACAPEGFEGRVYESAAPYTRILQVTAVDLDKGKNAELSYSIESGNPDNDFLISSGGYISVSSELDRNKKSFYQLVVRVKDHGTPMKSCTCNVNITVTVSNNAPPKFLVDEYMVNVYENMPARKSVVVIQAESLSSVVYEIISGNVANDFEVNPSTGVVSTLVPLDYEEQTIYSLTVQATNMVGLSATVSVVVHVEDVNDNVPEFLYNLYQGNITESKPPNSPVLDSLGRPLVISAEDDDSDHNAHLMYQIIEIEAQKYFTIDSNTGAIRSKVKLDHEQRNQFKFTVQVRDSGEPQLQSINPVDVLVTIVDINDSPPLFDKDVYSTILILPTYKDVAVVTVHATDADTASITNLEYSITAGDPERRFSIHPVSGVITVINSTNLMRNYVLTVTVSDGMNKGTAEVQISVQQAQESELRFAQNSYSATVMENDPTIQRIALVSAVGNALNEPLEYRILNPDKRFNISRTSGILRTTGIPFDRETKKEYNIVVEVRNTRVDERRAHVLVNVKVLDENDNAPMFVHLDYKAVVQVEAEVGSIVRTVLAIDRDIGKNGEVKYSLVEGGDNRFNINSTSGEIMLVKKLDPSNQNEDFILKIKASDKGNNKHSKVVDVPISVINKAMPVFDRPQYTKTIPENIQLHTAILHIQATSPDGRKVIYSISAGDDYNQFVITNKTGALKVVGAMDYETTQEYRLSVRASDTLTRAYAEVLVVITVEDINDIAPLFDAPVYTRTLSEAVAVGTSVANVLALDYDSGTNGQVSYHIVEDGSMTDSFYIDSHSGSILTSRVLDYESIQNHNFLVRAVDGGMPQLSSVTRINVKITDLNDNPPVFTKPAYEATISELAGKGAFVCVVSATDRDITDTEKLTYTIISGNEEMNFYIGSRTGIISVTNTRQPNLNADKTYILNISVSDTVFTNNAYVIVNVDGTNQFAPTFTRFEYSVSLDENQPIGTSVIQVSATDDDEGTGGRISYSIIGEDMRSKFIIDTRTGEISTASIFDREDPSERTMTIPIMAIDEGHRASFSSVKVILEDKNDNAPTFEVEKYEAYIYADINVGDQVLQVYASDADKDQNAKITYSIFAGMDDVVTELFEINPVDGSILLKSSIRGKETNLFQFFVKAVDGGNSPLGGDAQVEIYVMNQEDALPAFVDIIPQFSVMEDAQVGSVVGVVTAIHNETLVYSVVPGNLIRTNNKSKFSINDEGEVLVNSPLDYETCSWYTLTIKAATDTDPSLASFKEVRVDVQDVNDNAPRFESSSYDIKIPENAPVGYSVLRVHAYDKDTTSQIEYSLSSLQSNDVSAIFSIDRKTGWITTSSKLDREATSSYVFAVIAQHVMDEDDEEGANVRSTNTRVTVHVMDVNDSPPRFTSAIEPVTVPEDIEPPFVIIRVSASDADYMANAMITYHIAGGDPFGQFHIDNTTGKISTTKKLDREFKSKYTLNIKATDGVFFDTTNVTVLVGDVNDNAPVCLQMLYSELVNENLGVSSYIVEVLASDADAKDNSGITFTLFGEGAEDFEIHPESGILRTAALLDRETKSTYQLKVNATDGGDLSCTSDVLIGLVDDNDNPPVFEPTIITEAFSENTPTGSLLTRVQAIDPDEGTNRQFSYSFVDSAGGMFGIDRKLGIISLLKALDRELKDTYNLTIRATDMHKNELSSVAYLIVKVLDEDDNAPIFEYENYYVNVSEDVDIGVSIAQVLATSEDIEEITYSITGGNELGKFNIDTNTGLISVVDTLNFETVQDFILTVQASDNGQQQQTDISTINIKILDANDNKPEFSKQIYNAVINEAAQVGDSIIQIMATDKDAGDFGKVLYRIVRQYEEGEPKFEINQRDGLISLAAPLDREEMESYTLTVRAIDGGSPSMFTDVSVQVLITDINDNKPRFSNNNYTIVIQQETFPVSTLKTLEVTDPDSLTNGPPFTFEITQGNDEGLFGIRPTDSGSSGLFQAVKHISQKDPRTIDVVVRATDSGTPSLWAETYVSVIITESTHPPVPVTPLQITINSFPEYFPAGFIGKLHADDLDSHDELEYGFVSETEQFTIQDDSGKIFIQPAPQDDHYELNVSISDHKFTSFADVFVDVVFISQEMLDNALVITVRGVSPEDFLFDYYAIFKHSLAGTFISSDQPVDASNIQVITIQESRPGSEDIDIIFAVQHATKEDTYLRPKPLIRLINSSKTSLGDRVGIEVVSASSDACQLNTCSKNARCKSKLVQDAIRPATVMSTRMSLVSVRMEWKIECICKEEGECGPPAPIAPALSFSGNSFLSYTADEKRTRFSSSVRTIQPDGILMYGDGDEDFSVLEVKDGYLQYRFDCGSGEGLLKISSPKINDGLWHDVSVSRDNNEATLTLDGKHTVSGTARGQNKDLNIDRITVGGKVGSDRKRRERDTTVVTNGFRGCMDGVNLNGEQLTASGDSEDSDNIGECPEDFPLHCDSNPCQNQGQCNKLTYSFECICLTGWHGPQCQFRDKCLNNPCQNGGTCMRNGDSFTCSCSGNNFGGVLCTNYCSSSPCLNGGLCYEDRSGPSCLCENYIGERCESDIDECVSNPCELGFVCINIPGSYECKDCRQSSNSDCNVVDTVADPNIKSSVLNFNKEEIIGAGLCIFVIIVIVIIFTIVVRRRRRRRYSETDSTSPPVTRLDNRHDYKRDKVSNLDVHHVVHNPPPVPIRPRSYTPSNHNSLNNLNDNERYYDGHDGQNVYQGLPVSQQPSLHPIPSNSDSDSIAKPHWDFDNRSGTGSFADGKESHHNIPVNVPPIHQPPPPVRPSVRHPVYRKEGLGNADLTSMSSINTAVTENDDESLPASKRRMQERLNSIKEEQKRATLPQMSTAEAQTSPQEQIETLFPASGSPYSSPIHLTRSDVGGHTVLRPSPEIILINNTNQPSTPKSQKVLQTFAPLVEMDGYIKHNEASALVGCAVVKSPDKVRNALIIRGSPDLQSDIWKLQNGEYSDDETLWVPQNPNVSETDTLPHGATLQQQSSEQNQEAHELVDHLCYDNVPVGFKDNPNKETTFSSPTMKPKVLDLEDGQAGYASSCNGSPIWKPQPPLAFSDHASTLPHGVTIPQCIDTDSPSKQNSQSGILPILKKNKRSKSCPDIIGNTLKDSGSKTVRFNMQPEVTQFLVEDTFAGDSNGSPDQENFYSEISQDEQETNSQTQTVFDADGLTTEQRTNGHVETMFDSPAPTFTADQGMETEFDAEGLTTRCHSRGHVETEFGTLERGTTARNSNNDSNGGSPFSHQNRPPNYPSPPMYHSSSPRVTADTPLLTGSESSAGVIDAVCQTDSDDDDDGAEPLLPERQPEVQHILTVSSTPHFTCVDSLNEHCPSVHRGSQVIRIKGYHWDCSDWMPPTLTKIHEDVNQKEVPDSPTNLSVNTTMSQMNDFDLDDEYVGEDTDYPGDDDMPYPHTEEFQKQLQSYPPNISPQSTRH

>BfFat1

MEGNTGVLRICAIVFVTVLCRLAVCQRTPSPPGDFRFTRKEYNATIMENSQAKTYVKSRQKMGIYITSDLSASVKYSIVDGDPLQLFKAEQHLVGDFFFLRIRTKGGNTYVLNREVTDSYKLRVKAEVRKTNSRKLEFWVDVFLAVQDMNDLRPLFSPTSYYISVSEDTQIRSSIAQVTATDADIGTNGEFYYSFSQETDTFAIHPASGVVSLTRQLDYNQTPRYELTIIAEDRGLHPGGSGFSSTSRLQITVVQDNLHAPEILVRTLPMVVEHNAEPLTYAVITVSDKDSGRNGNIQGLEIIKGNEDGKFEIQTGGKQNEFAIKVLQTLDRETTPNGYNLTLRASDKGQPPKFSEKVLFVQLMDTNDHAPVFSQTRYEVTVSEFAPVNTPVVMVQATDPDTGRNAEIVYDIQRGNNKLRFGINSRTGLIYTKGELHREERASYELTVVATDRANPSNRKRNSAVVVINIEDANDHDPVFNDSKLSANINENEKAGTWVLEVSAYDLDAGENGYLTYSLANVNPVPFEIDHFNGWITTTKELDYEMMPQKYMLRVRASDWGSPYRRETEMTIPVKLQNMNDNTPQFEKVKCEGTLSRDAPIGQRVHTLSAIDDDPADYVRYEILSGNENGLFELNPTSGDLTLARKIGQFEPSFISLKVTATDGDNQASPVYLNMTVTNSMQGNDYINLQCQSTGVAQEHMRKVQQKSRINALEEDETMHFSDIHTVNLHAPEIDSGIPREIDIGEDSSVGTVLATVNARDSDTGYNGMVVYVIAEGNTDSCFTIGMQSGELSILSPLDRERTESYILNITATDLGKPSKGAWKRITINVMDANDNKPRFSQDKYEETVDENIDIGSTILQVYASDPDSGSAGAVRYSLLTGTEKFSINEKSGVIKTTAALDREEAAVHSLRIQARDQDESYPLSSVVTVTVTLQDLNDNAPKFIPENYKVKVREDLPKGAVILMLEAQDPDEGRGGEIRYSLQDGDEGKFDVDRLTGVIRLVGMLDFEDRQVYNITARAKDKGSPQLTSTCKIEVEVIDINENLYAPEFPSFVSNGTVYENEEVGTEVLRVVATDLDSGNDGKVVYSIRDGSGLGRFVIDDEGIIRTAEVLDRETESRYWLTVYAQDRAAVPLYNIIEVYIEVLDVNDNAPLTEFPIYYPSIDEGSGSGKSVIQVRASDPDEDGTQKLTFRITSGNPQGFFQINQNSGLITTTSRRLDREQQAEHILEISVSDNGSPPQVSTTHVVVTVADVNDNSPDFGTNTIRIRVPERPRTNEKGDIYRVVASDDDVGPNGDLAYSIKGGNEKGRFTIDPTNGMISTRKPLVDGQQYNLKVMQKFRINPRTGEIYTASPLDHETKDQHILTIMAKDQDVSVMRNFVRVVINVKDSNDHRPIFSAQEYTGGVYETAAYGTSVVQVVATDRDRGANSELTYTIETGNVGNVFSIDETLGIITVSKQLDRSALSQYHLTVKVRDNGNPPLSSTVPIHIGVTISNNAPPKFDAKEYAIEVSENARLGSLIVMLGATSRSSVTYEIIGGNTDGSFDVNPNSGVITCKKQLDYERQTSYNLTVQATNMVSLSSTATVLVHIADENDNPPVFSQSEYIGSISEAATIGSVVLDVNNIPLVIAATDADNELNSLLVYEIIESAAQKYFSIDSNTGALRTIRTLDHEDIAEFRFTVQVSDTGNPPLKAENPANVTIKVLDVNDSPPTCTQDIYEATLLLPTYKDVAIVTVEAEDADTEANTQLSYVLTMGNEDKKFSINKDTGVITIVNTTGLFDRYELSVRVGDGKFHTSCIIRIEVKRTVMSGLRFTEESYFSEVTENSTEVKTVAIVTAVGNLLNEPLEYKILNPNNMFEVSPTSGVLRTLGIPFDREEKEKYEIVIEVRDKRSTPRVAHVVVFVTIADINDNAPVFVNLPYYSVVQVDAEPGTPVRTVTAVDKDAGKNSEIRYSLDSGAHKRFRINRKTGQIMVKQPLEKDDTNKEYHLVIIAEDKGNPPLSARIEVPVTVMNKAMPVFEKPFYAVSIPENIQLHTPVINIKAASPDGLKLIYSITDGDPFNQFNIDFDTGVINVVGSLDYETKQNFRLTVRATDSRSGVDAEVVVDITVQDINDVAPVFEKPSYEATLSEAAAIGTTVIKVSATDLDSGVNQLMYYQILQDESNSTDYFHIDTSSGLILTARNLDHEKIEKHDFTVRVSDGGMPSLSSEVHVIVTVLDLNDNAPQFDQPSYDCMISELAPRGHFVTKVSASDADSTDNNRLVYAIVSGNEQMNFVIDPKTGIVSMSNLRKRELDPTYTLNVSVSDGVFTSSARVAVVVQSANTHSPVFSQLNYAVSFDENNPRGTYVVMVTATDEDAGAYGEISYSIASEKINQLFEIDADTGQIYSKMALDREDENQRMIIVPVVASDQGGRVSFCNVTVTLTDKNDNTPQFELASYQANIPTDAEEGAEVIQVNAIDQDIGTNSDIAYSLYDDSDTTAAVVKLFSVHSETGMITTRQKLTGKEGKAFQFFVQATDGGNPTRTSSAPVEILILGPDDIPPYFDPTVQHLYFVSEEMPVDSEVATVVADTNDTVKYSIVEADGPNSNKDNTFRIDSENGVIYVNKELDTETTAWYSLKVQAETLSSPPLLAFADVSIQVKDANDNKPKFDSKPYKITLPENASVGTSVIQVHAFDPDQGANGEIVYGFASDSNADEMSDFFTIDSESGWVTTLVPLDREMMSSYAFGVTATDKGEPKQLKDTTLVHVTVADVNDSPPTFRSSTYQGQVREDALPGTIVITVSTRDADIGDNTIPTYYITGGDPQGQFNIERKTGKVYVNGPLDRETKQLYVLNITATDGAFTAMATVNIDVQDVNDNSPICEQSRYTASISEDITPQTFVMEVLARDPDQGIHSQITYSLDGVGADKFLLDKDTGVLMTGAPLDREETPVYTLGVTAKDGGGKSCYTELTINLIDVNDNPPKFEKQQYLVPVYENTAVNTLLTRVQATDPDMGLNRKVMYSFVDSANGQFQVDENSGIVSLAKALDREAQASFNLTIRATDEGAPRRSSTSYLIISVLDINDNPPEFEFAEYAKNVSESTPIGSQIINVYAVSKDVGPNAEITYEIISGNEHGKFEIGSYSGKITIVNTLDYEASQGYYLTVKASDGGMPTLSDITTVSINVTDVNDNAPEFSMPMYSASISEDAHTGDSVIQVMATDRDSPPNAQVTYTIVRGDSKGQFNIAPKLGIVTVSGALDREEIQAYSLTVRAKDSGASPRYMDVVVNVDVLDINDSPPQFSQANYSVFVQTCSKGMECQFPQGGMESKPIGTSILMFTVKDFDAPQNGPPYTFSIISGNQGNEFHIDRSGILRTSVVFQSDMRRKYNLQVRVADAGTPSLYGYTNVVVEVIEESMYPPVVTPHQISISSFRDDFPGGVIGKLHATDNDMYDKLTYSLVSNNKRLFGVNPNDGKIIAQPGLDVGEYILNISVSDGKFITAGEVSVTVKLVTDEMLRNSVTVRFANIQPEEFLSSYQKEFFRTLRRLLKVKNKNIIVVSMQPVDGNLDVLFCIETTTKGSQKEDYYKATALRRELNQTVMDIQQNVGLSVLQIISDSCSHNKCPVGACRDILNLDIHAIAAISTAKISFVSPRHMRDYECKCPGGLIGKDCNPCNSNPCPRYKMCLQDPKSIEGFECVCPDGTSPPNCNQLPFDDKGPMTFGGNSYIRYTLANSVDQMSTQLSLAILPRSPNGKIMYARGEYDYSILELINGYLQYRFNCGSGEGKVTMEFGGSEVTDGKWHYVEVSRNGAYAELKLDHKYIARGSAAGENKILNLDDNDIYFGAEVANRLRKKRAAVVSNGFLGCMDDMMLNGERLPRAGANSVATLRDMTDVDFNCNDGMETLGVCGSNPCMNGGSCAVVSPSNRYICNCLPRFSGDNCQIDTQPCNSNPCKNGARCKNLLNDFKCECQREWYGKRCEHSTSCNCFNGATCLDTPSGKTCSCPDNFTGEKCQEDVNECSRNPCQNGATCHNTEGSYFCNCTANTNGLHCENIIEMIMPKIESNSINIGLEEIIGIICCIIFLLLLVIIFVMVRRCRQRRKRNPHMSDIVATGGDPNGIMMTEKRDDYHRDAKMVHIDTNLDDYRPPLPARPISYTPSLCGNSLNNLSDGAMGAEVEPVFSSSTDMLAVRNKNPAIVCSVAPTLPPPPPSNSASDSDSIQKPAWEFECPNVVEGYVESKVNDADAVMNNIANDEMQVYRNEGGSMTDMVSLSSLPSESCDEDNMPDYDIHCYETDGKHADMFRRQLYRLI

>MmFat1

MGRHLTLLLLLLLFLQQFGDSDGSQRLEPTPPIQFTHFQYNVTVHENSAAKTYVGHPRKMGIYILDPSWEIRYKIVSGDSENLFKAEEYVLGDFCFLRIRTKGGNTAILNREVRDHYTLIVKAVEKATDAEARAKVRVQVLDTNDLRPLFSPTSYSVSLPENTAIRTSIARVSATDADIGTNGEFYYSFKDRTDVFAIHPTSGVVVLTGRLDFLETQLYELEILAADRGMKLYGSSGVSSLAKLTVHVEQANECAPIITAVTLSPSELDKDPTYAIITVEDCDQGANGEIASLSIVAGDLLQQFKTVRSFPGSKAFKVKAVGAVDWDSHPYGYNLTLQAKDKGTPPQFSPVKVVHIISPQFRAGPVKFEMDVYRAEISEFAPPHTPVVLVKAIPSYSHLRYVFKSAPGKPKFGLNHNTGLISILEPIRRQHTSHFELEVTTSDKRASARVVVKVLGTNSNPPEFTQTSYKASIDENAPIGAAVTRVSAMDPDEGENGYVTYSIANLNHVPFVIDHFTGTVSTSENLDYELMPRVYTLRIRASDWGLPYRREVEVLATITLNNLNDNTPLFERINCEGTIPRDLGVGEQITTVSAIDADELQLVRYQIEAGNELDLFGLNPSSGVLSLKHSLTDGLGAKVSFHSLRITATDGENFATPLYINLTVAASRKPVNLQCEETGVAKMLAEKLLQANKLHSQGDVEDIFFDSYSVNTHTPQFGVTLPTGIEVKENLPVGANILFMNATDLDSGFNGKLVYAISGGNDDSCFTIDMETGVLKVLSPLDREVMDKYTLNITVYDLGIPQRAAWRLLDVTVLDANDNAPEFLQESYFVEVSEDKEVNSEIIQVEATDKDLGPSGHVTYAILTDTEKFSIDSMTGVVKIIQPLDREVQPVHYLKIEARDQATEEPRLFSTVLLKVSLDDVNDNPPRFIPPNYSVKVREDLPEGTIIMWLEAYDPDVGQSSQVRYSLLDHGEGHFDVDKLSGAVRIVQQLDFEKKQLYNLTVRAKDKGKPVSLSSTCYVEVEVVDVNENLHTPVFSSFVEKGVVKEDVPTGSSVMTVSAHDEDTGRDGEIRYSIRDGSGIGVFRIDEETGVIETSDRLDRESTSHYWLTVYATDQGVVPLSSFIEVYIEVEDVNDNAPQTSEPVYYPEIMENSPKDVSVVQIEAFDPDSSSNDKLTYRITSGNPQGFFSIHPKTGLITTTSRKLDREQQDEHILEVTVTDNGVPPRSTIARVIVKILDENDNRPQFLQKFYKIRLPEREKADGDRSASKREPLYRVIAADKDEGPNAELSYSIEEGNEHGRFSIEPKTGVVSSKKFSAAGEYDILSIKAVDNGRPQKSSTTRLHIEWISKPKPSSEPISFEESVFSFTVMESDPVAHMIGVISVEPPGMPLWFDIIGGNYDSHFDVDKGTGTIIVAKPLDAEQKSSYNLTVEATDGTTTILTQVLIKVIDTNDHRPQFSTSKYEVAVPEDTEPEVEILQISAVDRDEKNKLIYTLQSSIDPASLKKFRLDPATGALYTAEKLDHEAIHQHVLTVMVRDQDVPVKRNFARIVVNVSDKNDHAPWFTSPSYDGRVYESAAVGSVVLQVTALDKDKGRNAEVLYSIESGNIGNSFTIDPILGSIKTARELDRSHQVDYDLMVKATDKGDPPMSEMTSVRIAVTVADNASPKFTSKEYSAEISEAIRIGSFVGMVSAHSQSSVMYEIRDGNMGDAFNINPHSGSIITQRALDFETLPMYSLTVQGTNMAGLSTNTTVVVHVRDENDNPPVFTQAEYSGFISESASVNSVVLTDRNVPLVIRATDADRESNALLVYQIVEPSVHNYFAIDPTTGAIRTVLSLDYEETHAFHFTVQVHDMGTPRLFAEYAANVTVHVIDINDCPPVFSKSLYEVSLLLPTYRGVNVITVNATDADSKAFSQVMYSITEGNIGEKFSMDHKTGTIAIQNTTQLRSRYELTVRASDGRFTSMASVKINVKESRESPLKFTQDAYSAVVKENSTEARTLAVITAIGNPLNEPLFYRILNPDRRFKISHTSGVLSTTGIPFDREQQETFDVVVEVTKEHEPSAVAHVVVKVTVEDQNDNAPVFVNLPYYAVVKVDAEVGHVIRYVTAIDRDSGRNGDIHYYLKEHHDHFQIGPSGDISLKKQFEHDTLNKEYLVTVVAKDGGSPAFSAEVLVPITVMNKAMPVFEKAFYSAEIPENIQMHSPVVHIQANSPEGLKVFYSITDGDPFSQFTINFNTGVVNVIAPLDFESHPAYKLSVRATDSLTGAHAEVFVDIIVEDINDNPPVFVQPSYSTTLSEASVIGTPVLQVRATDSDSEPNRGISYQLIGNHSKSHDHFHIDSNTGLISLVRALDYEQSQQHRIFVRAVDGGMPALSSDVVVTVAVTDLNDNPPLFEQQVYEARISEHAAHGHFVMCVRACDADSSDLDKLEYSILSGNDHKSFIIDRETGIITLSNLRRHTLKPFYSLNVSVSDGVFRSSARVNVTVMGGNLHSPVFHQNEYEVELAENAPLHTLVVQVKASDRDSGIYSHVTYHIVNDFAKDRFYVNDRGQIFTLEKLDRETPAEKVISIRLMAKDAGGKVAFCTVNVILTDDNDNAPQFRSTKYEVNIGSSAAKGTSVVKVFASDADEGSNADVTYAIEADSESVKENLEINKLTGLITTKESLIGLENEFFTFFVRAVDSGSPPRESVVPVYIKILPPEVQLPRFSEPFYTYTISEDTPIGTEIDLIRVEHGGAVLYILVKGNTPESNRDEFFVIDRQNGRLKLEKSLDHETTKWYQFSILARCTLDDYEVVASIDVSIQVKDANDNSPVLESSPYEAFIVENLPGGSRVIQIRASDLDSGANGQVMYSLDQSQDADIIESFAINMETGWITTLKELDHEERASYQIKVVASDHGEKVQLSSTAIVGVTVTDVNDSPPRFTAEIYKGTVSEDDPPGGVIAILSTTDADTEEINRQVSYFITGGDALGQFAVENVQSDWRVYVKKPLDREQKDSYLLTVTATDGTFSSKARVEVKVLDANDNSPVCEKTSYSDTIPEDALPGKLVMQVSATDADIRSNAEITYTLFGSGAEKFKLNPDTGELRTLALLDREEQAVYNLLVKATDGGGRSCQAAIVLTLEDVNDNAPEFTAEPYTITVFENTEPGTPLTRVQATDADTGLNRKISYSLVESADGQFSINERSGIIQLEKHLDRELQAVYTLTLKAVDQGLPRRLTATGTVVVSVLDINDNPPVFEYREYGASVSEDIVIGTEVLQVYAASRDIEANAEITYAIISGNEHGKFSIDSKTGAIFIIESLDYESSHEYYLTVEATDGGTPSLSDVATVNINVTDINDNSPVFSQDTYTTVVSEDAALEQPVITIMADDADGPSNSHIHYSIIEGNQGSPFTIDPVRGEVKVTKPLDRETISGYTLTVQAADNGNPPRVNTTTVNIDVSDVNDNAPLFSRDNYSVIIQENKPVGFSVLKLVVTDKDSSHNGPPFFFTIVSGNDENAFEVNQHGVLLTAATIKRKVKDHYLLHVKVADSGKPQLSSMTHIDIRVIEESIHPPAILPLEIFITAFGEEYSGGVIGKIHATDQDVYDTLMYSLDPHMDGLFSVSSTGGKLIAHRKLDIGQYLLNVSVTDGKFTTVADITVHIQQVTQEMLNHTVAIRFANLTPEEFVGDYWRNFQRALRNILGVRKNDIQIVSLQPSEPHSHLDVLLFVERSGGTHVSTKQLLHKINSSVTDVEEIIGVRILEVFQKLCAGLDCPWKFCDEKVSVDENVMSTHSTARLSFVTPRHHRTAVCLCKDGTCPPVHHGCEDNPCPAGSECVADPREEKYSCVCPGGGFGKCPGSSSITFTGNSFVKYRLLENENRLEMKLSMRLRTYSSHAVVMYARGTDYSILEIHTGRLQYKFDCGSGPGIVSVQSIQVNDGQWHAVSLEVEGNYAKLVLDEVHTASGTAPGALKTLNLDNYVFFGGHLRQQGTKHGRGAQVASGFRGCMDSIYLNGQELPLNNKPRAYAHIEEWVDLSHGCLLTATEDCSSSPCQNGGVCNPSPTGGYYCKCSALYVGTFCEVSVNPCSSNPCLYGGTCMVDNGGFVCQCRGLYTGQRCQLSPYCKDDPCKNGGTCFDSLDGAVCQCDSGFRGERCQSDIDECAGNPCRNGALCENTHGSYHCNCSQEYRGKHCEDASPNHYVSTPWNIGLAEGIGIIVFIAGIVLLVMVFVLCRKMISRKKKRQAEPEDKRLGPTTAFLQRPYFDSKLNKNIYSDIPPQVPVRPISYTPSIPSDSRNNLDRNSFEGSAIPEHPEFSTFNPESMHGHRKAVAVCSVAPNLPPPPPSNSPSDSDSIQKPSWDFDYDAKVVDLDPCLSKKPLEEKPSQPYSARESLSEVQSLSSFQSESCDDNGYHWDTSDWMPSVPLPDIQEFPNYEAIDEHTPLYSADPNAIDTDYYPGGYDIESDFPPPPEDFPAPDELPPLPPEFSDQFESIHPPRDMPAAGSLGSSSRSRQRFNLNQYLPNFYPADMSEPQKQGAGENSPCREPYTPYPPGYQRNFEAPTIENMPMSVYASTASCSDVSACCEVESEVMMSDYESGDDGHFEEVTIPPLDSQQHTEV

>CgFat1

MGSIALDWWNRRKWLQMWVLLLVLHVPFTKGQVMSQDQFHFTSQLYNATIPERAPSRSYITASQKMGLYITDPTVDVSYRITEGDSSNIFKVEHKRVGDFVFLRIRTQTSSYGSLNREYISKYYLKVNAIGQVTQKDVLTAYTNVIIQVQDKNDLSPLFDKENYNVTVSEETGLHSTILTVSASDGDEGINAEVYYSLVSKTNFFAIHPSSGAVTVTRPLNFYEKPVHHLVVTAQDRGPKSIYSAVMQRNANITIHVIQANFNAPEIVVESFPNIKNTGSSGVVYAVLTVSDADYGKNGEIKEVSIFSDPSGLFKVLPSKDTQGEYHLLYTNSERQSSIFNNFYITIQASDSGNPQKVTRKKVHVHIEDIDDFGALFVADHYQTSVPEDLPVGASVYYLHPGVTNQRGETPPLTYSIISGNDKNLFMIREETGLIRTASLLDAEIDSSIELVIGAYNPKSIGSPHHGSATVTISILDTNDNAPVFKINETNVIFDENTPAGTIIYKVVATDADQGMNARISYSITNQETLPFEIDHFTGDIKLKQSLDYETMRRDFKTVVRATDWGSPFSRESEIHLYFHLQNINDNSPLFEKVNCSGYLSREAPLGTDIVTTPAVDFDYSDLTYYIKSGNEDGCFEVDASSGRLMLNCSLEAHTLDERQITIVVSDGKFDSDPVFVSLTLVNNKKNMQLSNKDANVQCKTTDAQERLLKILQMSSHNNNDGPTSGPILAASVTSNEHAPQFNSTLSKMLEISENAAVGTSILKVGATDADTGYNGLVQFSIIGGNPLDQFEMNPMSGDLIVISNLDREHIPKYDLAIQISDMAEPSLRKSAVANITVSLKDENDNAPKFEKDHYEASVFESILANATVVQVIAIDLDIGKNGEVVYSLAKDEDKFCVDSKSGIISVKKSLDREVQSVYYLPVRASDLGDKPLSSTATVKVILIDVNDNVPKFVPENYDIKIQEDIPVGTVITTIKAEDRDEGENGRLTYKLIYGVEDMFEIDGDTGVIRLIKPLDFETKQVYNISAHAEDGGNPSMVSACFINIEVVDVNENYEPPVFDDIVGYGRVKENIPVGSQVMIITARDPDADPAEVSMATPVTYSIREGSGLGYFSIDNEGIIRTTRVLDCESASHYWLTIYATDRGLVPLHARHEVYIKVTDVNDNIPQMRQPVYIMNVTENSGEGTVVGQVQAYDDDVTSQQELSYSIAEMDSWSFFTINKFTGVITTTRKKLDREREDRHTVQVVVTDSGYPSLSSTAHVVVEVLDVNDKKPEFLEKSYRSRVMAVTEDKGKTPLLRVTARDEDIGQNAQITYKLKGKKQTFGIDADTGMIYAKGLLSVGSYALKVQASDNGKERKKSTARVRLEVLPVPTSSENPPQFPVAFYQVQLMENCKIGEVLDVYQAEDADGDKLWYSLADGDPENQFNINPEYGSILTSKRLDREKRDHYNLTVMATDGIHQTSVKVYVTVLDINDNTPEFTQSEYKADISESAAIGSSVLTISAVDIDLNSRVFYKISGAANAITMDLFEIDSETGVISVKGALDREVMTQHKMTIMVRDQGFPSNRNFTRVTVNVQDENDHPPQFVSDVTEASVFETSAIGTSVVQLMAIDQDKGSNAEISYSVVSDVGNIDMAFALDSTLGIISVAKNLNKRERSDYSIVVKAADKGTPALSSTATINIHITMSNDAPPKFSQQKYVTDVMESLSLGSPVVTVKADSQSSVNYEIIKGNDNATFSVNPNSGVVYLKGRMDYETLDLYKLTVLATNNVGKFDMTSVWIHVLDANDNEPYFTKTFYTGSLSENSLPESLVLGIDGKPLVITARDDDTNENAQLVFEIVGDEAKEFFTISKGTGALKSKVSIDREKFSRFEFLVHVTDSGNPQLHALEPARVVINITDVNDNSPTFSLPEFQADILLPTAFDVEVITMTATDTDLGGNAKIIYTIKDGNKDGKFKIDRNTGTIYVVDDSNMLSKYQLTIEASDGKFESTTSLVLTVTQASDDFNFLLHHDEYNLFIHENVAVPQSLTVLQVNSALNQPIVFSLLNGKGMFDVVKTSGVLQTTGKPFDREERDHYKVIALVEDLSGKQKPLHILIHISILDENDNAPLFVNQPYDTVVSTDTKQGSVVTQVTAIDRDADLNGNVTYTLEKQDGDKFEVDAGTGHVRLITALSDQDVDQLYSLQIQATDKGHPSQSSEVTLQIRVVSNSHPIFDKYYYSVDVKESSPAGTAIMSLKAISPNNQKLIYSITDGDPYKEFTVDFNIGIISLTTPLDYESTKVYSLTVRATDVLSGSYGETKLQVNVLDVNDKSPSFTHYKYTKIISEAAPVGTSVLKVTATDADSGINAEIQYDLSPLDNSRSINIFQINPITGVLRTARLLDFEDTQEYLFHVVATDGGLPSLNMSVPVHIIVSDLNDNPPYFDQPTYSGVVTNPTPGLMVIKVTASDLDKCSQENLEYSIVGGNGDQVFRIDAKSGLISISPRRHSILYPAYMLNVSVSDGVFTSFTTVSISFEESNLHSPVFSKVLYQANLMENVGRGIPITTVKAQDGDRGYFGMISYSVLSHEMRQFFNIDADTGEIFAERLFDREEQSSYSVTVAATDNGGRMGFTTVVVNITDVNDVAPEFLHTGYKVSVPMNAGKGRPLIQVQARDSDLGEAGRVTYNIYDASLIISATLFTIDPTSGIVSVKADLSEKVGSVYQFFVEAVDHGSPQLKSNAPIEIYISNDNQPAPLTKPEHTFMITEDKRVGDIVGRVVKVTESLVDFSIVSGFTKDRNSPETVSINQEGEIFLLDLWRIQKLPEYKFTVVISLRENPQVVAHSQINILVQNIKSALPHFETSLYAISVAENQEKGTSVVQCRLLSEGKFITNAQYKFDHQTIMKYEHLFEINKYTGWITLVSELDREKQEVYNLTVQAEQKFGMSTQVVSNSTVTVTVTDCNDNPPVFSQAQYQTAVNEDALSGTVFLALKTTDADAKSNVDYFIADGDPMGRFKIHKNGDIYVTKPLDREMVPNYVLTVAASDGSLVSTAKVNVEILDANDNAPVCDQPIHQHVLSEDIGTTTIITRVKATDVDSMYTSNEKIHYIIKGEENNKLFTINREKGILTPKVELDRETQSHHHVVVSAVDGGGLSCDVDIYIELSDVNDNAPEFVDLPEKFGLLESATVNTLLLRITAEDRDLGINRQVRYGMTGDETFSIDQDSGIIKLMKPVDREEIPQYILNVYAYDQGIPSLTSTATLTIEILDENDNPPEFVLSQYAAAVPENATVGTEVVKVLATSRDIGLNAKMAYSITAGNKNMKFVIDESEGVIRVANDLDREVTSVYFLTVMATDLSDSPLSSSTYVRINITDVNDNKPFFSQASYRTSLLEDTAVGQLVYQVNANDTDEGQNALLTFSIIEGDSLHSFEIHPVTGEISIRTPLDREMIDKYKLVVEIRDSGLPPLSSTAVVTVDIEDVNDCPPLFSESSYNATVQRPPTGIEDQYVGYKILHYIVTDNDLDPNGPPFTFDIVSGNEGQEFRIEPTGILSTAGKFNRDILDLYTLTVRVFDNGTPNLYSDIVTTIHVIEEGMNPPEVKDLDISISSYDDNFPGGVIGQVDATDKDLFDSLSYQVVSPNRHLFDVGINDGRIIAYPGLDSGSYIVNVSVSDKKNTAYGIVKVDVLLISTAMLDSSVTMQIQNLSPKDFLVNYQREFQKAVKKALNVRANDVQIINVQASGNLGGAPRLKRSADKNDLDVLFAVKRSASGAYFRRRKLERKLRQNLNDIESELNVRIIKVFGDVCPQESCRDGQCIGVINFNKSLATLTVDGKSFVTAKHSYSFKCLCSNGIEDVFCPEVHDSCLTKICPPHQVCRHEPYQTPSCVCPEGQAGQFCESEDRCTDASCLNGIKPMTFSGNSFAKWTLMDEFTIEKRLSLSMRIKTHRSTASLMFATGDVDYSILEIYNGMIQYKFNYGSGAGLVMIPIHVDDGKWHTINVERNDKHAELTLDNKYTNMAIAPGKSRVLNLLNNDVYFGADVVIGYNGHPDVRRGFDGCMEEIKLFSIVLPLKGRNAVVKSQEFQEVEFHCKNTLSPPMGSNICSVVTCSNGGVCKPYSQYMGYEQYTCECPDRFEGNQCEIDLDPCANNPCGGNGECLNIENVPNEFRCRCKGNYQGQFCRYGKFCHSSPCKNGGSCIEGPSSFICHCPPGFTGLECEKVWSEIPGTKFVESNIESNESGITNTTLYIIVGVTSGLIFLAIVFVSIQCYRRRSSRTGRNVTLGIAFDDERDVMLKSRKDNCKVSDFDVSKFNNVPPSPQPPPVPNRPASYTPSNHDSLNTLNNLDNFHNYGSAADDLENCHNIPYLTQEFLSFHGPPPRSNASVAPSLPPPPPSNPPSDTDSIQKATWEGDCPNMLENLEEKKHPEKFPRLNRPTMLCADTTSFSSLPVSESEDEPAARCRKKGKDYHWDASDWAPRPSLPNISEVPIREIQDSPSSSPHSNESNTHIGFSGCQINDSALYTDPELLESEYVGDSEFADNEYDNEECYMSPPNYRQVLGLPQTPEEEEESYELPQHQINTHPNQYLPNHSFTQSHDQMSGDRDSRCEWPEPPSCDENEFYTSDNDEEVVSYGFPNTKKLVPGFVSDGGITDSEYNVRNSVIDRMSMSLGGYASTNASMSEISGLCEIEDSEVNLSEEESCDDNIDENTPLNNILERNTNV

>DmFat1

MFTMKIKKYVTPVKRKAFTILQWISLLCSLWLIPTVQSKADEKHTATLEYRLENQLQDLYRFSHSVYNVTIPENSLGKTYAKGVLHERLAGLRVGLNAEVKYRIISGDKEKLFKAEEKLVGDFAFLAIRTRTNNVVLNREKTEEYVIRVKAHVHLHDRNVSSYETEANIHIKVLDRNDLSPLFYPTQYTVVIPEDTPKYQSILKVTADDADLGINGEIYYSLLMDSEYFAIHPTTGEITLLQQLQYAENSHFELTVVAYDRGSWVNHQNHQASKTKVSISVKQVNFYAPEIFTKTFSSVTPTSNPLIYGIVRVNDKDTGINGNIGRLEIVDGNPDGTFLLKAAETKDEYYIELNQFAHLNQQHFIYNLTLLAEDLGTPRRFAYKSVPIQIKPESKNIPIFTQEIYEVSIPETAPINMPVIRLKVSDPDLGKNALVYLEIVGGNEGDEFRINPDSGMLYTAKQLDAEKKSSYTLTVSAIDQANVGSRKQSSAKVKISVQDMNDNDPIFENVNKVISINENNLAGSFVVKLTAKDRDSGENSYISYSIANLNAVPFEIDHFSGIVKTTSLLDFETMKRNYELIIRASDWGLPYRRQTEIKLSIVVKDINDNRPQFERVNCYGKVTKSAPMGTEVFVTSAIDFDAGDIISYRLSDGNEDGCFNLDPTSGSLSISCDLKKTTLTNRILKVSATDGTHFSDDLIINVHLMPEDLGGDSSILHGFGSFECRETGVARRLAETLSLAEKNNVKSASPSVFSDLSLTPSRYGQNVHRPEFVNFPQELSINESVQLGETVAWIEAKDRDLGYNGKLVFAISDGDYDSVFRIDPDRGELQIIGYLDRERQNEYVLNITVYDLGNPTKSTSKMLPITILDVNDNRPVIQKTLATFRLTESARIGTVVHCLHATDADSGINAQVTYALSVECSDFTVNATTGCLRLNKPLDREKQDNYALHITAKDGGSPVLSSEALVYVLVDDVNDNAPVFGVQEYIFKVREDLPRGTVLAVIEAVDEDIGPNAEIQFSLKEETQDEELFRIDKHTGAIRTQGYLDYENKQVHNLIVSAIDGGDPSLTSDMSIVIMIIDVNENRFAPEFDDFVYEGKVKENKPKGTFVMNVTARDMDTVDLNSKITYSITGGDGLGIFAVNDQGSITSLSQLDAETKNFYWLTLCAQDCAIVPLSNCVEVYIQVENENDNIPLTDKPVYYVNVTEASVENVEIITLKAFDPDIDPTQTITYNIVSGNLVGYFEIDSKTGVIKTTERKLDRENQAEHILEVAISDNGSPVLSSTSRIVVSVLDINDNSPEFDQRVYKVQVPSSATVNQSIFQVHAIDSDSGENGRITYSIKSGKGKNKFRIDSQRGHIHIAKPLDSDNEFEIHIKAEDNGIPKKSQTARVNIVVVPVNPNSQNAPLIVRKTSENVVDLTENDKPGFLVTQILAVDDDNDQLWYNISNGNDDNTFYIGQDNGNILLSKYLDYETQQSYNLTISVTDGTFTAFTNLLVQVIDINDNPPQFAKDVYHVNISENIEEESVIMQLHATDRDEDKKLFYHLHATQDPSSLALFRIDSISGNVIVTQRLDFEKTAQHILIVFVKDQGAPGKRNYAKIIVNVHDHNDHHPEFTAKIIQSKVPESAAIGSKLAEVRAIDRDSGHNAEIQYSIITGNVGSVFEIDPTFGIITLAGNLNINKIQEYMLQVKAVDLGNPPLSSQIPVHIIVTMSENDPPKFPTNNIAIEIFENLPIGTFVTQVTARSSSSIFFNIISGNINESFRINPSTGVIVINGNIDYESIKVFNLTVKGTNMAAESSCQNIIIHILDANDNIPYFVQNEYVGALPESAAIGSYVLKVHDSSKDHLTLQVKDADVGVNGMVEYHIVDDLAKNFFKIDSTTGAIELLRQLDYETNAGYTFDVTVSDMGKPKLHSTTTAHVTIRVINVNDCPPVFNERELNVTLFLPTFENVFVRQVSAKDADNDTLRFDIVDGNTNECFQIEKYTGIITTRNFEILNNENDRDYALHVRASDGIFSAILIVKIKVLSAIDSNFAFQRESYRFSAFENNTKVATIGLVNVIGNTLDENVEYRILNPTQLFDIGISSGALKTTGVIFDREVKDLYRLFVEAKSMLYDGMNSNVRRAVTSIDISVLDVNDNCPLFVNMPYYATVSIDDPKGTIIMQVKAIDLDSAENGEVRYELKKGNGELFKLDRKSGELSIKQHVEGHNRNYELTVAAYDGAITPCSSEAPLQVKVIDRSMPVFEKQFYTVSVKEDVEMYSALSVSIEAESPLGRSLIYTISSESQSFEIDYNTGSIFVVNELDYEKISSHDVSIRATDSLSGVYAEVVLSVSIMDVNDCYPEIESDIYNLTIPENASFGTQILKINATDNDSGANAKLSYYIESINGQNNSELFYIDVTDGNLYLKTPLDYEQIKYHHIVVNVKDHGSPSLSSRSNVFITVKDLNDNAPCFVEPSYFTKVSVAAVRGQFVALPKAYDKDISDTDSLEYKIVYGNELQTYSIDKLTGVISLQNMLNFTDKSSTVLNISVSDGVHTAYARLKISLLPENVYSPLFDQSTYEAQVPENLLHGHNIITVKASDGDFGTYANLYYEIVSEEMKKIFLIDQTTGVITSKVTFDREKKDEYVVLLKVSDGGGKFGFASLKVIVVDVNDNVPYFLLKEYKMVVSTTVEANQTILTVKAKDDDIVDNGSVHFQIVQKSNDKAVKDVIEINEKTGDIVFKSKAESYGVNSYQFFVRASDRGEPQFHSEVPVSIEIIETDANIPTFEKSSVLLKIIESTPPGTVLTKLHMIGNYTFKFSIAADQDHFMISDSGELILQQTLDREQQESHNLIVVAETSTVPVFFAYADVLIDVRDENDNYPKFDNTFYSASVAENSEKVISLVKVSATDADTGPNGDIRYYLESDTENIQNIFDIDIYSGWITLLTSLDREVQSEYNFKVIAADNGHPKHDAKVPVTIKIVDYNDNAPVFKLPIEGLSVFENALPGTVLINLLLIDPDIEKQEMDFFIVSGDKQAQFQIGKSGELFIAKPLDREQLMFYNLSIIATDGKFTAKANVEIDVKDINDNTPYCLKPRYHISTNESISIGTTLVEVKAIDFDFQSKLRFYLSGKGADDFSIGKESGILKVASALDRETTPKYKLVAHVQDGKDFTQECFSEIIITVNDINDNMPIFSMAQYRVSVPEDAQLNTLITKVHAMDKDFGVNRQIKYSLMGENHDYFKISKSTGIIRLHKSLDRETISLFNLTVKAEDCGVPKLHSIATVAVNILDINDNPPEFSMRQYSCKILENATHGTEVCKVYATSIDIGVNADIHYFIMSGNEQGKFKMDSTTGDLVLNATLDYEMSKFYFLTIQAIDGGTPPLSNNAYVNISILDINDNSPTFLQNLYRINVNEDIFVGSKILDVKATDEDSDVNGLVTYNIERGDNIGQFSIDPKNGTISVSRPLDRETISHYTLEIQACDQGDPQRCNSVPININILDTNDNAPIFSSSNYSVVLQENRLLGYVFLTFKISDADETPNTTPYTFDIRSGNEGGLFRLEQDGSLRTASRFNHNLQDEFVIQVRVFDNGTPPLYSDAWVVVKIIEESQYPPIVTPLEVTINSFEDDFSGAFIGKVHASDQDKYDELNFSLVSGPDDMYQSSKLFNISNNTGKIYAISNLDIGLYKLNVSVSDGKFHVFSIVKINVELVTNDMLKESVVIRFRRISASEFLLSHRKTFMRSIRNIMRCRQKDVILITLQSDYQKASQHAVGNRRARSIDSDLNVVFAVRKQQIIPDSDEFFTSDEIRQTLIDKKNEIENETNLVVEDVLPSTCQSNKNDCVHGECKQILQILKNNVTTTFTDVISFAAPSYIPVNTCVCRPGFDGKHCKETVNACSTDPCSPQRICMPSGSALGYQCVCPKGFSGTYCERKSSKCSNESCDMGLFTAVSFGGKSYAHYKINKVKAKFTLENGFSYSLQIRTVQQTGTLLYASGKVDYNILEIINGAVQYRFDLGSGEGVISVSSINISDGEWHQISLERSLNSAKVMVDNKHVSHGSAPGVNGILNIQSNDIFVGAEVRPHPSIIGYEDIQRGFIGCMANIKIAKESLPLYISGGSTIAALKRFTNVEFKCDPSNVLVRLGICGSQPCANSGICKELDTDVFECACQPRYSGKHCEIDLDPCSSGPCLFGGRCDYHGPNNYSCTCPIHLSGKRCEYGKFCTPNPCKNGGICEEGDGISHCMCRGYTGPTCEIDVDECENQPCGNGATCINEPGSFRCICPSYLTGASCGDPLYSNSISTKLKNFSIEHISGIISGVAVVLVIISCVLCCVVLKRSSSSKRRNRLEKDKNKSSYKEANLNSLVDKDNYCKPNVKLSNLEVNQRPISYTAVPNDNLVLSNRNFVNNLDILRSYGSAGDELENVPFEYQKVNRNKQHVNINSCHSTDADNAYKQEWCEQMHLRTFSENKLNNELKRDFGPSVSRFSTGKLIQVEMPNVCHSSSANFVDYSALANGQYHWDCSDWVRKSHNPLPDITEVPGAEIADSSSLHSNDSNESKSKKAFFVHREDGDVDPTRDIAALNEDIGSEYLDSEAESCLEPFMLPRSSNQPLSRLSSFNNIENEDYKSNTVPLPSKVSHSCKVYLRHPDSYLPTMHFPSETDGESSMTEGPISRMEIKTRRTISENSEEAYLFPCTVGEIGSNSNISVRLCEIEDSELEEFLPQQQTNN

>SpFat4

MPGALVLYLTLLCIFPGIAKADDRATTNDINLFVDENQPNGTYVGSVAEEGATSNSYSFLSTPPDEFHLNSTTGIITTADVIDRELLPNDLFILPVAVVTSSGTNIVDIKITVNDLNDNSPVFPQSVISLSFSENAHSGSVQILPSAMDADKDSNGYISTYLIESVSGGGDDAFLLQTFRPREDAEYTVSLVTNSLLDRETEDFYILNISATDNSSTPRTGYLIVNVTITDVNDNSPIFTQTTYFATVNESSAAGTSVVHLNATDIDQGSNGDIVYRLKNELTDNFALDPDTGWITLLRQLPYVANGYLFEVTATDKGSPSQSSSAFVNVMVEDENDHSPVIEVLIPEIEASGVASISETMLPGPLFNLKAIDEDREENGRVSMSIHSGNDRNDFSGTSISLPTGDVFFLLRVSPSANIDREIIASYNLTFAAEDMGTPPRRTYRSIIIYVVDENDHAPMFLQESYRAGLLESIQVGSFVKSVTATDADEGINADIVYSITGDEYGWFQIDNGTGLVTTREKLDHETAAQVTLIITASDQGLEPMANTTILTIDIQDVNDEYPVFNQSSYSETVEENSPARDLIQVLATDLDNGVNGDVTYTFSSDQYSGMFSLDPESGLLQITVPLDREEQDSYELEILASDGGSPSLQATATVTIAVLDQNDNPPQFYPTEYYASVIENGPAGLFVTVVSATDLDVGVNGAVIFSLSDSSKFQIDSYSGNVTTRESLDREQESSYTLTVTAQDGSGQAAAQSATIFVTVTDTLDNPLEFELNPYRFQLEENRPNRTEVGTVLATSMDLNVEITYVIVNGDPNGLFVIDFYSGVIKTTLSIDREAHESASFRLTVLAVDGSQNGQTVVEIEILDINDNAPVFALPEDQVDVVENWAVGNKFYAAQVTDADSPPNNVILYDLSVNFDDRFGINHTSGVLFLNKDLQNYPEKEYNLQIQATDYGTPPLSSSMNLLITVRDVNNNAPVFVDSINTTLRLTESIPVNSIVIHALATDADQGTNGLISYRITSGNTDSFGIFPDGMVYVKRALDREQVDQYDLTIEATDGGVPSMTSTLMLEIIVLDENDNRPFFDNATYNFYLSEEDDIGAAVGTIHAVDNDVGGNGELTYSFTANHTLFTLNSVTGAITSKVSLDRESLVEAGQPTTFSIRVRVHDSGQPQLQDHADVNIHVVDINDSPPVFNRESYQASVSELAQNETLIIRVSATDDDVGDNSYILYSIVSGNEEKRFHIDHVHGQIVLIAPLDREVTEGYVLRVKARDGGIVPQESFVEVEITVLDENDHRPSFSVDLQREIEVTECLQIGDTIGGVQATDMDINSNGQISYTITGGNLDGVFGVNANSGEVYLTGYLDHEATSQYSLNITARDGGSPSLQAVVSMVINIRDCNDNSPIFAYVGVPEIDEEKPEGFEVVRVSASDSDSGVNGEVRFSISSQRPSGDFFAIDPESGWITTNSRIDRESEIMATDQFILTVHGTDLAVPVESRRTATVDVVIFVRDINDNAPRFTSQQAAAISRSTSANTRVTTVHADDPDTGVNGQVTYTLISTGVPFRLDLITGELTLTQPIPSSANIYSLDIRADDAGSNPQQQSTPFSLAIIISDGQSSILNYVQNTYTGSVRENQPAGTEIVTVVAQYSDGRSATVRYYVSAVTAGGVDKGSLIRASVTNGIIRTDAVLDREDLGGGSSLVATVYAVDTSSSSSHVTSVQVTVNVLEVMATDADLDTIIFYSIVDGATDLFSIDSTTGQIRTRGVVDRESSAVYQLQLQASDGTLTSTTTIDINLLDENDNDPVFSLSTYSFLVPEDEMVGEDVGVVVATDPDAGLNGEVAYSVVEEGAPGEDVFYLDPATGAFRLQQSLDYEVKQHYFMTVMATDKGSSPRSSTATVYINVEDVNDNNPVYNPVDYSEEVPEDVAIGTSVVTLTATDLDSGSNGELQFSIASGDPSQRFTVYPNGTIVTIKNLDREPESFYNLEVVASDMTLDPEDRRSSTAQVSIIVTDINDNAPRFTNPDSVEIAEDTRTNVIIMVLRAEDDDVERNSYVEYYLLTPGVPFAVSRVEGNMQVTGPLDRETESSYSLLVQATDKGTPPQSSTMNLTIYITDVNDNAPAFSMTPYEATLDEDTPVGLEFLKVTASDPDEGLNSIIRYSIFSGDSGRAFAIDPVTGVISVNAGLNYEQRSSYTLTVRAQDQGYSTQVSSVTVTITVADVNDNAPVFEDSYAPSIMENNLPNADIVQVRADDADSGVNGMINFRLESDYDGLFTIGSTSGLIQVTNVLDYEDQNEFELIVIAEDSGTPSHQTRTTMTLTVNDDNDHDPEFVSDFYRATVSEDASADTFVIQVTATDVDATAALRYRLNSDIGSKFTIDPLGGRILTTGLLDREVQSSYLLTVTVDDGPSRLATTSVRVEVLDVNDNPPRFSPTSYTATLPATSQQGCFVAAIQATDTDLEDNAAIRYSITSGDTSKFVVDQDTGVVITAQGFTDENSPYDLQITAENVASGQNSAVASLRVIFSTASFPDITFPFSGTSSSYGENVMTGMTVTTVTAIGIVTYSIAGGNHGEHFGVGSQSGEVSIIKELDYEESKSFSLWIAATDTANTQLSDFVELQVSVLDVNDNAPLFDQSVYFTSVVEEQSGQVQVVSVSATDEDSLSNGDVSYAITLGNTNNAFDISSSGVITTTQPLDRETVASYILTVEATDGGNPSQSGSAIVVVTVTDINDNRMSFTKMYDATIPEDAPPGTHVVTLHTTDPDENNLSQFSIEAGGQSSLFAINPITGEITLNSSLDFEDEAVHYLLVKSFDPDSSHEVQTQVLVSVEDTNDNAPVFTENSINTTYPEIFFSSGNVIATVSATDADDGDNGQVDYILKTMTEHFRVETINNRGWIFPVPPISYIVPSPEDTSNPNTYTFSVFAVDRGTPALYGEASVIITVTQDNSYTPVFATPSYFSPVTANTRSNTRVLQVVAVDQDTGSNAAITYSISDGNGTEKFDVEADTGWILTKGVSLAGDVGITYQLLVKATDNGNIKKEDETTVTFLITDSNDNAPVFDDDTYRSSIAEDVGGYVATVRAEDDDSGINGEITYSISGADAALFSIDAVSGIVSLVGSLDRETSDEHQIQVTAEDRAMYSQSATAILIVTVTDVDDNPPFFLPREYRADVFENSPSATPVVTVTATDADTGTNADFEYIISGGDGTDFFTINPETGLILTQGNLDYETGKTTYHLTVAATNEQATMVDYAHVIVTLLGENEFYPQFTQHQYDFYVNEHATDGAPVGVVLATDDDLGDDGIVNYLFIGGSNLQGFNVNLESGQITVAYENGRLDRETADTVLLSVLAKNEGPITGADIDEAEVIIHVNDGNDAPVFSSEQYQARVLESEPPGSDVTVVTAADYDEIPSFRQFNYSILRGNEGDAFHIDAQTGRITTTSLLDRETLSVYFLTVAAIDTGNPPQTGTAVVSVELDDVNDNGPVFIGDAAEGSVFENEPPNELIMTLRATDPDSNPDPSQFTYTLLTSPDSSAFRLVGDELRTTQTLDREVKSDYYVQIEASDGESPAMSATSTIHITIADRNDNPSTLRQARIEVKMFQSMFPGGVIGSVKPIDPDTDDTFFCQITSGDLSKFSIQSNCDLFSASHSTESVVDLSVSGNDGSHQSVTSSFSVVYESFTNDTLTNSVTLRLADTSAEAFLANSYDRFKTSLSAFLGSRETLIVLSITDHPDLDKVDLVLAIKKQGSQHLLHDDLVDLLEANEATLESQSDITIETIDYTACSDSPCLNSGTCSHETNINLEELITESDPVIFVGLQTSHSFTCACPAEFSGQRCEIPTDYCGQATCKNGATCQNAIGGYICVCAPGYNGEDCKVEINECSSNPCINSECQDLINGFYCECSTGYSGVYCENGPCSTSPCVNGGSCVESGSTFVCQCDNSHWGNRCQYDTIGFQAGSYISSSALQSTSAVILLEFSTVSTKALLFYNHDSFTDSNAKFVALEILDGRLQLSFNFGSGQNSISASKPVSDGDWHKVEVRLEGMSVDLNILDEDCPSLSTDACRASQETTISLAFDNNPLTVGGVSDIAEITSRASQVSAADFVGCMNSIEVNGERLSMSNARDHSNLVEGCIREGCGETSCSDGSVCVDDWWKTWCDCDEMASGATCAEDNNSVSFGGGGRVDYMVKEDYKRQALLDDAKVLTRRRRRRASGSETVSFSFRTGVKDGLLLYVTSDAEFTALQLSNGSVMYSYGTGSSSSGQIINIPSSSLTDGAWHNITLTSSGGQVSLTVDNSPKSAMFADPHEFTGLGLTGMALGGAETPLVINGRSIEGFTGCLDGFQMNGEGLPLDGDSQRFEAVPSAGTGEGCSPADLCALNPCSQGEACVPSETYYTCVPVTCVPDVCLNGGVCSDGTGSVTCECAEGYSGDYCEGGTGPREGEGVNVIYIVIPIVILLLLVVFIVAAVFFIRRRSGNMKRKQANQQAALQNPKVSVLSSSVNPGFLADTFDDATMIGPDGKVNVSGMIHRQTPDIIEQNIMQQNNSNPSSNSLMMENDTGEHVKFEMENLRPRELDEAEHYDLENASSLAASDIDVAYHYKHFHDQGNRNRRKKKHRQNQNPMLARIQASPARLSPVSIGSHHNPNPLGSELAHSTPLDSQNAMLRRSPGLHSNYSANPTLRMGSIPSSGRATPARNISSPVNSDRSFQSELRSQAHRSDVSSLRSGGKPARMTTPLSHDGKSQPHSKGRTSKSPLVVGLTAEEVAQLNTARPDLMSGSHASTIEDLSSNSSRVHGGERPIDTPCDPSRLLEPPDSTSDDTNDSFTCSEMDSEYGKHGGFNSTEAAILDRLAEIEHAEDSVLPHVNGALKQKRLDSRGGSLSTLFTSEDENGHSRKEKTNGDVSLERLLGWGPRFDNLVGVFKDIAQLHESNGKIVLQSSLTQEEFV

>LvFat4

MPGALVLYLTLLCMFPGGAKAQNRATTNDINLFVDENQPPGTYVGSVAEDGATSNSYSFLSSPPNDFEFQLNSSTGIITTTVRIDRELLSNDLFILPVAVATSAATNIVDVKITVSDLNDNSPVFPQPVISLSFSENADSGSVQILPSAMDADKDSNGYISTYRIESVSGEGDDVFSLQTFRPREDAEYTVSLVTNSLLDRETEDFYILNISATDNSSTPRTGYLIVNVTITDVNDNSPIFTQTTYVATVNESSAAGTSVVHLNATDIDEGSNGEIVYRLKNEQSDNFALDPDTGWITLLRQLPYTSNGYLFEVTATDKGSPSQSSSAFVKVMVEDENDNSPVIMVLTQDIEASGVHQVSETIMPGPLFTLRAMDDDRDDNGRVSLNIYSGNERNDFSGSSIPLPTGDVVFILRVSQTANIDREITASYNLTFVAQDMGTPTQTTYRSIIIYVVDENDHAPEFLQGSYHALLSESLQIGSFVESVTATDADVGINADIVYSITGDEYGWFQIDNKTGLVTTKEELDHETAAQVILTITASDQGLEPMTNTTTLTIDILDENDEYPVFNQSIYSETVRENSPGRDLVQVLATDLDNGVNGEVTYTISSDQYSDIFSLDSESGLLQITASLDREEQDLYELEIIASDGGVPPLQATATVNIAVLDQNDNPPQFYPTEYYASIRENEPAGLFVTIVSATDLDVGVNGAITFSLSNSSKFQIDSRSGNVTTLESLDREQESSYTLTVHAQDGGGESATQPATIYVSVTDTLDNPLEFELNPYRFQLEENRPNSTEVGTVLATSMDLNVEITYAIVNGDPNGLFVIDFYSGVIKTRRSINREAFESTTFRLTVLAVDGSQNGQTVVEIEILDINDNAPVFALQEDQVDVVENWAIGYEFYAVQVTDADSPPNNLILYDISMNFDNRFGINHTSGVLFLSKDLQSYPEKEYNLQIQATDYGTPPLSSSMNLLVSVRDVNNNAPVFADSINTTLRLTESIPVNRIVIHALATDADEGTNGEITYSIASGNTADSFGIFPNGMVYVKRALDREQVDQYDLAIEATDGGVPSMTSTLMLEIIVLDENDNRPFFDNATYNFYLSEEAGVNAFVGTIHAVDNDVGKNGELTYSFTANHSLFTLNPMSGAITCKVSLDRESLVESGQPTTFSIRVRVRDGGQSPLQDHADVNIHIVDINDSPPAFNRESYQASVSELAQNQTLIIRVSATDDDVGDNANILYSIIGGNEERHFHIDHVHGQIILIAPLDREVTDNYVLRVKARDGGTVPQESFVDVEITVLDENDHRPSFDSNVQREIEVTECLQIGDIIGSVRATDMDINSNGRVSYTITGGNLDGVFGVNSNSGEVYLTGFLDHEATSQYLLNITARDGGSPSLQAVLSLVINIRDCNDNSPIFAYVGVPEIDEEEAEGFQVVRVSASDSDSGVNGEVRFSILSQRPARDFFAIDPESGWITTSSRIDRESEVIATDQFILTVHATDLAVPVESRRTATVDVVIFVRDINDNAPRFTSQQAAVISRSTSTNTKVATLHADDPDTGVNGQVTYTLLSTGVPFRLDLNSGDLTLTQTIPSSVNLYSLDIQAQDAGSNPQQQMTTFTLTIIISDGQSSVLNYVQSSYSGSVRENQPSGTEIVNVVAQYSDGRSATVRYYVSGVTAGGVNKGNLIVASPTNGIIRTGDVLDREDLGGGSSLVATVYAVDTSSSSSHVKSVQVRNMENVVTNSPVKTVMATDADLDTIISYSIIDGATNLFSIDSTTGQIKTRGVIDRESSAVYQLQLQASDGTLSSTTIIDISLLDENDNDPEFSMSIYSFLVPEDELVGEDVGVIIATDPDAGSNGEVVYSVVEEGAPGEDVFYLDPMTGAFRLQQTLDYEVKQHYYLTVMATDKGSSPRSSTATVYINVEDVNDNDPIYNPVYYSEEVPEDVAIGTSVVSVTATDLDSGLNGELQFSIVSGDPSQRFTVYPNGTIVTIRNLDREAESFYNLEVVASDMTLDPANRRSSTAQVSIIVTDINDNAPRFTSPDSVEIAEDTRTNVIIMVLRAEDKDVERNSYVEYYLLTSGVPFAVSRVEGNMQVTGSLDRETESSYNLLVQATDKGTSPQSSTMNVTIYITDVNDNAPAFSMTPYEATLDEDTPIGLEFLKIIASDPDEGLNSIIRYSIFSGDNGQAFDINAVSGVLSVNAALDYEQRSSYTLTIRAQDQGYSTKVSSTTVTITVTDVNDNAPVFQGSYSPTITENNLPNADIVQVFADDADSGSNGMVNFRLESDYNGLFTIGSTSGVIQVTEVLDYEDRNEFELIVIAEDSGLPSHQARTTMTLTVNDDNDHDPEFVSDFYRATVSEDASPDTFVIQVTATDVDVTADLRYQLNDDIGSKFTIDPLGGRIITSGVLDREVQSSYILTVTVRDGSSRQATTSVHVEVLDVNDNPPQFSPTSYTATLPATSSQGRFVAAIQASDADLQDNAVITYSITSGDTNKFRVDSNTGVVFTKQDFTNENNVYNLQVTAENVASGQNSAVASLRVEFSTASFPVITSPNSGTSSSYSENVQTGMTVTTVTATGSVTYSIVGGNHGEHFGVGSQSGVVSIIKEFNYEEIQSFSLWIAATDTGNAQLSDFVELQVAVLDVNDNAPLFDQAVYFSSVGEEQSGQVQVASVSATDDDSLSNGDVSYSIISGDTDNAFDINSSGIITTTQPLDREMVASYILTVEATDGGNPSQSGSAIVVVTVTDINDNRMSFTRTYDATIPENAPPSTHVVTLHTTDPDENNLSQFSIEAGGQSSLFAINPITGEITLNSSLDFEDEAVHYLLVKAFDPDSSHEVQTQVTINVEDTNDNAPVFTENLINATYPEIFFSSGNVIATLSATDEDSGDNGKVDYILKTMTEDFRVETITGRGWIFPEHPISYITPSPDDTSNPNTYTFSVFAVDRGTPTLYGEATVIITVTQDNSYTPVFEAPSYFSPVTADTTPSTRVLQVVAVDEDTGSNAAITYSITGGNGTAKFDVEADSGWIFTKPISLTGDVETIYEVLVKATDNGNIRKEAETSVTFLITNSNDDAPVFDDDTYRSSIAEDVGGYVTTVYAEDNDDGINGEIRYSISGADASLFSIDAVSGIVSLVGSLDRETSVEHQIQVIAEDRAMYPQSATATLIVTVTDVDDNPPVFLPRVYAADVFENSPSATPVVTLTATDADTGTNADFEYVISGGDGTDFFTINPETGLILTQGNLDFEAGKTMYYLTVAATNEQATMVDYAHVTVTLLGKNEFYPQFTQHQYDFYVNEHATDGAPVGIVLATDDDLGDDGIVNYLFIGGSNSQGFNINPESGQITVAYENGRLDRESADTILLSVLAKNEGPITGADIDEAEVIVHINDGNDAPIFSSEQYQARVLESEPPGTDVTVVTAADYDIIPSFRQFNYSILRGNEGDAFNIDARSGRITTASRLDRETVSVYFLTVAAIDTGNPPQTGTAVVSVELDDINDNGPVFIGDAAEGSVFENEPSNELVMILQATDPDSNPNPSQFTYTLLNSPDSSAFRLVGEELRTTQTLDREVKSDYYLQIEASDGESPAVSATSTIHITITDRNDNPSTLRQARIEVKMFQSMFPGGVIGSVKPIDPDTDDTFFCQITSGDTSKFSIQSNCDLHSASHSTESVLDLSVSGNDGSHQSVTSSFNVVYESFTNDTLTNSVTLRLSDTSAEAFLADSYDRFKTSLSAFLGSRETLIVLSITDHPDVNKVDLVLAIKKQGGQHLLHDDLVNLLEANEATLESQSDIIIETIDYTACADSPCLNSGTCSHETNINLEELITESDPVIFVGLQTSHTFTCACPAEFSGQRCEIPTDYCEHATCKNGATCQNAIGGYICICSPGYNGEDCKVEINECSSDPCINGECQDLINGFHCECTAGYSGVYCENGPCSTDPCENGGSCAESGSNFVCHCDNSHWGNRCQYDTIGFQAGSFISRPALPSTGAVILLEFSTISTKALLFFNHDSLTNSNAKFVALEILNGRLQLSFNFGSGLNMIAATKPVSDGAWHKVEVRMEGTSVVLNILDEDCPRLSADACRASQETTISLAFDNNPFTVGGVYDIEELLSRASQVSTADFVGCMNSIDVNGERLSMSNAQDQSNLVKGCVREGCSESSCSDGSVCVDEWWKTWCDCDEEASGATCTEDVNSVSFGGGGRVDYRVKEDYKRQALLDDAKNLAKRRRRRASGSESLSLSFRTGVRDGLLLYVTSDAEFTTLQLTNGSVMYSYGTGSSSSGQIINIPSSSLTNGAWHNITLTSSGGQVTLTVDDAPKSAMFANPHQFTGLGLTGMALSGTEMPLVIDGRTIEGFTGCLDGFQMNDEGLPLDGDSQRFEAVPSAGTGDGCSPADLCALNPCNQGEKCIPSGTYYSCVPVTCSPDVCQNGGVCSDGSGSVICQCADGYSGDYCDKGTGPQQADGPDILKIVIPIVVVLLLVVVILAMVYLIRRRRGSMKQKQAQQQAGLQDPKVSALSSSVNPGFLGDTFDEATMIGPDGKVNVSGLIHRQTPDIIEQNMAMQQNNSNPSSNSLMMENDTGEHVNLEMETLHPRGFDGAEHYDLENASSLAASDVDVPYHYKHFHDQGNRNRRKKKHRQNQNPMLARIQASPARLSPVSIGSHNQIPLGNDLAHSTPLDNHNALLRHSPGLHSNYSTNPTLRMGSIPSSGRATPARNISSPVNSDRSFQSELRSQAHRSDVSSLRSGGKPTRMTTPLSHEGKSQSHSKGRTSKSPLVVGLTAEEVAQLNTARPDLMSGSHASTLEDLSSNSSQVHGGERPHDTPVDPSRLLEPPDSTSDDTNDSFTCSEMDSEYGKHGVFNSTEAAILDKLAEIEHAEDSVLPHVNGALKQKRLDSRGGSLSTLFTSEDENGHSRKEKTNGDVSLERLLGWGPRFDNLVGVFKDIAQLHETNGKIVLQASLTQEEFV

>ApFat4

MSSAVVILYRVTLLCMVFGLASGQGYETDFVFTVDEGQPPGFSVGTVSTEPGYTYKFSDAPAEFTINGVSGEIQTAIQIDREVLQNDVFDILVLAKNNVTLRTKPIEVRITVADLNDNSPSFPETDISVSFTENGRAGTTVILESATDPDKADNGSIVEYSIWEGNSENAFRLVTIPGPDGLPIYINLETTLSLDHEVQDFYQLTIAARDNGSPPRFGFVHLNITVLDINDNPPIFDQSEFVINVNESTPAGTKLLEVHATDGDSGINGEVEYFIEEETSNFNVEPVSGNVTLQQKLNYISSDPYQVTIKARDKGVPTLYGRAFLSVYVIDENDHDPQITFSYIPQSESFATVSEGSATGTLLALVTVTDEDLAENGEVNLQIISGNELDHFMLDTVQFPTQTQTKVFKILVVGDLDRENIPQYNLTLLAWDNGFPRRQTIGSLLIHVVDTNDHSPEFQESEYTASLSEGQPIGSFVQSVTATDADFGLNAMVVYSITSGNSLDWFEINPDTGLVTTKALLDREVSSVVELNISATDQGPQPRTSGTTLTVIILDVNDEAPEFSSSGYTASVPENLNAGFEVLSVTATDGDLGSSGFVHYAFTDDVTHQNPGIFSIDMNTGTIFTEAVLDHEAISEYDLQVRAFDSGTPPQESVVSVHIDVVDENDNIPIFYPQTYLVTVRENGPPVSSVERVTATDRDSGNYGTVTYSIVSGNIAGKFDVVGATGEVMTVASLDREQQSAYTLFINATDGGGRMAEISAEVHITVGDIQDNPPVFDQSVYEFEIYENVNASSYVGSVTASSQDLNAQIMYILFSGDPMSQFEIDRNTGVIRTTGQVDREVTPAYELTVYASVGNLIGSTTVNIQILDVNDNDPQFPLSRDTTDVVENWEVGRHFYRVQATDADSPPNGDVVYNIIQNPGETFGINHTTGWMFLNKDLQVSSQDEYSVKINASDLGNPSRSATLELIVSVRDVNNHAPEFAAQHFETSISETTEVNYRFFSLTATDRDQGTNGLISYSILSGNERDSFGVFPNGEVYVKNALDREDISEYALRVEARDSGIPSRSASTELVVEILDENDNRPFFENSSYTFYLQEGSALNTVVGTLVATDADLGENAQLTYSFVGNQTQFSLDRNTGVITNLVGFNREAENGQSSFTFEAVVRDGGAIPFQDRTSITVVISDINDNAPVFSRSSYQVTISELAENGTHVLRVTASDADSGDNSYIVYSIMSGNSEKKFQIGRTNGQITLVGSLDRETTSEYPLTIKAQDAGSNPQHSLAEVLVQVLDENDHMPVFTHVEAELEVMECLAIGQPITMVSATDADIGENGLISYSITGGDVHKVFRIDPDTGAITLSKLLDHETRSSYTLNITARDSGVPQQTSVTSILVSVRDCNDNSPVFPQHSVVKWIDEEVPLNTLVTTVSASDPDSGINGEVRYSILSQEPEGEHFSIDPGTGSIFTNARIDYEYLKVHNSFFRLVVKASDQALPQESRRSATTEVVIFVRNINDNVPVFQTHNAVVISRSAAEGTVVTTVKAEDADDEDSANVTYFIVNRSVPFEINTASGEVTVTGRGFEPNVHLYSVRIHASDSQLASNYTSLVLNILVLGESNNGPTFTRGAYSGSVSENQEEGTEVVSVSAGFSSDPDANIDYFITSVVAGGISRDGDFRMNPSGVISTGVVLDREMLGQDAVYVVTVYAISGTSAYNSTQVHITVLDENDSPPTFARPVYQVNISEEARSGSPVTRLMAHDADTGSNAQISYSIVSGNDGRFTIDRDTGQIWTSGQLDRETVATYTLGVRASDGRQFQTATLEVSVQDTNDNDPAFTESTYSFSVLENHAVGTVLDAVTATDPDAGTNGEVVYSIVAEGAPGEDIFSLDSLTGEFTLLTELDFEEKQVYFLTVRANDRGTVPRSTTASVYFSILDVNDNEPVFNPTSYWVEVAEDVAVGTEIVMVTATDADSGSNAEIVFTISSGDPTSRFTIYPNGSLSTIIGLDREMQSFYNLEITATDQAADLVQQKSTTAQVTVIVLDINDNAPVFTTGNHVSVREDTSRGDVVMFVHAVDADVEKNSYIAYSLSPVPGNVFLINSVDGNIRLLNDLDRESVPEYTITVHATDKGQPAQSSSMDIVIEVLDINDNNPIFVLDSYHSSLPEDVPVGLEFLQVSATDADEGINAELRFSIFNGNYDSDFTIDPTSGVLSVANPLDRERRLSYSLVVRAQDSGDGLRIASTTVTITVQDINDNAPEFLTPYTPRIQENNQLGALVTQVSATDEDEGTNGRLTYSIEGNSYDGLFTIVPDSGEIHVARSLDREVEDSYALKIVAEDAGSPPRQGSTDIVITVTDDNDNPPQFELPSYTVTLQEEVGSNTPVLQVSATDADSTADLRYRLDSAIGNKFTINPISGQILTTSGRLDREEQANYFITVTVEDGGTWTRQTTVRVNLQDINDNAPRFVIQSYQALLPATTPAGAIVLGVEAQDADIGDNAVLTYSITSGDTNKFAIDASTGVITTAQALTLESTAYILEVSVSDSSPTHAAATAMVQVTFSTSSQFPSFQSPSSGTVYQFDEDVANGTEVVTLQASSPKTGPEGTVTYHITGGNIENRFDLDAQSGLLTIARGLDYEVNQHYSLLLEARDGSSPPLTTSIQIVVNVNDVNDNFPVFDLAKYSGRVIEGQPAGTSVIQVQATDADSGTFGNIQYALRFDGNTNNNFRIDPASGWLETNRGLDRETVPEYTLMVQAQDQGSPAKTTVVTVHVTVEDINDSPVSFTNLFSATVPENSPVNTRVVTITTTDPDTVKNVEYAITDGGGSLFAIDPVTGQIRVNGTLDREATSQYQLDVSADDGSYRKDTTVTITISDVNDNAPAFLQPSYSVSLPEGLPAGSAAAQVSAQDLDAGVNQDVFYTMKTMSDHFIINEDTGYITISTPVDYIQPVGSSDPNVYHLQVLARDLGVPSLYSEVQVTVTIYDANEYPPVFEETDYFSPVPSNTAVDTSILQVVATESSDQGQNAQIQYTIIGGNGSSRFDIDQDSGWIKVKLSLQSDIGNYYDIIVEAEDQGRPAPLSTQVNVQLLVTGSNDNTPAFDQSSYVASVVEDLPVGSEVTVVTANDGDTGVNGRLSYSIIGGNEAGLFAIGEDTGSVTVAKALDYEEFTSHTIQIKARDQGWQSKEGNTILTVNLLDVDDNPPIFNPNQYRPQVAENSPSGTYVTTVTATDADSSINAEFTYDIIGGDGEDSFTINRNTGVIRTQGELDFERGLTVFQLSVVAANEDTTMVGRAHVTVELTGVNEFYPQFGQPSYQFSVSEAAADRQVVGMVYASDADHGPDGQVQYLLIGASNRKGFAIEPHSGEIIVSWANGRLDRETEDTVLLSVLARNNEPITGDNVDEVQVTVTILDANDPPQFVDDLYQARVSEVDGVGTYVTTVTAVEYDQNNNFRQFSYAIVDGNQLSAFEIDAVSGIITVASTLDRETVATYRLQVGAIDTGVPPKTGYTEVVVDVDDVNDNGPIFLPGNDVGYISENEASNTVVMTLNATDPDLNSNPALFTFILVPNADSPSFTLESGTNVLRTTRTLDREVKSDYYLSIESRDGGMPEMTAVSTIHIIVVDQNDNPSSERDARIEVKAYQSVFPGGSLGVVRPIDLDTGDVFVCQIVSGDLNIFSLQPGCVLNSRMHSTESDYDLVISGNDGQHSSVTSNFDVSFRAFNNASLTDSIAIRLSDVVPETFLSNHFDRFSASVSSYLSTRETLLILSLNNVEDDATKMDLILAIKRDSVYLAREEVAKLITDHKSTITSQSGVSIEEVDYSPCSPNPCRNSGTCQDRIELRHTTVITDSSSIIFVTPSIKRVFRCICQGEFFGEFCEEQINQCDPDPCLNGGVCTDLIGDYRCSCPLGYTGKQCGTNIDDCADGPCLNNGVCEDLVNGYSCHCTEGYSGKNCQNGPCALQPCQNGGTCIEEGSSFRCECQYGESGDICEITSIGFQQGSYMEFPVLSPEQNIITLHFTTVLPNALLIYNHDGKSTADAEFLTLEVVAGKVQFSYNLGDGVTVITTEKMVADGQWHKVEARRIGKDGELIVDDCSMLSPAGTCRASGGTGSQIGLDLNGVPMSLGGTMSIDTILPRAWQVSSADFLGCIREVSVNGSALDLSAPLAERGTGQGCDRDTETCSSSPCSGSSVCVDEWWNHWCKCEEGFTGANCDKVPTPFSFGGGSYVEYVVKESFRRQQLLDAAKENPSGRRRRRETPPGSQTISMSFRTGSPTGLLLLVESGDDFTTLRVINSTVQYHFGSSGQTKGSVSLAKNVANWKWHTINLYREGATITLESGDQTESQRFNPIPHDFTALEVTSMSLGGTKTPVVIDGTDVTAFSGCLENFKINGAALPLDGANDMFEAVPSENTGEGCDGIDVCSPNPCLVDQICMVDGDGFRCECKAGFTGEDCKIPSTGPPESDNTLVIVLVSVFAFVVIVIIVGCLLLLRRYRNKARKAKEIATGNSKTNFSMDSLDNPGFSADTFDNPTMIGSQHNKTGNGAELISQQPDIIEQNTFSGMPEESFIVDSDDVVIGEEASGRDQEAPEHYDLENASSIAPSDIEPAYHYRYYHDGRERKQKRHGYRYSPNPMLARIGTPPCESPVSQVSSNHLNITHNPNPNSLHVRSSPSNVQHSTPMENIRASPANALARQSPGLQAMRQSPSLQLLKNESSRSATPLRLSRANTPVRNAASPIGSEHSYNQSELRSQGHRSDISSHLSGQGDQPAHVGAHRPPRPPSRLKETSPVDASRPLGLTVEEVKRLNTARPDLMSGSHASTIDNLSSVSSSKRQQQQQHQHHSNLPPLPPDLEPSPLLEPPESTSSDETAGSFTCSEVESDTEKLKSRKLDPGRLFLSGLSQMDDEEGDEPETYEQFSAPFQSKEGLDSVGGSLSTLFASEDEHVDPKKTNGQFSWDQFLNWGPRFETLEGVFVDIALLQDRAASRPPKKVYLEQTAEEFV

>PmFat4

MRPHHFARNRRKTLSMSSSVFMMYRVALLCMVFGLASGQGGYDTDFVFTVDEGQPPGFSVGTISTEQGYTYTFSDPPAEFTINRDSGEIQTAVEIDREQIPNDVFDILVLARHSVTSRTKPIEVRITVTDLNDNSPVFPETDISISFTENGRVGSSVILESATDPDKAENGSIVEYSISEGNSENAFRLMVIPGPDGLPIYINLETTVSLDHEIQDFYQLTVEARDNGSPPRSGFVHLNISVLDINDNPPIFDQSDFVIHVNESTPAGTKLLDVHATDGDSGINGEVEYFIDEETSNFNVEPVSGNITLQQKLNYISSDPYQVTVKARDKGVPTQYGRAFLSVYVIDENDHDPQISFSYIPASESFATVSEGVATGSIVALVTVTDEDLAENGEVDLQIISGNEMDHFMLDTLPLLFPTEVQTKVYKILVVGEIDRENIAEYNLTLVAWDNGSPRRRSTGNLLIHVVDTNDHSPEFQEFEYHANLSEGEPIGSFVQSVTATDADFGLNAMILYSITTGNALDWFEINPETGLVTTKALLDREVASVIELNISAADQGIQPRASETTLTVTILDENDEAPEFTSPNYTASVPENIAARFEVTTVTAMDGDEGTSGLVRYELTDDVGHQNPGLFSIDAVSGTIFTEAVLDREETPEYDLHVRAFDSGSPPRESVVSVHINVVDENDNVPVFYPQTYLVTLRENGPAVSFVEQVTATDSDSGNYGNVTYSIVGGNADGKFQINGATGEVMTVTALDREEKSSYTLLIGATDGGGRVAEISAEVRISVGDIQDSPPAFDMSVYEFEIYENANASSFVGSVSATSQDLIAQIRYIIFSGDPRSQFEIDGVTGVIRTTSALDRETTSAYELTVYANGGSMVGSATVRVQVLDVNDNAPQFPRSTDTTDVVENWEVGRHFYHVEATDEDNPPNGDVVYNIVQNPDGIFGINHTTGWMFLNTGLQASVQDEYVVQIVAADLGNPSLSATLELIVSVRDVNNHAPAFAAQHFETSISETTEVNHRFFDLVATDRDQGTNGMISYSILSGNDGDSFGVFPNGEVYVKNFLDREMISEYSLRVEARDHGIPSRTASTELLVEILDENDNRPFFGNSSYTFYLQEGSAVNTVVGSILATDADLGENAELTYTFVGNQTQFSLDRSTGVITNLVGFDREALEAETGQSRFVLDAVVRDGGATPFQDRTSITIIISDINDNPPVFSRSSYQVTISELAENGTHVLRVTASDSDSGDNSYIVYSIVNGNAEGKFQINRISGQITLIGPLDREATSEYSLTVKAQDAGSDPQHNLVEVQVQVLDENDHMPIFTHVEAVLEVTECLAIGQPITMVSATDDDIGENGLISYSITGGDEQKVFRVDPDTGDITLSKLLDHETRSSYTLNITARDSGVPQQTSLTSILVLVRDCNDNSPVFPQHTVVQRIDEELALNTHVVTVSASDPDSGINGQVRYSIVSQEPEGNHFSINPENGSILTSARIDYEYLKVHNGFFKLTVKATDQALPVESRRSATTEVVIFVRNINDNAPVFQTHNAVVISRNAPEGTTVTTVTADDPDDEDSANVTYYIVNRSVPFEINTVSGEISVNEFGFEPNVHMYSVRINAVDSQLTSNYTSLVLNILVLGDSNNGPTFARSMYSGSVSENQEQGTVVVSISAGYPGNPDAHVDYYITSVVAGGVPREGDFQMTRTGVISTGAVLDREQLGQDAMYEVTVYAISGTSAYSSTQVSHTLLLLIKYFVTPSALFGYIFIMLKRHLGAILALDEATPIGLRQIMKVEITVLDENDSPPTFDHPVYTANISEEARSSSPVTRLTAHDADIGSNAEISYSITRGNEDGHFTIDSNTGQIQTSGRLDRETTAMYTLEIRASDGRQDTVTSLEVNIQDTNDNDPVFTESMYSFSVPENHPVGTVVDAVSATDPDAGTNGDVVYSIVAEGAPGEDIFSLDSLTGEFTLLTELDYEQKQVYFLTVRANDRGTTPRSTTASVYFNILDVNDNAPVFDRTSYWVEVAEDVAVGTGIVMVTATDADSGYNGEIVFTISSGDPTNRFTIHPNGSITTIMGLDRETQSFYNLEITATDQAADPALQKSSMAQVTIIVLDINDNAPVFTTGNYATVREDTDVGEVVMFVQAVDADVEKNSYIEFSLSGVTDGVFLINPVDGNIRLLKSLDRETVPEYNITVNATDKGQPPQFTTMDIVIKVLDVNDNGPVFDLDSYHSSLPEDVPVGLEFLKVGAADADEGINAELRFSIFNGNYDSDFDIDQTTGVLSVANALDRERRSSYSLIVRAQDNGDGSHISSTTVTITVQDINDNAPVFLTPYTPRITENNQPGALVVQVSATDDDEGTNGQLTYRIKGNDYDGLFTIVPDTGVINVAALLNREEVDSYTLRIVAQDSGSPPREGATDILITVADDNDNPPQFAYPLYITTLQEEIGPNSPVLRVTANDADSTADLRYRLDTAIGNKFTINPITGQILTTSGRLDREDQASYDIIVTVEDGTTWTSQTTVRVNLLDINDNAPQFAVQSYQALIPATTSTGTIVLGVQAQDADIGDNARLTYSITNGDVTKFTIDADTGVITTAQPLTSQSSAYTLEVSARDSDPAHAPATATVQVTFDASGQFPSFQSPSNGAVYTFDEDVATDTEVVTFQASSPKTGQEGTVTYHIMGGNVDDRFDVDSQSGRLTIAQSLNYEDNQRFLLLIEARDGSTPPLTSLLQIEVNVNDVNDNYPVFDQATYSGRVTEELPAGVDVVQVHATDADSGLFGNILYTLLNGNTNNNFRIDRNSGLLETNKVLDRETIADYTLTVQAEDQGSPAKTAAVTVHVTVDDINDNPLSFTNLFSATVPENAPVDTDVITITTTDPDSVKNVQYSIISGGDGAFAIDPVTGQMRVNGTLDREVKGQYQVGVSADDGSYRKDTTVTITISDINDNAPAFIEPSYSVSLPEGLPSGSAALQVSAQDEDLGANQDVYYTMKTMSDHFIINEDTGYITVSNPVNFIHSGGSPDPNVYHLQVLARDRGIPALYSEVQVTVTIYDANEYPPVFEETQYFSPVPFSAAVATPILQVVATETIDQGQNAQVRYAIIGGNGSTRFDVDQDSGWIKVKLNLQADVGSYYDIIVEAEDQGRPSPLSVQVNVQLLVTGDNDNTPSFDQSTYLVSVYEDLSVGSEVTVITASDADAGMNGKLVYSIVSGNEAGLFAIEEETGSVTVAKALDYEDLTSHTMQIKAKDRGWQSRESSTILTVNLLDADDNPPIFNPNQYNPQVAENSPSATYVTTVTATDADTGVNAEFTYDIIGGDGKDYFTINRDTGVIRTQGNLDYEASLNVFQLSVEAANEDTSMFGIAHVTVQLTGVNEYYPHFVQPSYQFSVSEAAPDRQVVGMVYASDADHGPDGEVQYLLVGSSNHQGFAIDSFSGEITVSYANGQLDRETEDTIVLSVLAKNSEPITGDNVDEVQVTITILDANDPPQFLDNLYQASVSEVDGTGTYVTTVTAVEYDQNNDFRQFSYEIVDGNLLNAFEIDADSGSITVASALDRETVATYRLRIGAIDTGEPPQTGYTEVVVNIDDVNDNGPIFLPGNDVGYVSENEAINTIVMTLNATDPDLNSNPDLFRFFLVPNADSPSFTLESTTGILRTTRTLDREVKSDYYLSIESRDGATPEMTAVSTIHIIVQDQNDNPSSERDARIEVKAYQSVFPGGSIGVVRPIDLDTGDVFVCQIVSGDLNVFSLQPGCVLNSRMHSTESDYTLEISGDDGQHSSVNSNFDVSFRAFNNASLANSITLRISGVVSETFLSNHYDRFSASVSAFLSNRESLLILSLNNVQDDSNKMDLILAVKRDSGYLLREDVTKLINDNKATITSQSGVTIDEVDYSPCSINPCMNSGTCDDRMQLIHTTVIIDSPSIIFVTPSIHRVFHCACHGEFFGEFCQDQINQCDPDPCQNEGVCTDLIGDYSCSCPPGFTGKQCGTNIDDCADRPCMNGGDCEDLVNGYSCHCTEGYNGNNCQNGPCALQPCLNGGTCIEDGSSYRCECRYGESGDICEIMSVGFQQGSYMEFLALSPDLNIITLHFTTVSPNSLLMYNHDGTSTANAEFLTLEIVAGKMQFSYNLGDGITMITTDKMVSDGQWHKVEARRNGKNGELIVDDCPLSLPTGTCRATGATGSQTNLDVNNVALSLGGTMSIDTILPRAWQVSSADFLGCMREISINGETLDLSSPLAQRGTSEGCDRDTETCSNNPCSGNSVCVDEWWNRWCQCEAGFTGDNCDKVPTPFSFGGGSYVEYVVKESFQRQQLLDAAKANSRRRRRRDTTPGSQTISMSFRTGSLAGLLLLVESGDDFTSLQIVNRTICYHFGSSSQTRGTVSLTTNVADWKWHNLKLYREGATITLESGDETESKRFDQVPHDFTGLEVTGMSLGGTKTPLIIDGKNITAFSGCLEDFQINGAALPLDGANEMFDAVPSDNTGEGCDAVNVCTPNQCPKDQICTPDGDGFTCQCKEGFSGNDCTIPATGAPPDNTLVVVLASVFSVVLIVIIAGCLLLLCRYRNKARKAKESAVGNSKNNFSMDSLDNAGFSADTFDNPTMIGSQHNKTGNGMELIRQQPDIIEQNAFAGMPEESFIVDTDDVVIGEEASGRTHEALEHYDLENASSIAPSDIEPAYHYRYYHDGRERKQKRQGYRYSPNPMLARIGTPPCESPVSQVSSNHLSITHNPNPSSLHVRSSPSNVQHSTPMENTRASPASGIARQSPGLQAMRQSPSLQLLKRESARSATPLRLSRANTPVRNMASPVSSDHSYNQSELRSQGHRSDTSSHLSGHGDKSRISPMLAGAHRPPRPPSRLKDTSPSDSNRPLGLTVEEVKRLNTARPDLMTGSHASTIDNLSSVSSSKRRQQQHHHHNLPPLPSDLEPSPLLEPPDSTSSDETAGSFTCSEIDSDTEKLKSRSLDPGMLLLSRLSQMDDGDEDEDEPETYEQFSAPFQKKEGLDSVGGSLST

>ArFat4

MTLNNLARNRRRMWRMSSSVAIVCHLTLFLALFGFASGQTSDLVFTINEEQPPGVLVGEIPTESGFTYTFSDPPVEFELNRNNGEIRTSQIIDREQLRKDVFDLLVLATSNSRTNPIEVRIIVADLNDNSPAFPEPTDRISFSENRRIGTQVILESATDPDKASNGNIVQYSISDGNSENAFRLVVIPGPDGLPIYIHLETTFSLDYESQDFYQLTISARDDGSPSRSGVLHLNISVLDMNDNPPIFDQSEFIISINESTPAGTRLLDVHATDGDSGINGVVEYFIDEDTSKFDVEPDSGTIILQEKLDYVSSDPYQVTVKARDKGSPTLYGRAFLSVFVIDENDHDPQISFGFYSDTGNFATVTEGAAMGSIVALVTVTDGDQGENGEVSLQILNGNELQHFILETLTLLVPGFSQANIFKLLVASDVDRENVEQYNLTLVAHDNGSPRRQTTESLIIHVVDTNDHPPEFQATDYHAVLSEGEPIGSFVESLTATDADSGLNAVIVYSISAGNDLQWFEIDSETGLVTTKAQLDREVASVIELNITAIDQGVQPMSSSTTLTVTILDENDEAPVFTNPPYTTSVPENIIGRFEVISVLAVDGDQGTNGLVRYAFTADVEHQYPGIFSIDAVSGTIFTETALNHEDVSEYDLIVRAFDAGETPKESLANVHVDVGDENDNVPVFYPQTYLVTIQENEPENTFVERVTASDSDSGNYGTLTYSIISGNAAGKFQIDGSTGEVTTTSGLDREEESSYTLVISAVDGGGQAAELNSEVYISVGDIQDNPPTFDESRYEFEILESAVSNALVGQVTASSQDLNAQITYTIFSGDPNGQFEINDINGEIRTSRDLDRETTPSFELTIYASGKNLFGRTTVDVEILDVNDNSPQFLRSMDSTDVVENWEVGRHFYHAEATDADSFPNGIVVYDIFTNPDDTFGINRTTGWLFLTRDLQGSTQDNYFVQIRASDLGTPRMTALLDLIITVRDVNNHSPEFAAQRFQTSISETTEVNFRFVDLEATDRDQGTNGLITYSITTGNNGDAFGIFPDGQVYVKNFLDRESISDYILNVEARDGGIPSRTASTELLVEILDENDNRPFFDNSSYIFNLQEGSAIDTIVGTIFATDRDLGDNAELAYSFVENQTQFSLNPITGIIKNLVVFDREVLQAETGQSYFTFDAVVRDKGVVPFQDRSSITIVISDINDNAPVFSRSSYQATISELAENGTHVLRVTASDSDDGDNSYIVYSIMEGNEEKKFHIGRTSGQITLIGLLDRETTSEYMLTIKAQDAGTIPMSKMIGVTIEVLDENDHMPIFTQSQLELEVTECLAIGEPITMVSATDVDIGENGLISYSITDGDIQNAFRVNPDTGDITLSKLLDHETKSSYTLNVTARDSGFPPQTSVTSIIVHVRDCNDNSPVFPQHSVVKWIDEEIILNTLVAIVTASDPDSGINGDIRYSIESQEPDGEHFVINRENGRIVTNARIDYEYLKAHSGVFKVIVKATDQALPRESRRSATSEVVIFVRDINDNSPVFQTHNAVVLSRDATVGSQVTTVTANDPDDGDNGDITYTLINRSVPFEINSETGEVELTRNIEASALSYTLLVNAEDGGQIPGRTSSEITILIVDDTNNGPTFAVTSYTSSISENTAIGTEVVTITAGYLDNTNADIEYYITSVIGGDVPREGDFQMMTTTGVISNGVVLDRELLNADSLYQVTVYAVDRSSTTPRTASTQEVMRSVTEMMTVMITVLDENDSPPVFARDVYNASISEEAPSSSPITTLLAQDADIGSNAAISYSIISGNDGHFTIDPNTGLIRTSGQLDREIVAMYSLGIRASDGRQSTVTTLDVNIQDANDNDPEFTETTYSFTVNENHVVGTAVDSVWAVDLDTGTNGDVVYSILTEGAPGEDVFTLDSITGEFSLSTSLDYEQKQLYFLTVQANDRGTIPRSSTATVYFNVLDINDNPPVFDPTTYLVDVTEDVDIGAEIIAVQATDADSGPNAEIAFMISSGDPSSSFAIHPNGSITTVRKLDRETQSFYNLKITATDQAIDATLRKSSQVHVTVIVLDMNDNAPVFTTGNYVSVREDTTVGDIVMFVQATDADVEKNSYIEFSLSPVPGEVFLINPVDGNIRLWNALDREMQSEYTLTVLATDKGQPPQSTSMDIVIEVSDVNDNRPVFDPTSYHSTLPEDVPLGLEFLEVFATDADEGINAELRFSIISGNYDSDFDINPITGILSVANLLDHERRSSYSLTLRAQDNGDGSRFSSVTVTINVEDINDNAPVFPSHDQAEIMENNQPAAEVIQVSATDEDGGSNGQLTYRIQGNDYDGLFTIEPDTGAVHVGASLNREEVQMYTLTIMAQDAGNPPLETSTELTITVTDDNDNQPEFEFPHYTTTMQEGISPNSPVIQVTATDLDSSAALRYRLDNTIGSKFTIEPVTGEILTAGPLNREEQSSYDIIVTVEDGDLWTSQTTVTVHLDDINDNAPQFSVQGYQALIPSPTPAGTYVIAVEAHDADFGVNAKLTYSIINGDATRFSIDADTGFITTARQLTLESGSYTLEVSAADSNPSHTAAMVTVQITFDPSGEFPEFQSPSDGTIYTINEDTSANTEVVTLQAVSPKTGQQGDVTYHIVGGNAENSFIVDEQSGRLAVNTAGLDYEAIQRFSLLVEARDGDTQPLSSFIQVIVNVRDVNDNYPVFTQAGYSGNALEGQSAGIDVIQIIATDADSALFGNIRYSIRIDSNPNNDFRINPSTGWLQTNKVLDRESIPEYVLTVQAEDQGSPAKTAQVTVHITVDDINDNDLSFTNLFSATIPENSPIGTDVITITTSDLDIVNSVRYSIVGVGDGLSDFVIDSKTGRIRVNATLDREVKSQYQLPVSAGDESHVKDTTVTITISDVNDHSPTFNQASYSVSLPEGLASGSSALQVSATDLDIGANQDVYYTMKSRSDFFLINEDTGYITIKTPVDYIQPLDSSNPNVYYLEVLARDRGVPSLYTEVQVTVTIYDANEYPPVFEKAEYFSPVPFNSQIDQSILQIIATESLDQGQNAHIQYSIIGGNGSSLFDIEQDSGWIQVRLSLQSDIGSYYNLIVEAKDEGLPSPMSAQVNVQLLVTGSNDNTPIFDELTYQVSVREDLSVGSEVKRVTATDLDSGVNGMLLYTIGSGNEQGLFTINEGTGLVTVAKALDYEEFTSHTMQIAVRDQGWNDKHSITVLTVDLIDVDDNPPIFNPDQYDPKVAENSPSATYVTTVTAMDADTGTNAEFTYDIIGGNGVNLFIIDHDTGVIQTQGSLDYEGDLKIFQLSIEAANEDTSQFGIAHVKVHLTGVNEDNPRFVQASYQFSVSEAEVDGAVVGMVYASDGDQGVDGQVQYLLVGSSNQQGFSINILTGELLVSKANGELDRETQDTITLSVLAKNLGPITGDNIDEAQITITILDANDPPQFLSDLYQATVSEGDDVGTFVTVVSAVEYDQSNEFRQFSYAIMDGNHQGAFDIDALSGRITIASELDRERVSTYRLTVGAIDTGDPPQTGLTQVVINIEDVNDNGPIFLPGNDVGFVSENEAPLTNVMILNATDPDENSNPSLFTFTLLPNPDSSSFTLNSNTGLLRTTRTLDRELKSDYYLSIECGDGAVPEMKAVSTIHITVQDQNDNPSSERNARIEVKAYQSVFPGGTIGVVRPNDLDTGDVFACRIVNGDQNVFSIQSGCELNSRMHSTESEYTLEITGDDGQHPAILSDFSVSYKAFNNDSITNSITLRLSDLVPETFLSGYYDRFSASLLSFLSIRETLLVLSLNNIENEVNKMDLTLAIQKDNGYTLRDDVAKLITDNKATIMSQSGVIIDIVDYSPCSINPCSNSGTCSDHIKLEQTQAIIDSPSIIFVGPVMQRVFRCECINEFFGPTCEEQINECDSEPCLNGGLCMDEIGGYSCSCALGYTGNQCATNIDDCAGNPCLNGGTCLDQVGAYICRCKPGYSGDDCENGPCTKQPCQNDGVCIEEGSSYRCECQFGEWGDICQFKSIGFQQGSYMEFDALTGSQNIITMQFTTVSPNALLLYNHDGTSSTEAEFLTLEVISGKLQLSYNLGDGVTVISTDKEIADGQWHSVEARRIGKDGMLTVDDCSSSEADGFCRASGGNGNEMNLDVNAVPLSLGGTESIHVILERSWQVSSADFLGCIRNVYINGSPLDLSAPLAERGTGEGCDRNTETCSNNPCSGNSLCVDEWWKHWCQCQAGSSGDNCDKASTAFSFGGDSFVEYVVKESFQRQQLLDAAKADTRRRRRDAIPSSQTISMSFRTGSSHGLLLLVESGADFTVLQIVNGEAQYSFGSAGQTKGSVTLQMSLNDWKWHNINLYREGATISLEVGVDGASERFNEVPHDFTGLDVSGMSLGGTKKPLFIDGKNITAFSGCLEDFELNGAALPLDGANEMFDAIPSESTGQECGTVNVCLPNHCPVGQICNIDGDGYSCECQAGFSGDECKIPATGGPGSNTVLIALVVVFSILLLVIVLVCFLLLLRYRNKAKESRAVAATRAKNTFSMDSLDNPGYSSDTFDNPTMMGSHNKTLNNMDVIRQQPDIIEQNAHADMPGDSFIVDTDDVVIGEEASGRDLDVPEHYDLENASSIAPSDIEPAYHYRYYHDGRERKQKRHAYRYSPNPMLARIGTPPCESPVSQASSRHTPMNINPNPNSMHLRSSPSGVSHSTPMETTRASPASAVTRQSPALRQSPALLINNQPTLRSATPLRLSRANTPIRNIASPVGSDHSFNQSELRSQGHHSDVSSLRSGQGDRTHANPKPRETHRPPRPRSRLKEKSPGDLRSSLGLTVEEVKRLNTARPDLMTGSHASTIDNLSSVSSKHRHHPVLPPLPSDLEPSPLLEPPESSSSDETAGSFTCSEADSEHEKVKLRDLDPGMLLLSRLSQVDDGVEEDEEAETYEQFSAPFKKKEGLNSRGGSLSTLFASEDETMDPKKTNGQFSWDQFLNWGPRFETFMGVFADISQLQETTP

>AjFat4

MGRENCLCKLSTATMRNLWTSSPHWRPIFLVFYLLFTLASTQTDPRAAEYETFTVFEGLPAQTVIGTIATKTGFTYSLSEESPYFDIDGDSGTLRTRTVIDRDILPSDVFNLVVLSSAPTYPIEVQITVLDINDNAPVFPQPVLDIPFSESASIGTQVILDTAVDNDEKQNDVTADYAVVSSNAVGVFSLIITTNPDGDMTFLHLELISALDRETQDSYQLNISAQDGGTPIKYGYMLLNITVTDANDNEPIFETSAYDVSLNESVLVGTTVIQVRATDADIGSNAAITYSLKESSDSFDTRYFTIDPQTGVISVLEEVNYDVSSSYTLTVEASDNGHPRLVGRAYVSIRLVDQNNHAPTVNFSPPALENSITIEEGVPIEKTITVATVSDKDTGLNGLTTLNIFSGNDLHHFKLKIFRLNQDGSLYLLKINGSIDRERHPRYNITFQASDLGSPPRLSFANLIINVNDQNDHSPEFQKPFYAANLSELVPIGSFVQSVTATDEDSGANADIMYEIIGGNELDWFEIDENSGLVLTKKTIDRETTSAVTLNISASDQGIVVFTTSTLLNITILDENDEVPSFTETIYNASVPENSMSGYQLITVSASDNDEGDKGVVTYSFSQETVRRFPGVFNIDSNFGLISTATILDRETINRYELEVIAQDSGSPSLMSMAIVNVDVLDENDNNPVFYPENYFADILENKPSGTFVVQVIASDADSGESERIQYSITQDDSQGKFQIDSETGWVTTTVALDRELRSSYILTVTATDEGNREAVAPAIVQISVIDERDSPPIFSQNDYQFVIFENVPVASSIGTVHASTPDLNTDITYTIFSGDPNGVFQINEVSGQINVVRDVDREAQSFYQLTVFASGGSMIGRTVVNVTILDLNDNSPLFPRLTDSANVVENWEIGHEVYHAQAVDADSGPNAKIAYELVRNPDMTFAIDPNSGIVTLRKTLQNSESTDFFIQILAIDFGTPHQSSILNVSISIRDVNNHAPVFLSSHFGHSISEAIPVNTRFISVSATDGDEGENGEITYVISDGNIDDKFGIFPDGNVYVKSYLDREQTERYTLTIYAFDGGVPQRSSETTLTVQILDENDNRPLFENNTYVFYLEENLPPGFVLGQVRSKDRDIGLNAEITYSFVNNQTEFAIDPITGLITSKVMFDRESFYKRTRSNQIMFDIISTDSGELPLQDQTSVTVNVVDINDNTPEFSRQIYEVSISELAPNNTSVVQVSAKDADCGDNSYLTYTIVSGNEQRRFSINHVSGQIILSGNLDREFTDNYSLLVAAHDAGTPPKSATATVDISVLDGNDNIPVFVSSNVEIEVVESFPVGKELTSVTANDIDIDLNGVISYQITSGNVQNVFTIEPNTGSLFLTKSLDFETQSIYKLNITASDGGTSPNMNTITLVIYVRDYNDNPPAFPSNSVVLSVSENTEINTELVAVTAMDPDSDINGELQYSIVGQIPEGRKFGIHPFSGILYIAGEIDREDLLDYNGLFHLTVQATDQAQPESNRRMATKNVTIIVTDKNDNNPVFVSQSAAVISETASVGATVTTVSAIDKDEGINGEVTYKLNANDFFDVNPQSGVITLIRSINLSTPKYALLVTAKDNGTEQRQTHSALTVIIGDNENSGPSFTSNSYSASIAENAVNDTAVLTVSAMYADARSANIQYFITAVSTGSSSRERDFVISSTNGIIRTSAPLDHENGNDVYTMTVYAVDVDASTPRTRNTQVRITVTDINDNAPVFEKDSYRVTIPENASPGSLLTSVSATDIDSSGSISYRIINADPALFQIVSTSGELRTAAILDRETEQLYRFSVEASDGTLTSRASVEIEVNDFNDNDPIFSEPVYSFSVSEDEVINSEVAIVTATDADDGSNAELEYSIVEDGGFGEDVFQINSETGVISLTEKLDYETRAYYALTVQASDKGSPSRLSTATVYFNVLDVNDHSPIFNPDHYEQWVLENTSIGTQILQVIAEDADSGNNAIFKYTITSGNSGNLFTINDDGIIYTNKVLDRESKAFYNLAVTATDQPSDPSMARSSTAQVSILLSDINDNAPQFTNPGIISVPEDTSIGTIIMVIHATDPDATENSYISYSMSPVPGDVFRLDADGNLQVWGQLDRETRAEYTLTVIASDKGQPVQSSSMNIAVEITDSNDNPPVFLVDSPHVTLSESLPVGVEFLQVSATDADEGENSDLIFTIISGNTNDDFAIDSSRGVLFSKNPLDRERTSSYTITVQAKDSSRSPLYAITNVNIDISDVNDYRPVFLDSPYTINVQENAQSLPVIINRISATDLDANSNSQLTYSLTGNEDGDLFTIDPSTGFISIHQALDREVVEQYTLTVTARDAGSPSLTGTGTISIIVRDENDNSPVFDPDTFNITIDEEVPIGTDVLLVSATDADVGDNGNIRYQLNDEIGTMFTINPVSGQIITAGRLDREVLSTYNLVVTATDGNALSPANSRTATANVVVYLRDINDNGPVFPIVSLAATIPSTCGAGTYVTAIGGTDADEPSNSDIVYSITTGDTSKFQVGTSSGIITTKKQLTSESVSYVLEVTGSDLESAFPVAMATVTITFSSSQFPVFSAVETSYDIEESRTVGSHLVTVQAVSQNARTTYALSGGNIDEAFNIDRNGQIRIAKSLDRELTPDYELWVEARSESSPALSSFLMLDVTITDVNDNAPVFTKILYYGDVMENYAAPVPVVVVTANDADLAGNGQFEYEIDSAGNHQNAFYINPQTGEISTTKSLDREEYASYTLTVYAIDEGSPIMTGTSMVEVTIQDQNDNGIRFDSIYSANLPENSAFGSYVITVTATDHDGSTNSVITYSIKDDGDHNLFDIDPTSGRITVVGNLDYESQSIHRLTVEARDTSHLIETTVTINVLDVNDNAPVFTLVTYEKWIQEGLRVNTPVFEVTAVDVDEGDGGEVRYRMKTMSDYFKIDSVTGVISIKNEILFVTQDSTITDPNVYSVSVVAYDRGIPLFSNDTVILFHVQDSNDHPPVFEEESYFSPAPENIRIGQSIIQVVAKDELDIGINAEILYSVSGGNGSATFVVNPTSGWISPKASLEGFIGRYFQLLVKAQDQGQQDQMEATATVIILVTSINVNTPTFSGTPYQVIIPEDQLVPSQIFIASASDEDAGMNGEILYSITTGNELGLFNINPSTGAVSVIKPLDYESKNVHYLYITAVDQAWDSKQSTVVFTVQLTNINDNSPVFNPTSYTAMIAENSPSATTVVTVSATDADTPPNAGFYYSIIGGSGRDYFIVNSETGIILTQGNLDYESSNQDFELTVAATDVDPQVFRQGIAHVVIHVTGVNEYFPRFIQNSYDFFVREDAVDGFVVDRVIATDTDQGADGEVFYYLVGANTDKGFSINEITGEIFVSKAHGTLDRETENKVVLTVLAKNAGPIDGDNVDKATVTMFITDANDPPIFLEENYFGSVSEGAAVETSILTVTAVEYDELPSDRRFNYSITAGNINNAFKVDSFGAVKVNNNLDRETIDEYTLTVSATDEGNPPQSGFTTVVISLTDVNDNGPILETSAGSVLENRPIGSNVMTLIASDPDIAPQEPFSFSIYSGGSKFDLDRSSGLLTTNAVFDRESQSEYFLSIEISDSGSPQMTSISTLRIKIEDENDNPSSSRTARIEVKSYESNFPGGLIAEVKPNDVDTDDTFNCRLISSDTSMFSILRGCELHSTSHSGENQYQLTVSGNDGTHAEVQSSFSVEFQQFGSAALTSSLTLRLSNTNAEYFVMNSYNQFLTSVSSLLNSGESIIVFSLKDVSDNNQLDLLVAIEKSNGQYIPRSQASAFFQSNINTIESQSNVDIEMINFTPCQLNPCQNQGTCSDYVEMYDDEIIVETSEIIIVARNTSRVFECACAIEFHGTLCELEVDQCESVVCQNGGTCVDGSGSFSCQCLSGFSGDLCEINNDDCNGNLCTNGGTCIDLVNTHRCECPTGYFGVYCEEGPCTSQPCQNGGTCRERGGTYQCECKYGEKGNDCQFSSIGFEGGSYMEFISTLGSSNIITIEFTTVQANALLFYNHDGTTTYLSNFLALEVINGQLQLSYSDGGDIIRIKTMKVVSDGQWHTVQAKQDNQGCDLVLFDTDCPPSPDNAKMCRVSKTANLIRGLNLQGVPVSVGGVMSVRNVTDRFWQVSTADYVGCIRDIYVNSQQLGDSNVFRSAGTIDGCPRDLTTCSGQVCAGQSVCVDEWWTYRCECEEEKAGLTCDEVALPVSFGSGAYVEYTVKESYRRQQQLDNAKSNNNRKRRATNKQSLNMRFRTQADDGLLLLVNTGEEFTLLQVVDGALTYSYGTSYSTVGTITIASPKCNTGSWHNASIVRDDRHITLSLDDASKSSIFDKTPPDFSGVDVTLMSLGGTKDDLVVDGTTIAGLEGCVDSFSLNGEQLPFDGGNEAFEATVSTNTAKGCSVVDLCSSNPCLGTELCVDQETYYECRPLVAPKDSNSSLVIILVLVFIILAGIIAALLFIIIRKRKMEKKACIQNGAAVFPSSEDNLDQQPGHYNMAFDDPSLVKGQPEIMLCQPDILENESNRYKYPNISPICHNTIMDTDDVTIGIEDSIDEQIVPEHYDIENASSIAPSDVEATYYYRNYHDIEKRHRKHNYGKRSPNPLLARMQRESPVSHISRHSPNPLLRASPNMYTDITQKTSARQSPAGFKPNIRASPLHGSQSASPTKDLMQQRANSQYSLTSEQQSIYSAPVVRTSSLAVNELSLKSQSQTKLRNPNGVPNGMPVGLTVDEVKRLNTARPDLMMGSHASTMDNLSSLSSDDETGVKSSVLIDSTRLLEPPDSTSDESNDSFTCSEFESETEHTMSRSELEPGTLIFSRLAEVENETDEVDQDATIPYNYEGFNSIGGSLSTLFMSEDELPHLKQNGLFNWDEFLNWGPAFEKLVGVFKDISQLQDSSDSESCVKISYSINNTIEEEYV

>SkFat4

MVPSRLLQLVFVAFLLRLSALSTAEDDPLNRAVERANFNIDEGQPPGSFVGAISTRPGFTYRFSEESEYFSIDSISGEIRTKATIDRESLSSDLLSYVVFSSEPTYPIEVRITVDDINDNSPNFPESRIYVSFSESARTGTSVILDTATDLDSGSYGITENYAIVGGNTDDELFRLVVTTNPSGAAAFLHVVTTHTLDREAQDFYQLNISAQDGGIPPRFGFLLVDITIRDANDNPPVFDPSEYEVSLIESIEPDTLVLTVRATDRDIGENAEIIYFLDEREDLFTIHPSTGEIRTLRELDFESNEIYTVTVEAKDKGNPTQYGRAYVHVRLLDENDHSPVITFRFIPSSETVAKVAEETAVGTIIAIATVTDEDEGLNGQTSIQITRGNEQEHFRIYPLASLYAIQVAASLDRERISQYNLTFFVKDFGSPPRYTFAYLIIYVTDANDHAPEFEYEQYEVTLSEDLPSGSFVESLTATDEDADLNAQIFYRITEGNDLDWFEIDENTGLITTKSALDREIASRIVLNITAQDQGATQHLAETHIIINIADENDDAPTFVITSYNVTIMENFTPPREVVIVSATDMDSGVNGEVSYTLADHVEMDYPGVFTIIENTGKIMTLVTFDRETIPEYTLSVIATDGGVLPRSSTTQVNLKVGDLNDNSPIFYPVNYYGEIDENQPAGTTVKHGQVSATDLDFGSYGRVAYTIIQGNSDGRFSIDRNSGVIIATTSLDREQKNSYQLIINAQDDGGRSAIPYATVHITVVDILDNPPQFDKLGYTFEFFENVALGHSVGRVSASTGDQSSILSYAISSGDRDNVFQIDSVSGEITTAKEIDREVTPFYQLTVIVNGGPVVGQTLVNITISDKNDNSPEFPQPMITTAVVENWSVGHEVQQIHATDRDDGPNGQIVYELLRNPDQKFRIDETTGWIYLNLPVDSSVRTYYLQVLVTDFGVPQLSSILNVTVEVRDINDHSPVFVQTRYQTDIIESISRNTQFFQVSATDQDLGQNGLVTYSVTQGNTENKFGIFPDGNLCMVYISDVNDNAPQFHLSYYSASISETAMNNTPVVHAAATDPDAGENARITYYITDGNEGNQFHINPSTGQITLIGVLDREAVPQYILTVSAVDHGTPQLLSTTTQVVITVLDENDNAPVFAQSARSVDVAETLEVGELITIVSASDQDIDQNALINYIITGGNTYETFRIDQDSGYLYLAKPLDYETRNYYELSITARDNGRSPLTDTIKFEIHIRDTNDNAPVFTISSIIHQVTEGVEIGTDLIAVTATDPDSDINGDIVYSITTQEPPGEHFSIHPISGMISTSAEIDREDLPNGMFEIVVMAKDQAIPVSNRKSATKRVTIMVLDINDNAPVFVSQNAATIMESTLTGADVTTVTAVDPDAGSNGEVSYSLSESVNNPFSIHETTGKLTLSGYLLSSRLVYPLTVIATDHGSDRKSSSFLLNVIITSIANSGAIFTKSLYTANVYENSPIGTSMIRITASYPTNHNADIEYYITSIMSGNKRSGRDFVVDKQTGEITVGNELDREAGNTLYNIVAYAVDMTASTPQTRSTQIHVSLLDENDNPPRFDLTSYSVNILEDVNTNTVVTTVTATDADEGIDTQIAYRIVSGNDGKFYINEQTGQIRTMDELDRETTPSYTLAISASDGDLSSITTLDIYLLDANDNPPEFSKSVYAFVVSESETIGTSVGEVIAIDPDEGSNGNVTYSILEDWGQDRFSLDSYSGIFTLDKKLDFEEVQYYLIVVEASDGGTPSLSSSVSCYFSTTDVNDNVPIFDPSSYDAHVIEDVDVGYSVVKVTATDADAGPNGAISYSITAGDDGEEFQILEDGTIVTAKELDRELKEYYSLDITASDQPLEGTPQTATARVSIVIGDVNDNAPEFQNTNLTTVSEDAGAYTTVVELVATDADAGDNGDVDYSMESMSEFFLTSDDVIVVSDDGVLDRETDAQYVLTVYAWDKGVPRRTSTMQITVIIQDVNDNAPAFSSESYKVEVMENMPTGKEFFQVVATDADVGNNAIIEYHITNGNYNNDFSIDTHTGVLASENILDREGRYEYTLSVTAQDKGMVPLVSTATVTITLNDINDNIPQFESQNMEASINENNQVPALVTTCAASDPDEDSNGEMVFEILQVSAGQEGLFTIDTSGNVYVNEVLDRETIAEYHLNIQVSDNGNPSLTSSALLEIQVGDVNDNPPIFIVTNSSASVLEEQPINTTVTTVTARDADEGVNAHIKYHITNDKFTINPLTGEIRTNAVLNREENAIYVLEVIATDKVNVNSPDILSSTATVYVTVVDINDNDPAFEDLPYYAVIKEVEDYTGLTGAVADTYIYAASTNDADIGTNGLIHYAISKSNPQSKFKIGSRNGVVQVSSSLDAGDDYWVEITARDGGPGSYSIKTNLTVTFSSDSFPVFSTSPTSHFYSEDAYDPNDNVVTSVRATSSNGVVKYYMAAGNFQETFHVDRNRGEVTIEKPLDYEVMSYYQLWIEAKDDDHSSYISCTVHVTDVNDNAPVFTERVYFASVLEGEDSGTSVVTVSAIDHDSGSNGQVLYLLQSATDDFAIDSQTGELTTTKVLDRETNAFYTLTIHAVDQGTPSLTGTTTVEVDILDINDHAPSLTGWITVKSSLLSNVYQLFHLNVRASDQGNPPLSDETLVKIQVTEVNQHAPDFIGDPYSETVFENAQIGSSILTVLATDNDVGKNGDIRYSFASGNDDGLFAIGSENGVITVAEELDFEAQDTHILNVSARDLGVISLESFARVDVVLIDVNDNSPEFESQEYNPTIPENSPSGTSVVTCVATDADSTSNAVIVYKIIGGDGQYYFVINRQNGTIFSQSHLNYEDEKKSYELTVRAENEEPSMYDDTRVIVHITGMNEYYPEYIKHYYTYSVPENADDGQLVGIVAARDKDHGIDGQIRYVLVGGRTYEGFAIDGETGAIHVSYAHGTLDRESQDTVILLTLAKNGGVINGDDIDEAVVRINITDANDPPVFMPNSYVGNTRENSSIGVSVLTVSAEDYDLNEPDNLFSYEIVGGNINNAFAIDQQTGVISTSAVLDREEIQLYNLTVAAVDGGNPPQTGYAEVEISIDDVNDNGPMFYPEDTVGSVYENQSPGTSVMILSAYDPDTPVNGAPFSYYLLPSDDSEQFSVHQSTGLITTKASLNREDQSDYYLPIESRDSGVPQMTSTTRIHIMILDTNDNPSSSRSADIYVNAFQGVFPGGLIGIAHPLDPDVGDVFECEIVTADPMFSIYPGCELHSSVHSGIASYVLNISGSDTIHPSVYSNFIVDYHAFSNNSLNNSMTLVLGNIDAVTFLTSHFNLFYTSVSDELSSGETFMIIDLYDVEENMDLLVAIRLGGHQDVYYTRSDLASFFNNHEDHIEQDSGVEIIKIDYTPCENQPCINNGECTDHVEVYDNYIITDSNPIIFGSSQSQRVFVCVCKDPYFGERCEREPNLCESNPCLNGGTCHKDSLVFYWCECAPGFAGDLCDIVVDECESDPCKNGGTCHNHDNGYVCVCVAGYTGPDCDIEMDPCTPPPCFNEGECVPDDNGFTCECNFGERGDRCEFSSYSFLPMSYAQYESLGGATNWITMQFATTFENTLLLYNHDTNVGDKSEFIAIEVIDGKVWFSYNMGDGTTRVHTDKIVSDGEWHEIIATRERLRGVLKIDGCTDESLPPDYCFSSSQGVGAYPHLDLQGVPLNIGGIKTIDSITERPGQVSTYDFVGCMRDVYVDGIFLDLAEPLDSSDVSHQCPREEDMCESNPCQNGGTCIDEWWKYRCSCKDGFMGHHCEEEMSSFTFGDGSYIKYTIKNSFRRQKLLEGELKRKKRSTDPQTASLSFRTRHEDGLLFYMVDTEVDRYTILEVVKNKLQYIFSSGSLGTGSISLEIGVSDGEWHNATLTKIGFEVTLTLDGIDKTTTFDIPPHDFVSVEVQNIFLGGTEDPLNHDERNILGFEGCIDSFVMNDNLIPFIGESEIVVAEPSEGSTDSAIGCEGTDVCASNPCPAEHFCLDEWESYTCIPEGACKSDPCLNNGTCIPQDAGYLCICPKNYTGPQCDTALVCALDPCGEDQECIVNENGAYFCKNIAEEGSSQLEIIIVIVIIIFVLILAVIVFVIIYCHCRKKKSKQKQPPVVVNVDGIGNDAFTTDNDQKSSPTNSELARLGISGTQPDILALEQNKNVLVSQVDEDTTIMEDQSSIIPTNDEFYDLENASSIAPSDIDVTHHYRDYHRDGNRKLRNNHAHRMSPNHLHSRGVRPNLYAMNQLRHSPGLPSQYYGQMHRQSPHPRISPHHMMQSRASPIKELIHSRSNSEQSINQSELRSEPGFRSNASTVSENSRRTKSPISNGHLPNSRPQSRLKSPLTVVVDAEQPKGLSAEEVAMLNSARPNEHAGSLPSTLDGISSGSSDRNVQSVPFARNPQNLLEAPETSTDESNDSFTCSEYECGPEKLKFPEFDPSNIIFKRLAVVKEDDPNIPDTSRTYNYEGIDSAGGSLSTLQMSEDDIPHVSMKPPNGHFSWDYLLNWGPSFENLVGVFHDIALLEDGGIALKLDRDPSLEEYV

>BfFat4

MASPRVRTRWKHYYFTLLFILRALICSGQEGADRAASSARVAFSLEEGQPPGTFVGNIPTQPGFTYRFNQDPGIFSLDPSSGEIRTTQEIDREALSDDSLDLVVLSSLPTYPIEVRIDILDINDNSPIFPDPSIQISFSESARTGSQVILDTATDADIGNSDITDNYRIVSGNANNTFELVVTTNPSGEASFLHLQTNQNLDREIRSSYQLNISAQDGGSPPRYGYLLVNISVRDSNDNPPIFDQSEYIVSLNESAPIGTSVLTVRATDIDEGTNAEITYLLDSGEPATKFQIDPRTGVVTTLQELDYEESDNYYVNVQAHDNGSPIQYGRAYITINLVDENDHDPVIGFRYFPIGAQFASVEEDAALNTVVTLVTITDADQGPNGDVDLEILSGNEQGTFQVTHVPGLSIVKVARSLDREITDQYNLTMRATDHGNPPRRTIANLIIYVNDINDHEPVFERTRYNTTLSERVPIGSYVQGVTATDDDSGLNAQVRYAIVSGNEYGWFMIDGNTGLVTTQADLDHEVASGVVLNISASDQGISIQFTSYTQLTITIIDENDFVPTFSESPYNVTVLENQSAPREVITVTAQDDDQGSNGEVYYEFDTETANRYSGSFRLDSNTGRIDTLVTFDRETTDLYSLVVRASDRGNPPQQSAVRVNVIVGDLNDNNPVFYPVRYYASIRENQPSGTSVVQVSASDLDAGLNGRVTYQITRGNPDGKFTISPQSGQITTTAELDREVRASYQLEVTARDGTGQSSASPATIDVTVVDIQDNPPEFSQPGYNFVIFENVEIGKSVGMVSATFRDANANATYSIFSGDPAGIFRINSQTGLISTAKEVDREQQQLYQLFVVASGGRVVGQTQVNVTLRDLNDNPPSFLTSSAQADAVENWPLGREVYEAAAQDPDDGVNAQVTYELITNPSNKFQVNGDSGIVSLAGALSYEVGQYVLELQATDRGLPPQSATLTLTVTVRDVNDHAPVFSSSSYSTAIPESQPVNDQFFSVTATDEDAGLNGLISYSITGGNPGDRFGVFPDGNLYVRNALDREVQSRYVLIVEAQDNGVEAKSAVANVTVEILDENDNRPLFANSTYNLFLDEEAPAQTMVALVTAVDRDAGTNAELRYSFITEQSDFAINPSTGAISSLRMFDREELIQRTGLSTFTLEVAVTDGGDPPLQDRATLQITVRDINDNVPRFAQDTYQVSISENAQRSQQVVRVSASDMDYGNNALVRYTITEGNDEGKFLIDSSSGQIMLAQLLDREVEDFYSLLVDVRDSASENPLSSQCRVNISVLDENDNEPVFSPTLQTADVLESTRVNDLITTLTATDGDAGSNGEITYSISEGNAHGTFTLDSYTGKLYLAKALDYESTNVYRLNITASDHGTPPLSTVIPFAINVLDSNDNPPTFPPGDTVRQITEGVGLNTVIATVTATDPDSGSNGEIQYSIASQVPQGDQFAVNPSSGQVFTRAEIDREFASTFDITVRATDQAIPVSERRHAFKVITVIIRDVNDNNPLFVSQDAAAIAPSASRGDVILNVVAEDPDEGTNGEVRYAETSGNTNQFDIGGTTGVVTLAQNLSPSQTFYTLVVSATDQGPGRRSSNSEITFFITSPNNNGPAFSRSSYSGIVRENQPVGTSVTSVRASYSDGHSATIEYYITSIRAGDASVGRYFTIGRTTGRITTGVELDREEGYDSFVLEVYVVDKSSSSPRTRSTQVTITLEDENDNAPMFNTDYRDISIPEDLASGGLVTTVVANDADVGSNAVVLYSIIAGDDGQFRVDSDTGEIRTTGPLNREQSPTHSVTVQASDGQQASTIDVNISLTDVNDNAPQFLRSVYSFDIREDAAVNSEVNFVSATDTDEGSNADITYSITDENQGMFDITPKTGVIILRRPLDYEQTQHYVLTVQARDGGGRASSVTVYVNVKDLNDNPPVFDPDRYSADDISEDVPAGTSILTVSATDRDEGPNAEMDFSIVSGDPNDQFSIQSDGTIVTAAALDREQNYLYNLVVMATDRAIPAYMRLSSTAQVEIIVNDINDNPPRFVTPNVTSVYENSPVNTVVMTIRAEDPDEERNSYVEYSMASIPGNKFALDSVTGQLRVNGDLDREATPSYIVTVTATDKGRPPMSASMDLTINLLDRNDNNPVFGPVSYDVSVLEDVAVGTELVQVVAADVDEGTNGVVRYSIVSGNTDNDLAINAVTGRISVARLIDHERTTGYNLVVRARDQGTSPLEALATVTITVTDVNDYVPLFLNSPFVAHVMENDNNVPQFVTQISAQDEDSGTMGQVDYYLPDDKDGKFSITSSDGRISLLRTLDREDQSEFLLTVLAEDRGSPRLTGTGTVRVLVDDVNDNTPTFDQQSYTTTIAEDVPVNTDVLLVSATDLDAGENGNIRYTLTGDLGDKFEVNPATGQIITIGLLDRETQSLYTMVVTATGSSAADARSSSATVIVTVSDVDDNVPAFVDRSIDAYLPDNTPTGTFVALASATDPDEGTNGDVRYSLSGSDANRFTIDRVTGVISTAMTLSDSSSQYNLQITASDLGPNAGTDTASVTINFQPAASFPSISVGTDTFNYPETQSVGTVLTSVQGSSSKPSPANTITYHIAGGNEGDVFEVSPPGQVRLRRSLDYEETSSFNLWMEARDSDTPALSSYHRLTIALADVNDNPPIFSQDPYVGSVGENEGTLTSVLTVSASDADSGSNGEFEYRLRAAGNLDNSFQVNPNTGQVSTRRFLDREQRDSYSLVVEAVDKGSPTLTGTATVLITVEDKNDNPMQFRNLYSTSILEDTPIGSFVIQVSTTDPDIGSNAMAQYKFSLDSQYAEARGKFNMGRDSGNVTVASTLDREISDHYELLVIAEDSAWEANTPLSITILDVNDNPPRFSAPVFERNLPEGIRANQLVTRLVATDEDEGTNAEVFYRMKTLSNFFRLDESSGAILTKNSLEFVPDWTGTTNPNHHQFVVIATDRGTPPLSGEVTVIINIIDANDHAPVFQEVSYFSPVPENAQIGEMIIQVLAVDNQDIGSNAEVEYSITGGNGTNPNLFSIDRTTGWVSVSASLSGRQNIWYSVTVQARDQGSPALSTTTDVSILVTDVNVYAPAFSAPSYQVTIEESRPMNSHVFTVVATDRDSGVNGEIRYSIVGGDDYRQFYIDPRTGSVTVAAVLDYETTPTYHLNISARDRGLLYRENFVILTVDLTDVNDNDPMFDPATYDPSIPENSPSGTSVVMVTATDADTGANAAIRYQITGGDGQDLFIIDTVTGEITSQGGLDYERKQMYTLQVTATNVEASSRFGTCTVNIHLTGQNEYAPVFLQRLYTFEISESAKPGTTVGTVYATDRDDGSDGIVNYILVGSSNDKGFAIGLESGAITVAQRLDRETSSHIMLSVIAKNRGSILGNDIDEIQVNITIIDANDPPVFEFSLYEGHVSEGDNIGTSVLTVSAIDNDLRADFRSFVYGIQDGNEGSAFSINQISGIISTASRLDRETIDTYNLTVTATDTGIPPATGTTYVVVNIDDINDNGPEFVPPNVTGYVLENRAPTTVMTLSATDPDLDPNRGPFSYRLIGGHNSQYFTLDQGSGRLSTTRQIDREQQSDFYLIVETTDSGSPSMSSTHTVHIVVEDVNDNPSTPRTVDIYIHSQDGSFPGGPIGNVHPIDPDIGDTFSCTITQGNTQVFSIPSLCNLNTGRINTVTDYTLRVSGDDGVHASVVSTVHVHFREFTSDAVTNGIIVRLNNVTTSNFLKNSYTNFISALNDISGVYSTVLYSMENTGEDLDIVLALQQTNQQFMVPQQAATAITNRKTNIENTASVSIVAVNFNPCSDSPCQHGGVCSKTLQVTSSRNILESDPVIFVSVDTGNRYTCSCPTGYTGDSCEIEINECDATPCLNGGTCTDEIGTFTCECPPGYHGDRCQNDFDECSSNPCRNGGQCLQGLNDYTCSCAQGFTGKNCEIDVDYCISQPCLNNGTCTDGQTSYSCRCGFGEKGDNCEITSYGFEEVSYMQFPVLDQRNNDIMIEFATVMTNALLLYNYDAEESDSSDFIALEIMEGKLRLSYQLGDGITRISVEKNVADGQWHTVTARRNGKDGTLIVDNCGSSSPAGFCRNTGGTGTASGLDLSGLPMMLGGVQTIDAILVRPGQVSSNDFVGCIREVTINGVPINLAGPLSSRGISDRCPRVVSDVCSPDPCKNGAACQDKWSSHQCQCSDIYVGDNCEKRRVPFSFGGSSFVEFQIKESYVRQLQLGNQVSSRRRRANQDTSLSLKFRTRENSGLLLYAGGSSRYTVLEVKAGKLVYSYNAGSGHRTREIDVAVTNGLWHTVSLVRRSTSTSLFLQNEEQTQDWNTEITGTTHDFLSSDVATMSLGGTNTPVTIEGQTLPGFDGCIDELRLNGQLLPFVGSNDIVIATPSENVGEGCPSPAVCASNPCPNNLLCIDQWQQYECAEPGACGSSPCQNNGTCIPNDSGFRCRCDGNYNGTLCENALACIGFTCGTNQVCVGVGATGRACQCVDGWSGDNCEIKLPQTSEPGLHIGVIIVIVFFCLVAVIILIAFIVYHRRRMLRKKDQAPKTAKQNGGHMANGGQENRAYNTDDIDSVTPYLGDGMMSSDTMYDQKAMHFNEREVKMRDPTPDIIERGRGSVPIDNMDDDDVVIENDDGLTAMSRLPEDRDPFPEHYDLENASSIAPSDIDVTYQAYYKAFREGKRYKQPVPNRHNHHPRQSPSSLLHQVHMRDSPNHLARKSPNHLSHRDSPSFQSSARQSPASHLLRQSPNHLARQSPASHLEGPMRPLGMRTSSDHLPPSEHGSHYSGSSIGSKNRRRAKSPCASSHGRGSRPSSRLKQPIEQIEMDSGPPVGLSVEEVEMLNARPRHSVASTMDGASSFTERTMPVNDKIMSLLEPPDLLEPPESSTEESQDDSFTCSEFEYEREKPLRSRNDLDPRTNKMFPHGDNGSFGGSLSTLLEGDDPQKKHALPPNGTFSWDYLLNWGPNFENLVGVFSDIAALPP

>MmFat4

MNLAANRAPGRRRLPLPSPSLCQLLRVWGLLSLLPGSARVQAAEQRQVFQVMEEQPPGTLVGTIPTRPGFTYRLSESHALFAINSSTGALYTTATIDRESLPSDVVNLVVLSSSPTYPTEVRVLVRDLNDNAPVFPDPSIVVTFKEDSGSGRQVILDTATDSDIGSNGVDHHSYRIVSGNEAGRFRLDITLNPSGEGAFLHLVSKGGLDREVTPQYQLLVEVEDKGEPKRRGYLQVNVTVQDINDNPPVFGSSHYQAGVPEDAVVGSSVLQVAAADADEGTNADIRYRLQDEGTPFQMDPETGLITVREPLDFEARRQYSLTVQATDRGVPSLTGRAEAFIQLLDVNDNDPVVKFRYFPATSRYASVDENAQVGTVVALLTVTDADSPAANGNISVQILGGNEQRHFEVQRSKVPNLSLIKVASALDRERIPSYNLTVSVSDNSGAPPTAEVQARSSVASLVIFVNDINDHPPVFEQQVYRVNLSEEVPPGSYVSGVSATDGDSGLNANLRYSIVSGNGLGWFHISEHSGLVTTSAAGGLDRELASQIVLNISARDQGVHPKVSYAQLVVTVLDVNDEKPVFSQPEGYEVSVVENAPTGTELLVLGATDRDLGDNGTVRFSLQEAENDQRLFRLDPVSGRLSTASSLDREEQAFYCLSILATDLGSPPQSSTAQVNVSLLDINDNSPVFYPVQYFAHIQENEPGGSYVTTVSATDPDMGPNGTVKYSISAGDRSRFQIHAKSGVISTKMALDREEKTAYQLQVVATDGGNLQSPNQAIVTVTVLDTQDNPPVFSQAAYSFVVFENVALGYHVGSVSATTMDLNANISYLITTGDQRGMFAMNPVTGQLTTASVIDREEQSFYQLKIVASGGAVTGDTVVNITVKDLNDNAPHFLQAVESINAVENWQAGHSIFQAKAVDPDEGVNGRVLYSLKQNPKNLFTINEQNGNISLLGALDVHAGSYQVEIVASDMGVPQLSSSILLTVYVHDVNDNPPVFDQISYEVTLSESEPVNSRFFKVQASDKDSGANGEIAYTITDGNNGDAFGIFPDGQLYIKSELDRELQDRYVLLVVASDRAVEPLSATVNVTVLLEDVNDNRPLFNSTNYTFYFEEEQRAGSFVGKVSAVDKDFGPNGEVRYAFEVTQPNFELHAVTGEITSTHKFDRESLMRRRGTAVFSFTVTAMDRGLPQPLKDQATVHVYMKDINDNAPKFLKDFYQATVSETATNLTQVLRVSASDVDEGSNGLIHYSILKGNEERQFAIDSFSGQVTLVGKLDYEATSAYSLLIQAVDSGAIPLNSTCTLSIDILDENDNTPSFPKSTLFVDVLENMRIGELVSSVTATDSDSGVNADLHYTITGSNNHGTFSISPNTGSIFLAKKLDFETQSLYKLNITAKDQGRPPRSSTMSVVIQVRDFNDNPPSFPPGDIFKSIVENIPLGTSVISVTAHDPDADINGQLSYAIIQQMPRGNHFSIDEVKGTIYTSAEIDREFANLFELTVKANDQAVPIETRRYALKNVTILVTDLNDNVPMFISQNALAADPSAMIGSVLTTIMAADPDEGANGEVEYEILNGDTDTFTVDRYSGDLRVASALVPSQLIYNLIVSATDLGPERRKSTTELTVILQGLDGPVFTQTKYITILKEGEPIGTNVISIEAASPRGSEAPVEYYIVSVRCEEKTVGRLFTIGRQTGVIQTAAILDREQGACLYLVDVYAIEKSSAFPRTQRAEVEITLQDINDNPPVFPTDTLDLTVEENIGDGSKIMQLTAMDADEGANALVTYALISGADDSFRIDPESGDLIATKRLDREHRSKYSLLVRADDGLQSSDMRINITISDVNDHTPRFSRPVYSFDIPEDTTPGSLVAAILATDDDSGVNGEISYVVEEDDGDGVFFLNLVTGVFNLTRALDYETQQYYILTVRAEDGGGQSTTIRAYFNILDVNDNPPVFSMSSYSTSLMENLPLGSTVLVFNVTDADDGVNSQLSYSIASGDSLGQFAVDKHGVLKTLKALDRESQSFYNLVIQVHDLPQPPTSRFTSTAQVSIILLDVNDNPPMFLSPKLTYIPENTPIDTVVFKAQATDPDSGPNSYIEYTLLNPSGNKFSIGTIDGEVHLTGELDREEVSNYSLTVVATDKGQPPLSSSTEVVVMVLDINDNNPVFAQAMYRVQIKENILTGTDIIQVSAADNDEGTNGQVRYGIVGGNTHQEFRIDSVTGAITVAKSLDRETTPAYTLTVQATDRGSSPRTDSCTVAITLLDMNDFVPVFELSPYSVNVPENLGTLPRAILQVVARDDDQGPNSQLSYVLLGGNEDNAFVLTASGELRVTQSLDREARDHFVLVVTAADAGSPALTGTGTINIIVDDINDNVPTFANNMYLTSIAEDARTGTDVLLVNASDADAAANAVISYSIIGGNSQFTINPSTGQIITSALLDRETKDNYTLVVVASDAGSPESLSSSTSVLVTITDVNDNPPRFQHHPYVTHIPSPTPPGSFVFAVTVTDADIGSNSELHYSLSGRNSEKFHIDPLRGAIMAAGPLSGASEVTFSVHVKDGGSFPKTDSTTVTVRFANKADFPKVRAKEQTFMFPENQPVGTLVTTITGSSLRGETLSYYIASGNLGDTFQIDPLTGQVSISQPLDFEKIQKYVVWIEARDGGFPPFSSYEKLDITVLDINDNAPTFEEDPFVSEILENLSPRKILTVSATDKDSGPNGQLDYEIVNGNQESSFTINHATGEIRSIRPLDREKISHYELTVKSSDKGSPSQSTSVKVIISILDENDNAPRFSQIFSAYVSENSPLGYTVTRVTTSDEDIGINAISRYSIVDTSLPFTINPNTGDIVISRPLNREDTDRYRIRVSAHDSGWTVSTDVTIFVTDINDNTPRFSRPSYYLDCPELPELGSRVTQVSATDPDEGSNGQVFYFIKSQSEYFRINATTGEIFNKQVLKYQNVSGFSNVNINRHSFIVTASDRGNPSLLSETTVTINTVDSNDNPPQFLQNKYFTPVTKNVKVGTKLIKVTAVDDKDFGLNSEVEYFVSDGNHLGKFKLDNDTGWISIASSLVSDLNQNFLIRVTAKDKGNPPLSSQAVVHITVTEENYHTPEFSQNHISATIPESHSIGSVVRTVSARDRDTAMNGLISYNIISGNEEGIFAINSSTGVVTLAKALDYEMSSKHEMTISATDGGWVARTGYCSLTVSVIDVNDNSPVFVPDEFFPTVMENAPSGTTVIHLNATDADSGANAVIAYTVQSSDSDLFVIDPNMGVITTQGFLDFETKQSYHLTVKAFNVPDEEKCSFATVDIQLKGTNEYVPRFVSKLYYFEVSEAASRGTAVGEVFASDRDMGADGEVHYLIFGNSRKKGFQINKMTGQIYVSGLLDREKEERVSLKVLAKNFGNIRGADIDEVTVNITVLDANDPPVFSLSTYRVQISEGVPIGTHVTFVSAFDSDSIPSWSRFSYFIGSGNENGAFSINPQTGQITVTSGLDRESLPVYNLTVLAVDSGTPSATGSASLVVTLEDINDNGPVLTVSEGEVLENKRPGTLVMTLQSTDPDLPPNQGPFNYYLLSTGPATNYFSLSTAGVLSTTREIDREQIADFYLSVVTRDSGAPQMSSTGTVHITVLDQNDNPSQSRTVEIFVNYYGNLFPGGTLGSVKPQDPDVLDSFHCSLTSGVTSLFSIPAGSCDLSSQPRSTDGTFDLTVVSSDGVHSTVTNNIRVFFAGFSNATIDNSILLRVGVPTVKDFLTNHYLHFLRIASSQLTGLGTAVQLYAAYEENNRTFLLAAVKRNNNQYVNPSGVATFFESIKEILLRQSGVKVESVDHDPCIHGPCQNGGSCLRRLAVGSALKIQESLPVIIVANEPLQPSQCKCVPGYAGSWCEVDIDECLPAPCHNGGTCHNLVGGFSCSCPEGFTGRACERDINECLPSPCKHGAVCQNFPGGFNCVCKTGYTGKMCESSVNYCECNPCFNGGSCQSGVESYYCHCPFGVFGKHCELNSYGFEELSYMEFPSLDPNNNYIYVKFATIKSHALLLYNYDNQTGERAEFLALEIAEERLRFSYNLGSGTYKLTTMKKVSDGQFHTVIARRAGMAASLTVDSCSENQEPGYCTVSNVAVSDDWTLDVQPNRVTVGGIRSLEPILQRRGHVESHDFVGCVMEFAVNGRPLEPSQALAAQGILDQCPRLEGTCARNPCQHGGTCVDFWSWQQCQCMEGLTGKYCEKSVTPDTALSLEGKGRLDYHMSQSEKREYLLTQSIRDTTLEPFGVNSLEVKFRTRSENGILIHIQESSNYTTVKIKNGKVHFTSDAGVAGKVERIIPEAYIADGHWHTFRISKNGSITVLSVDRIHNRDIVHPTQDFGGIEVLSMSLGGIPPNQAHRDTQTGFNGCIASVLYGGESLPFSGKHSLASISKTDPSVKIGCRGPNICASNPCWGDLLCINQWYAYKCVPPGDCASHPCQNGGSCEPGLLSGYTCSCPESHTGRTCETVVACLGVLCPQGKVCKAGSPGGHVCVQSQGPDEISLPLWAVPAIVGSCATALALLVLSLILCNQCRGKMPKNPKEEKKPKEKKKKGSENVAFDDPDNIPPYGDDLAVRKQPEGNPKPDIIERENPYLIFDETDIPHNSETIPSAPLASPEQEIEHYDIDNASSIAPSDADIIQHYKQFRSHTPKFSIQRHSPLGFARQSPMPLGASSLTYQPSSYGQGLRTSSLSHSACPTPNPLSRHSPAPFSKPSAFYRNSPARELHLPLRDGGTLEMHGDPCQPGMFNYATRLGRRSKSPQAMASHGSRPGSRLKQPIAQIPLESSPPVGLSIEEVERLNTPRPRNPSICSADHGRSSSEEDCRRPLSRTRNPADGIPAPESSSDSDSHDSFTCSEMEYDREKPVVYTSRMPKLSQVNESDADDEDNYGARLKPRRYHGRRAEGGPVGTPAAASGAADSTLKLGQQAGNFNWDNLLNWGPGFGHYVDVFKDLASLPEKAAGNEEGKSGAAKPAAKDGEAEQYV

>CgFat4

MEISGFSECAKCGRAPSRKRWTGIVLVLLQFLVTYAQGQGPGTNFSVSEGRPPGTLVGNLIIQPSYSYYFNEPQSYFSLNPTSGQISTKTEIDRELLKSDRLNILVISRPPGNTSQIPIDIYITVLDINDNSPTFPESPVQRDISEKANIGYKIRLDTATDNDIGKNGNVTNYQIIKRNDDGRKFRTVFDPSKFGQVLYLELIDILDREEKDSYELTIRAADQGEYPLSGELVVQIQVSDANDNPPIFSLSQYTSTVNESSPVDTLVIRVDATDDDIGENGNIFYTMTDESKQFKIDERTGEIRTVSTPLHCYCDGSQVCLPGECLFLTLEARDGGNPSLNGRAYVKVSIKDENDHAPTINVMNQGPNDYISVNETAKNGDYVVAITVSDADSGMSANISSVQIVAGNELNHFRLNSFFQFKYNALLVNVNGLDRERIDQYNLTIQAVDMGSPPKTGTKSLIIIVADVNEYPPTFLQRQYVANVLETLPVGSFIANLVAEDKDSGINAKLTYKILSGNNNDWFEINSETGLVTLKKQLKYNLAPEVVMNISVEDGASVPLFNYTKLTVRISDENDVTPTFSQSSYQVHLLEGLQLGTEVISLTAADSDSGLNGSVIYSLHPEVQEMYPDTFNVNIQSGRVITLKQLDRETLSQYVLKVIARDQSVLPLSSTATIYLTVDDENDNVPVFYPQKYYTNVIRSATSGTKVVKVNAVDPDLGDGGRIYYSFPNDYPQFVLDTNTGWISTTYEFERSSQQEYSLQVTCRDTNTVHRAAVNAVVKITVVTSLNSLPVFTKSSYQFTVMENLAGNQFLGSVAVSRGESPSFAITDGDIEGSFNIEGQRGEIRTTKSLDREHISRYDLTVTATTNDGTSEVKVIVTVLDANDNKPEFKYEFLDVELQENSAVGHEVYYAFAVDDDSGQNGLLSYALTTNTNFVQINQNTGMITMSKPYQRVYGNKFNLTVTASDSSPSPNSASMVVQVHIVDVNDHNPVFPKTDYAITLEESTLVNTQFHTLTATDADVGRNAALVYNITKGNDDGKFGIFPDGKLYIAKQLDRETRDLYKLTVMAQDHGAPARSSEVNVTIHILDSNDNRPRFLNQTYSFYVSENVPPGQKIGAVKAVDADIGRNAELAFLLPEDQDDFAIDFQTGEITTLRSYDREALVSETGNDYYSVVVTVVDNGVQRQQDSVTARIYVLDQNDNPPHFDRDIYRTSVKENLAKYSFVYKVTATDLDVDENAALTYDIIDGNTDDMFNINPGTGQISLNGNLDTETQDLYSLQIQAADNGKSTTFTASAMVIISVLDVNDNTPLFHLTVYEVNIREDAKLGEKLIQVTADDTDLGINAEIEYSLSESDGTFVLDSHNGNLYLAKLVDYETRKVYSLKVIATDKGDPRLSSEADLNVRIIDVNDNSPEFTNDFSVLSISESTERFSSIGQILANDKDSGLYGQVEYHIVSQDPPGKNFYIDKNTGRLRLETLVDREEVASYMLTIVATDLAPNASLRLTSEKTFSIQILDENDNSPILRSAPAIQIQYPTSKNHIGTILASDPDSGSNGTIVFQLLSSESNAYFNLDSRLGKLYLTENLPQSPTMYKLNVQISDEGRPSQTTTVTITVILMQSLDTRTGPQFSNTPYLFSVRENVQANVNSVLSSEASVEYYIVNIKSSFGQPGYYFTLDKTSGMIRTNGKLDREVTGSQVNLTVCAVNTQSNSPQATLEEVVISIIDDNDTPPQFSQTSFVKNVGEDLPVGSEILRITVTDHDTTQGTQTLIIANGNYGNYALNSQTGALTIARRLDREVRSTDRLIIESSDGTNTAVATVKVNITDINDNAPVFTNVFYSFDVPEDTPIGTTIALVEAVDADQGLNGEVVYTLVSTWGQSIFHLDPQMGTIRLIKNVDYEQNHLFSLTILASDKGSPSQSTSVLVYLNVKDVNDNEPQFNHQSYAGQVLENAAVGTVVLTVQATDIDSGINGQLRYSLLEGNSSSDFGIGTDNGTIYTLQNLDRETRSVYSLVVIATDQAEPSSSQKSTTTEVIITILDVNDNSPEFVTPSQIWVKENSSVGSIVYTVSAVDIDEGVNAKVMYSLASHPVFSISPSTGQISLSSQLNRELIQNYTLQVTATDQGLESRFSTQKLVIMVEDVNDNPPVFVPSVYSKTVREDVKIGTTLLQVTATDSDSGLNGVVRFFITSGDDNADFSMDPSSGVLRVQKNLDYERVNKYTLTIQAEDMGVSPRYSVASVTITIQDVNDFQPVFQNSPFYALVRENMASSPPVPVITLSAVDLDSAPNSHLTYVLREGDKGVTSLFQINSVSGEITCNQTLDREQVPQYKVLVVAIDSGSERLTGTGTIYIEVEDVNDNAPMFDRSSSYVGHVQENQSGPTDILTVTATDNDSGPNAQISYSLLDDVDGRFAINTTSGILTSQKLLDREDTALYHLTVVAKDHGNLAKSAEANITVYVDDVNDNVPQFEKQTYNKTLNNPTSAGQFVVGVTAVDRDVGPNGRVTYRLQGGDCSRFNLDNQRGIITSATFMSGAGTRFQCTIEARDQGSQEKMSTTTLVVEISSVGPSSIPVFDTVNSPVSLNEGVPLEHVVNTVTARPATNNGVIKYSIAGGNVGSTFRIEESSGTIRVAGEVNYEMTPDFHLWVQATEGDNLLLSAYKEVVINIVDENDNTPRFQEGVYITSIRENVPVSSKVYTVNATDADSGDNGKVVYVLAGGNVDNIQGTFNVDPVTGVIQTKVALDRETQDFYNLIIEAHDMGLIRRTGTTTVRVTLIDVNDNSPTFSTIFSVTIPEDLPVNSFIIQVTSTDKDIGINAKAHYSIDTTANYSNLFNIDADSGNITLKSSIDREALRTQRITIPLIVDDGSFSTRGYLHLFVTDVNDNAPQIQPPVTFNFLELQPVGSTVGKLTALDNDVTSPNNVSYFSFKLPSSEFAIDDTTGVITSKETLTYIYHKSYSNALNQRELVVIATDLGTPAKSSEAIITIEIIDANDHAPVFDQDLYFSAVPESAPQGERILTVLAQDKLDVGQNAEVEYFIESGNGSSYFFINKTTGLITVAQVLSTKRNQDFTITIRAEDKGMPPKTATVPVHLSITAENSYTPVFQPASRVYNVQEDAPIGRSVARVTATDQDSPGPNGKVSYYIQGGNPGGVFQINSENGVISVAKALDYDSPTNIYLLNVSARDSALHYKEATVILTVQLTDVNDNPPLFTQSHFEGFVPENSPSDTSIIKVQARDIDTGDNARIEYYISESGSDQLALTLFKINKDTGTLMTKGILDYENQIKYSMVVIARNPNNVNMRNTVKVTVHVTSVNEYYPEFVQKTYAFSTKESAANGSVIGKVSATDRDKGIDGVVYYYLIGSSNVKGFSVNYKTGDIFVSGKPDYESSPHVVLNVLAKNWESVKGNDTDTCTVTISVEDANDAPVFTQSLYQASILENSAGGVTVTTVTANDRDNMPEDRQFSYRILRGGEEFSIDSTNGRIHTTGRGKLDRETNATHSILVGAVDRGTPPATGSATVRITLLDENDNSPYFTPADLTGYIKENMPVGTRVMDLVTNTADHDLDLVGEPGNQGPFRYQISTGSDNFEISAEGLVVTKQVLDREAPQGSEHDVTVIVRDAGTPTQSATLTFKVVVEDVNDSPPQARDLVIQVGVFETTLPTAPVADVRPLDEDITGTYTCTVTDEYYTITRGAGCQLTLLSFHTPPSRTLNVQGSDGNIMVSYNVRSLLVYFDNVTLDNVVIIYIDGITKAEFVKEKFDNFQKAVSKLFSIEDSVTILNVDSKDAGTLVYVSVVKASGTVVLHDFLKQQLNSNKQAVASAIFPNVVNPENRIQIGYSVCEVSTCNSGTCLNRINVRSGLHTLDSPLFVLTSPVMVPDFYCSCPSQYTGRYCEEPVQPCGGGFCSNGGYCQNGVCQCLEGWLGQNCTQDKNECTSSPCQNGGTCNNLPGSYTCQCPDGYTGQHCETGSNHCASSPCQNGGQCKNELDGFHCQCPYEFWGNRCQHVSKGFSEQSYMEFNSIDQNLKDIDLDLTFSTIKSKGLLLYNPSNTGKFLALEIFNRKVRFSFNFGDATATVLTVPKNVSTGEWFRVQVQRRLAVAELKVTHCPATSTECNTCQIGDDSCYRKGSQSNNYLDLNGHPMFLGGLKDIEIIQTRPGQISSHDFVGCVREFRINTVNHLTSTTPNSQSNVLDKCPRSSPSGHCRVGSCKNGGRCVEEWEGFSCRCTLGFSGTTCEIASGPLGFGSNSRVSFVQKESYRRDQIISQSNSRKRRAIVTSSVMIRYRTTAPQEPLLIVSTRTDQGLLWVGSGKLVYTFGSTSAVLSDGRVHDGEWHNATVVVTGNQVTLRLDDKEHQQTLSGAVKFSDVAITKMVLGSSESPINVDNSKVHAFSGCISLFKIDGTPVPLNGSTDRFDITPTESVESGCSALCAGNPCGGGACSVNLETRVCAQVSEPPESLSIGIIVVIVFFGVLLIVIAIVFVLFRMRRQRKDPKNQAGPKENGHVNKSYNNSSPSHQDSGYGENDYNRQNNLNTTYSPNGLHRPDLINSDPTQRKPYEIDDGTVIIDNGDVNMNQLNDMPEHYDLDNASSIAPSDIDVHMHYRGYRSGYNDRSRERSHKRHKESPATGYKSRESPGPGALKLQTSGRLRSSPANNLEVPHNPSHSARSSPANVGMRNSPINQLSRQSPQVRAGPLIHSNVRGTPVSNIHHSRTESEHSLASHHSKSSTSSSVPRTVLPNGHVKSSRHKYYDPNSRQVKGLTVEEIEKLNARPRRPSPVSLLDAVSSSEEGRHMANIRMSTINSDVELVAPESSSEDSANDSFTCSEFEYENEKNKNEFDPNAMIFSKVSEVDNEHEDPGHPNRTSHSDGLDSNGGSFASTVGSSEEGPHAEHKLLNGHFAWDYLMNWGPSYEKLVGVFKDIASLPDGETSENQGESVAADCEEYV

>DmFat4

MERLLLLFFLLLAGRESLCQTGDTKLELLAPRGRSYATTYEQYAAFPRRRSSSSSPSGEMQSRAVDTSADFEVLEGQPRGTTVGFIPTKPKFSYRFNEPPREFTLDPVTGEVKTNVVLDREGMRDHYDLVVLSSQPTYPIEVRIKVLDVNDNSPEFPEPSIAISFSESATSGTRLLLDAATDADVGENGVTDQYEIVAGNVDNKFRLVTTANPSGDTSYLHLETTGNLDRESRGSYQLNISARDGGSPPRFGYLQVNVTILDVNDNPPIFDHSDYNVSLNETALPGTPVVTVMASDNDLGDNSKITYYLAETEHQFTVNPETGVISTTERVNCPQQTNVKSSASQKSCVFTVFARDHGSPRQDGRTYVTVNLLDTNDHDPIISFRFFPDGGKVATVDENAVNGTVVAAVAVKDSDSGLNGRTSVRIVSGNELGHFRLEEAADLHIVRVNGVLDREEIGKYNLTVVAMDQGTPARTTTAHLIIDVNDVNDHEPVFEKSEYSAVLSELAPTGSFVASITATDEDTGVNAQVHYDILSGNELKWFSMDPLTGLIVTTGPLDREIRDTVELSISARDGGPNPKFAYTQLKVIILDENDEAPQFSQREQNVTLGEDAPPQTIVALMTATDHDQGTNGSVTFALAPSVERLYPLQFALDALTGQLTTRRPLDREKMSQYEISVIARDQGAPTPQSATATVWLNVADVNDNDPQFYPRHYIYSLADDDDDIKLKKEVEKERILLHVTASDKDDGDNALIEYRLESGGEGLFQLDARSGAISLRGDAPASMHWKPHYKLLVSARDAGQRRSQQDAIVEIVLKSKLEMLECGQAQAGGYEFQMVEDHEQQRNSQPNREVGIVQVKSTNGKANSHIEYDIIQGDRAQNFRIDTRSGRITTARPLDREEQANYRLTILASSSSSSSAAASSVSYGQCIVNIAIIDLNDNAPVFALDRESEPTISLPENAAVGQEIYLSRVRDRDAGVNSRISYSLTNNPNQQFRIGPVTGVLYLQRPIRAEPGSLIHVELMATDAGSPPLSSKLSLSVLIADVNDHTPVFDHTSYETSLPETTKVNTRFFALAATDIDLGDNGRISYEIIEGNTERMFGVFPDGYLFVRAPLDREERDYYALTVSCRDAGQPSRSSVVPVVIHVIDENDNAPQFTNSTFTFSIPENAPADTFVGKLTAVDRDIGRNAELSFTLSSQTQDFTIDTRNGFIKTLRPFDREALVKVSRNAEASGEDGSLRGSMAGNYMLLEATVSDNGIPRLQDKVKVKVIVTDVNDNAPEFLRAPYHVTISEGASEGTHITHVFTQDADEGLNGDVYYSLAKGNEAGQFNLDSATGQLSLGRRLDRESQEIHHLIVVAKDAALKHPLSSNASITIVVLDENDNAPEFTQSSSEVSVLETSPTGTELMRFRASDADQGVNSQVVFSISAGNRRDTFHIDSITGSLYLHKPLDYEDITSYTLNITASDCGTPSLSTTVLYNVLVVDDNDNPPIFPSTAIVRQIKEGIPLKTPIVTVTADDPDSGLNGKVSYAISKQEPQLPQGRHFGINTETGVIHTLREIDRESIDTFRLTVVATDRAQPSERQLSTEKLVTVIVEDINDNAPVFVSMNAAILPPKFSTSKGSSTAVMQVHAKDADSSSNGLVTYEIVSGPQELFKLQRNTGIITFTPGPQFKQEVRYQLTLKSTDEAVQSERRSSEVYITIITPGSGGSESSVPQFEQRSKLSGSVYENEPIGTSILTVTAHLASAEIEYFVTNVTATGSRGQVDRLFDIDAKLGILSTAAELDREAGPEEYEVEVYAIALGGQPRTSRTKVRVTVLDKNDSPPQFLDTPFVYNVSEDLQIGHTISTLRAHDPDTLGSVTFLLMDGHDGKFLLEPSTGKLILNDTLDRETKSKYELRIRVSDGVQYTEAYATIQVSDTNDNPPLFEDTVYSFDIPENAQRGYQVGQIVARDADLGQNAQLSYGVVSDWANDVFSLNPQTGMLTLTARLDYEEVQHYILIVQAQDNGQPSLSTTITVYCNVLDLNDNAPIFDPMSYSSEVFENVPIATEVVTVSAKDIDSGNNGLIEYSITAGDVDSEFGIDSNGTIRTRRNLDREHRSTYTLTVTARDCADEFASFSELEETQLKLKYRSPRKYQQTRQEFLAHQKQQRLSSTVKVTILIKDVNDEVPVFISANETAIMENVAINTVVIAVKAVDNDEGRNGYIDYLMKEARDEDMGQSDPLPFSLNPTDGQLRVVDALDRELRSSYLLNITARDRGEPPQSTESQLLIRILDENDNSPVFDPKQYSASVAENASIGAMVLQVSATDVDEGANGRIRYSIVLGDQNHDFSISEDTGVVRVAKNLNYERLSRYSLTVRAEDCALENPAGDTAELTINILDINDNRPTFLDSPYLARVMENTVPPNGGYVLTVNAYDADTPPLNSQVRYFLKEGDSDLFRINASSGDIALLKPLDREQQSEYTLTLVAMDTGSPPLTGTGIVRVEVQDINDNDPVFELQSYHATVRENLPSGTHVLTPRATDKDEGLNAKLRFNLLGEHMHRFHIDSETGEISTATTLDREETSVYHLTLMAQDSSITEPRASSVNLTISVSDVNDNIPKFDSTTYNVAVPERISKGEFVFGARALDLDDGENAVVHYTISGRDQHYFDINTKTGVVSTKLELKTKTKSHDDLTYTIVISAMDQGEQSLSSKAELTVILRPPELFPTFAYMANSHFAMSEDVRPGKMITKVSATSPKKGLVGKIRYAIAGGIMGDSLRVDPNSGLLSVGQDGLDYELTHLYEIWIEAADGDTPSLRSVTLITLNVTDANDNAPVMEQLIYNAEVLEEESPPQLIAVVKASDRDSGDNGNVIYRLQNDFDGTFEITESGEIYTRMRLDREEIGDYAFVVEAVDQGVPHMTGTASVLLHLLDKNDNPPKFTRLFSLNVTENAEIGSFVIRVTSSDLDLGANANASYSFSENPGEKFRIEPQSGNITVAGHLDREQQDEYILKVVASDGAWRAETPITITIQDQNDNAPEFEHSFYSFSFPELQQSIALVGQIIATDRDKQGPNSVISYSLQQPSPMFSIDPATGEVFSKKAVRFKHSQYVRSPENMYALTVLATDNGKPPLYSECLVNINIVDAHNNPPKFEQAEYLAPLPQDAVRGQRIVRVHANDKQDLGTNEMDYSLMTFNLSSIFSVGRHDGWITLVKPIQVPPNTRYELVVRATDRGVPPQSDETRVVIVVTGENMDTPRFSVNSYQVIVPENEPVGSTILTVGATDDDTGPNGMLRYSISGGNERQDFSVDERTGGIVIQQQLDYDLIQEYHLNITVQDLGYHPLSSVAMLTIILTDVNDNPPVFNHKEYHCYIPENKPVGTFVFQAHAADKDSPKNAIIHYAFLPSGPDRHFFIMNQSNGTISSAVSFDYEERRIYTLQIKAKNPDSSMESYANLYVHVLGVNEFYPQFLQPVFHFDVSETSAVGTRVGAVQATDKDSGEDGRVYYLLVGSSNDKGFRIDTNTGLIYVARHLDRETQNRVVLTVMAKNYGSIRGNDTDEAQVIISIQDGNDPPEFIKHYYTSTISEAAPVGTKVTTVKAIDKDVRTQNNQFSYSIINGNLKQSFKIDVQTGEISTASRLDREETSTYNLVIGAIDTGLPPQTGSATVHIELEDVNDNGPTFTPEGLNGYISENEPAGTSIMTLIASDPDLPRNGGPFTYQLIGGKHKSWLSVDRNSGVVRSTTSFDREMTPILEAIIEVEDSGKPKQKSQHLLTITVLDQNDNPSTTRSLHIAVSLFNGDLPSNVKLADVRPNDIDIVGDYRCRLQKNPAQSQLQLAIPRACDLITTSHTTPIASVFSYTGNDGKHGDVSSKVSVAFQSFNNETLANSVSIMVRNMTAYHFLANHYRPILEMIKSRMSNEDEVILYSLLEGGSGNSTNLQLLMAVRLAKTSYQQPKYLIERLREKRSAFSELLQKEVIVGYEPCSEPDVCENGGVCSATMRLLDAHSFVIQDSPALVLSGPRVVHDYSCQCTSGFSGEQCSRRQDPCLPNPCHSQVQCRRLGSDFQCMCPANRDGKHCEKERSDVCYSKPCRNGGSCQRSPDGSSYFCLCRPGFRGNQCESVSDSCRPNPCLHGGLCVSLKPGYKCNCTPGRYGRHCERFSYGFQPLSYMTFPALDVTTNDISIVFATTKPNSLLLYNYGMQSGGRSDFLAIELVHGRAYFSSGGARTAISTVIAGRNLADGGWHKVTATRNGRVMSLSVAKCADSGDVCTECLPGDSSCYADEVGPVGTLNFNKQPLMIGGLSSADPILERPGQVHSDDLVGCLHSVHIGGRALNLSLPLQQKGILAGCNRQACQPALAAERCGGFAGQCIDRWSSSLCQCGGHLQSPDCSDSLEPITLGEGAFVEFRISEIYRRMQLLDNLYNSKSAWLDNQQMRERRAVSNFSTASQIYEAPKMLSMLFRTYKDQGQILYAATNQMFTSLSLREGRLVYYSKQHLTINMTVQETSTLNDGKWHNVSLFSESRSLRLIVDGRQVGDELDIAGVHDFLDPYLTILNVGGEAFVGCLANVTVNNELQPLNGSGSIFPEVRYHGKIESGCRGDIGQDAAQVADPLSIGFTLVIVFFVILVVAILGSYVIYRFRGKQEKIGSLSCGVPGFKIKHPGGPVTQSQVDHVLVRNLHPSEAPSPPVGAGDHMRPPVGSHHLVGPELLTKKFKEPTAEMPQPQQQQQRPQRPDIIERESPLIREDHHLPIPPLHPLPLEHASSVDMGSEYPEHYDLENASSIAPSDIDIVYHYKGYREAAGLRKYKASVPPVSAYTHHKHQNSGSQQQQQQHRHTAPFVTRNQGGQPPPPPTSASRTHQSTPLARLSPSSELSSQQPRILTLHDISGKPLQSALLATTSSSGGVGKDVHSNSERSLNSPVMSQLSGQSSSASRQKPGVPQQQAQQTSMGLTAEEIERLNGRPRTCSLISTLDAVSSSSEAPRVSSSALHMSLGGDVDAHSSTSTDESGNDSFT

>SpG-CDH

MAVNLRWNRTHNLCLSVIYILATLQLTLGLALPRINVPSNALPGFKVTEVKKSGQFSELLSDSDVHNLFQIAENGALEIKNSLEHLANSDIALKVRHTLRGQSWDDLLNLHVEDSSLEFSKKSYRGYVFESHEASQEVMGLDDLQIASTKPVSYQLAGEDSANFRLQVNPEGRVQIFTTVPVDSETTSQFHMEIKASAQDLHGNPPLMSLPAEVTIIVRPLPDNFRPFEDDIMYPPNEPVKMVRVRRDVLPGESVNLTESTAMDTVVHTITETGADLRYAMGTPVNDKFSIDEMSGDVTLLQSVDYEAYSTNPQEILNVQITNTSDPDALDTLVVTFDILDADDLPVWTMPVYPYIAVVPTDAPNQACIYTLEASDQDLGSDITYSLRAGGDGAFTVGETDGCVYTAIVSGFPGYVQDLEYILSVMATGTSGSFDGGVTGTVRVYGGSYPPQFSQEEYSASVLEDQANQAVIQVTASSFSRNVPLTYSIVGPENRPLHTINPNDGTITLVDAILAEDLLSYFLTVRATEQVVDGLSSEVRVNVMVEDVNNCVPTFGQDILSFNDVLETTAIGEPVGTVTATDCDVGPNAELTYSITTPSSGFTIGSDTGVLSPAVVLDYEEGERFYTFAISATDGGTPQLSSTATVVISIANDEEAPYFTPPSYRFRMDEVAQVNYEVGTVYASDDDVNDVLTLSIDGGDGTFRIDQFGVISVARAAVESSYMFAVVATDMANNEANATVEISVTDLNDNIPIFPECDNYRGEVSEDATIDTDVITVTATDSDQGSNAEISYKLSSNSDASRFFRVDQNGLITTNTLLDRETTAVFEVTVEAEDDGNPALTGFCTFTVEVTDVNDNFPAFPFTGYQTSISTSAAPLDVVIEVQADDLDLDSDLTYSLSAADGGECNTGYFIIEPATGRIIVRQDLSPLIGTDGNINEQDCIVTAEDVGRQATDVPVQIDVRSSDDTSYQVPTFSGTPYQVTIREDSMSQEVLLVSVTGTAQVGFNIIRGSRPSTNFNRNFGLSNDDNQFTSSYITVLEALDFESVERYDLIVTARYPNSPYTVETNVIITILDVNDETPIFPAFTFYGNIAENQPLPNNDPVITLEATDSDVTPGFSQIVYSIDGDQTDFRIDENSGEIFAITEFDREAVAEDTIPLTVIASDGINESRATVFITIVDENDNQPEFNGTFSFDVSEDVEMGYEIGMVTATDADISEDLEYFISGGNVGGAFTVVANSGVIRKAGVLDYESQTSYSLDYSVNDGFNTETTTVVVNVINVNDVAPQFGESSYSATVLEEDDSDIPRVLLTVAATDGDADAVADAVVYDLLGTGAGTLFTIDPQTGNITLTGVLDREEIPRYVLSAMATDDNGNGLSSYVDVIIEVEDINDNFPFFPDQEYVGSVDENMPPNTLVLAVVAEDPDTADNLVYSFPIPNSNFNIDSGTGEITTAIQLDRERVSEYELEVQVTDGTNTATTPVIITINDVDDNRPVFTQDPFPPASVLETEPVGTTITTVQVTDPDVDHMDKVVFTINSGDDADLFRIVPDAATLSGLIQVNKPLDFESGNANDFTLTIQVEDSQGTIGTPQTTTIEISIENANDLAPVFEEDMYAGAVSEGADVGSQVGTFTLTATDLDEPGGVLNYVIDPSTDPEGQFGIDANQRLVVASPLDRETVASYELKVYAVDNGDPPMSGTATINVVIEDVNDTPPRFAEDYTPSVKENSDAVAEVVSVRAVDDDDPASGPPFLYRVAGTPNEWTDYFTIEGLGTETSGDITISTSGRAIDREAIPYFDIVFLIEEVNTDELLTGTQTLSIMVTDVNDNPHLAATKNILVYSYEGNIPTTGVGKVAVEDPDILEDKTYTAEGEFPDGFQLDTDTGDIVMLEGTPAGIYTMQVMVSDGGMFPDVVSTVIVTVKDIPQEAVFSSGSIRFSGTTAEELIDPNAEGVSKLDSLKVILAEAAGAQLENFDIFSVLNVEGMERTVDIRYAAHGSPYYPADKLDLAALSVSDQIAALGLDIAQLPVDLCVKENVCESSCTNVLVVDPTPTVVDSGSASFVAITSVLQAQCICGARTEAPGSCDSIPCLNGGTCVDVHGGTYKCECPYLFDGPNCQQTKRSFNDGYATFSTLRQCEETSLSIEFITETPSGTLFYNGPIQPTEVDDAIDMILLELTNGKAKLTINLGSVDGTTLNLELEVPKDNLGDGQWHRIDVYRNGRSVEMTVDRCEDAPFAETSSSSTLDTSNCRSSGETPGENKFLNVNTPLFLGGMSPDYNPDVVVPGGFDGCIKNLVSDGFLYDLADPGIFDKSDSGCLRTDGQCTVDGEPVCENGICEADLGSFVCICFPGFNGEFCSIVLPPYDFATDSYITYELLDESLYNDARSSNYHIMVRSRQENGLIWSISSANTYEYIRMEMVAGELQADWHLGDKPVSVTMGNFSINDGAWHAINFDRYDSVVTIKIDGGGGVKEIQNRESAFSGLDIDSNSLVIGAFVDLNTVTDDFMGCMNDPRINNNFLGMEGINVYATATKSAGVSEGCPSEVCDSNPCPGQDLVCTDYWRFYECECPVGEELSGPETCLAIIDCDPNPCYNGGTCTEGVPTGFNCTCAPTYYGTQCEATIGGQGASLGITPLGIILMIICILLIIILLLGLVLYTRQRDRKSALTFAIDPEDDIRENFINYDEEGGGEEDNDAYDLSTLRKPVDAISAGSIEKKPAMPVSEVPVGNRPSGVDPNVGDFINDRLKGANDDPEGPPYDEPHVYDFEGDGSSAGSLSSLNSSSTESEQNYDYLNDWGPQFRKLADMYGS

>LvG-CDH

MAVKLRWNMTRCMCLSAIFLLATLQLTIGLTLPKINVPSNALPGFRVTEVKKDGQSSELLSDSDIHNLFQIAENGALEVKNSLKHLANSDIALKIRHTLRDRSWDDLLNMYVEDSSLEFIKKSYRGYVLESDEANQEVVGLDDLQVNSTKPISYKLAGEDSDDFRLQVGPEGRVHIFTKAPIDMDTTSQYHLEMVASDLDLSKATAELLIDVATANNQVPPLVKESTNQFHAYGNDMLFPPAEPVKMVHVRVQRDVLPGESVDLPESTELDVVVHTITVVGTDLRYAMGSPANEKFSIDEMSGEIRLLETINYESYAIPQEIVNVVITNASDSEVSDTLVVTFDIQDADDPPVWTMPVYPYITVVPTDAPNQACIYRLQASDEDPGSEITFSLVAGESGAFSVGETDGCINTVVNAQNPNYEDGREYVLSVRATGTSNDFVGAVDGTVFIFGGSHPPQFSQEEYSASVREAQNNQMVIQVEASSFSRNVPVTYSIVGPENRPSHTINADSGVITLTEAVLVEDMQSYFLIVRATEQGGNQLSSDVRVNVMIEDINNCVPTFGQEILSFNDVSETTAIGEPVGEVTATDCDVGVNAELTYSITTPNSGFTIGPDDGVISPAVILDYEEGERYYTFVVAATDGGSPPTSGTATVVISIANAEEAPYFTPTSYSFRVDETAQVGYEVGTVYASDDDVGDSLTLDIPDGGGPFDINDSGVISMIAAATDSSYTFEVVATDQTGNSAMASVQVSFTDLNDNIPTFPNCGSYSGTVAEDAAIDTSIITVSASDLDQGSNAEVTYQLASNTDANQVFKVDQDGLITTKKLLDREDTSSFVVTVEAEDGGNPSLTGFCTFSVVVTDVDDNYPEFPFTDYKASVLSDAPSNSEVVSVPADDLDSSSTLQYDLASSQEECRRYFTMSPADPVIITRGDLSAISNNDVSCIVTASDGIRESRVDVDIFVRSPDDTTYQVPTFSNTPYQVTINENTTIGDVILLVSVDGAQEVGFDIVRGSRPSTNSDGTFTLTTRDDNLQSDLTLVEKLDFEAVQEYELLVTAQYVGVGQEFIAQTPVTVTIEDFNDVAPKFPAVTFYGNIAENQPVSNSDPIITLQATDGDVTPEYSQISYSFVNAESDFSIDEDTGEIFALKVFDREAIEDSSIALTVRASDGVNQDLATVFITIVDENDNEPEFNGTFSFDVLEDVGMGYDIGTVTATDDDISEVLEYFISGGNEGGAFTVDAEEGTIRKAGVLDYEARTSYELQYSVNDGKNVATTTVTINVLNVNDVAPQFDQSAYSASVIEEDDSNLPRILLSVAATDGDADAVDDAVVYGLVGTGAGTIFTIDSQTGNITLTQALDREEIPTYNLAAVATDDNGNGLTSYVDVTIEVEDINDNAPVFPDQEYVGSVEENRPPNTPVVAVVAEDPDTADDLMYSFPTPSPDFNINSQTGQITTARQFDRETPPSEYEIEVQATDGVNTASTTVTISIDDVDDNKPSFSEDVYPDASVLETEPIGTTITTVQAIDPDVDFRDAVEFSINSGDPDELFRIVADAATLQGLIQVNKELDFETLATNEFTLTVAVTDSQGPTDSGRPETAIVKIIVENANDLAPVFDQDVYDGAVSEDATVNSQVGTFTFSATDGDEPGATLNYIIDPTTDPDGQFAINENQQLIVASPLDRETVASYELKVYAVDNGEPPMSGTATVAVTVTDVNDTPPHFAQDYNPSVEEGPIEANVEVVSVEAVDDDDPPSGPPFLYNVAPQPNDWTTFFDIEGLGTSTSGSIRVSTTGLEIDRETHPYFDIVFLIAEVGTPEALTGTQTLTIMISDVNDNPHVAITKDILVYSYEGNIPTTEVGKVGVEDPDILEDKTYEAVGELPDFFQLDSDTGDITMAEGTPAGVYEMDIRVSDDGTYESVVSTVIVTVKDIPREAVFSSGSIRFSGTTAEELITPDSEGVSNLDRLKVILANAVGAQLANFDIFSVLNVVGMERTVDIRYAAHGSPYYPADQLDLAALSVSNEIEELGLSIAQIPVDLCVKENVCESSCTNVLVVDPTPTVVDSGTASLVAITSVLEAQCICGARTVAPGNCDSDPCLNGGTCEDVHGGTYRCTCPYLFDGPNCQQTKRSFENGYASFSTLRQCEETSLSIEFITEVSSGTLLYNGPIFTPTGDDPIDMILLELIGGKARLTINLGSTDSTDDNLVLEAPTDDTQLNDNEWHRIDVYRNGRFVEMTVDRCMGVFFAETSSSSTLDTSSCRVNGTTPGENNLRFLNVHTPLFLGGMSADYDVTVPSGFDGCIKNLVSDGFLYDLGTPGTSSKSEAGCPRTDGQCTDDNGMPVCNNGTCEADIDSFICICFPGFNGLTCDVELTPYDFAIESYITYELLDSSLYDDARSSNYQIMVRTRQENGLIWSISSANTYEYIRMEMVQGELKADWHLGDKPVSVTMVNFSINDGAWHAINFDRYDSVVTIKIDGGGGVKEIQNRESQYSGLDVDENSLVIGAFVDVNTVTDDFMGCMNDPRINHNFLGMEGTNDYAVATKSAGVTEGCPSDVCDSDPCPGSILVCTDYWRFYECLCPEGQEEVEDDPDTCMAIIDCVPNPCANGGTCVEGDPTGYTCDCPSGYYGDRCEAAFGEQGASLGITPLGIILMILCILLIIILLLGLVLYTQRRDRKSALAFAIDPEDDIRENFINYDEEGGGEEDNDAYDLSTLRKPVDAISAGSIEKKPVAPVSEVPVGRPSGVDPNVGDFINDRLKGANDDPEGPPYDEPHIYDYEGDGSTAGSLSSLNSSSTDSEQNYDYLNDWGPQFRKLADMYGS

>ApG-CDH

MAAAVRLFATLLFILIVLFAVTLVSGFALPRINVHFDARPGTVLTEFHNEGQSSELDPPSSYLSITDDGRLLTTRPLVDLLGTSLTVHVTHRKGGRSWNERIIVRVINSGKDSKLHFADSRYEGVITENNPVGQKVQGLSDLRVTVPVYAQDMPVQYELLGPGSEKFHQEVSEDDSIVRVYASAELHVAEKEFYKLTLEAVVNDEVAVTEVHILVLGLHPPVFIFPSFVYSIAENAPNGTVAGTVFAADEDRGANGHVTYHFLKPNRYFKLGQETGKLTVASPPPAGEYNMQVVARDDGTPSLESAPVPLQISVDSASSGKIPYGNDMLFPPDYKPSKRLRRDVLPEQRVPVPENTTVNTVIYNIASVNDTDRFAFVNNPGASFGLNETTGEVILLAELDYEETTQYNLDIQITNVENADKSDTQRIIIEVQNVDEPPQWEMIVYPYIEVVPVDAPSGASLYQLRASDPEGFTVRYYLNSGGSGMFTVDEDSGWIKTSLSPGQTYEQDRKYVLSVYAQDENGSSIPAEVNIFGGTHPPQFTKEVYQATVVEEVNPPQNVIDVRAFSFNNLAVTYSLLNNNDVDSRIPRHTISASSGQITLLDKVDRETLSSYFLDVRATEDTQSNDGLSTTVRVNVDVTDINDCNPVFGQKTYSFRNIPETIGTDTLIGSIEATDCDADPNNILTYTLSGAQAEKFRVVGSGDLYPASKLDFDVGEGFYELRATVRDQAGGTAEAIVWVYLTDLNDEPPVFLNTENYVYYVDEDAVNGYIVGTVYASDADKDSVITFSLQGTYPFRIDSATGVISHVGTNNLPEASYSFNVTATDGTYTSYAEVEIVVNDINDNPPVFQDCASYAPSISEDAADGTSLITVVATDADKGANAAIKYNIPGANIPFRVDADTGEIFLQDPLDRERNPTIDIVVTATDQPANSFSKTGFCVITVTVTDVNDNRPVFRLERYVVSVSSSTPTRQTILTVNAEDADAQSSITYRLTDQSPNLFGIRQTGEIFPLMGLSAYEGRTFNFKVVADDSVKEGTSNIEVNVLTALAVDRPPVWVTVFDDIRLPENTTVPQVIRILEATPQSSNEVGFSIVIGQIPETNSEAQFGVRNLGGVNRAELNLDIPLDFEVTSEYSLQVDATDDGTRLSSFGTQKVIVEDVNDETPQFTLSRFFAAVPEEATPPFDVITVAAVDADTVKEFRTITYSFDASFPDAQFFTINGITGQIFSQIKFDREVKDEYALQVIATDGAPSSNPALGGEPNKDYVRVTINVVDINDNPPVFSSPIYNVVVREDRPVGYEIIQVTATDVDSDSVPRYLITANNTGGVFDVDPATGQISIASPLDYETQTEYWLQYSATDGLNVEYTTIRIQVTNVNDERPVFSQPVYRDSVAENDANVPRQLLQVSAQDGDADADQGAIRYSLEGSGTDGVFSINSQDGWISLNQELDREAVGLWKFLVKATDGVGTPTSLTGYADVEVTVLDQNDNDPFFPELEYRGSVPENSVAGTQVMQVTAIDYDDPTGFGRLTYSTVTAAGIDNDGSALFEIDSSSGWVTVKSGASLDRETQDMYTLRIRAEDSSGAFAQTDAIIEILDLNDNKPEFIGGPYTTAVLETEPVGATVYEVSVSDPDIGFTDEVQFGIVSGNVGNRFEIVADPLTLVGLIRIANPLDCEALDKQYALQLTVTDNANGQSQTSITINVQDVNDVAPRFLPSNEYIAPSVLENVAVGTLIQTVTATDPEEGAFGLFTFAIDAASDRDGLFTISAPIGSGAERTAEVRTAKALDRETVETHVLTLLAIDQGVPALMGTATLNLTLEDINDTPPTFAQDYRPMIRENYPEIQSVGTISAKDSDPTGGAPFTLVVEPNTDTESFTWRDVGENQLNISSKPISFDREVQAFYSIPIRITDLAPNGNSAVNILVVEIEDENDNPHVGISKDMLVYSFEGNIPESPIGVVGVSDPDTIEDKTYTPNLSETLKQYFRVDADSGEVTILAGTPADTYSFTVLVRDDGTFPDQTSTVNVEVRDIPEEAVRSSGSFRFQGVTAEDLVGLPVGGGQSKLDVLKGILADIVPAKSENIDIFSVINVPGETDQVDVRYSAHGSPYYPPEKLDGAALENKKRIEDELGLTIGMIKIDMCLLESACESACTNVLEINPQPTLVNTPTASFTSVTTRVVAQCECGANIRQPGPCDFDACVNGGTCTNTQGGGHTCDCPTGFDGPDCQQTTREFKGQGFAHFGTLQQCEDTHTSLEFITTVPEGVLLYNGPMIPLTGDMPQDFILIQLVGGQPQLQIDLGSGSLTLSIPSARDLGDGKWHRLDVYRNGKDVEFMLDRCKDASVAEVSSSSTRQTDQCKQTGQTPGGNKFLNVNTPLQLGGRDLAAAFTYPSNISFASSYNGCMKDVEQDSTLYDLQTPGKVSNSDPGCSRLACGECNNGTCVGDFDSYVCLCKPGFTGTTCDQATPAYDFALDSYVRYQLKAPVAIDSREGNYMVAFRTRQKEGLLWTITSANRLEFTTIELIDGYIRSRWNLGDGEHSVYLNQYAVDDGSWHSVHLERYDFYVTVRIDGGGGVRQAENRDSTFSTLEVDPNSVLVGAFVVRVVEISQDFQGCMNDPRINNFYLGFKEDSTYATPQPSSTVTEGCPSYDPCASNPCPAPFICVDLWRKYECQCKPGFYEENGQCINIDACVPNPCLNGGTCTDLDVGYRCDCTEDYLGENCGTRRERVASPLGFGIGAILALLLCLLIIILLLLAFVFYKRDRDRKQALAFAIDPEDDIRENFINYDEECGEEDQDSYDVSTLRKPVVPVPMDDYVKPPTTEAPLSRAPRLPGDDPNVGDFINDRLKDANDDPDAYPNDSLKEYDFEGEGSSAGTLSSLNSSSTDGDQNYDYLNDWGPPFKKLADMYGGGEDD

>PmG-CDH

MAAAVRLFATLLNISIILVLILILVSGFALPSINVHFNTRPGTILTELQNQGQSFELDPTSSKYFGVTDSGKLVTNRPLVDLLGSSLTVGIRHRIGDHSWNEQINVRIVNTGRDSKLHFADSRYQGVITENNPLGEKVRGLSDLRVVVPIYAQGMPVQYELLGQGSDKFHLEVSEDDSTVGVYADAPLQVAEKEFYKLTLEAVVNDEVAMTEVQIWVLGLHPPVFAFSSFIYSIAENSPVGTIAGTVFATDEDMGENGHVTYHLLKPNRYFTVGQESGLVTVTETTPIGEYKMQVVARDAGTPFQESAPVPLQISVISSSSNMIPYGNDMMFAPDYKPSKRFRREVLPEERVPVNEDTAIDTVIFTVSSLNDTDRFAYVGTPNPLFGLNETTGEVTLLGQLNFEQATVHEFEVEITNVENTDKRDTQRIIIEVQNVNEPPQWEIVVYPYIAVVPVDASTGASIYRLRASDPEGSDVRFYLNSGGSGLFTVDEESGSIRTSLLEGQTYEQGRKYILSVYAQDENGSSIPAEVNIFGGSHPPQFTQEVYNVAVEEEKIGEQNVIDIDAFSFSNVAVTFALLNSETNQPIPRHSIDPSSGLIKLLENVDREVLDSYFLEVQATEDVQDGLSTTVRVNVEVTDINDCVPKFERETYSFRNVIETIDTTTPIGTVAATDCDADPNNILTYSLIGAQADKFRLDSQGVLYAATGLDYDVGDGSYELEAQVQDQAGGADTAKVWVYLTDVNDEPPVFLNTDGSVYYIDEDAPAGYIIGTVYASDADKTDTITFGIQGSSTFQIDPQTGVISRIGTGNLPEASYRFNVTATDGKYTSDAEVEIQVNDINDNTPVFPDCSTYTPSISESAGIGTFLIKVQATDADKGVNAEIAYDIAGSNNPFTIDAVTGDISTQDPLDRETTPTIDIVVTAQDNPGSIFSNQGFCFITVTITDVNDNRPTFPLEDYVVIISNTVQPFTTILTVQVEDADLPTPAGFTYGLTGQNPVLFEIDQDGEISPVADLSGYDDNTFNFKVEAEDGPNKAKSNVQVKVLATGVDQPPVWVSTFPDITVMENISINDVIGTLEARSQSGNGVGYRVVQGQIPQTNSEGQFAVQNVGDSMIANLYISAPLDYETTKFYKLQMEAYDDGNRLSILGTQTVNVQDVNDETPQFPVTNFFVAYPEDVDPPFLVTQVQAEDADTEPAFKTITYSLDPSFSDAPYFSINPTTGALTSLIKFDRESEDSYSVQVVATDGAPSSLPSAGGPNKGYLRVVINVVDKNDNPPVFDAPMYIRTIREDEPVGSEVIQVTATDVDPDSIPRYLITANNTGGAFEVDPATGAISIASPLDYETQREYWLMYTANDGLNVASTVIRIQLENVNDEKPEFEAPVYTAEVSENDPNVPRQLLQVKAVDGDADANQADIRYSLEGTGAGTTFTIDAVTGQITLTEMLDRESVSIWKLLVKATDGGSTGTSLTGYADLEVTVLDENDNSPFFPELEYRGSVPENSPGGTRVMQVTAIDYDDPNGFGRITYSTISSSEIPNDGSGLFQIDGSTGWVTVKSGAVLDREKNDTYTLRIRASDVPNSEALTDAIIQILDVNDNAPEFVGGPYATSVEETQPVGATVWEVSVTDADVDFNEEVQFNIIGGNVGGSFEIVADPLTLIGIIRIARQLDYESPNKLYTLTLTVDDNSGPDSQSQTTITIDVLDVNDIAPTFNPAEYVSANVLESVAVGTYIGTVSASDPEAGLFGQFTFSIDPASDPDGLFSIEEILPVGAVKRADVQTAKPLDREKVETHVLTLRATDLGVPPLTGYATLNLTLDDVNDTPPTFATDYRPTIKENLAVVQFVGYISAKDEDPTGGPPFTLVVEPNTDTNSFTWFDVDGLTGNNLSISSKAISFDREAQATYSIPIRITDKAPNGNSGVQNLIVEIEDENDNQHYGTTKEMLVYSFEGNIPDSPIGFVGVSDKDTLEDKTYFPDLSDVYKQYFRVDEDTGQVTILAGTPAGTYNFDVRVADDGKYDDQTSTVIVDVRDIPEEAVRSSGSFRFEGVTAEELITVPSGGGETKLDILKNILAEIIPAKPENVDIFSVINVPNQPNTVDVRYSAHGSPYYPPEQMDGAALANRDGIAQALGVTIGMIKIDMCLLESACESACTNVLEIDPQPTLINTPSASFTAVTTSTVAKCVCGANTQQPGLCDFDACLNGGTCTETQGGGHTCQCPTGYDGPDCQQTTREFKDGFAHFGTLQQCEETHTSLEFITTAAEGVLLYNGPMIPVTGDMPEDFMLIQLLGGQPKLEINLGSGTLSLSLPATTNLGDGKWHRLDVYRNGKDVEFMLDRCKDAAVAEASTSSSRQTDQCKITGQAPGDNKFLNVNTPLQLGGIDESPGFTYPVDITFAASYDGCMKNLEQDSTLYDLETPGKAAGSEAGCSKLVCGECNNGTCEGDFSTYTCLCDPGYTGEKCDQTTPAYDFATESYVRYQLKPTIVIDSREGNYQVSFRTREDEGLLWTITNLNGLEFTTLELINGYIRSRWNLGDGEHSMYLDQYAVNDGDWHMVHLERFDFYITVRIDGGGGVRQMESRESTFSTLEVDPNSLLLGAFVVHVVDISQDFQGCMNDPRINNFYLGFETESDYAIPQPTASVTEGCPSYDPCASNPCPVPFICKDVWRKYICICKPGFEELGNTCVAIDDCDPNPCLNGGICTDRESGFDCECPDGYRGDICDVKVVPRVGEPLNLSIGAILAMILCILIIILMLLAFVFYKRNQDRKQALAFAIDPDDDIRENFINYDEEGGGEEDQDAYDVSTLRKPVQPVQPMDDYIKPPTTVVPLSHAPRPTGDDPNVGDFINDRLKDANDDPDAYPNDSLKEFDYEGEGSSAGSLSSLNSSSTDGDQNYDYLNDWGPPFKKLADMYGGGEDD

>ArG-CDH

MASAVRLVAAPIHLALALTLLVTLVHGFALPTINVQYNAKVGTVITEIKNVGQTFILDPEGSDIFRITESGSLVTKRPLTGLLDTSHTLTIKHTKDVHSWYEQINVKIVDTGKDSKLHFADSRYEGFINEKNSPNSKVLGLSDIKAVVPVYAQDMPIVYSLLGDGSENFYFDLSDEELGVQVFAETVLSIEDKEFYQLKLRAVVNEEVAETEIQVVVLGLHPPVFMFPVFAYSIVENAPAGTIAGSVFATDEDRKGNGRVSYYLQKDNKYFAMQSKIGKLVVISPPPAGIYELDVVARDWGSPSLESVPAVIQIEVIPSSSVRIPYGDDIMYPPSDAQQGKRQRRAALPEQTVSVPESAAIGDVVYSVENVNDTDMFALNPQNPLFTVDAASGVVSLAEMLDFERNTMEDFTLDITSSIDAIKRDTQRVVVNVDDVDESPKWDLIVYPYIHVVPVDAPSGASLYQLMASDPEGATVGFFLKSGGNGLFEVDESSGWIKTKLSQGETYTQDEEYILSVYAEDATSNRVNGQVSIFGGSHPPQFTQEVYEATVTEEVNGQQNVITVSAFSFRNVGVTYEILENNVDYPHTIGTSGIITLTRKVDREQMSSYFLDIRATESGVDNPMSAEVRVNVQVLDVNDCDPKFERSTFSFRDVDETISTDTAIGSVAATDCDSGTNSILTYTLIGADGDNFRITETGQLFPNTRLDYDRGDSYYSFEVQVVDEAGGEDTATVRVFMKDVNDEPPVFLNTEGYLYYVDEDADSGYKIGTVYASDADPSDIITFAIAGTNLFQIDSSTGVISRLGTGNLPDARYIFNVTATDNGNTHTSYAVVEVQVNDINDNSPEFPDCGTYAPEVSEDASDQTFVIKVTATDADKGENAEILYRIIGSNLPFSIDQDTGDIHTQDPLDRETNPSYEITVSATDKPSNTFSNTGFCTFTVTILDVNDKRPEFPLETYVVFLSDTAQILDVFFTVTAEDADENSAIEYSLDTDQFGINQQGGIFPVVEVGPLSGTTVTFSVIASDGQKNSQSNVQVTIGTDVNPPTWDQSYGPLSIREDSPVNDIVQAVLARPATSGNSITYDIVIGQIPETNSNDQFSILRSPGTDGGNVIVRSQLDYETTKSYHLEMQAYSVETRLSAFTTLEVTLIDVNDESPKFTLGTYVAAVPEETANPTIITVEAFDADTTAAFKRVTYSIIQGENSNDFKIDPTTGVITSLIEFDRETKASYVFQVLAKDGAPSDIPALGGEPNSDQASVTINVIDINDNSPIFAENMYEKSVPENLPVGSEVIIVTATDVDSDSVPRYLITANNTGGAFDVNPTTGQISIASPLDYELTTEYWLQYTANDGLNVATTTIRIIVGNINDEKPIFEQDVYTVNVLEEDDSFPREILQVSANDGDAGADQTQIKYSLEGSGAGDKFTIGTSTGQIMLNAKLDREEESEWRFVAKATDANGGGLTGYADVIVVVDDINDNSPTFPDLEYRGSVPENSVGGTPVMTVTAVDLDDPTTPNGKIGYSTVSGTDTDNDGAELFDIDGNTGEVKVRPGAMLDRETQDFYQLVIQAQDGNGAGDTATTTAIITISDENDNAPSFVGGPFTAAALETLPRGATVIELPATDADVDFNDVVQFEITAGNSNNHFAIVSDPVTLIGIVQINEPLDYESSPPSYTLTVTVSDNINPSDLTTVTITKVDVNDITPTFTPSAYYPSNVLESVAVGTVIQRVTATDSEAGDFGVFTFAIDPETDPDGLFMITEPIGSGATRSGEVRVAKELDRETQAQHNLILWATDLGEPPLRGKANLTLVLDDVNDTPPRFEEYNATIEENVGTVQLVVKLKATDDDTTGADQFLYEVVTTNNPDTEKFTYEDTAGNTFTIRTKPIIFDREEQAFYTIPIRITELIPGGNIGVSDVIVTVLDKNDNPHFGRTKTMLVYSFEGNIPTSPIGFVGVLDPDTIEDKTYSTKSEFPSEYFQVNEDTGQVTILAGTPGGLYEFDVIVKDENVFPEAESTVIVDVRDLPEEAVRSSGSFRFEGVTAEELIQTPADGSLSKIDILKGILAEIIPAKPENVDIFSIINVKGQPNTVDVRYSAHGSPFYPPEKMDGAALENRDRIAEALGVTIGMIKIDMCLMESACESACTNVLEIDTTPTVVNTPSASFTSITTRTVAKCVCGADVQQPGPCDFDACLNGGTCKDTQGGGHTCTCPTGFNGPNCEQTMREFKNGFAHFSSLQQCEQTHTMVEFITTLPDGVILYNGPMTPLTPGDLPMDFILIQLESKKPVLYINLGSGTLRMEIPSTADLGDGKWHRLDVYRDGKSVEFMLDRCKSANITEASSSTIQQTEDCKSTGVTPGDNKFLNVNTPLQLGGVDQSSAFTYPSEITFGAGFDGCMRNVEQDGAVYDLQTPGKTVGTEPGCSRLECGECNNGTCEGDFNTFVCLCHPGFMGDVCDTSTPPYDFATDSYVNYQLLPAAQLDNRMGDYQVTFRTRMKEGLLWMITNQNGLEFTSIELVDGFVRSRWNLGDGEKSVHLDQYAVDDGDWHTVRLERFDYYITVKIDGGGGVRQKENHESLYSRLEVDPNSLILGAFVVRVVEISQDFIGCMNDPRINNFYLGFSGSTTYGTATPSNGVIEGCSSYDPCLSNPCPASYICIDLWRKYTCGCPDGSMEDEANGVCKPIDECDSSPCLNGGKCVELVIGFRCDCLDGYIGSICGTEKAFVITTLGLDLGAILAMILCLIIIIILLLAFVLYKRSHDQKQALALAIDPDDDIRENFINYDEEGGGEEDQDAYDVSTLRKPIDPIPMDDYIKPPVTEAPLNRAPRAPGDDPNVGDFINDRLKGADDDPDSNDTMRDFDYEGEGSSAGSLSSLNSSSSDGDQNYDYLNDWGPPFKKLADMYGGGEDD

>AjG-CDH

MAAYILNGRAYRVLSLCLLNGFISLALGLPTQNLFVDRNFKDNTNNLEYMSTELYSSKFNFSKEAYSGYILENSKVGSSVLGLEDLILPFTWEARDAGDLTLSGDGHQHFILSNTLSETGMKINFLTTAILDSESKSSYQFTLRAPKLGASTVVHIVRVTKSDDSLIFRPYLDDMIVPESKSRRRRDILPGVTATVNEDAPIGYVVYKFNESSSHRFMMTDNNPRFEVDYNDGEVRVSSALDYEMVNTETITIVTWDSQQSMTSNTTSLTITILDVDEPPVWMMEYIPYIAVVKPNVANGVFVYQLYAEDPDDANIEISYEFFIDTAGDIFRVDETTGEVFTNFGPGQSLVGITEVMLTVSANEKDTNMSSYTDIVIKVGTYPPQFTREIYYVDVTEGSSPRTLLVVKAKSFERMGIVAYSIAEDTSGISHSINSEGVITMNSVVDREVRDGYVLTIRADDIGSEIGSGYCKVYVTVQDVNDHVPEFSQVIYAFRGISEDIPIYRSVGKVVVKDDDGDVFGKILFTTPSNVPFWVNEGGNVYPSGPLDYEIDSHRYSFIVTATDLGGSSSTATVRVYMTNINDAPPVFSQPTYSFQVDENAPTNYKINNVYATDPDGDGLTFSIKREGQGYDLFEVDPSTGVVSRSGVTGMLTDASYTITIVATDDGACCDQTPRVNNIAEATVVIVVNDINDNTPIFPNCNSYDPVIFENATLKSEVITVLATDADKGSNGEVTYRLEEEKTKSNYFYIDPETGVLTTNKFLDREEIDTFSLSVLAEDGGKNKLTGICQFTVTVLDVNDVYPLPVRFTTTLARDTAVGEVVARMEAFDPDLEDQDGLVFSLTDPDNQYFEMESDGTLKVKSSLESYPDNEVSLRVDVRDKKNQKAQTSVTIVFTDARVNPPQFIQDPHPDESVSEDIGVLTVVFSARAVAADGSPVIYELLSGETKETNNPVKFKIDLEGNVFVSDSLDYEDTTRYELRIKAQDGNQESFIVVNVNVIDVNDKQPEFILSRGYAANIAEGDYQDAERTVIEVLAVDADTEQNFRTISYSQTPGPNSDVINHDFNVDPVTGTITTNANFDREETSNYIIYITAEDSAPSAIPSVPAGESNTATVAVRINIIDENDNKPVFDKGVYEATVMEDVAIGTSIAQITGTDADEGGSAGLNYLIAGGITQPDSIPYQNAVFEVEAHTGRIKVASSLDYENIQSYRLEYVATDGKHETVATVIITVENVNDIEPEFDQSQYIAEIVEENSGESFVLEVTAVDRDEGSAGAPSAIKYSLEGEDPDYFTIREDSGQIFVLKSLDHEQKNEWRFLARATDENGFGRSGYTDVKIVVNDINDNGPVFNAARYEGTIKEGDPPTDRYVMTMTASDADSPLYSANTYSIEQIPPSELPPGVLDGSALFEIGRTSGRIEAKSTANLDREANDIYYLQVKAEDMDPTESRSATATATIRVEDINDNAPIFDPTNYATTIPETLARGSRVLDILASDRDIGVNAELTFTIESGNNDGKFMIKPNPVNMQNAILVLNKDLDYESGDQTFSLRIQATDSLSSATAEVVITVTDENDEAPRFTNLPEEFSFPEGFIPQDAIITVSATDDENGENGRIDRYEIDPSTNEGGLFEIDPATGVLRLIKMLDRETVPVHNLRILAIDAGTPPMTGEGVLTIIVIDINDECPSFREDAYNPVVMENQDSPIEVVQVVPVDPDIIGTQKPFVFEVANGDYKPEGGQFTINQAFKLTQDGDTAIVSTLKEFDRELQELYQMEIRISDAHTPPNSCINILNIRIGDENDNPHQDGSKSMSVYTYEGGSGTGIGTTPVGPVNAPDKDSDDIDEKTFTKIDASDDVWDHFDVDRESGNILIKAGTPSGTYTMKVLVSDGKWEDSTATVTVKVTDLPEEAVYNSGSLRFTGTTAGDFIQEPNSKYSKLRKSLAAIIGAKEDNIDIFSVMDVVGEANTIDVRYSAHGSPYYKAERMNSAVRKSKSQVESETGLVITMVKIDACFMDEGVCEGSCYNNYTVTKEPYIIDLQSQSFTGITTYTTAACGCAGREIFPDGCSFDACMNGGTCTSTPEGFSCECPSGYEGGRCEEIKRSFSGSGSYAWVETLEQCEETVTSIEFITNKDGTLFYNGPMTGESGQPSDFILLELVNKRPQLQLNLGSVTSTFEFGASDLGELTDNEWHHLEIRRNGKEVEMVVDHCLSAKSTISEAGSSSIIDDSSCRIAGTVAGTSRLLNVNTPLQLGGRATSGWDYGSISFANNGFDGCIRNLIQDGKLYDLSDNNVGVSFKNSQEGCPRTDQNCIDNSDGSNKCVNGVCDANIDTAVCICYPGFTGVRCNEETPAHDFEVNSFVDYTVKAAAGLDTIGYEEHYHSMVRTWEESGLVWYIANLDTGEHTTIEIIDGTMRLRYNHGEGEFKMSLPNYRINNGAWHSVEYDRYGNYVTLKVDRGGGTRQVEGSPGTFQQFIASPESLRVGDSLGNEVIPLDFIGCMNDLRINNNYLGFSSSKFAEVTDIENVKEGCVSNACVDIYCPVTFICNDIWRRHECICPVGTLLEGTTCVAVNACFGVICDGGYRCEVNEAGKGVCVPNKDTLVKEATLSTGAIIALLICILIILILVLMFILYQRKMRQRKEPLFVDPGDDLIENLVHYDEEGGGEEDRDGYDINTLSKPIGVIDHTIEKKRRLPESAVPAFTGPDNVGDFINGRLSNADDDPNAPPYDTLHTFDYEGDGSTAGSLSSLHSGSSDDSQNYDYLNDWGPRFKKLADMYGGAEGGEY

>SkG-CDH

MAVRAPVLLFLAVAFTATLIHSATLPRLTVPHNVHSGYEIARLRHVGQKYSINAVGDAANIFQVSSDGVVTTRRKLTNRANTVVVLGIDQTTDTNSWQEIFNVYIIDNVSNEVHIVITVEDVNDESPQWSMPVYPYNAVVPTNAPTGVSIYQLQASDADSNSLNYNLDSTSDDQGLFEVDQTTGTIRTKLQGGQRYQEGFEYILRVRANDGVNYSPLAAVSIIGGRRPPQFVKARYEVDIPENTQSGYALVRVEAFSFTRGTVVTYQFMSQGADLMIFTIGTTSGQITVNKALDYEQGPRRYTLRIKAIEEGSEGLSSSVDVVVSVIDINDCTPRFEQSLYTVKNFPEDASTNAEVVTVLATDCDSGTNAQLSYSIDNNAFQINSLGEIRPSVKLDYESTNSLYTIIATAKDHGSPSKTGTATINVRIKNYNDEPPRFSQNTYKYFVAENAPDGFQVATVIATDPDGDGVKYSISAGNEDRNFEINRDTGVITLTATRDLTQASYLINVTAIDDNTCCDGLGSIHRSQAIVIIQVNDVNNNRPVFPNCANYNPTVAENSPEGTTVTQVLATDADVGTNGQVEYSIVRRETADLNFRIDPNLGIITTARRFDREVKRIYGISVAATDLAPDPLIGICQMNIEITDVNDNTPHFEQRSYEYQLREDTQVGTSFLRVAAQDADTGYNAEISYSLSDEEYFSVNNATGWVYVKNAIYLSQDDKIYLNLMATDQKGTGRQDTVPLSITISDINNEPPVWDASTYGPISLREDAATETVVARVKATSILDDPRVTYSLVQGQRPESNNPMRFYITTDREDKSGELKVFHRLDYETTPSFELRIRSLNAAQVPLPSYATVEINLIDVNDNVPLFSLPNYGASVPEMSIQGTFVFQVTAEDADTGSNAEITYSIIEDPETDDWNSFRINSRTGQIYTTQRFDRETKGSYLIEVRSQDGAPSDRTFDPDNPDAPNSDTAAVRIVVSDINDNAPAFDSTEFYATVEEDRETGFAITTVTATDEDEGANSQIRYQITSGNLGGVFRVVPEVGRIEIAAPLDYEQTRSYALTFVASDGLNENTAIVNIDVVNVNDNPPEFQAEYVPSIWEEDENIPVYVTTVYAIDPDLDSDDDHNIVYSLQGQGANEEFVIDPHSGVIHATKALDREDQAQWILQAVAADEDGAGLDGFADIVISLRDINDNAPTFPNGPYMGTVPEHSLAVPGTGPGTYVMTMAAVDLDDPNEGDNAQCTYKIIENAEKDGEVIFAIDPVTAEITTLVDWLDREDVPVYNIVVEATDGGGLTGSATATIELEDINDNAPEFTEDKYYRAASESLLVGASVLTVTALDMDVGVNAQLDYKITDGNIGNHFYMYNDIANNVGIIKIQEMLDYEDITQRQFTLTLEVCDPDFCDTAECLITVEDFNDNPPVFDPNLYQASVYENASLNTILGTVYATDADHSSTNNSRFYYTIAESTNPKNQFHVGPEDGIVTISNELDRETVEYHYLTIQAVDYGPPSLTGNATFQVTVLDINDNPPTFAEDYKPLVMENTPPPEYVVTIRAVDPDGPGNQGPYTYWVDNWNNILDNFTFEQIGDEVEIYTKIDFDREEQKYHYMSVVMADVHGLTGTETLTIEIDDMNDNPHSPGTKEIFVYNYKGEMPEFGLGELISKDPDALDGETTESAVGEVYAPDPDDKDDKSYYIVGDAPDYFWVDEDTGQTYIKEGCPDGVYEFTVLVSDGVWPDQNSTVIVTVKEIPEEAVMSSGSVRLDDMTAEEFISVPDNGESKLELFHQALSQYLPAKYENIDVFSVINVPDSNPPSVDVRWSAHGSPYYPPESMNMQIKLNQKEIENLVGITIGMVPVDLCLGEGACESSCTNFFMASTNPTLVDAGDNTLVGVTTIQEARCVCGARIAVHGPCASNPCYNGGTCTNTPSGYVCKCSKGFEGPDCEDLKRSFSGNGYAWYETLQQCGETHTSLEFITESPNGILLYNGPMTEVRDNEPDDYIALELVRGIPVLYLNLGSGTLVLQIDKSPRLDDGEWHRIDVFRNEKKVELMIDRCNTATVAETENSSTIDTSSCKIEGTTPGTNKLLNINTPLQLGGIILEPDYVYPADYLHTTNDFDGCLKNVDQDSKLYDLGSPSKSQNSDQGCKQTDQHCYGNGGVYLCGNGTCIGSWDDFFCICFPGYYGASCNIETPAYDFSDESYVTYELPPTIPLDDYKSYFHIMVRTRQENGLIWHMSDTNGWEYIRLLLHEGYLVTTFNLGDTGEGFSRKLPNYKINDGVWHSINLERNGNHFVVKVDGGGGVREMEFRMGSFYELAVDSTSLVLGAQVDVQQVTMDFIGCMKDPRISNVYLGMDGDMDGTSATLSEGVSSGCFSNVCDSDPCMEPLICFDIWREYICRCPVGEEFLNGTCIEIDECLSDPCLNGGACIDGVNGYICICPEGYSGIHCEQLMVFAYVGATATLSMGAGIAILICLLCLIILVLVCIVYKRRREKNVLGYMDYPEDDIRENMIVYDDEGGGEGDQDMYDIRPLSKPVDSDSPVNTDKKPLIPKEAPVRTMAVPPPQEHPPPQKDAPRRRAPPKGDSPDVGDFINDRLGDADDDPNAPPYDTLHLYDYEGEGSTAGSLSSLNTSSSSEPEQDYGYLKDLGPPFQKLADMYGGADSD

>BfG-CDH

MFRTVRSKMANRRALFPLTAGERTAALVISLFVFLASAASKDDVRTFGIPPQFSVFVPHDVEPGYTVGRLVRPAFVSPHCEGARLFSVDIRTGEVTTRKSLAGHKDESVSLAVSWPDGSRETDILYISVMDRQKLPTIPSDFHVGAIGENLPPGTAVGGLGNLWVHSPAMGEHFPAAHNFTYHLISDTDLPFTLGHHATKEGETVEIYTTKMLDRETQGEYDMTVTVSLGTEDENSGSKVWAWGWNSVARALIHVTVADDNDNPPVFTNEQYEAEILATTDRKPVVQVHAVDPDNGNNATVVYSLSPSVPGFTIVPKTGQIWTTGSAHLGTQNRHHLVIHARDSGDPSLVSQPASIIVQVVKSGDESDIDKSMEKSRSKRRALPNIQVSMAENAEVSSPILDVNNQPSDPVNDQFTIIYPEPSRRRVTIDGSTGVVRVGKLLDREEQEWENVTVEITNPYRQTNRVNVNIRLTDLNDNSPQWTMVPFPYQAVVPVNAPRGTLVYQLTAEDKDLGENAEFQFFLESDGDGRFELDTSTGDVTTSGLPFTANQEYIVHGRARDTGRNESPQAVVSIIAGNRPPQFMQQTYDVTVPEETLDQYPVVQVEAMSFRGSRISYSLKSSMDMFAINRLNGQITLHRGIDYETEQRQYTLSVEATETGVNPMSSIVQVRVTVADINDCTPEFANPIYSQRDVVENIPSTKAILQVSASDCDSDENQRITYSVDDDAHFRIASDGTIYPNSQLDYEVPNNMYEFLVKAVDHGTPVLTGSATVRVRMANINDEPPVFSQRTYTTFVAEDAEPGGLVATVHATDMDGDGITYAITGGNDGTFVIDAKDGIIRLSDNPSLQGSHYTLNVTATDDNASGGPGSLTGTAVVVVGINDINNNKPDFTQCAEYVPSVPEGQPSGTFVIQVTASDADQGSNGRVTYSIVHREDSETMFNIDADSGEISTAVVFDREERREHAVTVTATDNADNPLIGVCQLIVHITDVNDNDPVFENNKYAAVLSENTALHTSFLRVAAQDADMGTNAEILYSLSEEVPQYLGIDRQTGWLYVKSAISQTQRIIRRVIATDGGGRSTSVRLDVAITDVENEPPMWEREYPAVTIPENTPIDQVVATVKASSTLLDSRITYSLVDGQLPETNKPKRFYLRTNRATRTADICVFHPLDFETTPRFELKIRAENAARIPLAAYTIVEIRLTDVNDEVPDFTAKSYAMSVAEQSPKGTLVGQVTAVDADTGDAGKVTYSISPDPDPSLRDWEKFRIDPSTGEVFTKDMFDREKKSFYLLEVKAEDGAVSDRVELSNVNIPNSREAYVRISISDVNDNSPTFPRTQYEASVDEDKDVGYSVVTLTANDEDEGANAKLRYQITTGNVGGVFDVVPEIGTIVVAAPLDFEAVQEYELQLVASDGKNENTTKVNIKVNNINDEEPEFTRNEYTGSIREEDSNTPIPILQVTARDPDRGASDADIRYSLQGQGANDEFSINIITGQIYASKSLDREERAVWRFIALATDEKGRGLVGFSDVAISLGDINDNAPEFNDEPYVGSVLENTAAGTSVMTVTATDADDPDVGRNAKLTYRITKNAKQNRVNLFRIDRNTGKIFTTVGNLDRETTKEYTLVVRAEDGDGLWGTGTVTVQVGDINDNPPAFNQRIYSTTMSEGLGKDGIVTTVAASDADVGDNARLFYSIVGGNRNNQFRMDNSDNKGIVRVNRRVDYEEPTQRKFNLTLEVRDLDFTSRGYCVINVLDYNDNPPVFNPVYYEKEIYENATVGTLLRILSATDRDTGNNAVFKYSINPSSDPDQQFLIGAQNGILTVNKALDRETLPQHRLTIQATDIGPPSLVGNATFVVNLLDINDNGPVFKEDYRPAIPENTEGPLHVQLIEAVDYDSDPPNGRPFVYAVPDPNPLARAFDFKDNGDDTAVVTTKRSFDRETQELYELLVIIWDSGQPQMSATNTLTVTIANENDNPHYGGTKEVTVYNFKGAMPDSPIGIVHAPDRDDGALVNPDVKTYIFESTEPKYFWLNESSGMAYIREKTPAGRHTFSVRVSDGIWPDAVSSLVVNVKEVTEEQLVNSGSIRFDGTTAEEFITPGKDGQSKYDLFRQAIAKVVGAQVQNVDIFSVLNVPSRRSVDVRYTAHGSPVYPAHKLNGLAAQNRQTLEELVGLTVAEVNVDPCVQEICQTGGCSSRLETSDTPTLVNSGSASFVSVTATMVHECTCPQRAEVRGLCDSSPCYNGGTCINTPTGYRCKCRNGYDGIDCQQTKRSFRANGFAWFDPLPQCLDTRISLEFITKHPDGLLLYTGPVAPLATGEPRDFMAVELVSGKPRLTINLGDGPLQLDIAVTTTVNDKKWHRLDIIRQGSTTRFMLDRCKNSVITETVGTGRGTSAEDRTGCEAIGRVPGKSRFLNVLSPLQLGGIAQKSLPYPTLTNLDFQGCIQNLVQDTKVIDLAEAQLSQNSEEGCTQTDANCQPNSFTPICGPNGTCVGTWDSFYCTCHPGFHGDRCQQATPEYSFDSESWIRYQLRRPVSARNTQHQLMFRTRVPNGLLTAAASRDRQEFTSLELMNGKVQYRFNIGNGVHIVRLEDFTVDDGEWHVVNVERFGNEVSLKLDGGGGQREITIALGSHREILIDQNSVLVGANVTRAAEEQNVYNDFIGCINDIRLNEEYLPMEGENSVAMATTEGIQPGCWDHEECLSAPCVIPFVCVDLWRGYICRDDCESAPCLNGGICDVHAYGFVCTCPVGWTGVICDQEDQTSARIVGVVGISIGAVVAILLCLLVLLVLVLACVMYKKQLKAATTKYLAVDGHDDVRENIISYNDEGGGEEDQESYDIRKLQKPDRESPTRAKRPLEPRIDVPPDDVPQMRPTLPQGDNPDVADFIGARLHDADNDPTAPPHDSIQPYDYEGQGSTAGSLSSLTSASSEDDQDYEYLDNWGPQFRNLADMYSGAGGGASA

>DmG-CDH

MAARRCLNQLRQRYITNRFNICTCAIFLISLPFILAIEETTFAGLSAENAARMLAGSPGDVEKSSLSHHSEMSLVLPHDTYPGFSIKKFKTHPVKINGSSHSGAAAYHMLDTDYSKYFTVLEDGVVMTTADISPLVNRPVQLVVVEQTPNATNTHNLQLFVMHRNDMLRFSGSLLDASGEVRENQPAGTRVRGVPLMQAFSGSILDEELATPKKVRYTIIDGNVDDAFALQERKANKNIQISAKSLVINGDDESGVWLVTNRPLDREERAHYDLSVEASDVDGLDRTVSKIQITVLDENDNRPIFKSLDYKFAIAGQKSASMESNSSVTYQRFAIMGKVEATDADGDKIAYRLKSPSNVVIIVPQTGEIMLAGEPTSNELLIEVIAHDLRYPSLVSAKPAKVLLEFLAAEPVSFIMQHLEHDDINNHSHHREKRRVTRAVRPTKRIEFTEADGDTEGKSVFQLEKETDKETFKIRDDNPWVTVETNGAVRVKKKWDYEELGPEKTIDFWVIITNMGHNAGIKYTDNQRVIILVKDVNDEPPYFINRPLPMQAVVQLNAPPNTPVFTLQARDPDTDHNIHYFIVRDRTGGRFEVDERSGVVRTRGTDLFQLDMEYVLYVKAEDQNGKVDDRRFQSTPEERLSIVGGKRAPQFYMPSYEAEIPENQKKDSDIISIKAKSFADREIRYTLKAQGQGAGTFNIGPTSGIVKLAKELDFEDLRQPHVYSLIVTATEDSGGFSTSVDLTIRVTDVNDNAPKFELPDYQAHNVDEDIPLGTSILRVKAMDSDSGSNAEIEYLVSDDHFAVDSNGIIVNNKQLDADNNNAYYEFIVTAKDKGEPPKSGVATVRVYTKNKNDEEPKFSQQVYTPNVDENAGPNTLVTTVVASDKDGDNVRFGFVGGGTSSGQFVIEDITGVIRLHNKAISLDKDKYELNVTAMDDGSCCVNGDQTIHTSTAVVVVFITDVNDNKPVFKDCSTYYPKVEEGAPNGSPVIKVVATDEDKGVNGQVKYSIVQQPNQKGTKFTVDEETGEVSTNKVFDREGDDGKFVSVTVKATDQGDPSLEGVCSFTVEITDVNDNPPLFDRQKYVENVKQDASIGTNILRVSASDEDADNNGAIVYSLTAPFNPNDLEYFEIQAESGWIVLKKPLDRETYKLEAMAQDKGYPPLSRTVEVQIDVVDRANNPPVWDHTVYGPIYVKENMPVGGKVVSIKASSGIEGNPTVFYRLMPGSTAQTNKFHTFYLQQRPDNGDTWADIKVNHPLDYESIKEYNLTIRVENNGAQQLASEATVYIMLEDVNDEIPLFTEREQETVLEGEPIGTKVTQVNAIDKDGTFPNNQVYYYIVDSPRNEGKEFFEINLQSGEIFTKTVFDREKKGAYALEVEARDGAPSARPNSNGPNSVTKFIRIGIADKNDNPPYFDKSLYEAEVDENEDIQHTVLTVTAKDHDESSRIRYEITSGNIGGAFAVKNMTGAIYVAGALDYETRRRYELRLAASDNLKENYTTVIIHVKDVNDNPPVFERPTYRTQITEEDDRNLPKRVLQVTATDGDKDRPQNIVYFLTGQGIDPDNPANSKFDINRTTGEIFVLKPLDRDQPNGRPQWRFTVFAQDEGGEGLVGYADVQVNLKDINDNAPIFPQGVYFGNVTENGTAGMVVMTMTAVDYDDPNEGSNARLVYSIEKNVIEEETGSPIFEIEPDTGVIKTAVCCLDRERTPDYSIQVVAMDGGGLKGTGTASIRVKDINDMPPQFTKDEWFTEVDETDGTALPEMPILTVTVHDEDETNKFQYKVIDNSGYGADKFTMVRNNDGTGSLKIVQPLDYEDQLQSNGFRFRIQVNDKGEDNDNDKYHVAYSWVVVKLRDINDNKPHFERANVEVSVFEDTKVGTELEKFKATDPDQGGKSKVSYSIDRSSDRQRQFAINQNGSVTIQRSLDREVVPRHQVKILAIDDGSPPKTATATLTVIVQDINDNAPKFLKDYRPVLPEHVPPRKVVEILATDDDDRSKSNGPPFQFRLDPSADDIIRASFKVEQDQKGANGDGMAVISSLRSFDREQQKEYMIPIVIKDHGSPAMTGTSTLTVIIGDVNDNKMQPGSKDIFVYNYQGQSPDTPIGRVYVYDLDDWDLPDKKFYWEAMEHPRFKLDEDSGMVTMRAGTREGRYHLRFKVYDRKHTQTDIPANVTVTVREIPHEAVVNSGSVRLSGISDEDFIRVWNYRTQSMSRSKMDRFRDKLADLLNTERENVDIFSVQLKRKHPPLTDVRFSAHGSPYYKPVRLNGIVLMHREEIEKDVGINITMVGIDECLYENQMCEGSCTNSLEISPLPYMVNANKTALVGVRVDTIADCTCGARNFTKPESCRTTPCHNGGRCVDTRFGPHCSCPVGYTGPRCQQTTRSFRGNGWAWYPPLEMCDESHLSLEFITRKPDGLIIYNGPIVPPERDETLISDFIALELERGYPRLLIDFGSGTLELRVKTKKTLDDGEWHRIDLFWDTESIRMVVDFCKSAEIAEMEDGTPPEFDDMSCQARGQIPPFNEYLNVNAPLQVGGLYREQFDQSLYFWHYMPTAKGFDGCIRNLVHNSKLYDLAHPGLSRNSVAGCPQTEEVCAQTETTARCWEHGNCVGSLSEARCHCRPGWTGPACNIPTIPTTFKAQSYVKYALSFEPDRFSTQVQLRFRTREEYGELFRVSDQHNREYGILEIKDGHLHFRYNLNSLRTEEKDLWLNAIVVNDGQWHVVKVNRYGSAATLELDGGEGRRYNETFEFVGHQWLLVDKQEGVYAGGKAEYTGVRTFEVYADYQKSCLDDIRLEGKHLPLPPAMNGTQWGQATMARNLEKGCPSNKPCSNVICPDPFECVDLWNVYECTCPAGYKSSGSTCVNDNECLLFPCRNGGRCRDHHPPKKYECHCPMGFTGMHCELELLASGVLTPSRDFIVALALCLGTLILLVLVFVVYNRRREAHIKYPGPDDDVRENIINYDDEGGGEDDMTAFDITPLQIPIGGPMPPELAPMKMPIMYPVMTLMPGQEPNVGMFIEEHKKRADGDPNAPPFDDLRNYAYEGGGSTAGSLSSLASGTDDEQQEYDYLGAWGPRFDKLANMYGPEAPNPHNTELEL

>CgG-CDH

MKSMGWNGPSAYVICYLTIIHFCCINNAFGETNSLVIPFIIPVNSFITKLSTSHAEFSINDKLANSLFDISKDGSLTTKKELISHAGRSFTLSQSNDNLEDPFFDIVHLKISTSWNLKSFTSQTFHGMIRENLPLDSEVRWNENISIHFDAAKFTLEPTSKHFKLKVLKSTDSSAGSQVKVVTTSETVHASDREHVFILKAWINHIDFTSTILKIKVIDANDCIPKFSKKVYHATVGGDITHAPRNSILKVSTTDQNNDKIIYSLDDSSSFYITPNSGLLFLKEVHPGFHKLKVFATDTNGNQCSAFVLIHVLSSTLKFRPFHQISKRQTKIMTVEKTYVVLENQTAGTVVFGVASKPPPPAGTEFYSILYDSIESFQVDTRGNVYLKNGLTLNYENPRHRNVTIRFKITSSLSQDVQIWTVHLNVTDVNDEKPRFVNQPRPFLATVPVNPRVGQLVYELLATDPDTNSDIHYNLESAGEGKFTIEHIEGDFGRLGRIVTTVSGNGQFEPGQIYDLVVSAQDVAAPGVQKSNFMVVSVLVGSRPPQFYREQYVAFIAENNQPGYKLQSEGTTLIVQAKAFQSKSEKGVVDYQLTDSSGYISSVFSINSDGEIEALQSLDYETSPHSYSLKLVGTEKSTGLSSASQLIVYLEDDNDNAPVFELSTYTNVTSEGTPVNSLLFSVTATDKDSGSNAELTYTVSDDNFVIETRRDNITGKYVGDLRVAKKLDYDSRADRQYKFDVRATDHGNKSFSGQASVVLYVTNINDEAPVFSEEKDDIVAKIREDQRAGSYVTTVQAVDPDGDNIKYYFSPKHQNAENLFQIEPNSGIITLTNAIPPSIPSYVLNITAYDDGSCCGGYPRLSSSSFVIIMIMDINNNNPTFPSCTYAPSVLENQPPGTRVVDVTATDPDRGENGKITYSVVTPNNQEQNFQVNAENGTVYTKKMFDRESEAGLRGYSVTIKAEDHGESQQLNTLCTFWVKIKDLNDNPPLFDTESYMQTVLRSTSVNKRIMGVLAVDKDKEINAEVQYSLVDNPGEYFRVDTDTGGLYLQKSLSTVPHYQEKIMLRVMAKDKGTPPLSSNATITITLTTGNQNPPTWNENYEGQTYLVNETSPIGYPIATFSATSHVDPPLDGVSFALVDSNGNSQQMVEDFRVDSQDRTMKLKVANQLDVSVKSLYTLRLRVTNQGLTPLSNEIRITVKVLDMNNKQPQFEGLDPTLSNSYRGSVPENEDAGQSVITVKAVDPDLQEPNNVVSYSLVPDKYGAYQKFQIDPQRGLISTNYTFDRETLKVYYITVMAQDGRASDTPYHEPPGTPNSATTQVMVTIADKNDNTPYFEKLLYEKEVDEQPADSSKSILTVKARDIDDADTLTYTITSGNTGNVFGIKSKTGDIYVAKDLDFETPPNLYKLNITVNDGYHSNYTEVKIRVRDINDNTPEFSQSEYIVTSIVEEYQPPPGGLFLIQVSATDKDTSRQTFFRYSLNTEYGDTFQINSRTGVITLHKSLDRDLPYGHAEYQFNVLVVDEPGSEGALVGYAYVKVKPQDINDKTPEFTQALTGFVPENSEKGKIVMTVRAVDYDLGENGTVYYTLGNNRPADPQTGDSLFVIDGKTGLVISNTRTLDREKVDQYLLPVVATDGGKVPLSSTATLTIQVTDKNDERPRFNKKIYRATLPESQKSGLIITVAATDDDIGNNSKIAYSLKSDLNFFSISSLPSNKGALNVYKPVDYEDPNQRFFNLTVRARDNNPLHYDEAYIEITVTDANDEAPKFTEPVKTDTFPENIPEKFLLHTFTAYDKDSYPNNEFYFQIREHSDRFYVEQEGNQAHVKIQAGLDGQTLDRETNDYYSIQVLAIDKGIPAQTGTATLRIKVLDINDNPPTFAENYRPIVMEETAPIITVGTFSAMDPDTLIHGPPFVFELPPCKENPTCHNGDETFSLIFDPDGAKGNGTCTVLANKMFLREQQKFYYLPVIMRDTGGRSNHMSGTSTLTIEIGDKNNNQHTDGYKEIFVYNYKGMFGDIEIGRANANDPDDWDYIDKHYTMINPNGMDQFFNVSKINGTITMKRGVPTGIFEFKVEVHDFKVFTEKNATATIRVTVTEISDEAVFRSGSVRLSGITAEEFVETSSDISGLSHYDKFRRLLAEKLRVEVSFVEIFSVMKNGPFTDVRYAAHGSPWYPASKLDGIIALNKDEFAAVAGGQIVMVNIDECQKEICESGGCSNILYVDERKPSVVNTKTKSFVGLGTKIVAECQCKARDFARPLKCAPDFCFNGGTCVKDNWGDVKCECHPMFDGPRCQQTTHSFSGGYTLYPPLAQCEESITSIEFATLKESGLIFYNGPVDKMKKGDPQDFISLSLIQGYPELIVNHGTGPLTLRVMGKDKGGITRMPVLNDGNWHHLHIIRKGREVTLTVDHCRNAEFHVRSENQEDRRPCEATGSTSGQNKYLNVNQMMQMGGRYSTPDFPKDITTERFEGCIRNLYHNGELYDLYTSFNHPGVNHANGCPAEESACSSISGRKCGDFGVCQMVTLGQSEVECVCKPGYRPSRPGSSKCDTETTVVDFTKKAYMTWMLKDNFFNMLKKTTLDVHIKFRTRDPDGGVLLHLPSNSYEFISLQIINGHVAVIYNLKDYDNSMMENVISLSSAPAANGQWHSLSMKRIGRWFQLKMDSGEGRYYNETWGSLHESQFFNLKRDQIVSGSLVKFRPNANFHGKDLRDTCVTDIRINNEWFPMMMEENAHSEAAEVDLISQVKMGCVRDDCAHARCHDLGQVIKQICVPLWGTHDCRCPPGSMPGVGNSCLQIDHCLGINPCKAGGICINLPFPGTFRCLCPKGWTGPTCNQQASTVEEVVTAGGIKMEVLVIIIVSLVAVTFIAFLVFLLWRSLSKQDKRKSFLYDDQYDDIRENVVNHDEEGAGEEDQTCYDISRLRKPDSTFDRGYYYSSLSTKYQKGLPLDSPEVNAFIADRIQDLNEDPITLPHDNVMVFSYEGGSSTAGSLSSLNTNSSDNDQDYDYLHDWGPKFSSLAVMYRAEEED

>SpPCDH9

MTPLRILAPLLIETFFLWMSVVSARDIFYDIDEGVGPGTVIGNVADDLAITIDANTEFSMLGVPNETAYVSLDSQTGELTTVLDLDREELCPGSSALCEIEVNAIELGTREVITVKVTINDINDHAPEFRDDLTNMSIPESVVPGTRFPLSTASDEDIGENAIQGYRLSDEYAETFGLVQNEFPGGLIIIQLEVIGSLDRENKDNYVMTLYADDGGDPVLSGVTTLNVTVLDSDDHSPVFDRTSYQVSVAENIGVGQHIIQVRASDPDTGTNGQIIYDFGGSVSAKIIELFEIDSESGWLSVKSDLDFEDESSHQVSIRATNNVPNPLPDFTTVTVNLIDVNDNKPRLTISALGDGGRFKHIAENSPEDVDVAYVRVTDMDTGVNGQAILTLEDDFGHFYLESFREGQYFLKTAGVLDREDIDFYNITILAEDRGSPVLSSRRRFAVFVDDENDNSPIFSSSVYHATISENNEPGHRVATVQAIDKDELENGEVVYSLLDDKDGSFGIHPFNGVLTANVSLDREDGESIDLMIRACDRGQPQGCSDVPLTVRVLDMNDNGPTFGGDLIEMRIDENKPIGTIVGRAIATDADEGDNGRLRYSILTDAVFRIDEDSGRIYSTAELDREIQELYHFTVRAVDDGLSPKTATATVVVTVNDGNDHSPEFTVPSANNDIRFIPVSADPGLHIMTVESEDEDKDENAAVSYSISHGNAYGAFGIQANGQVVTAQDLEPKWEGVHDITIRATDGGNPSASSTAVLRVVISNEGFKRSLPPANFTALFNLTIDYYLNLGNNAASSRGILNDWPMIVIISLAGCAVILVIVFLLVAARCRTKNREQGKYIVPSGEELFATRQAAKPADNSGKTSDTDSGLTSTASSSSNIPSKNIRKWRAQQDRDRDSMGSSNPGSTTNLGTMPPGITGVANVDLRLGNRQMTTFGSNSDLHSESIASITSARRTPDPDAEVQRLLRKLRRDSEDRNSEGGSGSSYDSGHGGEPEIERESGYRTNLSSADGSSRRSASYPRIAALAAQNINNTQTPSGGHRHSYMASPHCTPECLTLGHSDECWMPNPASISKSKTVHFSKHDDNRSSGGSYRSYHSNADRPPATSPKPPNPAIAFGNVSKVTPKPKPIPNGSTTAGLGQQPPATRDLNGYYGNNPAVIVEDERNGNAMPLTPIREHPFERRSSQDAPSLVVTKHSPDKYKNNNDVDRNGDVRYRNDLNRGHINGVNPLHRNSMNGTTPNGSTPNGGQLNGIQNSRSNNSRNSSGDSHISSEDSADTYSGSSAASSEMKPVRYSPDEVEAVLEKCGGDRSAGVSADGDRMYTDWV

>LvPCDH9

MTPLRIFLPLLIQISFLMMSVDSASDLFYYVDEEVVPGTVIGNVADDLAITVDDSTEFSLLGVPNETMYVSLDPQTGELTTVLELDREALCPGSSAPCQIEISAIELGTREVITIRVTINDTNDHAPEFDNAVTNINIPESVAPGTRFPLSGASDEDIGKNSIQGYRLSGEYADIFGLVQNEYPGGLIIIQLEVLHNLDRENKDNYQMTLIADDGGDPPQSGGTILNVTVLDSDDHSPVFDRASYEVSVDEDIGVGQQIIQVRASDPDTGTNGQIIYDFGGSVSAKIRELFEIDSESGWLLVKSDLDFEDDASHQVSVRATNNVPNPLPDFTTVTVNLNDVNDNKPRMTISAIDGGISGGRFKHILENSPEGTDLAFIRVTDVDTGDNGRARLTIEDDFERFRLEQITEGQYFLQTAAELDREDIDFYNITILAEDRGSPVMSSRKRFEVYIDDENDNFPEFSSAEYHATISENNEPGHRVATLQATDEDEFENGEVVYSLVDDRDGAFGIHPTNGVLTANASLNRENGQYIELMIRACDRGQPQKCKDVPLTVRVLDMNDNAPTFGGELIELRIDENQPSGTLVGRAIATDADEGDNSRLRYSILTDAVFRIDEDNGRIFSTAELDREIQELYHFTVRAVDNGLSPKTATATVVVTVNDVNDHSPEFILPSANDDIRFIPVSAEPGLHIMTVESEDEDKDENAAVSYAISHGNTNGAFGIQANGQVVTAQYLEPMWEGVHDITIRATDGGSPSSSSTAVLRVVIANEDFKRSLPPANFTSLFNLTIDYYLSVNNGASSRGILNDWPMIVIISLAGSAVILVIVFLLVAARCRTKNREQGKYIVPSGEELFASRQAAKPADIGGKISDTDSGLTSTSASNASTIQSKNIRKWRAQQERDRDSMGSSNPGSTTNLGTLPPGITGAANVDMRLGNRQMSTFGSNSDLHSESIASMTSARRTPDPDVEVQRLLRKLRRDSEDRNSEGGSGSSYDSGHGGDREIERENGYRGNLSSADGSSRRSASYPRIAALAAQNISNSQTPSGSHRHSYMASPHCTPECRTLGHSDECWMPSPSSITKPKTVQFSKHDDNRSSGGSYRSYQSNTDRPPATSPKPPNPAIAFGNVSKVTPNHKPIPKGSTNPGLGQQPPTTRDHNGYYGNNPAVIVDDERNGNALPLTPIREHPSERRSSQDAPSLVVTKPSPDKYRNNNDVLNGDVRYRNDLNRGHINGVNPLHRNSMNGSIPNGGTPNGVLPNGRSTSRNSSGDSHNSSSDSVGTYSSSSAASNEMTPVRYSPDEVEAVLEKCGGDRSGGVSADGERMYTDWV

>ApPCDH9

MAHSASILCIIFTCNLLTVAWTQEITYTVMEEHEAYTVGNLATDLDIETSQDTRFQLLTATWENQTYFLLNGETGDISTTQPIDRETICALNSGTACQYELEVLVSPLSQYHFIRVTVIIEDVNDHPPKFSKAILPLEIPETIALGTKIPLETAEDPDVGVNSVQEYSLLNDFGGKFGLFVQRYADNSVSLQLEVTGELDRETRSSYLLTLLAHDGGDPPKTGSVILNVTVLDSNDNAPQFLRPNYVVSIEENAAVGTEILQVEAMDPDWGTNGQIEYSFSSSVTQATRQVLQIDEVTGMITVKGLLDFEQQQSFQLVIRAANRVTNPVPDFTTVTINIIDINDNYPSLTVNALEAGANGWVYISEDTPTGTPVAFVKASDPDGGSSGRVRISLSNTLSDFGLQEVSDGQYFLSTLRTLDREAVDVYNISIQAMDQGSPPKTTISHLVIIVEDINDNPPYFATPIYYANVDENNHLSLPVATLSASDPDEGINADITYQLWTQRDMFAVDASSGVITAEVVLDREAQSSVSLFLSACDHGVPTRCSNATVIIDVQDQNDHQPVFEEPGYQFSIPENQFVNSVVGFTKATDEDTGPNAQLTYAIREADSNGASDFFHIVPQTGKIVTTRVIDFEEHKEFQFTVTAADHGIAPQTSEVLVSITVRDENDNAPEVISPNMGNDTFFIPQSAEAGFLVVPIQASDRDQGMNGQLSYSISGGDILGIFGINTAGQLLTTQKIASSWEGAHEVTIQISDGGTPTQITPLELIVVITDLDFNTSLPAAVFFERFNLTAADFGLEDKFNSGDRDDDGTQHASPLSSWPVITAIVLGSLFILLVLIFLLVHFKRRSKTKPKQVYTVPSDLPSSDSRTSTDYFSEVHNMTPQRKLSTDSSRLSVTASLGSVQSKNIMKWRAQAAQANGTLDSRLGNHQMTTFGSNSDVMSDTVARRTPDTDAEVQKLLTILRTETDAVSDNSNRSSYDSGQGDHDEHDSTQDIFPIQHSVRGAPRSSSCPKLSGLSGQGQIPSRDHYMASPHCTPECATLGHSDQCWNHQELPRRPRSASASIIPQQPIHRPISLYADKSVGYDMNTQMPSSNKGSVSWSDETPVTNVGRGYRDNYGYASAQNYSRSNGSVGTDSHYNGNYGTVARGNSGKQRHPLNNSHNTPPQDHVNNKHYRNSGTPLILSPITEDPMEITDTRMAARNRDAYSDYSTCRGSGDQVYPISTLVRHKNGEVYTNYGHGELQSLTQDAVDADVGYQTDAASGYYPQNSVQTCL

>PmPCDH9

MALSASVLCISLICNLLTVAWTQEITYTILEEQEAYTVGNLAADLNIETTPVTRFQFLTATWENQTYFRLNGLTGDISTTGKIDRESVCALNELTCQYELEVLVMPQSEYRFIRIHVVIEDVNDHPPRFSEAILPLEISETVALGTKIPLETAEDPDVRENSVQEYSLLNDYDGTFGLFVQRFVDNTISLQLEVTAELDRETRSRYLLTLLAHDGGDPPKTGSVILNVTVQDSNDNAPQFQRSSYTVSIEENAAISTEILQVVAMDPDWGTNGQIEYSFSSSVTQAARQALQIDEVTGMISIKGLLDYEQQQSFQLVIRAANRVANPVPDYTTVTINIIDINDNYPSLTVTPLEAGPNGWVHLSEDTSTGTLVAFVKASDPDGGSSGMVRISLSDTLSDFGLQEVSDGQYFLSTLRTLDREAVDVYNISIQAMDQGSPPKTTIRHLVIIVEDINDNPPFFATPIYYANIDENNRLSSPVVTLSASDPDEGMNSEITYQLWTQRDMFAVDASTGVITAEVVLDREAQASISLFVSACDQGVPTLCSNATVIVDVQDQNDHQPVFKQPGYQFTIPENQFVNSVVGFTKATDDDAGLNAQLTYAIREADSNGASEFFHIVAQTGKILTTQVIDFEEHKEFQFTVTVTDHGIAPQTSEVLVSITVRDENDNAPEVISPNSANDTFFIPQSAGAGFLVVPIEASDQDQGVNSQLSYSISSGDKRGIFGINTAGQLLTAQKIASSWEGVHAVTVQISDGGTPMQTTPLELIIVITDLDFNTSLPAKVFFERFNLTAADFGLADQDDSDQQDSDPLSSWPVITAIVLGSVFILLVLVFLLVYLKCRSKAKPQQVYTVPPDLPSSESHTSADYFSEVHSMTPQRKLSTDTCLSVTTSLGSVQSKNIMKWRAQAVQANGTLDSRLGNLQMTTFGSNSDVMSDTSARRTPETDAEVQRLLNILRMETDAVSDNSRSSYDSGQGDHEEHDSAQNVFPIQHYVRGTPRSASCPKLSGLSGQGQASSRDHYMASPHCTQECATLGHSDQCWNRHERPHERPHRPRSASASIIPQQPIHRPIGLYADRSGGYNIDAHSPGGNTLTKSHNDKTSLSDSRRGSNDNYTNAAARNHSRNDYSPATDGHHSGNYGTVSRGATGKQRANANSSHGSTLPQDHASNKHYRNSNTPLILSPITEDPMEITDPRSAAHNVNSYEDFKNRCGISDQVYPISNVVRHKNGAVYDHYGLGELQSDAADVDFGYQTDVTSGYFPQQSIQTCL

>ArPCDH9

MIFPTTLIWILGCSLVPLVTTQEITYNIPEEEVGYHVGNLVEDLTLPTDSATRFQLLTTASANLTYFELNSVTGDITTIRKIDRETVCPIRSETCQYELEVLVSPQSSFRFINIHVIIEDINDNAPFFSEFVLPIEVPETVAIGTKIPLENAQDPDIGINSVQEYELLNDYGGKFGVYLQRYADNSVLLQLEVTGGLDRETRGTYLLTLLAHDGGNPSLTGSVTLNVTVLDSNDNTPQFQQSSYAVSIEENIAIGTEILKVQAVDPDWGTNGQIQYSFSSSVTQAARQVLEIDGASGSLSVIGILDHEQQASYQLIIQATNRVTNPVPDFTTVTIDIIDMNDNAPSLTVNALGGGTNDQVHVMENTAIGTLIAFIKASDPDSGRSGDVRISLSNTHSDFELQEVSDGQYFIASLRALDRETLRGYNISVRAEDHGSPARSTVQYLLISVDDINDNAPYFSSPIYYTNIQENNIPPTPIVTLTATDPDEGTNSDIIYHLFMEGNDFAVDPSSGVVTSNIVLDREVLSTMNMRISACDNGLPILCRNTTLVIDIDDQNDNAPLIEQSSYQFTVTENELVNSVVGFIKATDSDYGQNSQLAYAIQEADAKEFFRIVEQTGKLLTTRNIDREEYQSFEFTVMVSDHGVTRQVSQVPVRVDVHDANDNAPNVVYPTAADDTFFIPATADPGFLVVSVDVSDPDQGLNSQLSFSMSSGNTDGIFGVNSAGQLITAQTIQTKWEGVHKVTVQISDGGETPQITPLSITVVITNLDFNMSLPANVFFKRFNLTAADFGLIDDTVPHTDNNNPITTWPVITAIVLGSTVLLLLLVIILIHFKCRARPTPKQTYKEPSNLSNSDTVTSVEYYSESHGVQRKLSTDSSQVSITTSLSSIPSKNIMKWRAQAAQNGSIDSRLGNLQMTTFGSNSDVRSDTSARQTPDNDAEVQKLLHVAQLHPIHSETDAVSDNSNRSSYDSGQGDHDYHDPHHDHYTPSDRVRSSSCPRLSNSSQQGGPYNRDHYMASPNCTPECATLGHSDLCWDDASTPRRPRSASANVYSQQPIHKPISMYANRSTGYHGKPHGNNDRLSYDDYDNASPHHNTFPRNTGTNASQVANTHQNGFNTGPSAKQRSNSSHSNAATSDHSNNGKHYRNNAPPLILSPITEDPMELTDSKNSIAANNLNEAYQYPHGNTEQAYPISTLIRHKDGTVYPRYDNYGRPHSYAGHDDTEADYGYRSDVTADMTPEYY

>AjPCDH9

MTMFSWTSGSVFFIIFKLFLCTSKAQHDIVYDLYEEEPDLYIVGNLVENLGLQLGSGAAFSILGDLDSVPFAVNNDGELYTTEVIDREVGKCSEYLDDDCQYNLRILIKDGSNIQVKRVLIRLNDTNDNNPIFFYNQYQIDVSESVAVGTEIPMESAVDEDVGVNDIDTYSLDSTYNGMFGVVVTDTVDGAKTVHLKINQALDRETRDQYNIVLRASDNGTPKREGTTVVVLKIVDSNDNSPQFTEPEYRKSVPEDVPVGYEILNLHAEDTDEGDNAQIEYTFGNSVTSIVKEHFEVDPDDGVIRTIKNLDHERNDVFVMIIEAKDKGQNSISGFATVTIDVTDVNDNAPVLEVSYSADIDREGNTIQILESINVGTFIGLLLASDADSGENGQFDISFDGTYGHFNLIADPSAPKEFAFTAGSGIDREKIPEYNLTIIAQDKGNENNRKTTVLQINIIVNDVNDFTPYFTSPVNRFVMPEHNDPGYLVDTVIASDNDEGLNAEIIYTLEKADGYFQIDPSSGEITAIQSIDRESSASLSFYVSACDKGTPQLCNTTSVIVEILDKNDKQPYFLQQKYSFEIQENKAAQTLIGRVTATDEDVGVNGELRYTLSGSSDNFIINSSTGKIYSKRPFDHEEKKMYRLTVVVSDAGIPAKTNQVIVEIIIKDINDNAPFIAFPTPNNDTFFIPATAPSDFWIINVIAFDIDDGRNGLVEYSIKDGNKNDAFKILSNGTVMTKRDLIKDWQGVYDLKINVTDKALQKKSNVTDLTIVIANKEFSEPLRLSEFYRRFNLSVQYYRDLSRSTNDEANWPYIVVIALGGVAFILFIIFCVLLARCHRKDKMEHTYKVPKEEELFQAGKVSVDGKKDPNNQLVSPAHETNPAPYAQSKNIMKWKMQSPDARLGNKQMMTFSSNSDLNTGSNPSLASTRNTHEADAEVQRLLYKLKINDTDSMSSNSEMDPSSDSGHGDSEHDAYSRHDVGRNHPRNALKNRSTYMASPHCTPDCKVYGHSDLCWMPDPNQDPILLTKQRQNATNYHGGNTRPSPITNSYLSTFDPGENVHKAASCPQIVTATGLAAVSGTRSIEDPDHGRVKSPGAQPLTPIREDPHENSDLDLKNSGTIDGKVTFHRERGNHAPASKNYDNFPGRSSSCSVLPQTSPSVGLLRNDSGVITSRGNSSDNLACSAINGDPDDICSYSTVPGRHRMDPMYDPEKLTPTSEKFFSEWV

>SkPCDH9

MDKMHSFLRTVAVRLVLFVLICIGKSLTTAWEIEYSIFEEQPPGIPVGNLISDLGIEEAGMLGAYDFRFFQPPNGNGSFLQVGGRSGVLTTTQTVDRERLCANVERCIHEVIITFQHQSVEFVKIRIEVLDLNDHAPTFPQNIIWIDISETTAIGSTFPQGGSLFAVDPDAGNNTVQLYELYNSYSDTFGLNAYYSIVEEAIVAEIVVLTTLDREETSFYELVLVAKDFGNPIRSGSALINVSLIDENDHIPDFQQNTYHVEVSENMADVVEFLVVHAVDLDDGVNGQVVYSFSPRTPPYIQELFAIDSVSGAMKTLQSLDYEAQQHFQIIIQATDLGPSPTPTYATVIIDVADLNDNFPVISLSSTSAVGTNQALVSEAVSPNYEVAYVTISDADSGLNGQMTLQLINSNNDFVLKEIYTNELYILTVGQHSLDREIIAEYNLTLQATDAGDDPKSSRAYFFVKLADINDNFPVFSKRVYTAQIPENNIVGAYITKVTALDPDLDANGQVEYVLWNYHHEFDINIITGEITAKKLLDYEERSFYNINVTACDHGTVQQCSDATVRLFILDQNDHAPFFSQNSYTFSVSENLPPDTSIGKIRASDLDTGENSRLIYSLLGEHSQQFRINHRTGDVFTQVSFNYESETQCDLVVMVTDHGTPARTDSATVHVAIIDQNDNKPVIIYPQGYNIIYVPLSAKPGLAVMTIQAADADDGQNGELRYYITDGNIFDLFQISETTGEIYTAKNLEENLVGLHKITVKVEDQGVDKQSVSLRLNVIISDLPFNHSLVNYTNIGNLTEVLPPAKIQTFFLFTVSGIAILVLGSLILVLVIVVVIIIVRCRTNEKAVRTYNFRREESLFNTSKSTSRTATPQNVANTVDAYLAMQSPDVTCVTPGLMHAGSFDKHSGMGSSVMIQNMYNDNSESIRSSHSGIMPMSDTDAEVDQLLSFPTKHQQDQQDEIASYDSGRGESDRDAREHNSANGLASNSQKDHKETDDALEDTSYMASPRCTVECKTLGHSDQCWMPPQEPNPRQHRIPVDIPDNYLASYNCDDRGMASPTYSDSDSSPDYELENGVQWPIASFTSFGYDPPKPAASNTNFDRQGSARLRHHLFSEPLTPITEHPAESLTELLEGAQELERQKKNDLHSQMSLGDHLSSRSSSTSSGNSYVNRNPPNDSAMNRAAHRQSPKSFSDYSCTMSESSLSEGEFEIDSYPRKNVVNLLDNQRETAITDDGEHCDAAQLVKHIDSLFFSDSTV

>BfPCDH9

MAQVRTLSMRFLLLSVLTVCNVCGTEVRYSVLEEEPPNTYIGNLARDLGVVGAATPRTFAILSNSSQSRV

TVDEVTGVLSTDGRIDRDVLCPRAQVCEVGLEVAMLPKQFFQLIQVRITVEDQNDNTPRFRSSVVTLDLS

EATAVNTRVPLDSATDLDSERFSVQTYTLQTSADHAPFDINIFDGVDGSKNVELVVAQALDREARSFYRL

VLTAFDGGNPRRYDSQVLNVNILDTNDNSPVFEEESYSVSIMENSPVGTLVLDLNATDPDEGTNGEVVYS

FSNSMSDSLLSLFALDPITGRITINAPLDRESAAGKVYQLTVQARDRGVNSAPTFATVLINVGDTNDNAP

LVTLNFVTSSEGPVYLSEDVPVETFLAYLTVSDRDGGRNGRVNVTLDGANKHFKLQSVPQGDGQFLIVTS

SKLDRETLPTYNVTVIAKDAGKPPKVSYKSFDIILMDVNDNSPDFGTDRLEFELAEEGEAGTYVGTATAT

DPDQGANGEVVYSTSDGDQTVYVDPENGNIIVNEVIDRETREFIQFTMVARDGGVPSRMGSVLVTIRITD

KNDNAPAFTESTYPFYITESVAVGTSVGTVVSTDADIGRNAKRRYRIIQGNEEGKFSINPENGVIYTADQ

LDYEQVRQYELIVEVRDSGVPQMSDTCLVRVFVQDENDNRPEITFPNNRKNVVYVPLTAPVGYLATSIQA

VDWDEGPNGELSYSITNGNRLGIFVISELGDIRTVKLIKPDWAGLYTLTVMVTDHGATPSSATTLLNIFL

SDAVYNGSTMIGGDLLPANVTDWNRFNIANTRQKDDGFSIPIIIVIALSCALVLLVSIVVVVILKCRRRN

KEERTYNNAEKQPVPQQRNSTNRGSGRKRNTSHREIVVTVNEAAHKMDYNDIIKEEPDGQESSSPGSLTE

TSPPDSESKLLKKDPPKVKNLAKKEQPVRAESETDESGSGPERKERQEAGSSEDEEARLLRMLKDGHTSD

ESLNSTHSNHDSGRGSVHSNHDSGRGDSDKEVRSSPTADLRPQSCEGASNKTELPVETGMPPPPCNVLSR

CTQECRILGHSDRCWMPVPPNNHIPNINGSNNNSPSHHSRLNSPVRNSPEKAKILKIKDFNNPSTGNGRK

VTFVDQRPDSPVSYDGSHHSSPTNSNKGLNLKNSPSHVASASYRNSPTKTGKSLYEDTNRNAAYGSPNRN

TFVDNNVTHRTPYEDSNKNPYSPHKADPRFTGYEAVKPNRKPLCLRTFDPNREFKGTTTLGDSSTSSEEG

NELYNMEDVSEVLDNVEGTPPDSPYGDSSPYKPKELARELDELTFSSFRV

>MmPCDH9

MDLRDFYLLAALIACLRLDSAIAQELIYTIREELPENVPIGNIPKDLNISHINAATGTSASLVYRLVSKAGDAPLVKVSSSTGEIFTTSNRIDREKLCAGASYAEENECFFELEVVILPNDFFRLIKIKIIVKDTNDNAPMFPSPVINISIPENTLINSRFPIPSATDPDTGFNGVQHYELLNGQSVFGLDIVETPEGEKWPQLIVQQNLDREQKDTYVMKIKVEDGGTPQKSSTAILQVTVSDVNDNRPVFKEGQVEVHIPENAPVGTSVIQLHATDADIGSNAEIRYIFGAQVAPATKRLFALNNTTGLITVQRSLDREETAIHKVTVLASDGSSTPARATVTINVTDVNDNPPNIDLRYIISPINGTVYLSEKDPVNTKIALITVSDKDTDVNGKVICFIEREVPFHLKAVYDNQYLLETSSLLDYEGTKEFSFKIVASDSGKPSLNQTALVRVKLEDENDNPPIFNQPVIELSVSENNRRGLYLTTISATDEDSGKNADIVYQLGPNASFFDLDRKTGVLTASRVFNREEQERFIFTVTARDNGTPPLQSQAAVIVTVLDENDNSPKFTHNHFQFFVSENLPKYSTVGVITVTDEDAGENKAVTLSILNDNENFVLDPYSGVIKSNVSFDREQQSSYTFDVKATDGGQPPRSSTAKVTINVMDVNDNSPVVISPPSNTSFKLVPLSAIPGSVVAEVFAVDIDTGMNAELKYTIVSGNNKGLFRIDPVTGNITLEEKPAPTDVGLHRLVVNISDLGYPKALHTLVLVFLYVNDTAGNTSYIYDLIRRTMETPLDRNIGDSGQPYQNEDYLTIMIAIVAGAMVVIVVIFVTVLVRCRHASRFKAAQRSKQGAEWMSPNQENKQNKKKKRKKRKSPKSSLLNFVTIEESKPDDAVHEPINGTISLPAELEEQGIGRFDWGPAPPTTFKPNSPDLAKHYKSASPQPAFHLKPDTPVSVKKHHVIQELPLDNTFVGGCDTLSKRSSTSSDHFSASECSSQGGFKTKGPLHTRQPQDEFYDQASPDKRTEADGNSDPNSDGPLGPRGLAEATEMCTQECLVLGHSDNCWMPPGLGPYQHPKSPLSTFAPQKEWIKKDKLVNGHTLTRAWKEDTNRNQFNDRKQYGSNEGHFNNGGHMADIPLANLKSYKQAGGTIESPKEHQL

>CgPCDH9

MRMYSVFLCVSIFASCIVGEDINVVYSIDEETPNGTYIGNVAMDSNLRSQMTESDFKTLKFSLLSGESYVSMFTIDETSGNLYTARVVDREKLCPFTAVCKISFEASAKSTIRSFFTKIKLVIYIQDINDHSPVFPRSSMSLEIPESTLIGNSFTIDGAKDADTSPNYTLNSYTLEQSDSPFSLSFVQNLDGTSVVKLEVDKELDREVQDSYNLVVIAADGGNPPRSERLLVNVRITDVNDNAPMLSSTIYNVSVKEDEPVGSVILTLKATDLDSGKNSEIRYRLSNHQSENIKQLFEIDEITGQLRIAKPLPYNDGNPYRVIVEASDLGDQPLTVQASVKVLVEDSANNPPEIHVTLLPTSDIARISEDASVGAVIAHVVVIDRDSGMNGYVTCMLDYPYFKIDRLEVNEYKVTVAQPLDREQLPNHTITIVCRDAGTPSLTSTHTFSAIILDSNDNAPKFSSNIYQGSIPENNRKGTHVIQVQATDVDQGQNGEVLYQLLQNNVTQQYGLTIDSSTGKVSTSITFDRETTPTFVFKVIASDKGPTPLSSTATVMVKVIDENDVAPAFLEKVFLFKVSEQKAAGTVVGRLIARDFDEGVNGEFEFYAESGTQPFEVAKDGVVKTTMSLDREKQEVYDFTVFVTDKGQPPKSSSARVKITIDDENDNSPKFVFPSSTNNTVLVSQNTKPGSIIARIRAVDPDKEQNGEITYSIEGANFSEVFYSNNHTGDIIAKKGLEEYSGQTFSLVAVARDLGSAFQATRVEFFVRVAGADYLDSTSQTNITIVIVLVTVTLALSGFIILTICIIRWLDHNRKNQKDIVKVEKTFSSPSDTTEEVSKLSIANRYIEGADDPSRKISNISRYVDHSEIEKRPRNQVTFAPYTKDEETNFSVPNPVYGEPLPSFPSHQIEDNQSNCSMNSTNSDSGRGLSDDETRDGVGRGMLYHSRKGKVRNNIPYQLNIPHQERSTNFFPDNTSPVSPINLTRTESSFGNSFRFHNRPSTSYDIHHTFSFSDKPRLQEQDSDGTSGIHTMEEIPSPFFSGNVV

>SpPCDH15

MATHQRLQGLKLHVLVLLAVLTFIGPTPTQAFECQRSDGLNTDPVIEFEISESNPAGTSIGFLPITGITGSSGSITLVVTQDNPSIFINVLTKQLMLVQTVDADPPNAVKSISLTVQCQTLINFNYDILIKVNDANDNTPFFIGAPYTVSVSEATAINEVIFAGIEADDNDGDNRNGQISFEIVPNQNDPMSNSYFDMPNTGKGEVTLLQSLDFETRQQWTVSIVAKDRGVVPRSSMATLTVTVLNADDQAPMFVGCNTTICTNVEYRASVLEKTPRLGPLQFSPNQIRVVNSSGGTVDPQSVTFSFTGGQPPQYSSFFSINPTTGQVNLLQQVRRSEYDVFSISVAATKGSSSSTATVIIEVTPINNFQPTFPNTTMTGYISENVIAGTRVTADSNGIVPLLIQATDPDLDQDEDPGLSYLLDDNSVFTLDLVGTNGVYLSYSGLSNTPLDAETQASYNVSIIARPTNTSGQFDASQQLPTLQVTILIRDLNDNSPEFQQNQYFDPVTNQYTVNLKDNAAFNTEVISIPVTDADATSLPPIIDHTFVSLPAIALFSVVQQNSDVIIRLISEGSLVIGSEYIIQLRASDSQAPQDLNKQSNVYVRIIIISGVNNRPPMFDQTSYSTSADENLPFNALVDTVRATDPDGEFITYSITSGNINNAFMTVDGANNGEIRVQGSLDREVLASYSLVVEASDGNLTASAVVLITINDVNDEPPVFSGADTFLILESISNELVGQVQATDQDQLNSANSAIIYSFLPEVPEFRIEPTTGRIFTTVGLDREQQDIYTLTVMARDTTTNPLSATLDIVIVVQDTNDNRPVFESAEYIIEVPENTPLQGFFAVQALDRDANAPLRYAITAGETNIFSIDPLTGNLSLLVPLDYENIGARSFYFEVTVTDVDGVNLTDVANITIVVTDVNDNIPSFEKTQYDVIALREYQIGDIVLANITVTDGDPAGSPNSELTFRFLPPSDLFEFTDPTQGNVYVIGDISNTTMWHNLTIVVSDGGDPANIAMVPLSILLRVEKPIFPENVIIVDDVMEEEEPGVVVAVIEAMANIGDVILYTIVNSTDPSAFTMNTTDNVATIYTNMTLDRELVENYSISVQANIQGATPDENNLPEQTGRRKRRQADLFSEVAEPTDPNVVYIYVNVADINDNGPYFLKENYYAGVSTVARVGTQVIAVEAFDDDEGENGVSTYSIVAVVQGEPLFRIDRATGIFSTTQSLEGEEAGSQFEYTVLAEDPNNNFTIAETMVKISLIDNSHRAILAGNIDPDLMRENQDALSEILSEALTADVYIEDVSQREVGNGLDPTGSDVLFYALDANGDPILGNDMVEILLSQNTSLNERYSTIVENKTITDIRRPTQPTTGPGVSPDGNGPTVEAIALICLACVLFICVVIAIGVVIISWKKREMEKDKAGRMYLPSMYNTFNPYGNGEDLEMAQCPDKAFLPSTSNPVYYEDQSNQA

>LvPCDH15

MTTQRRIQGLNMHILVLLAVLSFIGPTPTQAIDRQKRQSLPGPDTDCWRNDDISDTVIDLTVRESAIPGTVIGVLPITGTTGSSGTITLTVVQDNDRVSVNVLTKQIILIQSVDADVDNVNEKFITLQIRCQTTSSSLSPTWIVNIIVDDVNDNTPFFIGAPYTVNVPEGMYNNEVIFNGIEGDDNDGNTVNGQIEFDVLFYPADPLSVLYFDIPNTGKGEVTFDGTLDFEEKQQWVVLIEVRMKDLAAVSFYGVTLMEYTSSILEKTSRATPLEFFPNQIMVVNSSGGTVDPQSVIFSFTGGEPPAYSTFFSINPSTGDVMLVQQVRRTEYAVFVLNVTATKGVSSTTATLRIEIIPINEAPPYFQSQTLIGYISENVVPGTQVTSDRDGTVPLLIQAFDPDLDQDEDSTLNYLLRDNSIFTTNLAPGNGVYLAYSGVPDLDAETQDSHNVSIVARPSNESGQFDPSQQLPTLFVTVLVKDLNEDPPQFHRNQFYNSQTDRYTVNLKDNAVVNTEVISIPVIDPDATSLPPIVDHEFVSSQASSLFSVVTEGNNVVIRLVSAGSLIADREYIIRLHATDRQDANKRSTASVYINIILGVNNRPPEFSQTTYPTSANENLPYNALVATIQASDPDGELITYSITSGNINNAFMTTGGASNGEIRVQGSLDREVLDTYSLIFEASDGNLTASVNVLITINDVNDEPPVFTGQDTFLIVEGVSNELVGQIMATDADQPGTANSAIRFFFVPDVPQFRIEPATGRIFTIMALDREQQDVYMVTVVATDTTSDPLEASIPITIIVQDTNDNRPIFETDEYIIEVPEESLLQGFYAIQALDRDSDAPLLYEITLGDTNIFSIDPVTGNLSLIIPLDFENGARSFFLQVTVTDVDVNGENLTDVTNVTILVTDVNDNVPEFEQPRYDVIALREYQIGDIVLANITVIDEDLENTPNSQLTFRLEPPSQLFEFIDPTQGNLYVTGDISNTSMWHNLTIVVSDNGDPANIATVPLSILLRVEKPIFSQNVIIVDDVMEEEEPGALVAVIEAQANVGDVIVYTIVNSTDPSAFTMNASNNVATIYTAKTLDREQVNNYSIKVQADIQGAEPDENTLPEETNRRRKRQAENSEVAEPSEPNVVYIYVNVADINDNGPYFPKTNYYAGVSTVARVGTQVIQVEAFDDDEGEYAISNHSIVPTEEGKWLFRIDQRTGMIVTNQSLEGEAAGSQFEYVVTADDPSNSFQTNTTMLKISLIDNSHRAILAGDIDPDVMRENQNALSEILSDVLNADVTIEYVSPREVENSLDTTGSDVLFYALDAEGNPIPGKNMVEILRSQNTSLNERYSSIVGNKTITDIRTPTGTSTGPGVFPGSSGPTVEAIALICLACVLFVCVVIAIGVVIISWKKREMEKEKAGRMYLPAMYNTFNPYGNGEDLEMAQCPDKAFLPSTSNPVYYDDQSIQAGDDMFPNGTPSFLASDQLDGQFDGETQERTLEFGPEDEEAQAVADSILAGTSTMPEDNLMRSMTATLPGSADLESSPSYSNQGTLPAYSRTDELPSTFFTPGTGYTNHALSVDDEPDLKRELSSSPYMTKSNPLMGRDEDKSYTQSEASSNPSKSTSPSGSFDPRKPYPSSLADSYTNEVHLLRMGRADSRPGSIDSGHQSLDRRAKTGSASGTPIKVPPPVAPKIATPSAFGNIPPNSDEENVSDEILNGMQDDLSIPPVPASEISSYSYTNSPSSTPPNPKRAAPMLAPHGVNHADIDVSGISGTSGSQENDLSSEHWEASDVMTTIL

>ApPCDH15

MTTTMTINRRDPFMECLLFILLLWGITQQQCEASGSSRAIHRRQAPTDLNGLDCLRNDGGGDIISFSLSEDALPGHVIGVLPLKGEPAGDNATIRLTISPSPQSQFVLLNVSDKALLLAQQVDPDLETQQSRLGLQLQCEVLNFGIPLQFDYRINIDILNVNDNTPVFLGLPYQANISELAQVDTIIFSGIRATDMDGANSNGQVIYSVEYNPTDPDASDYFTIKNGGIGAVSLLRPLDYETKRTWEVLIRARDNGPEPRSNTTVLFLNVLDGEDLPPQFLPCNFTRGSSCEPVTYSTTILEKANMSGPLVFEPGPIRAMDADLDVATNGPIQYTLSDGFPPEYIDYFRIDSETGEVFLLQPVYRADFKEFTLQVTASESPAEGGLYAITSATVTVQEVNEHRPVFAQDYYQGYVYENSRIGTQVSMSNRSTVPLKLHASDMDIAQGRPLMLSYYLSDATAHFILEAGPDGSFAYVVVSSNQLDREVANNYTIRVLAVENDTFALFQSEEAVVDITVLDVNDKPPEFVPNEYSTGENRYRATVEENAEAGTVIMTLSTTDMDLPPYEPVEFVINYIDNDGETKFGLAQSMPSSVNIILEENGTILEGEEYSISMQAIDGGISSLMSEFAYIEITVLPANVSHAPTFEQPVYEASASEGLPEGAFVTTVTATDRDEGDTVYYNITAGNIDNVFTVDSGSGEVKLVGTLNREAVASYELTIQASDGALSSTCILQITILDVNDNNPVFNASLPAEFLVLEGLADEFVGQVMASDMDEPDTPNSNVQYSLVSDAFRIDPDNGMIFTVGALDREQQDRYVLDVVATDQAASPRSTTLQVVVVVEDVNDNSPVFEPKEYSVNVMENLLAQDFLVLQALDRDAAAVLEYLITSGDTQTFTIVPETGSLSLLKTLDYEAMQTYTLEVTARDTENASAANATATVTVNVLDENDHDPVFTMTSYEGAVLRTADLGTVAASGIRAVDGDLADTANGQVFYNLMPASVYFTILDPSEGVVVTKTSLQNAPDVNNLTVVAYDNGNPSRSQTASLIIRVRMERPVFPQSVYMVDDLKEEEPPGTKVIELRASANQGDQIEYEIIAGDPDGKFELVSQENIGTIQTLKVLDRENVSSYSLMVRASVVEATTGGGNRRKRRQADTNVVEVVIDLVDINDNVPAFEKTAYFVGVSDSAGIGTTVITMKAIDQDGGNNSVIDYSMQAKPASSDNQVNKPAADSFTIDASTGVIKTAVAELAAEPRTFTYSVFGRERLGPPENAANTTVTISLVNEDNRVILATSLPPSLLRENQALLEAALEELLGAEVVIEDIGVRLYGDNLEKSDPSGSDVQFYAINVNTGEPFTTDELIQLLQNVSQLDAVLRDISPDGNGRVIEVRKPRTGQRPVTSDAEPTVEAIALLCLAVALFILCILAIAVVVVSWKKREMEREKQARLYLPVYNTFNPYSTAEDHEIPGTSVPDGRIFTTPSAANPVYFDDHAVQVNPRSACDSHSSLLIGAGQDGDERGRLLFEEGAPSFLKPRETQEHTMDFDDEMSSEDLTDEATAAANSIAAAVRSGSSRKSSKRSKRSSRSSSRSKTPSSSGPPSYSSSGTINKGNTSSPPSKIHEVSTIPYRNSETPREPLRPPPLFDPRPSPTSDPRSPFESGGYDNLGADVEDGFGPVGSPISGARTNSSLSTHKPGSLEGRQRNQGVPLKTSYFSHYKQASKNDPATANAQRSEPWEPSDQKLSHVALEDNYSVKLVHMENKPSMRSHPSTPSRDGRKSEMPSRRGSRRKHKRAAQPHSHHEDRRGSTSSDDGGNGHLSHRGGQTNSWSRSKPARSSSRSRKLKRRTSGSDSNFSFGVADGKKGLHQGSKDNDKNGVDGNNPDILQPIYQYTQPPWNELDIINTVL

>PmPCDH15

MMAVYRRDVIAEFLLCVVVLLWQITQHHCGASGSSHNIYRRQAVDFDDLDCLRNDNTTSNIISFSIREDALPWDRIGVLPLKGQPAGENRTISLTITQSEQSQFVFLNVSEKTLLLAQPVDPDMENQPTRLNLELTCNALNLPFPFHSDYRISINILNINDNWPMFVGLPYQTNLSELTLVDSIIFNGISAVDMDGLNSNGQVIYSVEYNPADPDASSYFTIKNGGRGEVWLLQPLDYETKRTWDVLISARDNGEQPLSNTTILRINVIDGEDLPPQFLPCNFTGGNCEPVTYSTTILEKTNVTGPLVFEPGPISAIDADLDVSTDGPIHYSLSAGFPPEYINYFRMDPDTGEVYLLQPVNRADFKEFTLGVMATESASEGGLHAITSAKVTVQEVNEHRPVFTQDSYQGYVYENSRVGTQVSMSKDSMVPLKLHATDMDVEPGRPLMLSYFLSDATAHFILEAGPDGSFAYIVVSSNQLNREVVNNYTIRALAVENDTFDMLQSQDAVISITVLDVNDNAPEFVPNRYSSGDNRYSATVEEDAEAGTIIMTLSTTDMDSPPHEPVEFVIKYISNDGRTKFGQAQATNSSVHIILEENGTILEGEEYSISMEAVDGGQSPQPLVSEVVYIEITVVPANVSHAPSFEQSVYEASASEGLPVGAFVTTVVATDQDEEDTVYYNITAGNIDNVFTVDSTSGEVKVVGTLNREDVPSYELTIQASDGSLSSTCTLLITILDVNDNNPVFNSSLPTNFLVLEGLRDEFVGQVMAYDIDEPDTPNSEIQYSLVSDTFRIDADNGSIYTIVALDREQKDRYELEVIATDQAASPRSTALQVVVVVQDVNDNGPVFERSQYSTDVVENYLAEDFLILQALDRDETAVLEYLITSGDTQIFSIVPETGNMSLLKELDYEKMQSYTLEVTVRDTENDEATNATTTVTVNVLDENDHDPVFAMNSYKGAVLRTADLGTIAASGIRAIDGDFPNTTNSQVFYSLTPASVYFTIPDPSEGTVVTKTSLQNAPDEHNLTIVAYDNGNPSRSRTASLVILVRMERPVFPQSVYMVDDLKEEEPPGTNVVELQASANQGDQIEYDIIAGDPDGKFELVSLNNVGTIKTLQVLDRENISSYSLMVQANVVEATTSGGNGRKRRQTDSNVVEVVIDLVDINDNVPTFGKTAYFIGVSDSAGIGTRVITMTAIDLDSNNNSLIDYSMTARPTSNSGNQIDKPAVDSFSIDTSTGVIKTAIAELAAEPRIFTYTVRGQERFGPPQNVANTSVMISLINEDNRAVLVTNISPSLLGDNQALLEAALEELLGAEVVIEDVGVRLYGDDLEKSDPSGSDVQFYAINTETGEPFTTDELLELLQDVSKLDAVLQGISPDGRVIEVRKPRTGRRPVTSDAEPTVEAIALLCLAVALFILCILAIAVVVVSWKKREMEREKQARLYLPMYNTFNPYSTAEDHEIPGPSVPDGRIFTTPSAANPVYFDDHAIQVNPRSACDSDTSPIVGAGLDDSDGRLLFEEGAPSFLKPRETQEHTIDFENELSSDDLADEATAAANSIAAAVRNGGSQKSSQRSRNRSSRSGSHSKTPSSSGPPSYSSSGTITKDETSPNESSKIHQVSTIPYRHGEATPREPIRPPPLFDPRPTPTSDPRSPFESGSYDNLGADLGDGFSPTGSPVSAARTDSSLSTHKDDLAGPSGSRSTPWEPSNQKHSHVALDDYQVKLVHMENRQSSQKQPKTSSSKETTTKSNAPTRKGSRKKHKRAGRPHRHRENRSSSTSSDEGMNRALSDSSGKMHSSSGRPKGSSSRSRKRRASDQDSNISFGVAEGKKYPSDSRDHNENEMAQTNPDLLKPIYQYTQPPWNERDIMNTVL

>ArPCDH15

MMMAYKSRIDRLQCLLCVVVAIILLNISQHQCEGSSHERARYRRQTEITVCTRNDNDPSNIVSFSVREDALPGDLIGFLPIIGNPMDPNATIHLSVIDSAQSNIVLLNTSEKSLLLAQRVNPDLPDRQISLQLQVKCDLLRLGLSFSYDINVEIININDNSPEFVNLPYIANISELTATDTVIYSGIQAVDLDGSNSNGQVIYSVEFNPQDPEASNYFTIKNGGRGEVWLQRSLDYEAKRIWEVLIVAKDNGDEQQQSNTTVLTVNVLDGDDMPPQFLPCNFTIGEPCPPITYSATILEKQTLSGAITFEPGPIRAVDPDTDVTMNQPITYSISTGYPPEYGEYFNINAATGDMFLLQPVYRADFKEFTLEVMASESLSMESNFYVITSATITIQEVNEHRPVFTQDIYQGFVYENAQIGTQVSMNEFTTEPLKLHALDLDVEQGRPLMLSYFLSDATAHFLLEAGPDGSFAYIVINSNQLDREMVNNYTLRALAIENSTFDMLESESATINIKVLDVNDNPPQFVINEYSQGFNHFRGTVEENAEVGTVIMSLSTEDVDLPPRGPVIFDLEYYNPGSNTLFGQIRVNDTSVNFVLLQSGGIVEGQQYSFYMKATDGNLDTLVSERIYIEVIVLPANISHPPVFINSIYSASASENLPSGASVITVTALDEDGDTLIYRITGGNVNGAFTVDPLSGNVMVIGILDREVLARYELTIEATDGLMVGNCILMINILDVNDNNPVFINQQTEFLVVEGVAGKMVGKVEATDMDEPNTPNSRVRYSLVSDAFSIDSFNGGIITVVALDRETQDRYELDIVASDEAASPRTTTLRITVIVQDVNDNGPMFVPVEYTVDVIENDIVKGFLVLRALDIDETAVLEYTIISGDVQTFSIDPNNGSLSLLKVLDFETIQTHMLQVTVHDTGSIELLNATATVTVNVLDVNDHAPVFTMNSYQGAVLRSADLETVAASGIRATDGDLSSTPNGQVHYAFSPASVYFYIPDKTEGVFVTKTSLQNAPEIHNLTVIAYDDGNPSSSAMASLIILVRMEKPVFPQSVYTVNDLKEEEPVGTKVIELQASANQGDEIEYQIIAGDEDDKFELVSLNNMGTVTTKAVLDRENVSTYSLMVQASVIEATTGGGNGRRRRQATDPSVVEIYIELVDINDNQPTFPESAYFTGVSESASVGTAVITMTAIDLDSNNNSLISYSMSMMETSAAAANDASKPTPDSFSMGESTGIIRTLAEFAAEPRIFKYTVTGRERFGPPDKNANTTITISLINEDNRAIIAGNVPPTLLRNDQDLVEAALEDILDAEVVIEDVGVRNYGTSLEKSDPSGSDAQFYVIDRSTGEPLTNDRIIELLKDLTKLDEVYKDISTDGNGRVIEVRKPTTGRQAINTDAEPAVEAVALLCLAVALFICCILAIAVVIVSWKKREMEREKQARLYLPMYNTFNPYNTAEDHEIAGCSGGSRIVTPRAAANPVYFDDHAIQVYPLDSSVEDDDTRLLFEDGAPSFLKPREAQEHTMDFDNEISSDDPEATAAANSIAAAVAGGSSRRSSQKSRSSRSGSRRTPSGSGPPSYTSNGKITQNGTSTSPSSKIHEVSTIPYRHSETPKEPLRPPPLFDPRPTPTSDPRSPFESGSYDNYGADLGDGFSRTSGSPISGARTDSSLSTQKLESLEGLERDHDAPLMNSYLSQFMPSSKNAPADSSVPQNAAWAPSNEKLAHVALEDDAGIKLLVHSKTHNITPDNRNTRKASHKKHKRSQQHPRRPSLGDGRKYSTSSEEASNKSSADSGSGARPKRSSSQSRKSKLRPSKADENFSPAGGDDKKWRRNSDDINNDPDLLKPIYQYTQAPWNEADIMNTVL

>AjPCDH15

MTAKTSLTEEQSEELTKECSIDTIEFNIQESHPIDVPVATLGFAGTVEEPNPTIILSINVLNLPGALRLVGRDLYLVYSLVDTSVPALIVSVICKLYIDPTSSTVANVNIIVKDENLNAPEFQNEPYEVSISELTPITTAVFDGITAVDSDSGSNGDISYSIIPNPEDPEAQNYFGISVVSTGDIYLKQPLDFETKRVWKVKVEAVDGGDVPQSATAIVTINVLNGDDIPPYFLCYKNDDRCTTVWYYTTVIEETTYNSSLDILPGPVKAVDGDVNITDSDPIVYSIISGYTPGYEDYIAINASTGDVYLLQPVNYESHQFFGLKLKATQGAFDTIALLNVSVTDMNEFPPTLLQTSLVGYVSENSVEGIVVASNEQGTAQLEIYAEDKDVPLNGEGDLVYITNDTSGVFQVIPSFDTLSSKIVVSGHIDREMKDVYQFSLFVRENQTNEEYSSDPILITILVLDINDEAPKFQPNAYSNGLNGYKVSLRENSPPGYIFFQVMAIDPDLGQNGEFEFSIQSVTNNGYSKFSVEQETETAFLKYVGGMLIARETYVVNLAATDKGDRNTQTSVASIEVDILPTNDTHPPEFGRQLYTASVTEEASVGTLVTTLIAVDEDGDTVMYEIISGNEDNAFIIEPLSGKLKVNSGLDRETLDSYLLTVQATDGTLMDTTDVQVIVQDSNDNNPIFNSSHLAFTVPEGISGEIVGQVQAYDLDEPGTINSQVEFTLISDAFTIDPVTGVITTSEALDRETQAQYMITVTAQDKAANPRSTTIIVTITVLDVNDNGPTFEKSTYTVMVMENSPITDVVTVKATDSDIETNIRYMIVSGDTNIFALDSTSGDLSVLAPLDYEEQKMYTLTISATDIDQQDANLVAMTTVVVQVQNQNDNVPQFQKPFYRGSVPEYADLGTVVIDNVIATDADIGEFGQLTYAFYPESDWFIINENKILTKTGLTKTTQDDFNLTIIAEDMGELPLNGTASIFIQIVDRGVTFYEVPTFTEPEYSANITENSEVNTTVATVQALANQGEIVTYEIIDGNENGFFWIRSANNIGTILTRTILDRESTPEFILTIQASVTNSLGGSVDTSGGRKRRAEDPSITIIVITVGDINDNPPIFTQSLYVVGVSERASAGTAVVTVQANDPDTEDNIITYTLQPYLSGSQKGLDVFEIDSRSGRITNLEKFTSNSQTYKFTVLATVETEEFAEASCKVFIAIIAEEHRTVLVLTAAYDDALINEIHNENQLENVLNAEVEIYDIRSNSDPIYTDNYFYAIRRDTGVPLTSEEIQELIDGNTDVLSNQILPEGAYYLVQAEPVIQEDEPWKLEYILIIIFAGLLFMFTFLAIIVVYVSWKKASKDRRQMEREKQARMYVPMYTNLTAINDPNATEMDLIRISELSSDVHLRRSNPVYFDDQSVQASEQLFPNGAPTFLNARSNHVQRSQVEVYDNPACTIENGEVHPPQREETESEQAEPGSGPPSYTSEEQPTQLASPSHHYTSISETPDFIDADTLPKKEPLLYDSPQGDGYSSSPEGHNILSTPPPQSPPSPATPPPMATPVSLQGVYITPAKDVTRYDPPTMSSFQPIDQELPPPPPPPVSPPVSPPPQNNIPPPIDFPPPPEYPPPPENYREDDEESSYGKPSISSCTESEEKHQDNINELPYRMMKPPVPFDQPSEGDVVTPYGSWNLKNLKLNHIERRDQDRTTIIATYKVNRTESAKSNGQQTTDATSVGDSGVNSEHGSPSSHSPQEGNVSSNDVDSVVRRDDWGLDNIQITAL

>SkPCDH15

MKIRFHNPLAAVLAILCICEHLVFSEESDCFSYSDNFLSVDEDTSVGETIAYLNSEGTAGGSNSTILHLGTMDGSDVPCTEYVVLDEEMQAINLTKALDRDEPVGIKSISCKLTCTDRILGFQVDYIINFHVEDVNDNYPYFVGELPYHTAVSELEDVGTIIFRVSAEDPDSGGAVSYYIRPNPDDPESNESFSISLPSIGDIKLTKTLDYESKKEWRILVEAKDRDQVDPKSTTTILTVTVQDGDDQGPQFLPCIRVDNVCAMQTYTTHIQEKQNITGFFEFSPGPIFAIDQDVNISTEIIYSFLQGSPPEYIDYFEIHPHTGNITLKTPVDRGLFQFFSLTIKAQEGTNTARYQTANVDIYIDDVNDNKPVFLETSYSGFVKENSAVSTTVVTTATGTSPLQIIAVDLDIDEGVSPQLEYKFIGADGWFSPSKIGDTLYVVVSGELDREMNTSFFFWVVAMETTTEETMESDPVAITVTLLDENDNYPIFAGGNNYRVMVKEDAIVGSVILSLTASDEDLGDNGIIDFSITHIANGASDLFNINQTNATAVITLAKSELEARKEYTITVMAKDRGPENNQKSTLAFVTVDVIPSLNARKPVFTENPFHASVSESALPGNVFETVTATDADGDTLHYNITGGDPDNEFSINEFSGEVSITTSLNRERQSLYILTVSATDLNQTVTTELIVTVLDINDNNPIFNSTWPSVFSILEGQIGIVGQVQAYDLDEPNTVNSQVEYTLTSDKFNISPQEGVISTIVALDREQQSVYELVVTAQDQADSPRSSTATFTVIVLDENDHGPVFANTYYEYTVPENVLENDFLTVQATDEDESAILNYNITTGDAGLFGINSLTGSLSLLTTLDYETKENYILTISAIDIHQIDETIATADTTVFIHVENTANGAIRYYIVPPNEFFTMLDEQLGVIVTKTNLKKTTLNEYILTVVAYDGGVPPLNDTALVIIQITDREREKPVFEQNIYRVSDLKEEQPAGTPVMEIHATAPSGDQIDYHIVSGSGNESFRIESQDSTGFIYTTEVLDREVTASYILIIEASLQDDMDAESGKRKKRSESNIAEVIIELEDINDNPPMFTQSKYIGGVNEDASPFTDVLKVWVLDADEGPSGEANYSIKRDGISNDEVLQASYFQIDRTTGLIETTVAITEKAGSKYEFTVVAEDNPFGEQRFMAQAEVSISVINKDNKVVLVADVPPTVINDNIDRVLEVLEEILNTTVVLEKVGSRQYGDDYEKTNPSQTDILFYAIDENGKPMSREEIMRILAENEALLNELFSSFLPGKVVTVRPPETAIITRYYSLAHTDAALISLACILFVLSFIAIIVIWCSWKQYVYFSSMVDINMLMTDGKYQATGLVSMVI

>BfPCDH15

MEAEVRLCWILLVVGVVSFVLPTAAQQVSELALDCALRNSQAGPTTFAYVDENSPNGTILVPYMDIVGETSGENATITLSLKDTPDSHWVILDPTNQTLYLNVTGNNTLDRDGTAGNNGITSLRVTVVCVSNTWGTIEHPVTVVVTDLNDNAPQFHPDSYFVEVNELTPVGITILDGFSVTDLDGTNEIKFKIVENEDDPVADEFFTIPLPKTGAIVVSRQLDYDSMAPPRQYRLKVEAWDTSDEQDPSKNTAHTIVTVNITDGDDLGPLFEPCCDPVTYMANVTEKSEPNFLNPIPVSPNNIAAVDQDKNIQPVEERPNIVYSFFSGSPSVYREYLSIDNRTGDIILQRAVNREDYQTFSIVVKAEQDRNDPKPAYANLLITVLDLNDHTPEFDEREYVGFVGENSVAGTTVVTREDGAEPLEIRAMDLDGTEVMYSLLNYTDKFRLRPVGDVQYLIITQSLDREEQDTYELSVMASDGELTNMTTVVVTVVDRNDFTPQFHPPGPYITRLKDDALAGHVVYELNVTDGDGGAYGEVTFSILHVTNNGENKFAVQERNGTVTLEDESLLTGEVYTLTVMAADGAPEDERKSSVTTLEVRVIPANNQSRPTFDPARYYVSISEGAQIGTSLTTVHAFDAEGEPLHFGIADGNPDNTFNISSKVNATEEGQIILNKELDREDIPAYTLIVLVDDGNENGTATATVEVVVTDINDNNPIFNSSFLTEFTIQEEQDPPVLIGRVESTDADIGAKGEVEYHLVSSSFEISPVGAIYATQRLDREEQDQYVLVVTANDKAPDGRSSSVTLTVNVTDINDNSPQFPEDLYEVVLDENENATVLLTLQATDADLDPSLTYSIADGDTSMFTIDSTTGQLSNLQPLDFEMGPEYTLVVQADDSQNQGNTTVIVAVNDMNEFAPVFSQNVYTAEVLDNAVVGTIVTTVNATDNDVKGTPSAQVLYRLADEDSMAAGLFRVDRVSGVVTSRVNLRESPGDTYQLAVEAYDGGDPVMSSQTTVNITVLSSDTRPVFQKNLYEVPSLSENTPVGTDIVNVFAEGAAAYSIERGNAEGIFRIDSGGVVYVNKTLDHEAVTSYRLLIRAYSDQPMRKRRRRATETDDPDYAEILIALQDENDNPPVFTQDKYVTGVTETIGTFASVLKVEATDSDSGNFSTVRYSLRQSGEEDDTGNFAIEGSTGVIKTAIPFTGKMGKVLTVTVEASDGNNVASAQVLVTVMNEDNKVILVGNAPATMVDENRDKLLNLLSNITGGVAVIDNISPSVQAGTVDATKTEVGFYILDKETNQPMSKERILEILASKMDEINKLFGSFFPGDEVLEVRAPEERQVITTSLSFTEGALIALGCLIFLASVVGIIVIIITWRRRTNAMMQKHRLLYIPNYNAYEELDGTNTNGGVPYLRAYESQEVKMEIPEGTDPNEQDVVFQAEDGSFYAIQMRNPVFDKGTLLYSSLGSGSSMLYEADTPTSSGTYSYNIPKIPFRHRRVPMTVAPLISCHHVPMASSESVDTDFESANVPPAYPPAPPPDFFDRDSIIAIDADASSSEEESPVSLFSTSSGPLLTVPQKYHVPIGCVRTPSGSFHSPLSGQTPTSGRTSFSGHPLPSSATYSKFGGLSIPSYPTSGYTSIGGTPIPSIPCSSGTPFSSPARSGISQNAPPPSTTAHLPSRREFRAARGVPVRIVPLTLASPVPRNGRKSSESARSQSSSVGDSSSAESGGDSPRQGRPRRRKSSSKGVVDKM

>MmPCDH15

MFLQFAVWKCLPHGILIASLLVVSWGQYDDDWQYEDCKLARGGPPATIVAIDEESRNGTILVDNMLIKGTAGGPDPTIELSLKDNVDYWVLLDPVKQMLFLNSTGRVLDRDPPMNIHSIVVQVQCVNKKVGTVIYHEVRIVVRDRNDNSPTFKHESYYATVNELTPVGTTIFTGFSGDNGATDIDDGPNGQIEYVIQYNPEDPTSNDTFEIPLMLTGNVVLRKRLNYEDKTRYYVIIQANDRAQNLNERRTTTTTLTVDVLDGDDLGPMFLPCVLVPNTRDCRPLTYQAAIPELRTPEELNPILVTPPIQAIDQDRNIQPPSDRPGILYSILVGTPEDYPRFFHMHPRTAELTLLEPVNRDFHQKFDLVIKAEQDNGHPLPAFASLHIEILDENNQSPYFTMPSYQGYILESAPVGATISESLNLTTPLRIVALDKDIEDTKDPELHLFLNDYTSVFTVTPTGITRYLTLLQPVDREEQQTYTFLITAFDGVQESEPVVVNIRVMDANDNTPTFPEISYDVYVYTDMSPGDSVIQLTAVDADEGSNGEISYEILVGGKGDFVINKTTGLVSIAPGVELIVGQTYALTVQASDNAPPAERRHSICTVYIEVLPPNNQSPPRFPQLMYSLEVSEAMRIGAILLNLQATDREGDPITYAIENGDPQRVFNLSETTGILSLGKALDRESTDRYILIVTASDGRPDGTSTATVNIVVTDVNDNAPVFDPYLPRNLSVVEEEANAFVGQVRATDPDAGINGQVHYSLGNFNNLFRITSNGSIYTAVKLNREARDHYELVVVATDGAVHPRHSTLTLYIKVLDIDDNSPVFTNSTYTVVVEENLPAGTSFLQIEAKDVDLGANVSYRIRSPEVKHLFALHPFTGELSLLRSLDYEAFPDQEASITFLVEAFDIYGTMPPGIATVTVIVKDMNDYPPVFSKRIYKGMVAPDAVKGTPITTVYAEDADPPGMPASRVRYRVDDVQFPYPASIFDVEEDSGRVVTRVNLNEEPTTIFKLVVVAFDDGEPVMSSSATVRILVLHPGEIPRFTQEEYRPPPVSELAARGTVVGVISAAAINQSIVYSIVAGNEEDKFGINNVTGVIYVNSPLDYETRTSYVLRVQADSLEVVLANLRVPSKSNTAKVYIEIQDENDHPPVFQKKFYIGGVSEDARMFASVLRVKATDRDTGNYSAMAYRLIIPPIKEGKEGFVVETYTGLIKTAMLFHNMRRSYFKFQVIATDDYGKGLSGKADVLVSVVNQLDMQVIVSNVPPTLVEKKIEDLTEILDRYVQEQIPGAKVVVESIGARRHGDAYSLEDYSKCDLTVYAIDPQTNRAIDRNELFKFLDGKLLDINKDFQPYYGEGGRILEIRTPEAVTSIKKRGESLGYTEGALLALAFIIILCCIPAILVVLVSYRQRQAECTKTARIQSAMPAAKPAAPVPAAPAPPPPPPPPPPGAHLYEELGESAMYEMPQYGSRRRLLPPAGQEEYGEVIGEAEEEYEEEEWARKRMIKLVVDREYESSSPGEDSAPESQRSRTHKPSGRSNVNGNIYIAQNGSVVRTRRACVADNLKVPSPGLLGRHLKKLDTLAGTREENVPLNTLFKGPFSTEKAKRTPTLVTFAPCPVVAEHSAVKPSGTRLKHTAEQESMVDSRLSRESMEFHGDSAPSDEEELWMGPWNSLHIPMTKL

>DmPCDH15

MAARNSLTPQQGLGFFGLLILLCSAVLGKSQMCEVETGQTNIILDIEESRESFIGQPTTPAELPIFGDPDTEIALNLVFPKGQPIFQLNGKKLQLLQPLDRDEENLSHIVFQVSCTTRSTGKKRTIPIIVRVSDINDNAPRFMNTPYEVTVPESTPVGTTIFRNIQALDKDAGVNGLVEYFIAEGSPNSTNVEKYSADGYGTFAISFPHQGQVTVAKTLDFEKIQTYYLTIVASDRARNTADRLSSTTTLTVNIADSNDLDPSFIYSGCVSLDGACINPEYSASVPAGSLLGVLTVLPERIQAVDLDTINSPIRYSFASGMPGNYADYFQIDESTGVLKQTKAVDTSTAKKYDIIVKAEEVSPGPQRFTTAKLEIFVKPVDANPPVISSSQAEGYVDENSPIGTRVLDAHGNPISFMTTDADLSDSDPKPDYIYELTTPSFNVTGDGILVVNEENLDRDPPAPGRFKFQVVAREPRTNAASAPLSLTVHLRDVNDNAPKLAMVAPISITAGDQSESRLVTQVTATDNDEGPNAVVTYSIYHVSNNGIQKFTINATTGEIRTQGRLLAGEQYSITVQATDIGGLSSQAIVEVSVTPGPNTKPPRFQKPIYEVQVSEGAEINSTVTVVHAEDPENDAVVYSIISGNDLRQFAVGQESGVIIVIRKLDRESLTRYQLILKAEDTGGLSSSATVNIKVTDINDKNPEFEASTLPYVFQVEEGKAQASVGVVHATDADEGINAEITYSIPTDIPFTINATSGEILTAKELDYEQLNEYKFVVTAKDGAPDARLGTASVTVMVLDLPDEVPKFSDARIDVHIPENEENFLVATVQAFDPDSMPEITYVLRKGNAELFKISEKSGEVRTIKGLDYESQKQHQLTIGTIENDGNGPGDTILLVVDVEDRNDLPPRFITVPDPVTVNDDQGIGTIIATLPAIDGDGTSPGNVVRYEIVGRGKAPKYFQVDPDTGAVRIRDELRKEEDTEYQVDIRAYDLGEPQLSSVAPLRVDVHHLLSSGNNEIKLDNKLESGTGMSSESIGLAFSDDSYTTSVPESMEANSTLKLIQIVNSKTSGDGPPAFRCEFVSGNEGGIFNLSSADHGCNLLLIQPLDFENKSSYSLQLRLTSHRYFVNPLKDTTSVEIIVQDENDNAPEFEFNRLRGQQDTFYTVVTEEMDVDTTILQVRATDRDSGKFGTVRYTLYDDDENRVNMPTSFFMMSEDTGVLRTAKHFKNENDFPLTFLVEARDSDGQEQGSHRTRARIVVNKLADINRMALSFPNAAPSDLRNYYTELEELLDKKTGLVSGIERMSSQKYLAKNGSVIENPAATDIWFYLIDPRTEQLVSRKDSIVETTLLEPAARSELNIALPRATAENISFPLERKEHVHKVKAAVAIDNEVFPFTLIAISLVILILGTIGIIYICISWSKYKNFKQRMRQYSSTNPPRYDPVIVNQQASSASETIANMKEYETQMLAMAVPPDVDDDLQLDFSAKNHAFSLDNVSYITHKENTNGGGQSSPSHSDATTATIATLRRHKNLNNASMNNNLAINNRQNTFNRTLEMNTRNNANPLASPPNGALSGTLTLGRIKHQNSNHYQNGAYNIDPTGPNNMAHAKNNAYSTMGRRGNTFGDVGLLNGNGELMNATLGRNGQLNNRLYGGEVPITNPLFQRSNSDHNHLSSTNENVSFGKRDYGQIGFSYLNDLDRSEVETTTEL

>SpUECDH

MARTSRTVPRIGRIGSVVPSALIFWIMVIILGECQPGVHGICSIDCQRNTNYCKECVRDPEQCATVPYYETTELGAILFNVTVDTDQPVPTDITIRISGDVESMAMFRVEGLQVILNKTFDYEMVEDYRFDIDCNDGTGWSTTPESSGEKGNAETGIVLITDVPIQAFDDDAKDYGFEYFILEDVPFNVTGPTPDGCQLGEVFLRLNGTVDFEDQEFYNFTLIAEDGCKSGFNSREGYVTVIVQVIDTDDEPPAFSQDNYDVTIPERNSTFVYTGLSASNGDSFNTPLRYSFIEQRGEWSCLYYLKIDPLTADVTIRNELDFETSPGTCIITVQAVQENDFNMVGTTNLTVTVMNVEECPSFILQSYSGQLTEDDPFVITDGGSERLIMGMTDEDGVFGGPSYIEPNDTIELVEMNMPEGTMTRFFLIKFLDPASIESDTKIQIFLSDDKADMECDSVTNVTVRLMPDGLAPNFTQSYYHGNVLENATEGTSIIQVEAWNANMTSEGIVYRLQMASDGGEDVFSVDAESGDVSVVEDGLDAEKVKQYTLIVEAVDERGTYYRYSITSVIIDVTDVNEHAPTFAEGVVEVDIEEEVPTDSSVYTVQANDLDVDAILEYSIGETAGPFAIDSKTGEISTDGRLDRDEGTTAYNITVVVMDGTNEDDCLVTITLTDINDNAPEFTGGSQALSVSENTTDTEMLQVIEATDIDEGVNAEIVYVLESPDDGLFSVDNETGMLYAHGPFDYEAVKEYTLVISASDSKFLTLTTVTVTITDVNDNAPVFISFCLNIEVLEDVGVDDVICTVGAVDDDGDGEDTVLYSLDPSSGDVPFNVNQLTGEIKTDGTLDRETEDIYIITIIAEDSGEPPLSSNTTITVIVLDVNDNIPMFAKTSITVRLQTDDLADPPVLLSRVKATDDDLAPWNITQCEKTGGEDSFVVLTGQDGSCEIYAYEELPLSTDPYSVSFYAFNPDNRDRPSANVTLTVDIRDTVCDVTFENEADDDVIVLKSELETNVPVVTLNVTSNCNSDDIVFNISSQTYRPDLDASPLPSINFTINDQTGEIFPTDIVMEGKYILLVRAYNQSDPEVSETTEVTIVVTGENTAPAFEEPFYTADITEDSMTMVIDLGPEVTDVDTPSFANGELSFSKVSETDGSGSSSDYFEVSSSGIVTTTQAILNLIKVGTFEVVVMVIDGAIEPLSNTTVVIITVDIDDQSNPVVSPPMITKDCPDQVEIEEETVLPVHICRVFGSSTYDDQVVFGLESGSEYFDIGTDLGNITTSKPFDYEYDDHSYTLLVYAEGTTSKLRNYTSFTVIVTDINDNAPVFSESPYRFSIKENAHGSTECLTFVGLIQSTDADSKINQFVTYTTDDDDFEVRDNDLMAVNCISRNSVKYATGEVRVTITALNNGTNATLTTDVNVTIEVVDINDHAPEFVNDDFSVSLEDLSTVRVIFQGDVNDMDEGPFNNESVFSLADDQDDVIYSLFHMSSDGDLTVQDIGISDEYLIYEVIIMATDMGQPPLSSSRTVTVYIGIVDVTEPYFLPSDRNQTFERKENQDDCLNISYPLICNETTYLEVYYEHVVKPESGTDLFSINETDNALSLCWKELDREVSSSYTVTLTASYVNSSMCNSINVSTSDPTRKRRNVQEVGTNTIILTIIVTDENDSPPSFQFPSNTIGTAYYLAAIDSTQFGSSVYTVIAEDADASWEFNYTLTSLSEDDVEGPENTIAIDPSDGRITTNSLLYPSLSDAWSWEWEKENNFRASRTQDPYNVTVCDTSPTSSDISQCDTTRLTFKILLSFEHIIIRFNKPVATILPQQNDIISCIAENMDPTNMLEINLQFLSPVKEDDQDMTDMWLYAVDLSKLPSSPYLKYEDFEIMWDDTEGLEECNDGTMEPRRLQLSKILPGMSTTSAIEAWVPIALGIIVFIGNLICIILLLVSWKTMHLESGRELARELTVRDEEKGIVNPVFQFDDEADDDVEIMATEAEEGATGSEEPLITTVYLTDGKTQSTIAMTANDEPKDVEKAVGTDAITIPEREYDFQEKEMDFDFEYDDKVDLNTAGMM

>LvUECHD

MFNVTVTSDQPQENVSIRISPNSADKEAQDIFSIEGGQVFYDGGFDFETKSKINFVIDCNDGDGWSSGVDSLSHKVSVRDVNEHAPYFDVINIITATIAENAEIGTLLVTDVNIQAIDKDGSDEELSYNISDDVPFQVTSGCQSGQVFLLLNDEIDYETQMSYNFTLIVKDGTPCSSGFDPKEGNVTIMVLVNDTDDQQPVFSRDNYDVTIPERDSSFVYTDISATGGDTLNTPLQYSFIGQSGDWGCLDYLAIDPDTAEVTLKKELDYETSLEPCIITIQAVQQNNCEMVGQANLSVTVADVNECPSFISDSYSGQLTDADPFVIVDDGSERLIISMTDEDLTFVGDYFIVPDDVVELVMVSTQEGSDYPSFYIKFLDTETITDDTELQIYLSDNTTDVECDDSVTNVTVQLTPDGLAPNFTESRYRGEVAENATDGTEVVQVEATNSDVTSEGIVYRLQMASDGGENVFGVDVDSGVVSVVDGTDLDAETVQQYTLLVEAVDQRGTYYRSSIVSVIIEVIDINEHVPMFPNDDMDVKIEEEVPIGFPIRTLQANDSDVDAILEYSISEPDVPFRIDQKTGEMFTDGELDRDEGPSTYNVTVVVSDGQNVNEYDYILTISLTDINDNAPEFTGGPIEVSVPENTTDPESLKVIEATDIDEGVNAEIVYSLLSPDNDIFSIDNETGMLYANGPFDYETVREYTLVISASDTRFSTLTTVTVRITDVNDNAPVFTSSCSMVEVEEDADVDRVICTVAAEDIDDDGNDAITYSLDQSNGNVPFKIDPTTGEIKIAEELDRESESSYNITILAEDDGDPPLSNSTTVTVVVLDVNDMEPVFEDTSLEVYIQKDELAFPPVLISSVKATDADLHPWNIIQCEKTGGEDSFVVLTGQDGACEVYAYKELDISPDPYLVTFYAFNPDNRDRRSENVTLSVHIRETVCFITFVNEDEYDVTVLKSELDSSVPLNLPLNVTSNCNEDDVMFALLSQRYRKDKLADEFLPAENFTIDPMTGGIFPTDNVMEGEHILLVSAYNMSDSEVNETTEVRIVVTGENTPPEFTKEKYFASITTDSESMIVVIELDGEASDDDYPSFGNGELRFEKGNETSEDFETDYFAVSPTGQVTTKSDKLNEMNNRIFELVVKVIDGGKEPLSDTAVVVIDVDIDESNPIVSPPMITTDCSNRVEIEEEMPHPVAICNVSGSSDYDGEVIFGLHSSSAGYFNIRHDSGAITTKHTFDYENDDHSYTVLVYVEGTTSKLRNYTSIDVTITDINDNAPDFSEGELGFSMDEDDHGAENCTTRVGLIQTKDIDSEENQKVTYETDHVDFEVQENILTAVTCISRNDVEYSTGEVEIKITAVNAGTTRNQTSNAIITIMIEDLNDYAPVFKNDAGDFRVTLVDLSEIQVVFQSEVVDEDKGPYNNQTMFSLADDQDDLIYSLFQMSSDGNLTTRNSSAIKDDYQQELYEVQIVAKDMGQPPLSSSKIVKVYVGNFDLTEPYFPTEDRIYPIEIEENSEESCHDFPIPLTCNDTDVVVIYELDAENDVGEEIDESLFFYVDDENNSLCIKELDREKSSLYTVTLTASYDSNACNTSSSVTRKRRAAEVPSNTIVFNITVTDTLDSPPSIEFPGFSMRSSFYLAAIDNLQIGSPIYTVSATDDDATWNFEFTLINIDDGDDGRPKNTITINPSTGQIVTNGPLFPWIGDTDNWEWKGANFPAERTQDRYNLTVCDISPNITDAQCSNTTQLTFKILLSFEHIIIRFNKPVASIQPQQNDILRCIMEKMNPEKELEITLQFLSVGMENGISHPDEKVTDMWVYGVDLSRLPESPYLKFEEFEELWVNTDGLDECVDDAEMTIARLQLSKILPEVARTSAIEAWVPIALGIIIFIGNLICIILLIISWRNMQIESGKEIARELTIRDEDRLEKGITNPAYDFHEINGDVEIMASEAVESATGSEEPLITKVYLTDGKTQSTIAMTATDDPKDVEKAVGTDDDILLLPVPPPNRDYDSDEEMMNVYPFDEPDEGYEFKVDIGEAPASPTIESPPENPVLIPLADYDSEDTKDKDGTKDELKMEDQEESLGSKPKPPPVAPKPTDQTPKEPNAEIDSLMAEDRGHHDDEANTKTAQNEDTPDSAKSLNDPSSLIEPFPPVDYQTETTNTEDKEQSPAVEHDNTNIPPPPPPPPMPLVQEVPLVDDQVQDHSLPPPPPPIVQPIVPPIDYADGKTVTFGGETVVGEGAGDGGSTTDLETASEASSRSATTEESSPKLQPSRSRPPFASEIELVAASFNGAVYPRSRKENPELLASPQSTCESDGESIGYLEDESLISVSEIGTSGIGVTQL

>ApUECDH

MNILLITTLTLATCLHLANAECKFLCNGQDCRPNTNVNVDEESSGPQDVYQVFVTNESAPIVEYTNCTGAHCNKFEFDDADKKVKMIDNSGSLPDFDAQSGSPIPELVATFSLKCEDATLDVSIRTQNINDNLAKFENAEYSATIPELTKTGVVVPLDDIILTDEDRSEENLNINLQDLNNDEIPFQFEADFGSLISTDKVTIRIKVKNPPAVDFERHPGYILTLSVNDTQVGPYQNSVTTATLNITIEDLDDKDPRFLNDSYMATTSEGYTGPLEVQPPIEAKDQDSLNIPVKYSIAGAAPCSSGSFMIDENQGNISVINAVLDAGECAIQLRAEQADDDSRFATAILVLTINDVDSNCPYFVDKSITGRIDPDDRDAYVIDGVDLLSLEAKDDDKEFVGNITLSEENHPFQLVISDETSNPRIVRFKVKETLSLEVNQTYSLQVEAQSESCFDAASVSITVANLDPVYNKTRYDTTIEEGRVYSDSILQVTAESSAPPGEITYVLKSATDGGDSYFSVDATSGEIKVPEKTDAEEVERFILFIEAQDNMWQPARISFANVYVTVKDVNDNVPVFEDTAYDKSIPENYEVGDLVLTVRARDADRDDVLEYMIDVPDLPFVIRSETGEIILNKTLDVDAPNSQAEYMFAVIVSDGNEDHDQSVNVTISVQDENDNSPVFDGEYSWDLTEEELINYEVFQVFATDADVDTELTYSLEYTPNNKYTFEILNDQVGNITVNGTFDREEQSEVEVTVRASDGLNIPVKKVKFIILDVNDQRPAFDSRLSCSSSSPFEISETAEKDTPVCQFRATDDDKPNTINSEITYSLLGETDFGIEGDGEDYRLIVNGTLDRETKELYDITIMASDSGTPQLNSTLELKIILTDVNDNAPAFDQSSRDIVLEEEDSYAAGSLVVGLTVTDQDKNSFFVYEAVGDNVDSFFVRPTDNGCEVFVRKTLIDSGQPYIILITVSNPDKPELVDDRSTIRLRIFPGKVQCLQFIPQNPSLTLSAPLDNSTIIDMDVNYTCTDNLDTSYIEFEEQTETYRADQNDVGGQTDFFSIDEFTGEINGNSAAPVGLYSITLEMFNSSDVNNSIRANTKVEVRLVPIDNAAPQFSAEVFVLPPLILPLDMDRELFDLTPNVTDDDYPQFGNGELMFDKLKEECLDCTETDVIQVTPNGKLFLAVESTTITALSLNVTVRVQDRALVPLNDTALVSISLKQPSTSPPTFTKECTAPVRIEELPVIYGFRRQEPGNFSLNSETGQIYTTRPFDYDTEDRMFELIVFASTSETSPERITVVTVEVVDINDHAPTFTQEDYTFEFDEDTNDALCSQHKWSIKATDDDSTPVNSIIQYSIVGDSVDFDIDPSLGIITKIRCIDAENANYTSGLITLTINATNPDAERQLMSSVEVTFIIRDMNDNSPTFDPVDMPPERFPYLPSFSEIVNVTATDKDVSEFNSGIEYSLDTPEQAASVFQFFTIDPTSGVISTLNVTDGVIGETADLVIRVNARDKGAPPRSAPEPARVVIIVNPTYKDEPYFREDHRQQEISIPENAETCSNPFTAPYLPNNKELRVLYTIDQVVPPSAIDQFCLNFTDLMTPSLCTKQMLDRENSTEYTITVRASSCNTSVSNCEPGNQCGVKRQAAIRALNGPDFSFVVIRLLVDDINDANPEIEKDFVAGANKFLIAVPDDTKYGAKIFQVQATDEDATSDLAYTLETEDKTLPAFSFDQSNGILQVRDIVSNYLDDASVDPAKEQASYNVTFKVTDTLTDDPSKASAEVQAKIKVLYIFEHAVLVINKTPTEVETEQSTYLDCLSKSLGITLVSERLEAHLVTDVNGVSTIDDKRTDLWVYAIDVSKDGGSFIKYWDIKTKWDSSSSAISNCGWEIIIQPVQPPAVAKHMYDVSMILGIALLVLAGLIGVASILMIVAVLYMWKDVKLQGGRQISRKLDMLMLEKDQLGFENQGFQDDESIEEASVAHASEIEEEAQVCLVETAVGADEELPRYIEVPVVAAQISPLQKGPEYYESQELVLDFLEYEEDEDLGVPNFAHIHTKEYNEEQFPNLVPASIEVVNEQQGHASDKPDDQDNDKLEVISDHKQDDEPEDNREDKPDDVSSDESDGNSDEVSDGTVEGKINEETDDKQDDKLDDKPDDKLDDKPDDKLDDEPDDKLDDKPDDKLDDKPDDKPDDKPDDKPDEKPDDKTDDKPDDKTDDKPDDKPNDKPDDEPDDKPDDEPDEMPDDKPDDQQPEVATKEDEDSPSEGQSSDTFPPPPEEREGNQGHVTQATPKSVHFDLPDQGSDRSSSSASSDSTPLMDFGIGMVPDYLEELEVTQL

>ArUECDH

MPDMEDFPSGKNKLPFGVIEFSCTPAGGTEQKLTLIVSTLNTNDNKATFVGKTEYEATIPELARPGVLLDFGDIILMKDMDRLRKELTYNLITEEDVPFEVSHLDDSFTADGRIEFEVKVKNPPAVDFERIRQYNLTLAVTDAQDGVFKDHISSVTLIITVEDLDDKDPVFTHLNYAATIEEGTVGDLTVMPADICAYDQDLGLNVAVTYSITDIVGDCPADSLEIDAYTGVISVNDTRLKAGQCIIELTARQVDDDQRSARATLTLTISDIDTNCPGFAVTELTGRVDPEDAFVYEADGVDKLLLEVTDDDATFSGNLTLHDLDEHPFRLATEGTEQGAPTLYFNVTGSPVLTINQTYNLQVTVSASDNCTVERASVKITVQNLAPRFNQERYDTEITEGYVSIDPIETVFAVSLGAPNKISYFLKSATNGGEGFFKVDRNSGEIFLTSVEVDAERVEEFILFIEAMDRSWEPSRVSFAYVFVTVNDSNDNAPVFEDTPYEEVIPESGYNDPIFTVKALDADASDVLQYSLLTENSPFKINIEGGLSVVGVLDVDNVTDSQSEYILVVSVSDGENDVSVNVTISVMDVNDNPPMFDEDDYIWELPEGEPLTNETFEVTVTDKDIDANISFSLEEGSDDRFQLLDSKFGEITVDGTFDRETKEEIMLVVIATDGKFTSETTVIVNVTDINDNNPEFIVSSSTSLEVAENAVIGTSVFVFMVIDADEPGTDNTQIMLVIQNETDFGVKVADDGDGYQLFVNGTLDRETRETYEMSIVASNPGFPEVSATLNVTVTLLDVNDNAPEFLVTDEDIALETAATFDLIANLSVIDRDKEPSYFKFSSSGEDSDLFFVSPTDEGCLVFLKSKLELSSEQYILDITVWNPDSPELKGDHSTIRLRFFYGELPCFEIVPKDPEVTLVNPGDTEFVVLTMGVNISDCSDPSAESDIMYNVTRATYKKGEWFLLFMTGVLSLTSPSPGLYVVLVEAYNSSEVDIPTLTIRMYSTVEVRLTEVANNPPMFTAPEFTIPTRILPVESGTELWDFGQNVTDADSPAFGNGELLFFIDGVECYGGCDASDVFNMTADGKLILAKDLTTIRTVNLTIRVQDRAVEPLNDTAPVSLGFRPASTGPPMFVDECETPILIQEESDEVDMLVCDLRAVSGDDLPVIYGFKGDEPGNFTINSATGKIFTTRAFDYDDSDRQFELLVFARTYDTSQERFTVVNVQIVDINDHAPEFLQDSYQFELDEDLNSACQNGSMPPGEWLVKATDDDSTLINQRVEYSIVGGSEDFTIDTTTGKISKLRCINAENYTSDVITLIINATNPDAILQPMGSTSVSFVIRDLNDNAPSFDEASYVPVWVPYLMSDNFIKSVVAQDEDVSEYNSGVTYSFATDQDLNVYRYFTIGETTGEIRTTNVTGYNTEELFGDDNEITIIVNARDNGSPPREADPQGIVAVLYDPNNLNEPYFPEKDREQEISPPENAENWCDEFTPPYITSSPEIVVLYSIIDVVPSNDTSTFFLPAIDQPKLCSSKTFDREQISEYVITIKAMSCNETEEDCSVSIARAGLRDRARRFSLEAVEANSGNADFSVVVITLRVEDVSDATPEFDKDFIEGANKFIIAVPDNTPYGQIIFQAKATDDDAQSRLNYSIEYDDHSSTPNFVMDDLTGRLAIQDRVESYLSDVTFDLNEEQAAYVVTLKVNDDLTNQSYKASSEVEAKIKLLFIYEYAILVIDKKPTDVSIESAALVECLSDELGLTLVVDSIEAHVILPRKVFFTYSPIEIDQKRTDVWVHMIDTKDGVKFVKYWDFKKMWDSNKGKSKCFSTDVTLRPPQPHSVVAEQFDESMIASFALLVMASFIGVSTVLMMVAILYMWHNVKLQGGRQISRRLDMLMLTENDQLSTQYSGVDNQGYEPDFVVEATTSIKEQDVKVDVGEQANLEAAGMSAEPIEPVYANVAKVSIQALQSEPKSALTKGPEHYESQEVVFDFPYEEESDTDTGVADFARIRTQAYPDNKEPSPDPASVEIVNEQPDKLDNADSSGAALMVSAEQQDKSDAQETTKDPTEAPSTEIPTGDVAEEPVQDLCPLQPEDVSKELSKEQASGSESDSKQEAFDDLPLPPPPPPTPGEVSADSEPATPKLDPKQATKELDNDIPFPPPPPEVLSNSNEKNKSTPSTPRSAHSEGHSSSQSPSEGTPLTDFENGEIPDFLENIEITKL

>MmEGFLAM

MDLISTFSLHFLLLACSLPPGAVSLRTALRKSGKVGPPLDIKLGALNCTAFSIQWKTPKRSGSSIIGYTV

FYSEVGSDKSLRERSHNVPVGQDTLITEEVIGDLKPGTEYQVSVAAYSQTGKGRLSFPRHVTTLSQDSCL

PPAAPQQPHVLVVSDSEVALSWRPGENEGSAPIQSYSVEFIRPDFDKSWTIIQERLQMDSMVIKGLDPDT

NYQFAVKAMNAHGFSPRSWPSNTVRTLGPGEAGSGHYGPGYITNPGVSEDDDGSEDELDLDVSFEEVKPL

PATKVGNKKFSVESKKTSVSNSVMGSRLAQPTSASLHETTVAIPPTPAQRKGKNSVAMMSRLFDMSCDET

LCSADSFCVNDYAWGGSRCHCNLGKGGEACSEDIFIQYPQFFGHSYVTFEPLKNSYQAFQVTLEFRAEAE

DGLLLYCGESEHGRGDFMSLALIRRSLHFRFNCGTGIAIIISETKIKLGAWHTVTLYRDGLNGMLQLNNG

TPVTGQSQGQYSKITFRTPLYLGGAPSAYWLVRATGTNRGFQGCVQSLSVNGKKIDMRPWPLGKALNGAD

VGECSSGICDEASCIHGGTCAAIKADSYICLCPLGFRGRHCEDAFALTIPQFRESLRSYAATPWPLEPQH

YLSFTEFEITFRPDSGDGVLLYSYDTGSKDFLSINMAAGHVEFRFDCGSGTGVLRSEAPLTLGQWHDLRV

SRTAKNGILQVDKQKVVEGMAEGGFTQIKCNTDIFIGGVPNYDDVKKNSGILHPFSGSIQKIILNDRTIH

VKHDFTSGVNVENAAHPCVGAPCAHGGSCRPRKEGYECDCPLGFEGLNCQKAIIEAIEIPQFIGRSYLTY

DNPNILKRVSGSRSNAFMRFKTTAKDGLLLWRGDSPMRPNSDFISLGLRDGALIFSYNLGSGVASIMVNG

SFSDGRWHRVKAVRDGQSGKITVDDYGARTGKSPGLMRQLNINGALYVGGMKEIALHTNRQYLRGLVGCI

SHFTLSTDYHISLVEDAVDGKNINTCGAK

>CgEGFLAM

MASYLTVIACFTTLLTWNIAENTNNVVLVAFFQGGCSKDSVCQHVCVDIDNGYFCRCHPGYSINKDGISC

SGSPLENTVDTANNQLFSKGGENKTLGSIQDENIAQNSAQRNEASISLHENSILGPASLMMYPTCRRNIC

KNYAKCSIENSMVKCHCPLGFIGKSCDKVRRVKYPQFSGVDSFLTLPRLTNGYKEFEISMKFKPSSDSGL

LLFTSEHPTGKGDFFSLALVNGHVEFRFDCGTGPAIIQSPNKVTIGQWNQVKAKRMENQGWLWMNSLGPI

SGLAQGAYTRITLRTELFLGGHRSMSSVQNRVNTMGGFRGCVQSLIFNQQQYDFRKATQHTDMELGPTYG

KTHSQLAQNVSIDAELGDAVDGQNIEDCSDGVCDDIVCHNGGSCKIISPDQFVCLCPLGFYGPDCVQKGK

IEVPEFKGHSVLQYQGLGRNSLSYTEIEITFKPTSPEGLILYNGYTNNKLGDYIAILMREGFAEFQFDLG

TGPAVIRSSLPVSLNSWHRIKVSRTGLQGVLEVDDQIPVQGLSKGAYTQLTLLQPLFVGGHPDFDITSRH

LNQSSSYQGCVQKLLLNGRPVRLMEEAIHGYNVEPCPHPCNSEPCQNSGKCVPAMGQYRCLCSVGFTEEN

CQKNLSISTAKFSGSSFLVYDSKDIKNRVSGKQFNLQIEIRGYSLRGLLFWTSENLPLHEGTGDFLSLGF

KGNELLFQYNLGSGRGVISYNKTQLSDGKWHTINAQRNGRYSSLTVDGTQTEEGTSVGMFSILNVKGPVY

IGGLPDVSYNTQSMYKTGFQGCIRDVVLANDFPLKLTESATRGQGVTQCPAD
