# Supplementary figures and images for: Genome-wide identification and spatiotemporal expression analysis of cadherin superfamily members in echinoderms
Source: EvoDevo. 2023 Dec 20;14:15. doi: 10.1186/s13227-023-00219-7 (PMC10734073; doi:10.1186/s13227-023-00219-7)

Figure S1

A

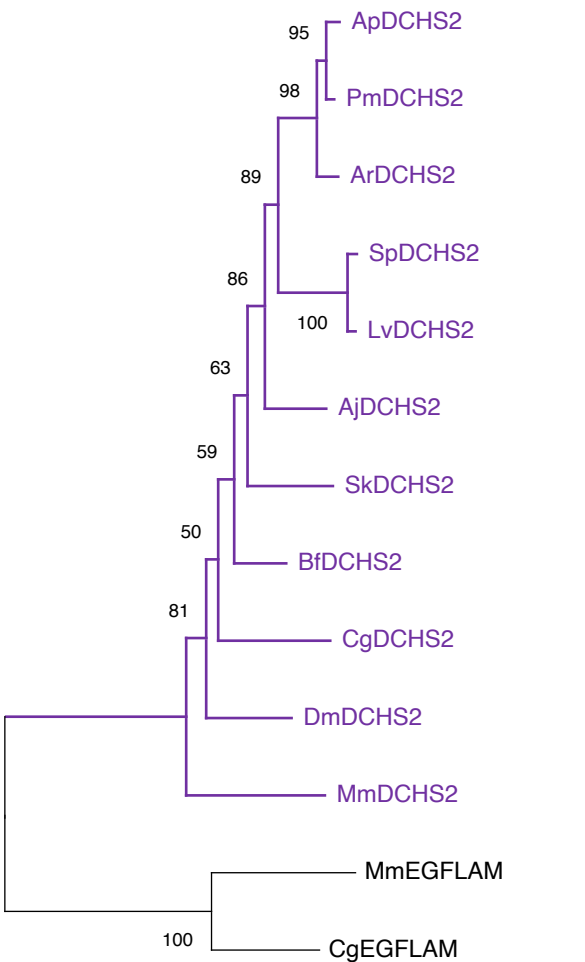

B

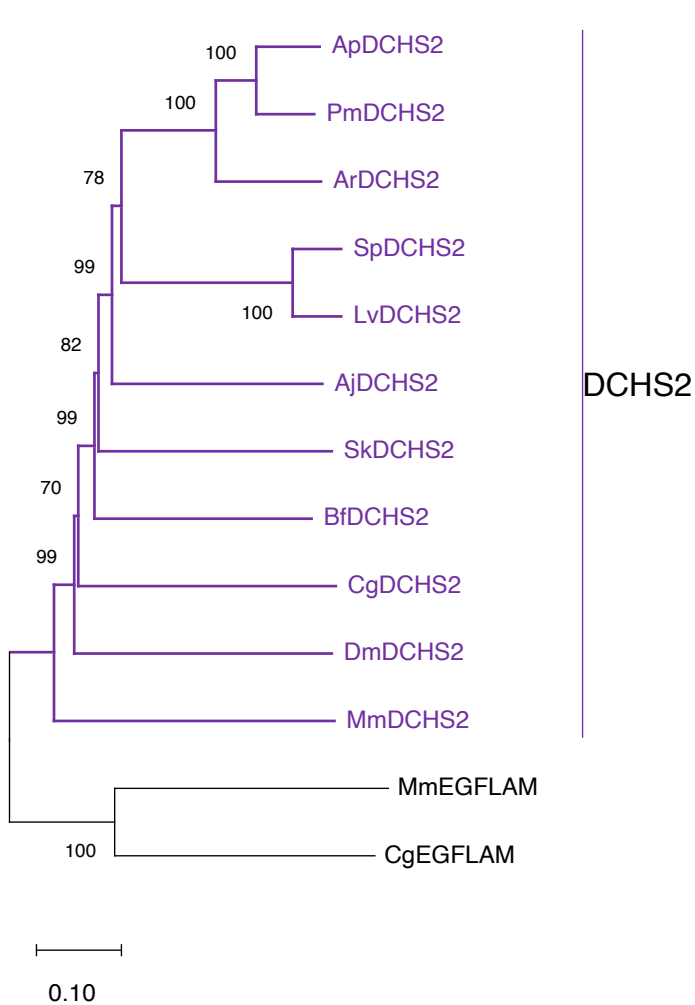

Figure S2

A

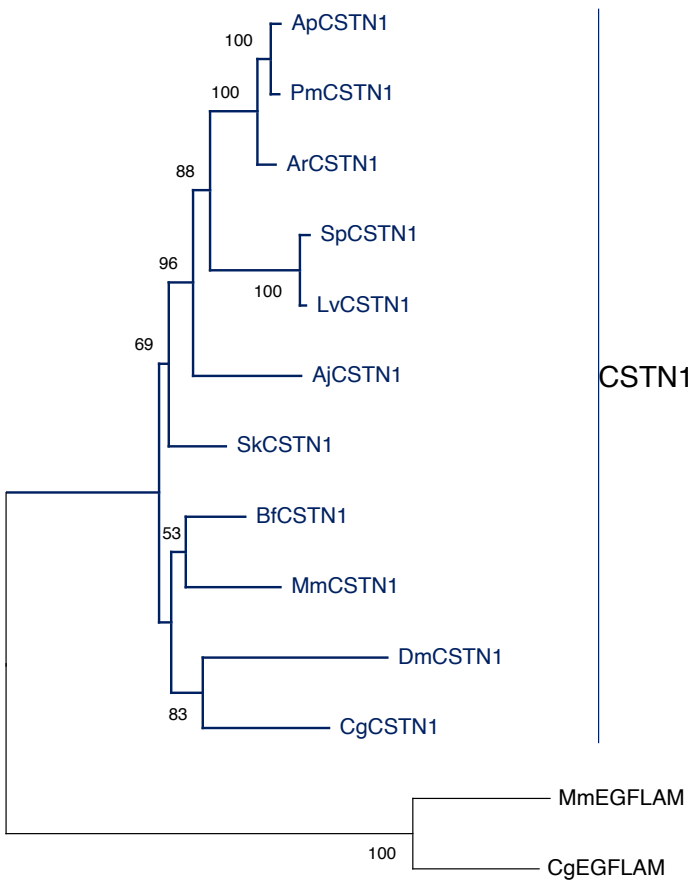

B

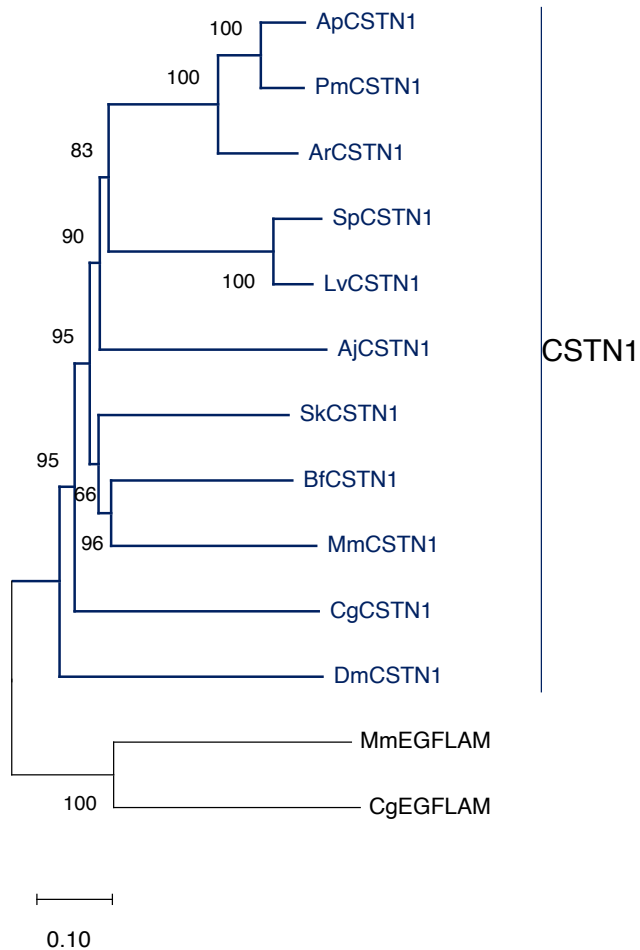

Figure S3

A

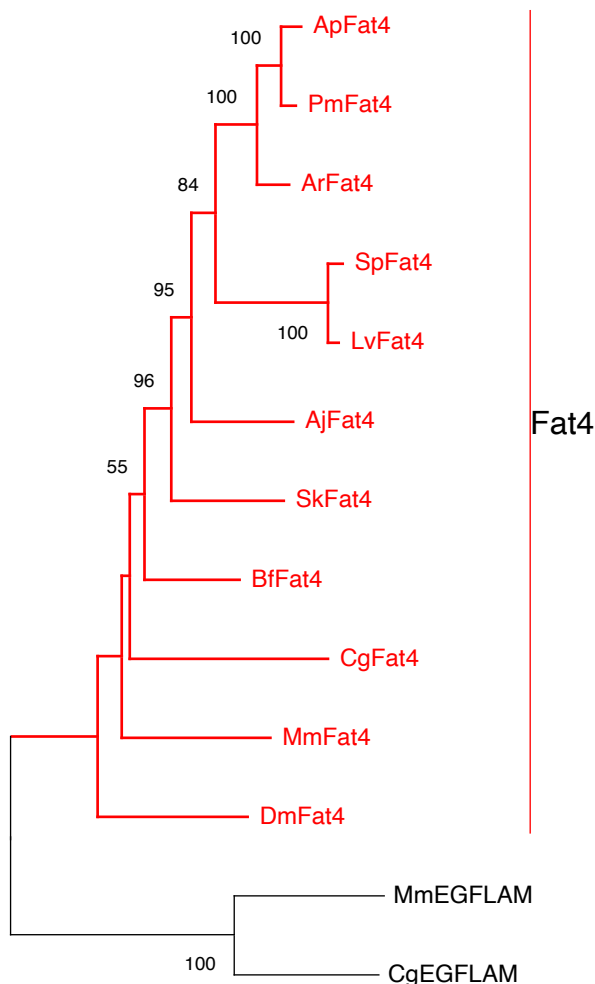

B

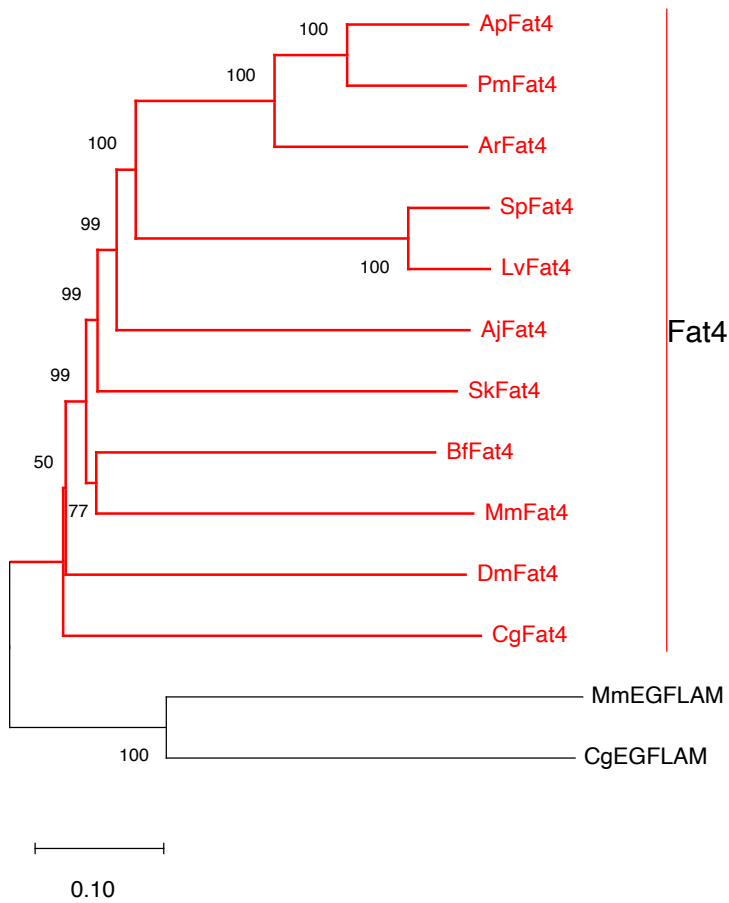

Supplement: Supplementary file 2 — Additional file 2: Fig. S1. Phylogenetic analysis of amino acid sequences for echinoderm DCHS2 utilizing maximum likelihood and neighbor-joining methods. Both analyses were conducted on echinoderm, non-echinoderm deuterostome, and protostome dachsous-2 (DCHS2) proteins using MEGA11 software. The clade representing the DCHS2 orthologs is colored purple. Two EGFLAM sequences from Mus musculus and Crassostrea gigas were included as an outgroup for both analyses. Full species and protein names for each taxon and their respective GenBank accession number(s) and their amino acid sequences are listed in Additional file 1. A. A maximum likelihood analysis was performed using the WAG+F+G substitution model with 500 bootstrap replicates utilizing amino acid sites with >90% coverage across all taxa. B. A neighbor-joining analysis was performed using the p-distance substitution model with 5000 bootstrap replicates with pairwise deletion of amino acid sites. Fig. S2. Phylogenetic analysis of amino acid sequences for echinoderm CSTN1 utilizing maximum likelihood and neighbor-joining methods. Both analyses were conducted on echinoderm, non-echinoderm deuterostome, and protostome calsyntenin-1 (CSTN1) proteins using MEGA11 software. The clade representing the CSTN1 orthologs is colored dark blue. Two EGFLAM sequences from Mus musculus and Crassostrea gigas were included as an outgroup for both analyses. Full species and protein names for each taxon and their respective GenBank accession number(s) and their amino acid sequences are listed in Additional file 1. A. A maximum likelihood analysis was performed using the WAG+F+G substitution model with 500 bootstrap replicates utilizing amino acid sites with >90% coverage across all taxa. B. A neighbor-joining analysis was performed using the p-distance substitution model with 5000 bootstrap replicates with pairwise deletion of amino acid sites. Fig. S3. Phylogenetic analysis of amino acid sequences for echinoderm Fat4 utilizing maximum [file 13227_2023_219_MOESM2_ESM.pdf]

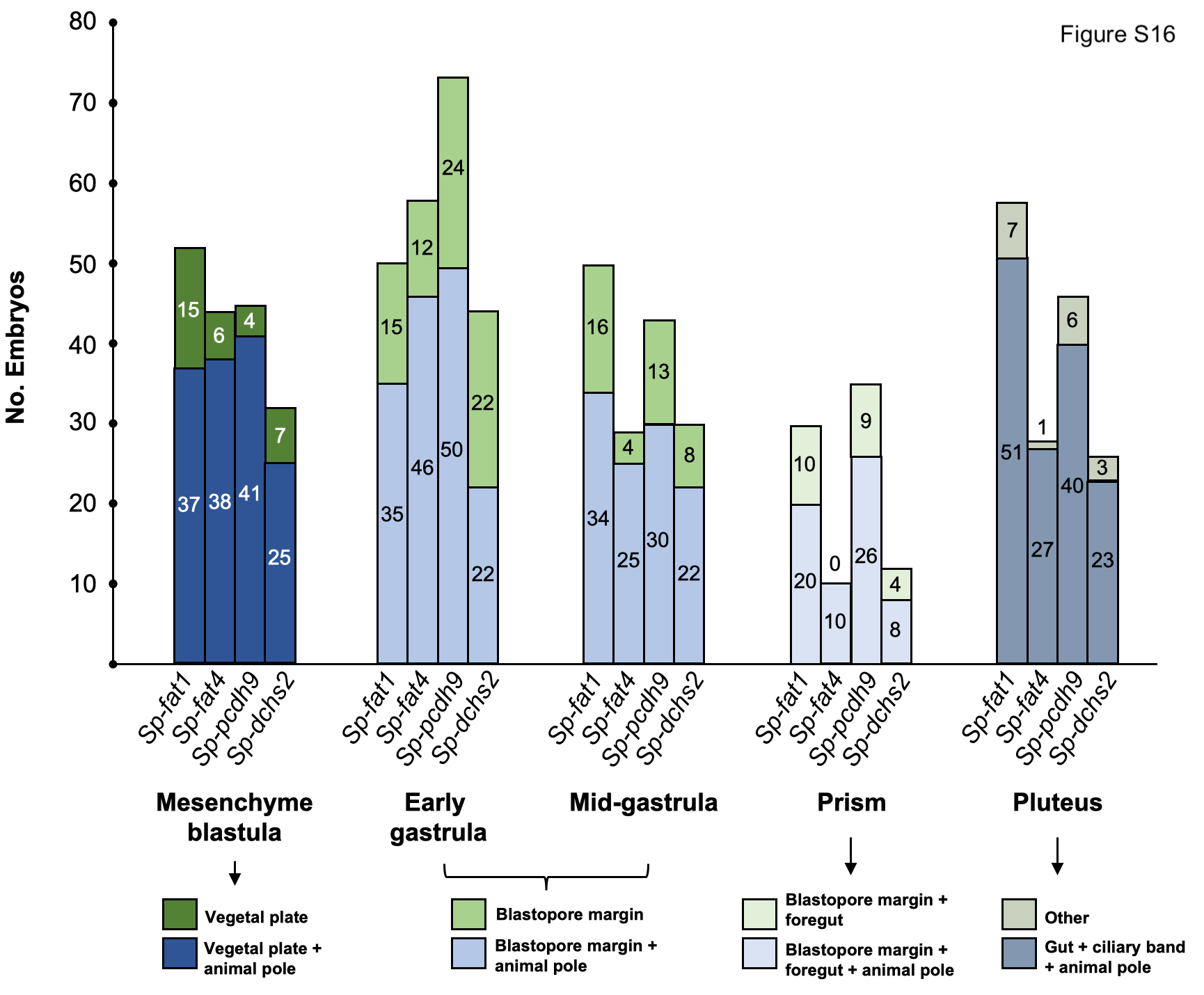

Supplement: Supplementary file 4 — Additional file 4: Fig. S16. Quantification of whole mount in situ hybridization expression patterns. For each developmental stage and gene, the number of embryos that exhibited elevated expression in the indicated region(s) is shown. The probes that were utilized for each gene, which have their nucleotide sequences listed in Additional file 5, are as follows: Sp-dchs2—Probe 2, Sp-fat1—Probe 2, Sp-fat4—Probe 1, Sp-pcdh9—Probe 1. [file 13227_2023_219_MOESM4_ESM.png]
