# Supplementary material for: Genome-wide identification and spatiotemporal expression analysis of cadherin superfamily members in echinoderms
Source: EvoDevo. 2023 Dec 20;14:15. doi: 10.1186/s13227-023-00219-7 (PMC10734073; doi:10.1186/s13227-023-00219-7)
Supplement: Supplementary file 3 — Additional file 3: Fig. S4. Comparison of echinoderm cadherin-23 (CDH23) structure to other bilaterians. Protein domain structures for various echinoderm, non-echinoderm deuterostome, and protostome species were visualized using SMART. Transmembrane domains are shown as blue rectangles while predicted signal peptides are shown in red. Class names for each representative taxon are given in parentheses. Fig. S5. Comparison of echinoderm cadherin-88C (CDH88C) structure to other bilaterians. Protein domain structures for various echinoderm, non-echinoderm deuterostome, and protostome species were visualized using SMART. Transmembrane domains are shown as blue rectangles while predicted signal peptides are shown in red. Class names for each representative taxon are given in parentheses. Drosophila melanogaster was included as a representative protostome species. Fig. S6. Comparison of echinoderm calsyntenin-1 (CSTN1) structure to other bilaterians. Protein domain structures for various echinoderm, non-echinoderm deuterostome, and protostome species were visualized using SMART. Transmembrane domains are shown as blue rectangles while predicted signal peptides are shown in red. Class names for each representative taxon are given in parentheses. Drosophila melanogaster was included as a representative protostome species. Fig. S7. Comparison of echinoderm fat atypical cadherin 1 (Fat1) structure to other bilaterians. Protein domain structures for various echinoderm, non-echinoderm deuterostome, and protostome species were visualized using SMART. Transmembrane domains are shown as blue rectangles while predicted signal peptides are shown in red. Class names for each representative taxon are given in parentheses. Drosophila melanogaster was included as a representative protostome species. Fig. S8. Comparison of echinoderm protocadherin-15 (PCDH15) structure to other bilaterians. Protein domain structures for various echinoderm, non-echinoderm deuterostome, and protostome speci [file 13227_2023_219_MOESM3_ESM.pptx]

## Slide 1
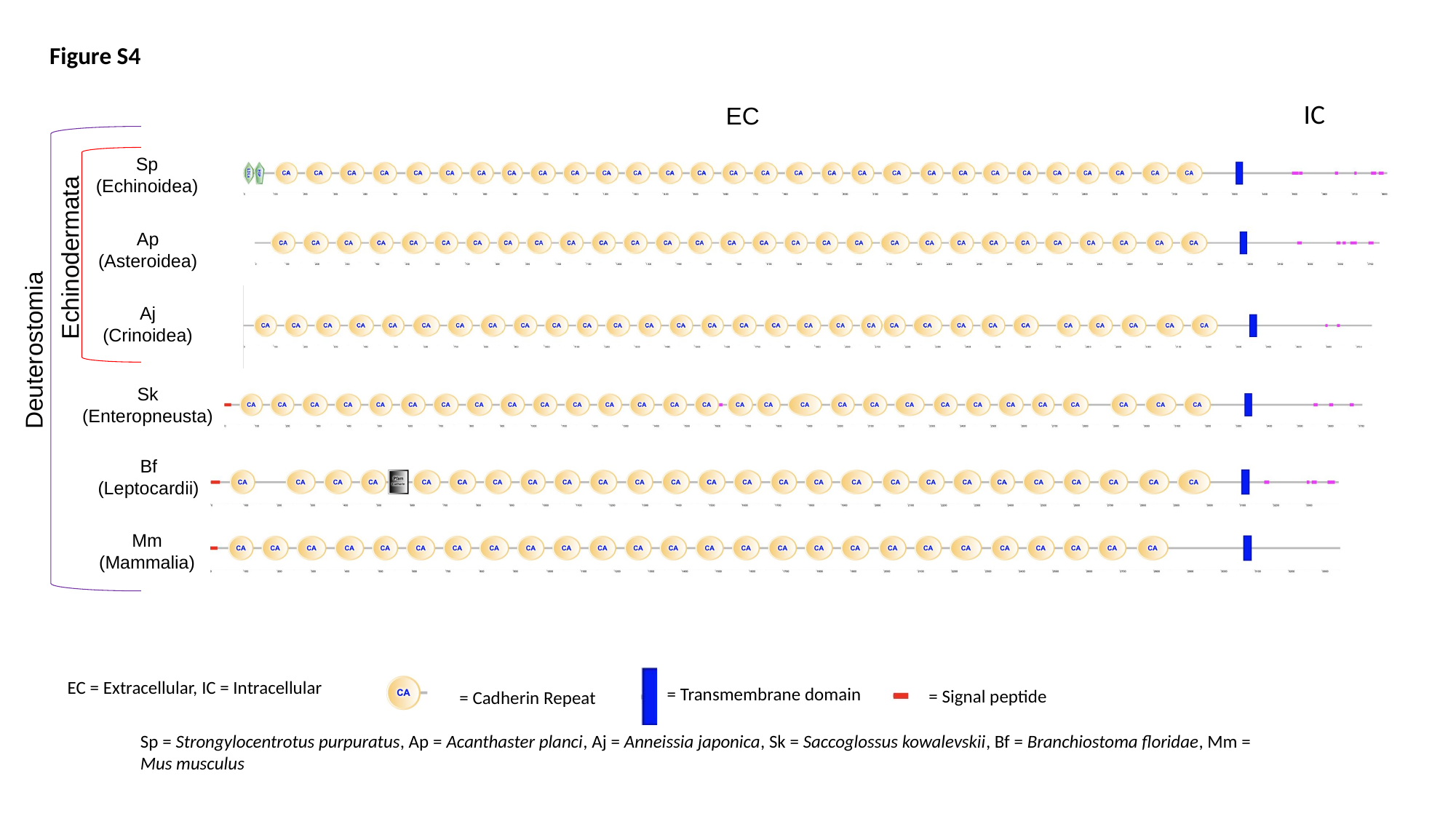

Figure S4
IC
EC
Sp
(Echinoidea)
Echinodermata
Ap
(Asteroidea)
Aj
(Crinoidea)
Deuterostomia
Sk
(Enteropneusta)
Bf
(Leptocardii)
Mm
(Mammalia)
EC = Extracellular, IC = Intracellular
= Cadherin Repeat
= Transmembrane domain
= Signal peptide
Sp = Strongylocentrotus purpuratus, Ap = Acanthaster planci, Aj = Anneissia japonica, Sk = Saccoglossus kowalevskii, Bf = Branchiostoma floridae, Mm = Mus musculus

## Slide 2
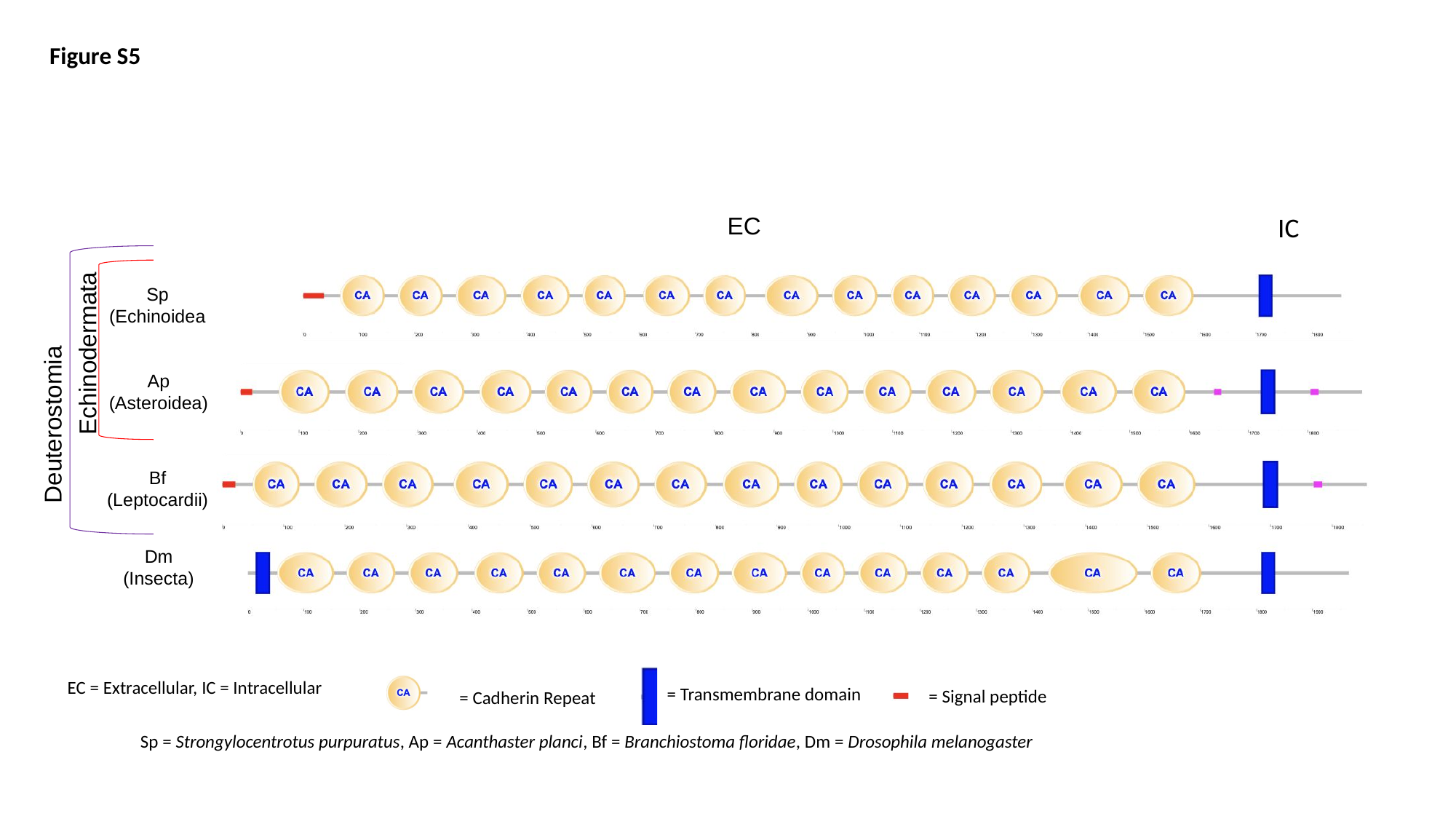

Figure S5
EC
IC
Sp
(Echinoidea
Echinodermata
Ap
(Asteroidea)
Deuterostomia
Bf
(Leptocardii)
Dm
(Insecta)
EC = Extracellular, IC = Intracellular
= Cadherin Repeat
= Transmembrane domain
= Signal peptide
Sp = Strongylocentrotus purpuratus, Ap = Acanthaster planci, Bf = Branchiostoma floridae, Dm = Drosophila melanogaster

## Slide 3
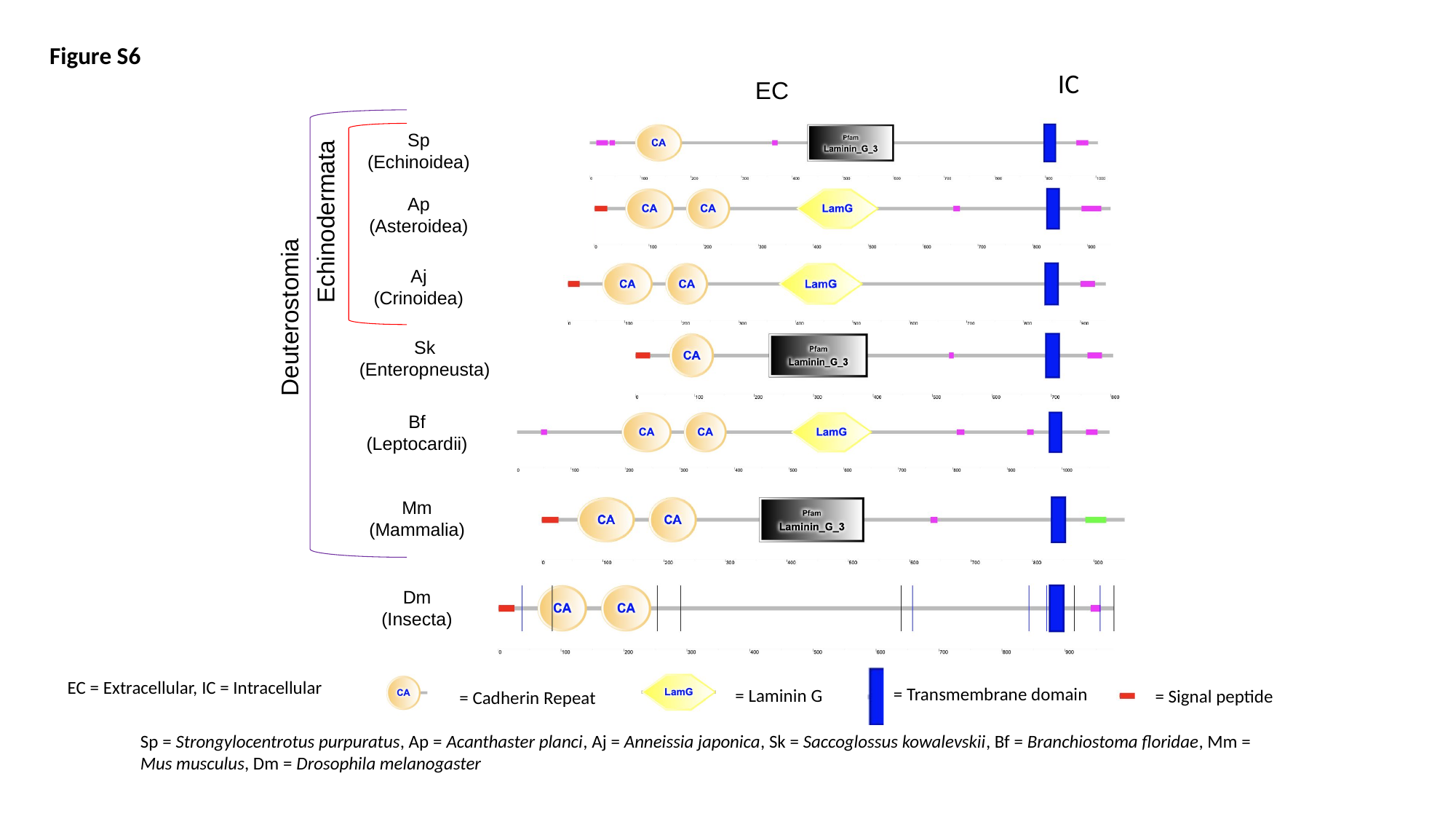

Figure S6
IC
EC
Sp
(Echinoidea)
Echinodermata
Ap
(Asteroidea)
Aj
(Crinoidea)
Deuterostomia
Sk
(Enteropneusta)
Bf
(Leptocardii)
Mm
(Mammalia)
Dm
(Insecta)
EC = Extracellular, IC = Intracellular
= Cadherin Repeat
= Laminin G
= Transmembrane domain
= Signal peptide
Sp = Strongylocentrotus purpuratus, Ap = Acanthaster planci, Aj = Anneissia japonica, Sk = Saccoglossus kowalevskii, Bf = Branchiostoma floridae, Mm = Mus musculus, Dm = Drosophila melanogaster

## Slide 4
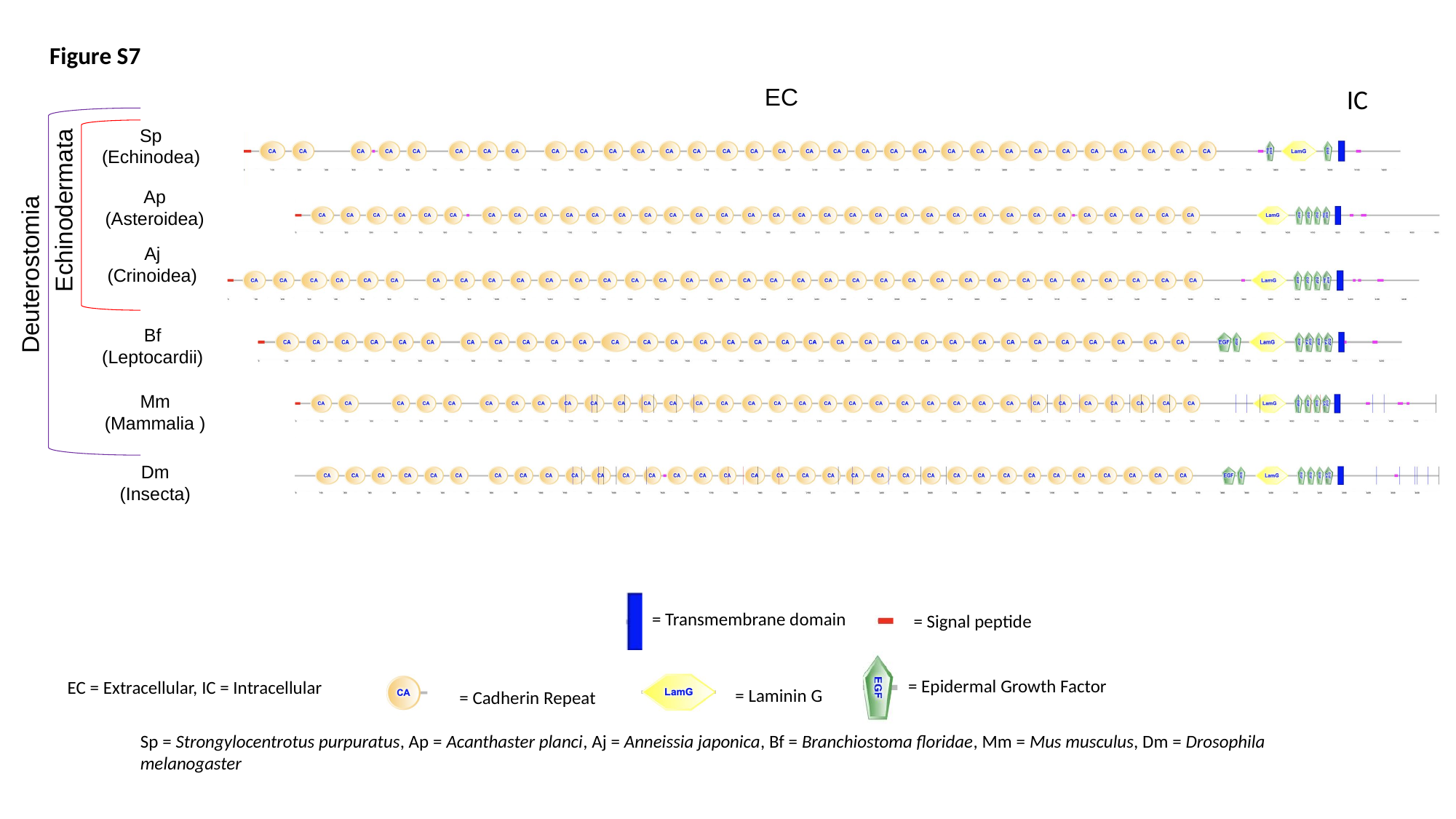

Figure S7
EC
IC
Sp
(Echinodea)
Ap
(Asteroidea)
Echinodermata
Deuterostomia
Aj
(Crinoidea)
Bf
(Leptocardii)
Mm
(Mammalia )
Dm
(Insecta)
= Transmembrane domain
= Signal peptide
= Epidermal Growth Factor
EC = Extracellular, IC = Intracellular
= Cadherin Repeat
= Laminin G
Sp = Strongylocentrotus purpuratus, Ap = Acanthaster planci, Aj = Anneissia japonica, Bf = Branchiostoma floridae, Mm = Mus musculus, Dm = Drosophila melanogaster

## Slide 5
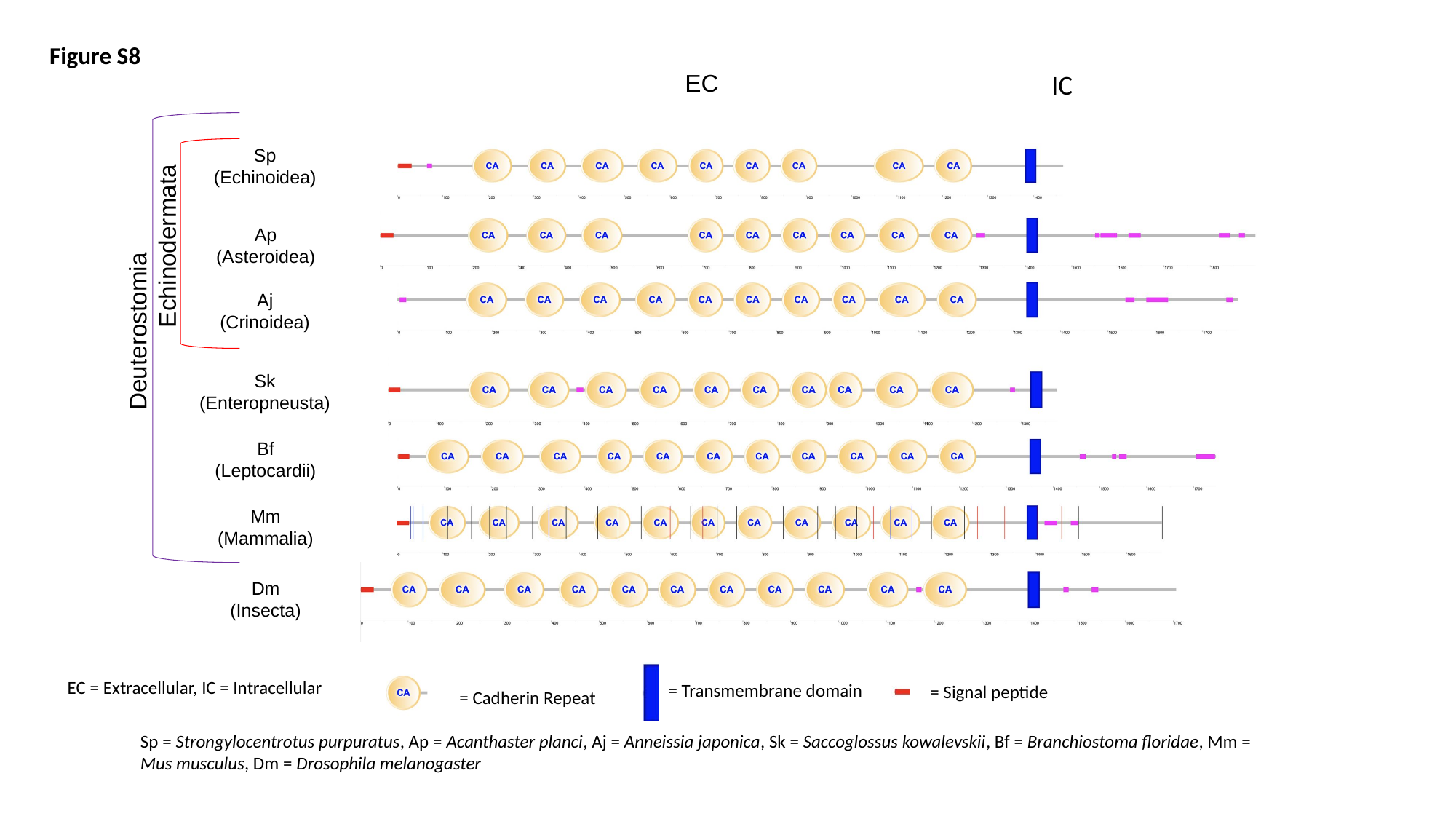

Figure S8
EC
IC
Sp
(Echinoidea)
Echinodermata
Ap
(Asteroidea)
Aj
(Crinoidea)
Deuterostomia
Sk
(Enteropneusta)
Bf
(Leptocardii)
Mm
(Mammalia)
Dm
(Insecta)
EC = Extracellular, IC = Intracellular
= Cadherin Repeat
= Transmembrane domain
= Signal peptide
Sp = Strongylocentrotus purpuratus, Ap = Acanthaster planci, Aj = Anneissia japonica, Sk = Saccoglossus kowalevskii, Bf = Branchiostoma floridae, Mm = Mus musculus, Dm = Drosophila melanogaster

## Slide 6
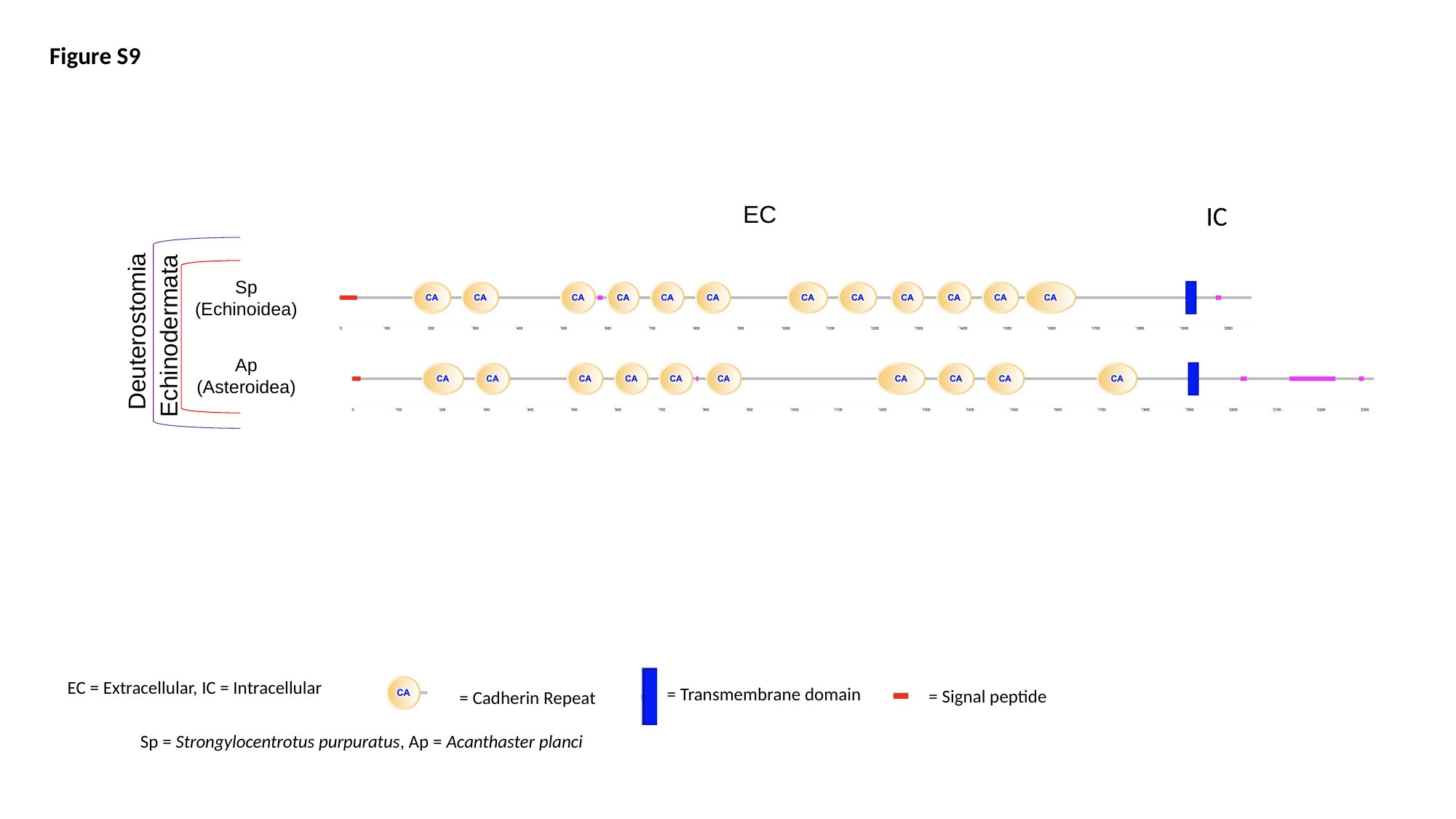

Figure S9
EC
IC
Sp
(Echinoidea)
Echinodermata
Deuterostomia
Ap
(Asteroidea)
EC = Extracellular, IC = Intracellular
= Cadherin Repeat
= Transmembrane domain
= Signal peptide
Sp = Strongylocentrotus purpuratus, Ap = Acanthaster planci

## Slide 7
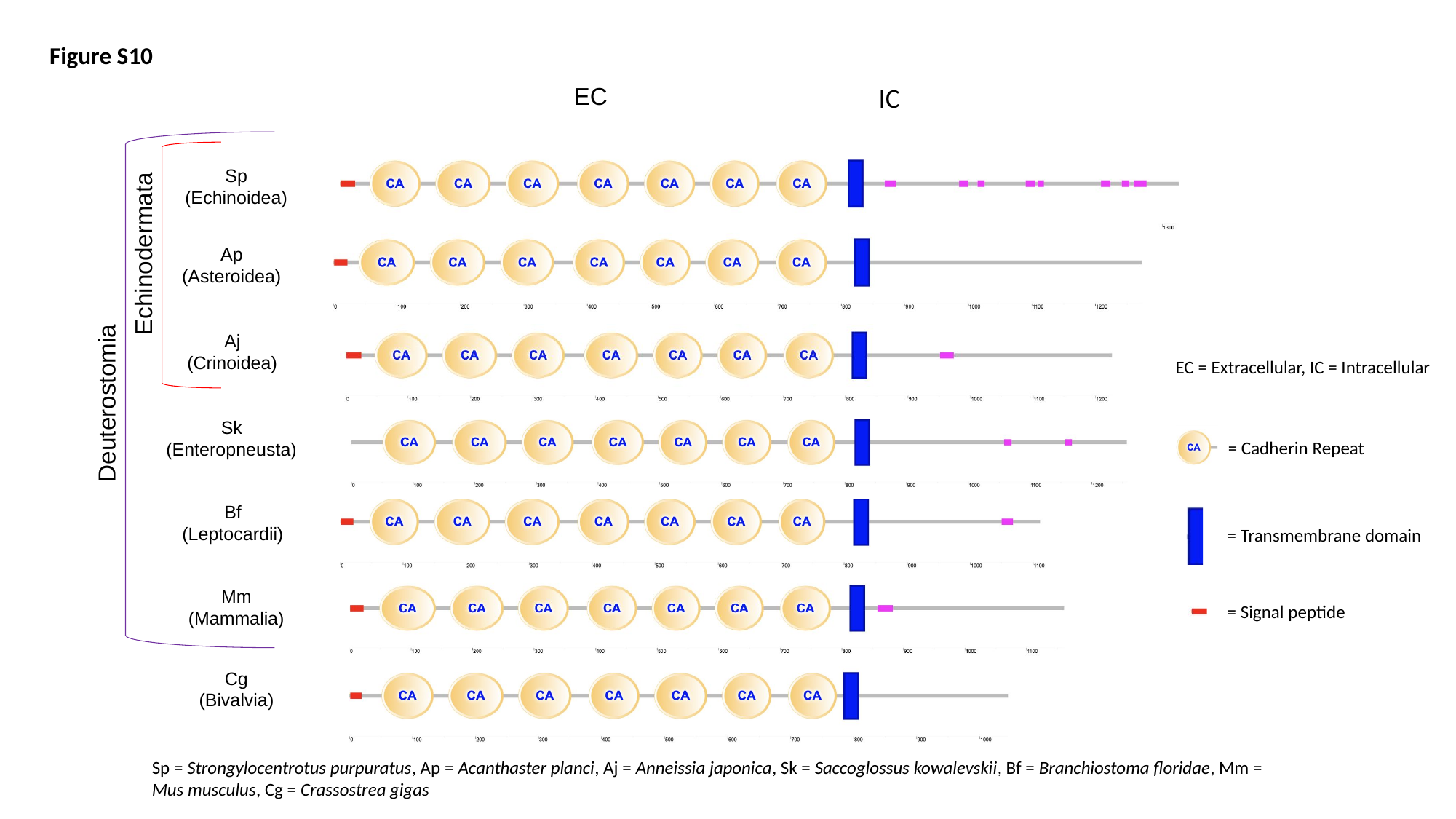

Figure S10
EC
IC
Sp
(Echinoidea)
Echinodermata
Ap
(Asteroidea)
Aj
(Crinoidea)
EC = Extracellular, IC = Intracellular
Deuterostomia
Sk
(Enteropneusta)
= Cadherin Repeat
Bf
(Leptocardii)
= Transmembrane domain
Mm
(Mammalia)
= Signal peptide
Cg
(Bivalvia)
Sp = Strongylocentrotus purpuratus, Ap = Acanthaster planci, Aj = Anneissia japonica, Sk = Saccoglossus kowalevskii, Bf = Branchiostoma floridae, Mm = Mus musculus, Cg = Crassostrea gigas

## Slide 8
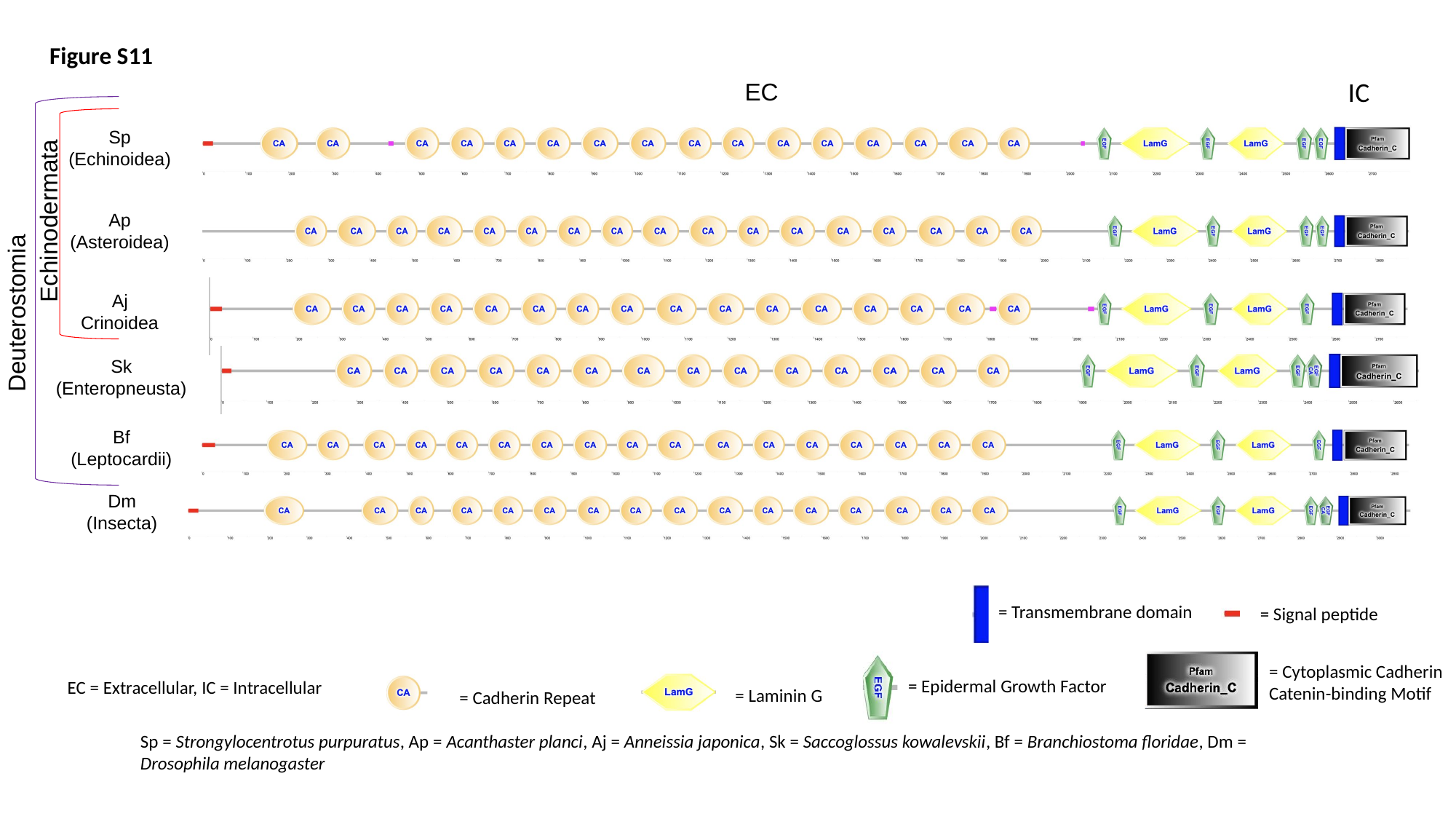

Figure S11
IC
EC
Sp
(Echinoidea)
Echinodermata
Ap
(Asteroidea)
Aj
Crinoidea
Deuterostomia
Sk
(Enteropneusta)
Bf
(Leptocardii)
Dm
(Insecta)
= Transmembrane domain
= Signal peptide
= Epidermal Growth Factor
= Cytoplasmic Cadherin
Catenin-binding Motif
EC = Extracellular, IC = Intracellular
= Cadherin Repeat
= Laminin G
Sp = Strongylocentrotus purpuratus, Ap = Acanthaster planci, Aj = Anneissia japonica, Sk = Saccoglossus kowalevskii, Bf = Branchiostoma floridae, Dm = Drosophila melanogaster

## Slide 9
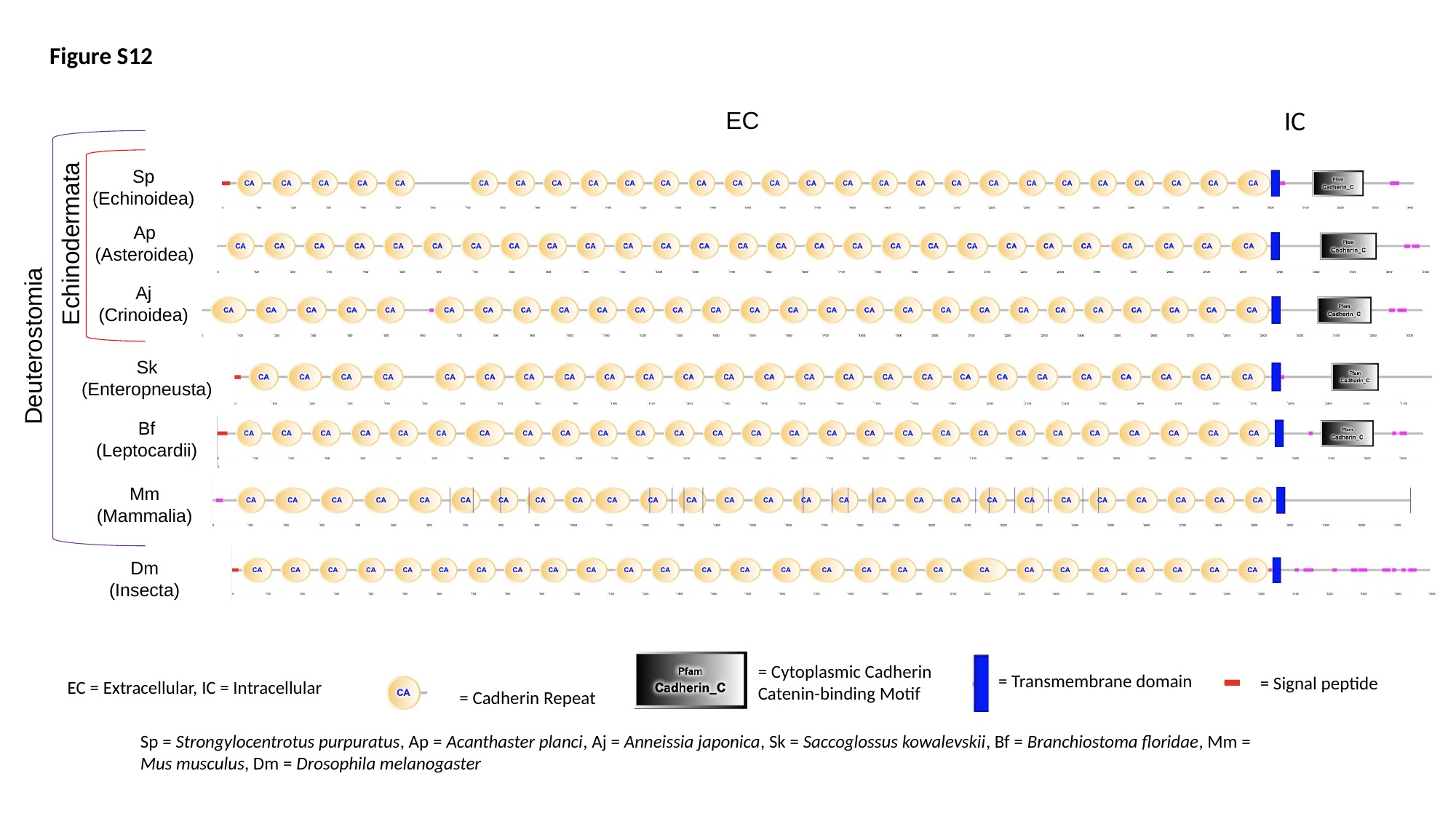

Figure S12
IC
EC
Sp
(Echinoidea)
Ap
(Asteroidea)
Echinodermata
Aj
(Crinoidea)
Deuterostomia
Sk
(Enteropneusta)
Bf
(Leptocardii)
Mm
(Mammalia)
Dm
(Insecta)
= Cytoplasmic Cadherin
Catenin-binding Motif
= Transmembrane domain
= Signal peptide
EC = Extracellular, IC = Intracellular
= Cadherin Repeat
Sp = Strongylocentrotus purpuratus, Ap = Acanthaster planci, Aj = Anneissia japonica, Sk = Saccoglossus kowalevskii, Bf = Branchiostoma floridae, Mm = Mus musculus, Dm = Drosophila melanogaster

## Slide 10
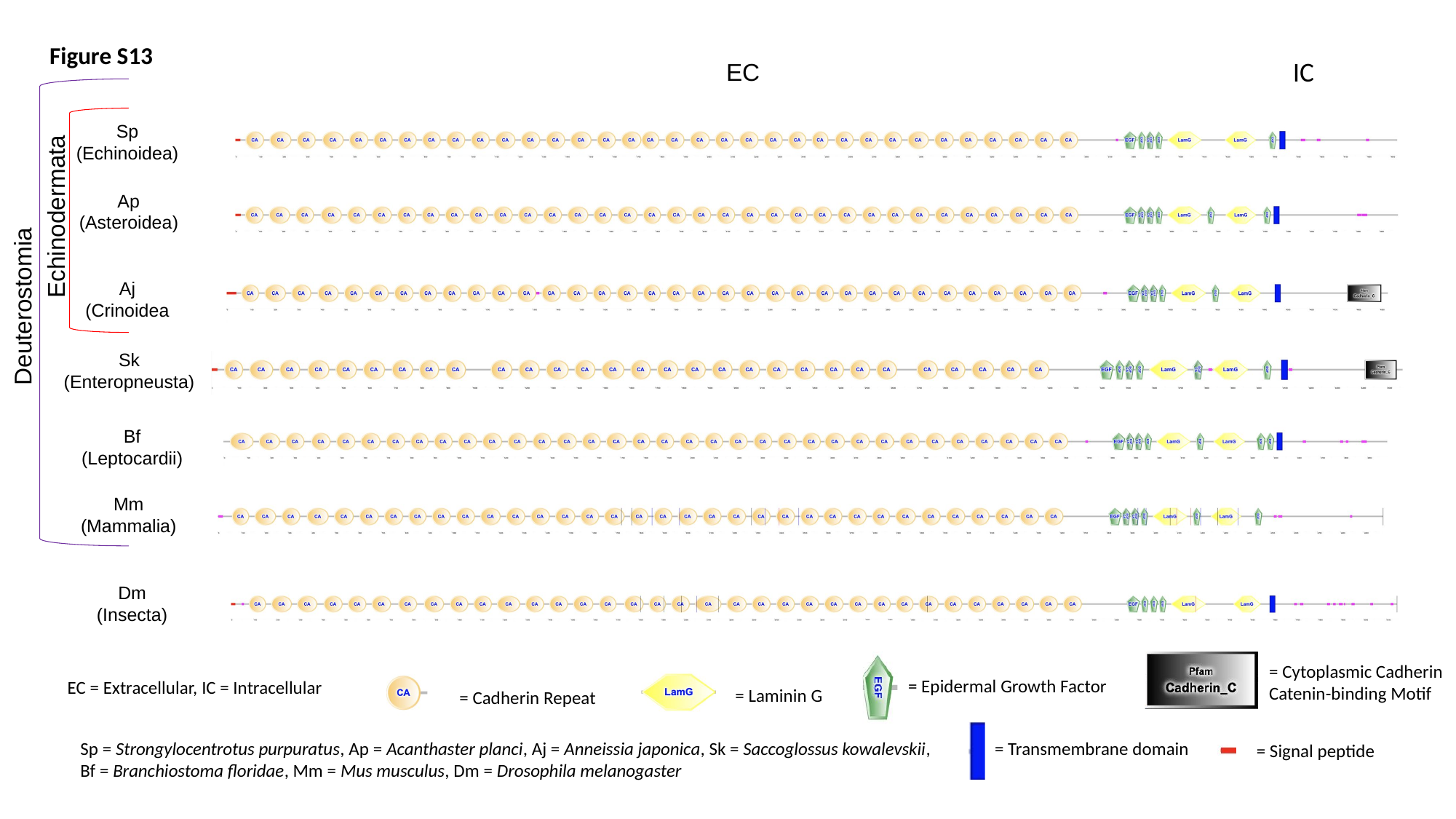

Figure S13
IC
EC
Sp
(Echinoidea)
Echinodermata
Ap
(Asteroidea)
Aj
(Crinoidea
Deuterostomia
Sk
(Enteropneusta)
Bf
(Leptocardii)
Mm
(Mammalia)
Dm
(Insecta)
= Epidermal Growth Factor
= Cytoplasmic Cadherin
Catenin-binding Motif
EC = Extracellular, IC = Intracellular
= Cadherin Repeat
= Laminin G
Sp = Strongylocentrotus purpuratus, Ap = Acanthaster planci, Aj = Anneissia japonica, Sk = Saccoglossus kowalevskii, Bf = Branchiostoma floridae, Mm = Mus musculus, Dm = Drosophila melanogaster
= Transmembrane domain
= Signal peptide

## Slide 11
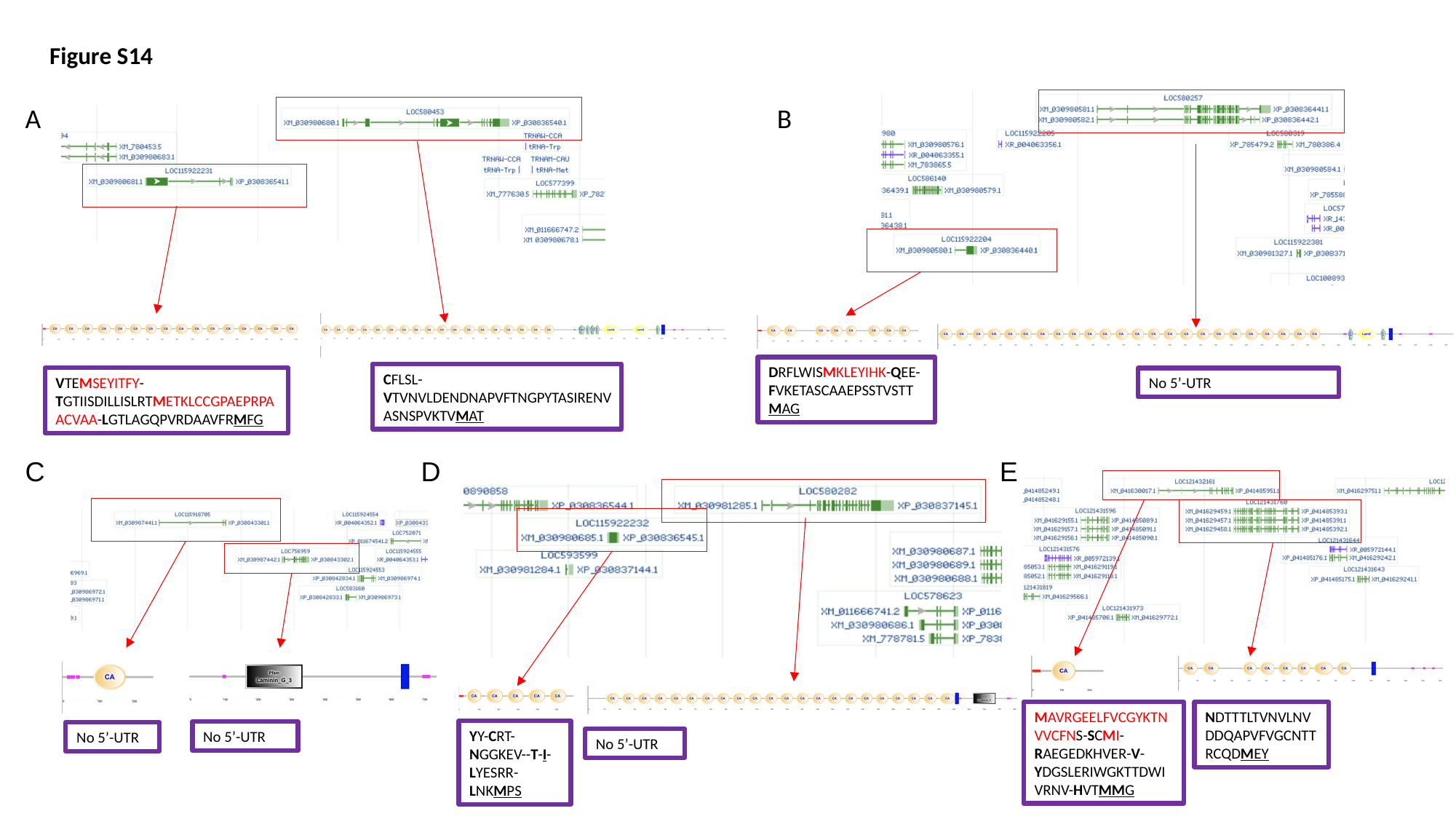

Figure S14
A
B
DRFLWISMKLEYIHK-QEE-FVKETASCAAEPSSTVSTTMAG
CFLSL-VTVNVLDENDNAPVFTNGPYTASIRENVASNSPVKTVMAT
VTEMSEYITFY-TGTIISDILLISLRTMETKLCCGPAEPRPAACVAA-LGTLAGQPVRDAAVFRMFG
No 5’-UTR
C
D
E
MAVRGEELFVCGYKTNVVCFNS-SCMI-RAEGEDKHVER-V-YDGSLERIWGKTTDWIVRNV-HVTMMG
NDTTTLTVNVLNVDDQAPVFVGCNTTRCQDMEY
YY-CRT-NGGKEV--T-I-LYESRR-LNKMPS
No 5’-UTR
No 5’-UTR
No 5’-UTR

## Slide 12
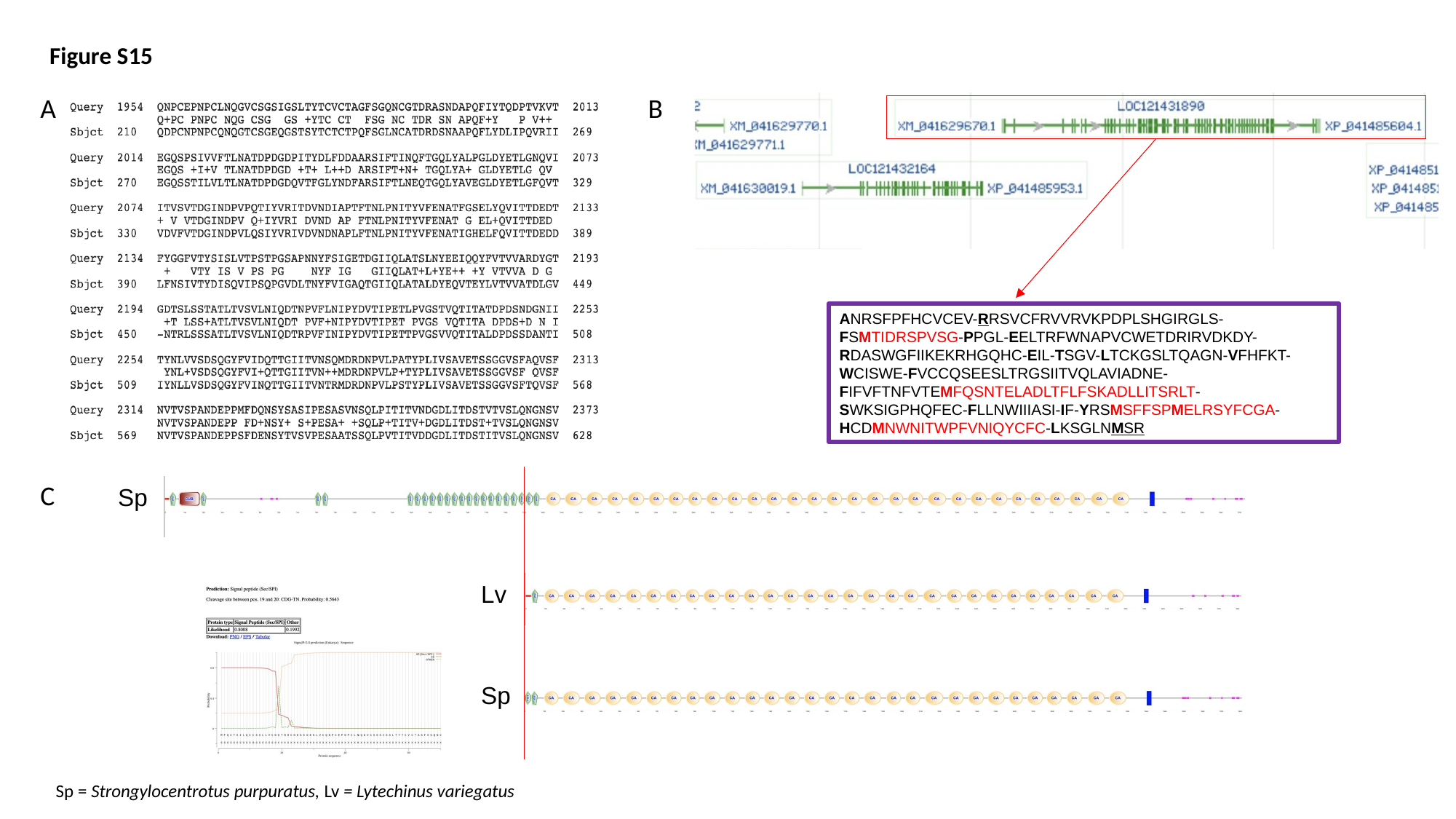

Figure S15
A B
ANRSFPFHCVCEV-RRSVCFRVVRVKPDPLSHGIRGLS-FSMTIDRSPVSG-PPGL-EELTRFWNAPVCWETDRIRVDKDY-RDASWGFIIKEKRHGQHC-EIL-TSGV-LTCKGSLTQAGN-VFHFKT-WCISWE-FVCCQSEESLTRGSIITVQLAVIADNE-FIFVFTNFVTEMFQSNTELADLTFLFSKADLLITSRLT-SWKSIGPHQFEC-FLLNWIIIASI-IF-YRSMSFFSPMELRSYFCGA-HCDMNWNITWPFVNIQYCFC-LKSGLNMSR
C
Sp
Lv
Sp
Sp = Strongylocentrotus purpuratus, Lv = Lytechinus variegatus
