# Supplementary material for: Genome-wide identification and spatiotemporal expression analysis of cadherin superfamily members in echinoderms
Source: EvoDevo. 2023 Dec 20;14:15. doi: 10.1186/s13227-023-00219-7 (PMC10734073; doi:10.1186/s13227-023-00219-7)
Supplement: Supplementary file 5 — Additional file 5: Sequences of probes used for in situ hybridization. Nucleotide sequences for DNA probes that were complementary to target mRNAs corresponding to each cadherin-encoding gene were designed using nonoverlapping regions within the Strongylocentrotus purpuratus version 5.0 genome. Target specificity of probe sequences to only their intended target mRNAs, which are numbered and bolded, was confirmed using BlastN. [file 13227_2023_219_MOESM5_ESM.docx]

Supplementary File- Sequences of probes used for *in situ* hybridization

**1) Sp-fat1**

**Probe 1**

ATTCGTGAAGACCTCCCAACAGGAGCCGCCGTCTTGAGCGTTCAAGCTCATGATCCTGATAACCCACCCAATGGAACTGTCCGTTACAGACTTGATACTTCTGGGTCTTCCAAGTTCAGCATTGACACAGATTATGGCACTCTTCGGCTGGTAGAGATGCTTGACTATGAAGTGACCCAGCTTTATGAGATCGTCGTTGCCATCCGAGATGAAGGAATACCAGCTATGAGCTCAACATGCAGGATAGTCGTTGAGGTTGTGGACGTCAATGAGAACACTCATGCTCCTGTATTCCCGAACTATGTGCTGACTGGATCTGTACATGAAGGACATATCAATGATACCGAAGTGATGACCATATCCGCTGCTGACATGGATATGGGCATAGATGGAGAGGTGTCCTACACAATCCGAGATGGAAGTGGACTTGGAAGGTTCAGCATTGACAGTTCAGGTTAGTTTTGTTGATTTGTAAATTTCTTTTGAATCTTTTTTGTATTTGTTCATAGAACTTGATGAACTTGGCACGTTAAAATGCAACAAATGTGAAAACCATTGCTAAAAGATGGAACACATTTACAATGTCTAGATATGACTTTGTTTAAAGATTGAGAATAATAACTGACATCTACATGTATCTCATGGATGTCATTGTGTGGTTTTTATGAAATGAAGTGAAACGACTGAAGACCACAAAATCGATGATTATGGGTAGATCTAGAATTCTAGGCTAAATTTTAAGGAATCGGTTTTTCCATCCAAACTGCTAGGTGTATGATTAGAGGGCAGTATATTATACTGGAGCCAGTATATATAAAACTGATAATGTTATATTGCACCTAAATTGGTCTCCCGCTAAGATGGGTGAACTTGCAGAAACACCATGGACTGGGATTTATGCCATGATGATGAAGAAAGTGTATTCCATGCAAAGATGATCTCTCAAGCAGCTGTGTGAGTGGTGTAATTTGAGGAGAATGGCATTTTAATTTGATTTTAGGCTATTTCCTGTCGGCAAAATGACAGCTTGGTTTGTCAATGGGCTCATCATTTCTTGGCTCATTAGTGAAGAGGAGAAGCCTGGGATGAAAT

**Probe 2**

CTGGAAGGAGAAGAGGGATCTACCTTCCAATTCTTTGTTGGTGTGACTGATGGTGGAAATCCTCCACTGAACTGGGTGACAGCAGCAGAGATCTACGTTCTCTCCCAAGATGATGAACTGCCATTCTTTGATGGGCTGGTCAACGAACCTGACATTGATGTCGCAGAGAACACACCAGTGGGAACAATCCTATCCACCTTCTTGGCTCATAGCAACTCATCGCTCACCTATTCCTTGGTTCCTGGTAACGAAGCCCACACCAACGATCCATCGCACTTCTCCATCGATGAGCACGGCAACCTGACGTTGGTGAGCTCCTTGGACTTTGAGTCTGTCTCTTGGTACAAGCTCATCATCAAGGCCAGCACAAACACAGAGCCATCTATAGCTACCTACTTCACCGTCCTTGTGACTGTGACCGATGTCAACGACAACATGCCTGTCTTTGAAGACACAACGTACAGCATCAGCGTTCCAGAGAACACTCCGGTTGATGATGCTATCCTGAAGGTTATTGCTAGGGATGCGGATGCTGGGTCCAACGGCCAAGTCCTCTACTACCTTGATGTTGAGAATGACCTGGAAGTGTTTGAGAAGTTTGAGGTCAACGAGATGTCAGGTATGTTGACCATCCAAGAGGGTCTTGACCGGGAGACGGCCGTCACCTACGACATCCTCATCCTAGCCAGGGATGTCACCAACGCTGATTTCTCAGCAACTGCCACGGTTCACGTCACTGTGGTTGACTTCAACGACTCGCCGCCACGTTTCACGCAGAAGGTCTATCCGGGAGAAGTCCTCGAAAGTGATCCCATCGGAACCGTGACTGTCTCCATCCAAGCTGTTGATGGCGACGTTGGCACCAACGCTCACGTCGTCTACTACATCACATCAGGGGACCCATTCAATCACTTTGCCATCGAGAGCAACTCTGGCAAGATCTACGTTGCTAACCCTCTGGATCGGGAGCTGAAGGATACGTATCGCCTCAATGTAACAGCCACCGA

**2) Sp-fat4**

**Probe 1**

GACCAGTATTCGGGCATGTTCAGTCTAGATCCAGAGAGTGGATTGCTGCAAATAACAGTACCCCTAGATCGTGAAGAACAAGATTCCTATGAACTTGAAATCTTGGCATCAGATGGTGGTTCCCCTTCACTTCAGGCCACAGCTACTGTCACTATTGCTGTATTAGATCAAAATGACAACCCTCCACAGTTCTACCCTACAGAGTACTATGCCAGTGTGATAGAGAATGGACCAGCTGGACTCTTTGTGACTGTGGTTTCAGCAACAGATCTTGATGTGGGTGTTAACGGAGCTGTCATTTTCTCTCTGTCAGACTCATCCAAATTCCAGATTGACAGCTATTCCGGAAACGTTACCACACGAGAGTCACTGGACAGGGAACAGGAATCTTCATATACCCTGACAGTAACTGCACAAGATGGTAGTGGACAGGCTGCTGCTCAGTCTGCGACAATTTTTGTTACTGTCACTGATACACTGGACAATCCACTTGAATTTGAACTGAATCCTTACAGGTTTCAACTGGAAGAGAATCGTCCAAATCGCACTGAAGTTGGAACTGTCCTAGCTACAAGTATGGATCTGAATGTTGAAATCACATACGTGATTGTAAATGGTGATCCAAATGGACTGTTTGTGATAGACTTCTATTCTGGTGTTATCAAGACGACGCTCTCAATTGATCGAGAAGCACATGAATCAGCTTCCTTTAGGTTGACTGTGTTAGCTGTTGATGGGTCCCAGAATGGACAGACTGTTGTTGAGATCGAGATATTGGACATTAATGATAATGCACCAGTGTTTGCCCTTCCGGAAGATCAGGTGGATGTTGTTGAAAACTGGGCTGTTGGAAACAAGTTCTATGCAGCACAAGTCACTGATGCAGACAGTCCCCCAAACAATGTGATCCTGTATGATTTATCTGTAAACTTTGATGACCGTTTTGGCATCAATCATACTTCTGGAGTGCTGTTCTTGAACAAGGATCTGCAAAACTACCCAGAGAAGGAGTATAATCTGCAAATCCAGGCAACGGACTA

**Probe 2**

GCAGCAGAACAACAGCAATCCTTCTAGCAACTCCCTCATGATGGAAAATGACACAGGAGAGCATGTTAAATTTGAAATGGAAAACCTTAGACCACGTGAATTGGATGAAGCAGAGCACTACGATCTAGAGAATGCGAGTAGTTTGGCAGCATCGGATATAGATGTTGCCTATCACTATAAACACTTCCACGACCAAGGAAATAGGAATCGTAGGAAGAAAAAGCATAGGCAGAACCAGAACCCAATGCTTGCCAGGATCCAGGCATCGCCAGCCAGACTGAGTCCAGTCAGCATTGGGTCTCATCATAATCCCAACCCATTAGGAAGTGAGCTAGCACACAGTACCCCATTAGATAGTCAAAACGCCATGCTAAGACGTAGCCCAGGACTCCATTCCAATTATAGTGCAAATCCGACGCTGCGCATGGGATCCATTCCAAGCAGTGGCCGTGCAACTCCTGCCAGAAACATAAGCAGTCCTGTCAACTCAGACCGTAGCTTCCAGAGCGAGCTACGCAGCCAAGCTCATCGCTCCGACGTGAGCAGTCTCCGGAGCGGGGGTAAACCAGCTAGAATGACAACACCCCTGTCGCACGATGGCAAAAGCCAACCGCATTCAAAGGGAAGGACTAGTAAAAGCCCCTTGGTTGTGGGATTGACAGCTGAAGAAGTAGCTCAGCTGAACACAGCAAGGCCTGACCTAATGTCAGGCAGCCATGCTAGCACTATTGAAGACCTATCGTCCAACAGCAGCCGGGTTCATGGTGGAGAGCGCCCCATCGATACTCCATGTGATCCATCGCGTTTGCTAGAACCTCCCGATTCCACATCGGACGATACAAATGACTCCTTTACCTGCTCTGAGATGGACTCAGAGTATGGAAAACATGGAGGATTCAACAGCACCGAGGCTGCAATACTCGATAGACTTGCTGAAATCGAGCATGCGGAGGATAGCGTGCTTCCTCATGTGAATGGTGCCCTTAAACAGAAGCGGCTCGA

**3) Sp-dchs2**

**Probe 1**

AGGGAAACGGGAGTCTACATGGATATTGTCGTCAATAAAACTCTTGATCGAGAAACAATCTCCATGTACACCTTGATCATTGAGGCTTATGATGGAGGAGACCCACGGCTCACTGCAAATACCACAGTCAATATCAAAGTAACCGATGTGAATGACAACTCACCAGTATTCACCTTAACTTCCTTTACTGCTACCATCAATGAGAGCGCTCCTGTAAACACGACTGTGCTTCAGGTAACAGCATCTGACGAGGATGAAGGTTTGAATGGGGAGGTTGTCTTCAATCTACAAAGAGACGACGGCTTCTTCAGAATTGACCCTGCCTCTGGAATAGTATATCTTAACAAGGTCCTGAACTATGAAGATGCCCAGGCCCATGATTTTCTAGTCATAGCTCAAGATAAAGCGGAACCTCCCATGTCCAGCCAGCCTGCTTATGTTTCCATCATGGTTATCAACATCAATGAAAAACCCCCTTCTCTTGAGGTTCTGTTCCTTCCTGATGGAGGAGCATCCCAAGTCTCGGAAGCAGCGGTTGCTGATACCACCCTTGCCCGCATCTCCGTCACCGACCCTGATGACGGTGTCCTCACTAATGTCTCCATGACTATCACAGGTGGTGCTGGTCAGTTTGACCTCCAAAAGAATAGCAATCAAGTCTACTTTCTGATTGTGGCTACTACAGCAAGTTCTTTTGACCGTGAGGAAGTCGCAAGTTACGACATTTCAATTCGAGCAACCGATCACGGTTCCCCGCCACAGCATGCTGAGGAGAACCTGACCATTGCAGTGACCGACGTTAATGATAACCCACCTATCTTTGACCTTCCCTTGTACCATGCGACCATCATCGAGGCGTCCGAACCTGGTACCCCGGTCAAGCAGGTCCATGCAACGGATGCTGACGAAGGAGTCAACGCCCAGATCATCTACAGGATATCTCCTGAAGGCACTGACTACTCTGACTGGTTTGAAATCAATCCCATGTCAGGGC

**Probe 2**

GCTTGTGGTCGCTGAAGATAGGGATGCCAGAGAATTTGGCCGTGTGACCTATACCATCGACTCCAGCACCAACGATGGGACATTTGCTATCAACCAAACTACAGGTCTTATCACCCTGGTTGGTTCCTTAGACTGGGTCCAGCATCCAGTCTACACCCTGGAAGTCATTGCTACCGACGGGGGCGGTCGTACCTCCGCCAACCACGCCCAGGTCACCATCACGGTGGCCGGGCCAGACACGTCGCCCCCGGAGTTTGAATCCTCGTCCTATGCATTTTCCGTTCAGGAGAACGCGGCTCAGGACCTTGTCGGTGCAGTCAAAGCAGGGCATATTGATCCAGGTATCACCTTTCCAATAACCTACACGCTGACATCTGGTGATCCTAATGACTTCTTCCGGATCAACACTACCAGTGGGGAGATATTTACAAGAGGTTCTGGGCCTGATCATGAGCAGCAAGCTTTTGTCCTCATCACCATCCAGGCAGCCAGTGGAATGCCTCCTACCTACGACGATGTCCAGGTGAACATCACCGTCATTGATGTCAACGACAACGTCCCACAGTTCATGGCGCAGACTGCGCGTACAAGCATTCCAGAGAGCACGGGAATCGGAAGTTCTATCTATGTCGCTACAGCAATGGATCTGGACAGTGGTGATAACGGATTGGTGAGGTATCGTCTGACGTCGAATCCAGGGAACACGTTCAGGATAGTTGAGGCAACTGGTGAGATCATCTTAAATAAGGAGGTGAGCTACAACGATCAGCTAAGCTTTGAGGTGGACATCCAGGCATATGACGCTGGTGAGCCATCGCTCGAAGGTAGCCTGACCCTCACCGTCATCATCCTCGATGTGAACGATAATGGTCCCGTCTTCACCATGGGTTTCTACCCGGTATCTATTCTTGAAAACCTACCAATCAGCACCCCTGTCGTTCAGGTCGAAGCAACTGACAATGACCGCGGCGCAAACGCCCTCATCACCTAT

**4) Sp-pcdh9**

**Probe 1**

AAGCCTCGCTTGACCATATCAGCTCTCGGAGATGGCGGAAGATTCAAACACATTGCAGAAAACTCCCCTGAAGATGTAGACGTTGCGTATGTTCGTGTCACTGATATGGATACCGGAGTCAATGGCCAAGCTATATTGACATTAGAAGATGACTTTGGACACTTTTACTTAGAATCATTCAGAGAAGGCCAGTATTTCCTCAAGACAGCAGGCGTATTGGATCGCGAAGACATTGATTTTTACAACATCACCATTCTCGCTGAAGATCGAGGATCCCCTGTGCTCTCGTCACGGAGACGGTTTGCAGTGTTCGTTGATGACGAGAACGACAATTCTCCCATATTCTCGTCGTCCGTTTATCACGCTACGATTAGTGAAAACAACGAGCCGGGTCACCGCGTGGCTACCGTACAGGCTATCGACAAAGACGAGTTAGAGAATGGCGAAGTTGTATACAGCCTCCTAGATGATAAAGATGGTTCCTTTGGGATCCATCCATTCAATGGAGTCCTCACTGCTAATGTAAGTCTTGATAGAGAAGACGGGGAGTCCATTGACCTCATGATCAGGGCCTGTGACCGTGGACAACCACAGGGATGTTCAGATGTACCTTTGACAGTTAGGGTTCTGGATATGAACGACAACGGCCCCACGTTTGGCGGGGACTTAATCGAGATGAGAATAGATGAAAATAAACCAATTGGTACTATCGTCGGTCGAGCCATAGCTACAGATGCTGATGAAGGAGATAATGGTCGACTGAGGTACAGTATCTTGACTGATGCTGTGTTCAGGATTGATGAAGATAGCGGAAGGATCTACTCAACAGC

TGAACTTGATCGGGAGATTCAAGAGCTTTATCACTTTACAGTACGCGCAGTCGACGACGGATTGTCCCCCAAAACGGCTACTGCCACAGTGGTCGTGACTGTGAATGATGGGAACGACCATAGTCCAGAATTCACTGTGCCATCGGCAAACAATGACATTCGATTCATTCCAGTCTCTGCGG

**Probe 2**

AGAGATTGAGCGGGAAAGTGGTTATAGAACCAATCTATCTTCAGCTGATGGTAGCTCAAGGCGATCAGCTTCTTACCCGCGTATCGCAGCACTGGCAGCACAAAATATCAACAATACCCAAACTCCGTCCGGAGGCCACCGTCATAGCTACATGGCCAGCCCTCACTGTACACCTGAATGTTTGACCCTAGGTCACTCAGACGAGTGCTGGATGCCCAACCCAGCATCTATCAGCAAGTCAAAGACTGTTCATTTCTCCAAGCACGACGATAACCGGTCATCAGGCGGTAGCTACCGCAGCTATCACTCCAACGCGGACCGTCCTCCAGCAACAAGCCCCAAACCACCCAACCCTGCCATCGCCTTCGGTAACGTCTCTAAGGTTACCCCGAAGCCCAAACCTATCCCCAACGGTTCGACCACCGCTGGACTGGGGCAGCAACCGCCTGCAACGAGAGACCTCAACGGTTACTATGGTAATAACCCTGCAGTCATTGTAGAAGACGAACGAAATGGGAACGCAATGCCATTAACTCCGATCAGAGAACATCCCTTCGAAAGAAGAAGTTCGCAAGATGCTCCTAGCCTTGTCGTTACAAAGCATTCTCCAGACAAATACAAAAATAACAACGACGTCGATAGAAACGGCGACGTCAGATATCGTAACGACCTCAACAGAGGACATATAAACGGTGTAAACCCATTGCATAGAAACAGTATGAACGGTACCACACCAAACGGTAGCACCCCGAACGGCGGCCAACTGAATGGCATACAGAATAGTAGGAGTAATAACAGTAGGAACTCTAGTGGTGACAGTCATATTTCGAGCGAGGATAGCGCAGACACGTACTCGGGTTCGTCGGCCGCGAGCAGCGAAATGAAACCAGTTCGTTACTCGCCTGACGAGGTCGAGGCTGTCCTCGAGAAATGCGGAGGAGATCGATCAGCGGGAGTATCGGCAGACGGCGATAGGATGTACACGGATTGGGTATAACACGCGGT
